# Mitovirus and Mitochondrial Coding Sequences from Basal Fungus *Entomophthora muscae*

**DOI:** 10.3390/v11040351

**Published:** 2019-04-17

**Authors:** Max L. Nibert, Humberto J. Debat, Austin R. Manny, Igor V. Grigoriev, Henrik H. De Fine Licht

**Affiliations:** 1 Department of Microbiology and Program in Virology, Harvard Medical School, Boston, MA 02115, USA; austinmanny@g.harvard.edu; 2 Instituto de Patología Vegetal, Centro de Investigaciones Agropecuarias, Instituto Nacional de Tecnología Agropecuaria (IPAVE-CIAP-INTA), Córdoba X5020ICA, Argentina; debat.humberto@inta.gob.ar; 3 U.S. Department of Energy Joint Genome Institute, Walnut Creek, CA 94598, USA; ivgrigoriev@lbl.gov; 4 Department of Plant and Microbial Biology, University of California Berkeley, Berkeley, CA 94720, USA; 5 Department of Plant and Environmental Sciences, University of Copenhagen, DK-1871 Frederiksberg, Denmark; hhdefinelicht@plen.ku.dk

**Keywords:** database mining, *Entomophthora*, *Entomophthoromycotina*, fungal virus, mitochondrion, mycovirus, virus discovery, *Mitovirus*, *Narnaviridae*

## Abstract

Fungi constituting the *Entomophthora muscae* species complex (members of subphylum *Entomophthoromycotina*, phylum *Zoopagamycota*) commonly kill their insect hosts and manipulate host behaviors in the process. In this study, we made use of public transcriptome data to identify and characterize eight new species of mitoviruses associated with several different *E. muscae* isolates. Mitoviruses are simple RNA viruses that replicate in host mitochondria and are frequently found in more phylogenetically apical fungi (members of subphylum *Glomeromyoctina*, phylum *Mucoromycota*, phylum *Basidiomycota* and phylum *Ascomycota*) as well as in plants. *E. muscae* is the first fungus from phylum *Zoopagomycota*, and thereby the most phylogenetically basal fungus, found to harbor mitoviruses to date. Multiple UGA (Trp) codons are found not only in each of the new mitovirus sequences from *E. muscae* but also in mitochondrial core-gene coding sequences newly assembled from *E. muscae* transcriptome data, suggesting that UGA (Trp) is not a rarely used codon in the mitochondria of this fungus. The presence of mitoviruses in these basal fungi has possible implications for the evolution of these viruses.

## 1. Introduction

The classification scheme for kingdom *Fungi* currently applied by the National Center for Biotechnology Information (NCBI; Bethesda, MD, USA) includes eight major phyla: *Ascomycota*, *Basidiomycota*, *Mucoromycota* (subphyla *Glomeromycotina*, *Mortierellomycotina*, and *Mucoromycotina*), *Zoopagomycota* (subphyla *Entomophthoromycotina*, *Kickxellomycotina*, and *Zoopagomycotina*), *Blastocladiomycota*, *Chytridiomycota*, *Cryptomycota*, and *Microsporidia*, in approximate order of increasing time since they emerged as divergent taxa [1,2]. Fungal mitoviruses have been reported to date only from the most recently (apically) diverging phyla, *Ascomycota* and *Basidiomycota*, as well as from subphylum *Glomeromycotina* in phylum *Mucoromycota* [3,4,5]. We remain interested to discover fungal mitoviruses from other, less recently (basally) diverging phyla or subphyla, as part of an effort to understand the deeper evolutionary history of these viruses.

Mitoviruses are currently classified in genus *Mitovirus*, family *Narnaviridae*. They have small plus-strand RNA genomes and replicate persistently in host cell mitochondria [3,6,7,8]. Though initially reported only from fungi [3,4,5], they have recently been found also in plants [9,10]. Several mitoviruses reported also from invertebrates [11,12] might instead have been derived from fungal symbionts of the sampled animals. Mitovirus genomes range between 2.0 and 4.5 kb in length, each encompassing a single long open reading frame that encodes a deduced protein with the conserved motifs of a viral RNA-dependent RNA polymerase (RdRp) [13]. Mitoviruses do not form virions and are thought to persist and replicate instead as ribonucleoprotein complexes inside infected mitochondria, which are transmitted to daughter cells during cell division, as well as horizontally during hyphal anastomosis in the case of fungal mitoviruses and vertically through spores or seeds in the case of fungal or plant mitoviruses, respectively [3,8,9,10,14]. Mitovirus infections in fungi have been linked to morphological abnormalities in mitochondria, defective in vitro growth, and hypovirulence in some phytopathogenic fungi [8,15,16,17,18]. In fact, a recent report based on exhaustive phylogenetic analyses of RNA-dependent polymerases suggests that mitoviruses represent one of the most phylogenetically basal groups of eukaryotic RNA viruses [19].

*Entomophthoromycotina* is one of three subphyla that currently constitute fungal phylum *Zoopagomycota* [1,2]. A few entomophthoroid fungi that are normally soil saprobes can also cause human infections, including *Conidiobolus coronatus* (order *Entomophthorales*) and *Basidiobolus ranarum* (order *Basidiobolales*) [20,21]. Entomophthoroid fungi are probably best known, however, as insect parasites, which exhibit generally narrow host ranges and can cause epizootic events in which large numbers of susceptible insects are killed within local geographic regions [22]. The *Entomophthora muscae* species complex (order *Entomophthorales*) [23] is one such group of entomopathogenic fungi, which infect and kill their dipteran, commonly muscoid fly, hosts. *E. muscae* is also known to manipulate fly behaviors, in particular inducing the behavior known as “summiting”, in which the fly climbs to a high surface where it becomes affixed via fungal outgrowths and then strikes a characteristic pose considered to aid in dispersal of spores from the subsequent carcass [24].

De Fine Licht et al. [25] have reported on the transcriptomics of *E. muscae* isolates, in a broad effort to understand their host specificity and pathogenicity. In that report, the authors mention the presence of several apparent viral transcripts in their transcriptome shotgun assemblies but provide no further descriptions of those viruses. As described in detail below, we subsequently discovered a number of apparent mitovirus sequences from that transcriptome study by searching for mitovirus-like sequences within the Transcriptome Shotgun Assembly (TSA) database at NCBI. Recognizing several interesting features of these apparent new mitoviruses from *E. muscae*, we have gone on to characterize their sequences in detail, as we report here. As a part of this work, we have also identified additional strains of these mitoviruses by assembling sequence reads from two other transcriptome studies of *E. muscae*, as obtained from the Sequence Read Archive (SRA) database at NCBI. Included among our findings is identification of numerous UGA (Trp) codons in the mitovirus sequences from *E. muscae*, as well as in mitochondrial core-gene coding sequences from *E. muscae*, leading us to conclude that UGA (Trp) is not a rarely used codon in *E. muscae*, unlike the case in many other basal fungi [4]. *E. muscae* is hereby the first fungus from phylum *Zoopagomycota*, and also the most phylogenetically basal fungus [1,2], that has been reported to harbor mitoviruses to date, with possible implications for mitovirus evolution.

## 2. Materials and Methods

### 2.1. Assembly and Analysis of Mitovirus Sequences from E. muscae

The main steps in this process are detailed in Results. All searches of the TSA, SRA, and NR databases were performed using the BLAST web server at NCBI. The 15 TSA hits from the initial TBLASTN searches were: GEMZ01003603.1, GEMZ01006022.1, GEMZ01006112.1, GEMZ01008256.1, GEMZ01008924.1, GEMZ01011847.1, GEMZ01008924.1, and GEMZ01021189.1; GENA01003603.1, GENA01006022.1, GENA01006112.1, GENA01008256.1, GENA01008924.1, GENA01011847.1, GENA01008924.1, and GENA01021189.1; and GEND01018947.1 [25]. The mitovirus queries for those searches were: BAJ23143.2, BAN85985.1, AVA17449.1–AVA17452.1, and AXY40441.1–AXY40444.1 [5,26,27,28]. To identify mitovirus-matching reads in SRA accessions prior to contig assembly, searches were performed using Discontiguous MegaBLAST or BLASTN. Sequence reads were then assembled into contigs using CAP3 [29] as implemented at http://biosrv.cab.unina.it/webcap3/ or galaxy.pasteur.fr/. Terminal residues in each assembled contig were trimmed back to those that agree between at least two mitovirus strains of the same species. Following contig assembly, mapped reads were re-identified using MegaBLAST, and coverage depths were determined using the “Map Reads to Reference” tool in CLC Genomics Workbench v7. RPKM and coverage depth values are shown in Table S1.

For generating transcriptome shotgun assemblies from SRA libraries, we used Trinity v2.2.0 [30] with default parameters as implemented through https://usegalaxy.org/ and rnaSPAdes v3.13.0 [31] with default parameters as implemented locally. The contigs from each Trinity assembly were subjected to searches with TBLASTN using mitovirus RdRp sequences as queries. Identified contigs were then extended by iterative mapping of reads from the corresponding BioProject using the “Map to Reference” tool in Geneious v8.1.9 with low-sensitivity parameters. The contigs from each rnaSPAdes assembly were subjected to taxonomic classification using locally implemented DIAMOND v0.9.22.123 [32] in BLASTX mode against the NCBI NR database, with an E-value threshold of 1e−3 and a “top” parameter of 1 to identify the best species-level hits for each contig.

Codon usage was analyzed using the Codon Usage tool in Sequence Manipulation Suite at http://www.bioinformatics.org/sms2/. Pairwise and multiple sequence alignments were performed using MAFFT v7 [33] as implemented at https://mafft.cbrc.jp/alignment/server/. For determining % sequence identity, pairwise alignments were performed using EMBOSS Needleall as implemented at http://www.bioinformatics.nl/cgi-bin/emboss/needleall. Maximum-likelihood phylogenetic analyses were performed using the program IQ-Tree [34], including the “Find best and apply” option (ModelFinder) [35] and the “Bootstrap ultrafast” option (UFboot) [36], as implemented at https://www.hiv.lanl.gov/content/sequence/HIV/HIVTools.html.

### 2.2. Assembly and Analysis of Mitochondrial Gene Coding Sequences from E. muscae

Programs were used as described above for mitovirus sequences. The following TSA hits arose from the initial TBLASTN searches for mitochondrial gene coding sequences from *E. muscae* and were then used as queries of the SRA transcriptome libraries from *E. muscae* isolate Berkeley: *atp6*, GENA01019820.1; *atp9*, GENA01019993.1; *cob*, GEND01033240.1; *cox1*, GEND01036136.1 and GEND01034538.1; *cox2*, GEND01003800.1; *cox3*, GEND01033140.1; *nad1*, GENA01026764.1 and GENA01028096.1; *nad2*, GENA01003576.1; *nad3*, GENA01020377.1; *nad4*, GEND01006792.1 and GEND01006791.1; *nad5*, GENA01001061.1; and *nad6*, GEND01032932.1 and GENA01021432.1 [25]. RPKM and coverage depth values are shown in Table S2.

### 2.3. SRA Accessions Used for Assembling Contigs

SRA accessions that we used in this study for assembling mitovirus or mitochondrial transcript contigs from each *E. muscae* isolate are listed in Table S3. For *E. muscae* isolate KVL-14-117, the SRA accessions only from media-grown fungus (not fly-grown fungus) were used for assembling mitovirus contigs, in an effort to ensure that the identified viruses were derived from *E. muscae*, and not the fly hosts or a different fly symbiont. For *E. muscae* isolate Berkeley, the SRA accessions from only (i) flies infected with *E. muscae* (not uninfected flies) and (ii) later times post-infection (72, 96, and 120 h, not 24 and 48 h) were used for assembling mitovirus or mitochondrial transcript contigs, in an effort to increase the proportion of reads that were derived from *E. muscae*, not the fly hosts.

### 2.4. Newly Reported Sequences

Coding-complete mitovirus sequences from *E. muscae* isolates KVL-14-117, KVL-14-118, HHdFL130914-1, HHdFL050913-1, and Berkeley have been deposited in GenBank as accessions MK682513.1–MK682534.1 and BK010729.1–BK010736.1. Mitochondrial core-gene coding sequences from *E. muscae* isolate Berkeley have been deposited in GenBank as accessions BK010748–BK010759. In addition, the mitovirus and mitochondrial core-gene sequences from all five of these *E. muscae* isolates have been included in the Supplementary Materials for this report as Data S1 and Data S2, respectively.

### 2.5. Sequencing at the Joint Genome Institute, 1000 Fungal Genomes Project

The sequence reads from *E. muscae* isolate HHdFL130914-1 (accession SRX2782457) have not been reported in a peer-reviewed article to date, and the methods are therefore described here. A stranded cDNA library was generated using an Illumina TruSeq Stranded RNA LT kit (San Diego, CA, USA). mRNA was purified from 1 µg of total RNA using magnetic beads containing poly-T oligos. mRNA was fragmented and reverse-transcribed using random hexamers and Superscript II (Invitrogen, Carlsbad, CA, USA) followed by second strand synthesis. The fragmented cDNA was treated with end repair, A-tailing, adapter ligation, and eight cycles of PCR. The prepared library was quantified using KAPA Biosystem’s next-generation sequencing library qPCR kit (Wilmington MA, USA) and run on a Roche LightCycler 480 real-time PCR instrument (Pleasanton CA, USA). The quantified libraries were then prepared for sequencing on the Illumina HiSeq platform using an Illumina TruSeq Rapid paired-end cluster kit. Sequencing of the flow cell was performed on the Illumina HiSeq2500 sequencer using Illumina HiSeq TruSeq SBS sequencing kits, following a 2 × 150-nt indexed run recipe.

## 3. Results

### 3.1. Mitovirus-like Sequences in Transcriptome Data from E. muscae

We used TBLASTN to search entries from basal fungi (not phylum *Ascomycota*, phylum *Basidiomycota*, or subphylum *Glomeromycotina*) in the TSA database at NCBI, using RdRp sequences of previously reported fungal mitoviruses as queries. The searches yielded 15 strong hits (E-values, 5e−52 to 2e−10 with different queries; see Materials and Methods for further details), all derived from the same transcriptomics study of muscoid-fly pathogens from the *E. muscae* species complex (BioProject PRJEB10825) [25], including *E. muscae sensu stricto* isolates from house flies (*Musca domestica*) and *E. muscae sensu lato* isolates from cabbage flies (*Delia radicum*). The lengths of these 15 hits range from 2321 to 2813 nt, within the expected interval for mitovirus genomes [3,4].

Before analyzing these apparent new mitovirus sequences, we examined the TSA metadata to learn about the samples and to identify the SRA accessions from which the TSA hits had been assembled. After reviewing the metadata and the sequences themselves, we chose to reassemble the mitovirus-like contigs for each *E. muscae* isolate, using the SRA-derived sequence reads that mapped to each respective TSA hit and including any additional reads that lengthened or shortened each contig. We were thereby able to reassemble seven distinct mitovirus-like sequences from media-grown *E. muscae* house fly isolate KVL-14-117, seven distinct mitovirus-like sequences from fly-grown *E. muscae* house fly isolate KVL-14-118 (one of the seven sequences includes two small gaps in sequencing coverage, as inferred by alignment with related sequences from other isolates), and one mitovirus-like sequence from fly-grown *E. muscae* cabbage fly isolate HHdFL050913-1. A small number of reads (22 total) matching the mitovirus from isolate HHdFL050913-1 were also identified from fly-grown *E. muscae* cabbage fly isolate HHdFL040913-2 [25] but were sufficient in coverage to assemble only a few short contigs.

**Figure 1:**
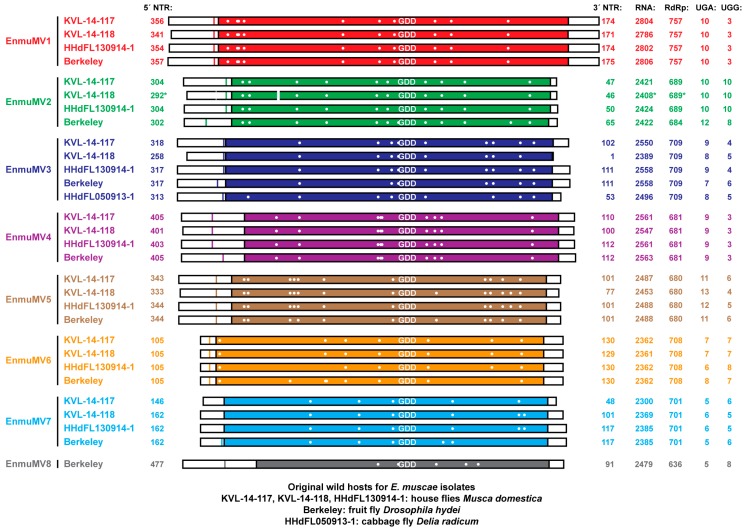
Summary diagram of mitovirus sequences from *E. muscae* isolates. Mitovirus strains from five different *E. muscae* isolates, representing eight apparent new mitovirus species, are labeled at left. The RdRp-encoding open reading frame ORF is shown as a colored box within each assembled RNA sequence (see matching colors in Figure 2), starting with the first in-frame AUG codon by convention. The position of the first in-frame stop codon upstream of the proposed AUG start codon is shown as a colored line. Positions of UGA (Trp) codons in each RdRp ORF are shown as white dots. RNA sequences are aligned with respect to the catalytic motif GDD in each deduced RdRp sequence. Positions of two small gaps in sequencing coverage in the assembled EnmuMV2-KVL-14-118 sequence are shown as white breaks in the boxes; asterisks indicate inferred values consequent to these coverage gaps. For each sequence, lengths of the 5´ NTR, 3´ NTR, and overall RNA sequence are labeled in nt; length of the RdRp sequence in aa; and UGA and UGG codons in raw counts.

By reviewing the SRA database for other transcriptome-associated accessions that are annotated as relating to *E. muscae* or other *Entomophthoromycotina* members, we found such accessions from five other BioProjects: PRJNA372837 for *E. muscae* house fly isolate HHdFL130914-1 (also known as KVL-14-115 [37]), PRJNA435715 for *E. muscae* fruit fly isolate Berkeley (originally obtained from *Drosophila hydei*) [24], PRJNA259024 for *Conidiobolus thromboides* isolate FSU 785, PRJNA67455 for *Conidiobolus coronatus* isolate NRRL 28638, and PRJNA501640 for *Zoophthora radicans* isolate ATCC 208865/ARSEF 4784. We therefore used the newly assembled mitovirus-like sequences described above as queries to search the SRA transcriptome libraries from these five other BioProjects and obtained numerous strong hits from two of them, those for *E. muscae* house fly isolate HHdFL130914-1 and *E. muscae* fruit fly isolate Berkeley. Assembling these SRA reads from isolates HHdFL130914-1 and Berkeley then gave rise to seven distinct mitovirus-like sequences from each of these additional isolates, for a total of 29 mitovirus-like sequences assembled from five different *E. muscae* isolates (Figure 1).

Given the large number of mitovirus-like sequences associated with *E. muscae*, we reasoned that there might be sequences of yet other, divergent mitoviruses remaining to be identified in the SRA transcriptome libraries. To address this possibility, we generated our own shotgun assemblies from the libraries analyzed in the preceding paragraph and then searched these new assemblies for other mitoviruses. In this manner, we were able to identify one additional mitovirus-like sequence from *E. muscae* isolate Berkeley (sequence length, 2479 nt) (Figure 1), bringing the overall total to 30 such sequences from the five *E. muscae* isolates.

Especially in plants, fragments of mitovirus genome sequences have been commonly endogenized in host DNA [9,38,39,40]. To address this possibility for the mitovirus sequences reported here, we noted that there are six SRA libraries at NCBI from PacBio SMRT runs on genomic DNA from *E. muscae* isolate HHdFL130914-1 (BioProject PRJNA346904), an isolate that we showed above to harbor sequences from seven mitovirus strains in its RNA transcriptome data. These six SRA libraries from *E. muscae* DNA were found to register no MegaBLAST hits to the seven mitovirus sequences, providing evidence against these virus sequences being derived from endogenized elements. A small number of mitovirus-matching reads (28 total) found in three SRA libraries arising from Illumina runs on genomic DNA from the same BioProject as the PacBio SMRT runs seem unlikely to be significant, though they may be reminiscent of evidence for non-genomic DNA fragments detected for *Gigaspora margarita* mitoviruses [28]. Contigs for *E. muscae* mitoviruses found as hits from the Whole Genome Shotgun (WGS) database at NCBI are in fact derived from the transcriptome study BioProject PRJEB10825 [25], and thus from RNA not DNA (H.H.D.F.L.).

### 3.2. Basic Features of the Apparent Mitovirus Sequences from E. muscae

The lengths of our 30 newly assembled mitovirus-like contigs range from 2300 to 2806 nt (Figure 1), within the expected interval for mitovirus genomes [3,4]. Using genetic code 4 in which UGA encodes Trp not “stop” (as expected for translation in the mitochondria of most animals and fungi including members of phylum *Zoopagomycota* [41,42,43]), a single long open reading frame (ORF) is found in each contig, flanked by one or more stop codon at the plus-strand 5´ end in 27 of the 30 sequences and one or more stop codon at the plus-strand 3´ end in all 30 sequences, suggesting to us that all of the contigs are coding complete. The contigs encode proteins of nine different lengths (assuming that the first in-frame AUG is the start codon in each): four encode a 757-aa protein, five encode a 709-aa protein, four encode a 708-aa protein, four encode a 701-aa protein, three encode a 689-aa protein, one encodes a 684-aa protein, four encode a 681-aa protein, four encode a 680-aa protein, and one encodes a 636-aa protein, suggesting that there may be multiple mitovirus species represented by these sequences. When used in BLASTP searches of the Nonredundant Protein Sequences (NR) database for Viruses at NCBI, each of these deduced protein sequences showed strongest similarities to mitovirus RdRps, and the approximate position of the RdRp catalytic motif GDD is shown and centered for each in Figure 1 (also shown in the multiple sequence alignment of the eight sequences from *E. muscae* isolate Berkeley in Figure S1). For nine contigs, the top hit was to the Erysiphe necator mitovirus 1 (ATS94398; E-values, 1e−91 to 8e−82; identity scores, 37–40%); for nine other contigs, the top hit was to Erysiphe necator mitovirus 2 (ATS94399; E-values, 1e−94 to 5e−70; identity scores, 34–38%); for eight other contigs, the top hit was to Erysiphe necator mitovirus 3 (ATS94400; E-values, 0.0 to 5e−119; identity scores, 36–45%); and for the remaining four contigs, the top hit was to Hubei narna-like virus 25 (APG77157; E-values, 0.0; identity scores, 90%), which is also an apparent mitovirus [10]. These BLASTP findings suggest again that there may be multiple mitovirus species represented by these sequences. The mitovirus-like contigs may be missing some residues at one or both ends relative to the full-length viral genomes, but even so, there is a generally long nontranslated region (NTR) at the plus-strand 5´ end of each contig (105–477 nt, median 317 nt; again assuming that the first in-frame AUG is the start codon in each) and a generally shorter NTR at the plus-strand 3´ end of each contig (1–175 nt, median 106 nt) (Figure 1).

Most fungal mitoviruses contain a number of in-frame UGA codons in the RdRp open reading frame, encoding Trp per genetic code 4; however, some do not, and almost all of those that do not derive from fungal hosts from phylum *Basidiomycota* or subphylum *Glomeromycotina* in which UGA(Trp) is a rarely used mitochondrial codon [4,28]. The 30 mitovirus-like sequences from *E. muscae* described above fit the more typical pattern for fungal mitoviruses, in that a substantial fraction of their Trp codons are UGA (vs. UGG): 38–77%, numbering between 5 and 13 UGA(Trp) codons in the different apparent mitovirus sequences (Figure 1).

### 3.3. Pairwise and Phylogenetic Comparisons of the Mitovirus Sequences from E. muscae

**Figure 2:**
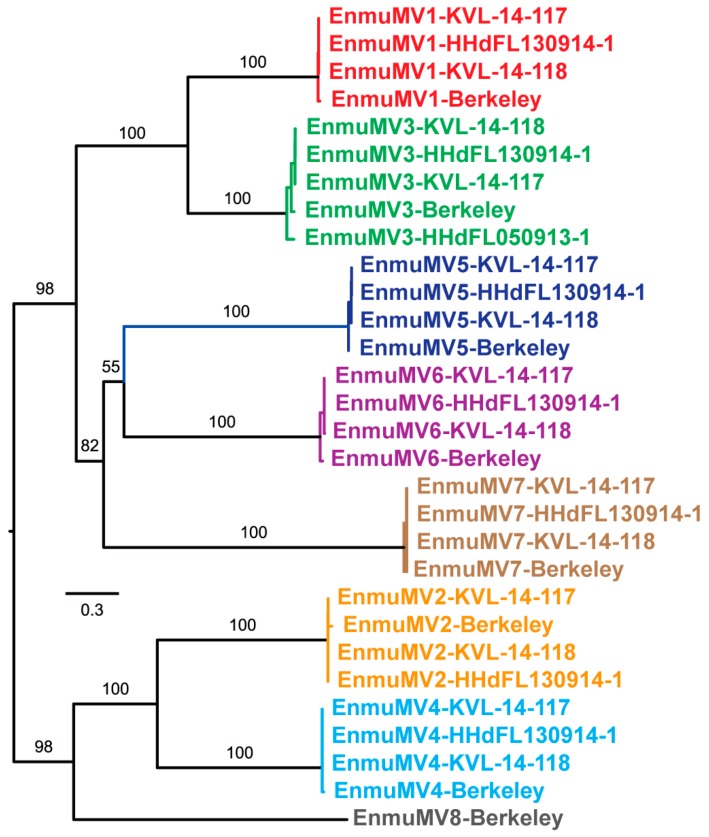
Maximum-likelihood phylogenetic analysis of *E. muscae* mitoviruses. Deduced RdRp sequences were aligned using MAFFT-L-INS-i. The best-fit (per BIC score) aa-substitution model used for the phylogenetic analysis shown here was LG+I+G4. The tree is displayed as a midpoint-rooted rectangular phylogram with branch support values from UFboot (1000 replicates) shown in %; support values for distal branches have been deleted for visual clarity. Scale bar indicates average number of substitutions per alignment position. Labels of different colors highlight the clustering of strains of eight different mitovirus species suggested by these results, as well as by the results in Figure 1 and Figure S2.

Features of the mitovirus-like contigs described above led us to suspect that there are multiple new mitovirus species represented by these sequences. To address this possibility, we performed pairwise comparisons of the nt sequences using EMBOSS Needleall. These comparisons grouped the sequences into eight distinct clusters, with pairwise identities >86% within each cluster and <50% between any two clusters (Figure S2). The degree of divergence between these clusters, as well as the low identity scores with previously reported mitoviruses found in the BLASTP searches described above (≤42%), led us to conclude that these eight clusters represent eight new mitovirus species. Phylogenetic analyses involving the *E. muscae* mitoviruses alone corroborated this conclusion by showing the distribution of the 30 viruses into eight well-delimited clades (Figure 2). In addition, by examining which members of each clade derive from which *E. muscae* isolate, we found that one of the *E. muscae* isolates (Berkeley; from fruit fly) harbors strains of all eight of the new mitovirus species, three of the *E. muscae* isolates (KVL-14-117, KVL-14-118, and HHdFL130914-1; from house flies) harbor strains of even of the new mitovirus species, and the remaining one isolate (HHdFL050913-1; from cabbage fly) harbors a strain of only one of the new mitovirus species (Figure 1 and Figure 2). We suggest the names of these new species to be “Entomophthora muscae mitovirus 1” through “Entomophthora muscae mitovirus 8” and further suggest the abbreviations EnmuMV1 through EnmuMV8 for the common names of these viruses. Six of these eight viruses (EnmuMV1, EnmuMV2, EnmuMV4, EnmuMV5, EnmuMV6, and EnmuMV7) are represented by four strains each, one of these viruses (EnmuMV3) is represented by five strains, and the remaining one of these viruses (EnmuMV8) is represented by only one strain in the results presented here. In fact, the few short mitovirus-like contigs assembled from fly-grown *E. muscae* cabbage fly isolate HHdFL040913-2 are sufficient to identify it as a sixth strain of EnmuMV3, most closely related to EnmuMV3-HHdFL050913-1 (95% nt identity), i.e., from another cabbage fly isolate of *E. muscae*.

**Figure 3:**
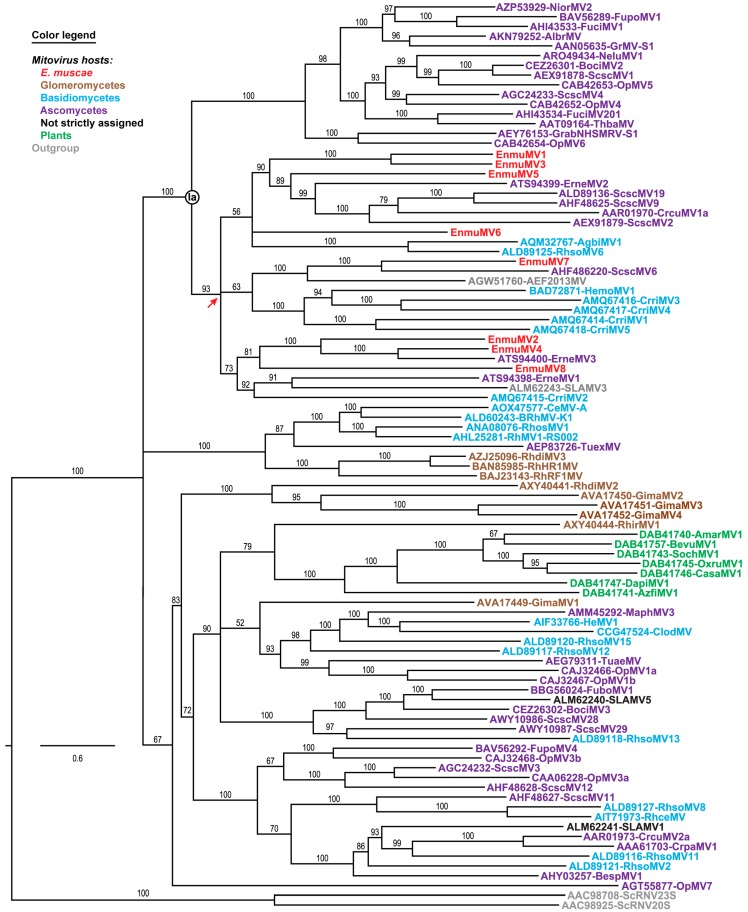
Maximum-likelihood phylogenetic analysis of *E. muscae* mitoviruses and representative other mitoviruses. Deduced RdRp sequences were aligned using MAFFT-L-INS-i. The best-fit substitution model (per BIC score) used for the phylogenetic analysis shown here was LG + F + R8. The site proportions and rates for the FreeRate model were (0.0224, 0.0123) (0.0271, 0.0755), (0.0627, 0.2087), (0.1161, 0.4202), (0.1784, 0.6877), (0.2655, 1.0570), (0.2163, 1.4069), and (0.1115, 2.0462). The tree is displayed as a rectangular phylogram rooted on a set of two members of current genus *Narnavirus* included as outgroup. Branch support values from UFboot (1000 replicates) are shown in %; branches with <50% support have been collapsed to the preceding node. Scale bar indicates average number of substitutions per alignment position. Previously identified clade Ia of current genus *Mitovirus* is labeled. Red arrow, most recent common ancestor shared by all eight *E. muscae* mitoviruses. Virus names matching the sequence accession numbers and abbreviations are provided in Table S6. Color-coding: mitoviruses from *E. muscae* (subphylum *Entomophthoromycotina*, phylum *Zoopagomycota*), red; members of subphylum *Glomeromycotina*, phylum *Mucoromycota*, brown; members of phylum *Basidiomycota*, cyan; members of phylum *Ascomycota*, purple; not assigned to a specific fungal host, black; plants, green; and outgroup, gray.

Phylogenetic analyses were next performed to ascertain the positions of the eight *E. muscae* mitoviruses relative to others in current genus *Mitovirus*. A representative set of 83 previously reported, full-length mitovirus RdRp sequences were obtained from the Protein database at NCBI and then used in multiple sequence alignments along with the eight mitovirus RdRp sequences from *E. muscae* isolate Berkeley, plus two RdRp sequences from members of current genus *Narnavirus* as an outgroup. Each multiple sequence alignment was analyzed using the ModelFinder module within IQ-TREE to identify the best-fit substitution model, which was then directly applied for maximum-likelihood phylogenetic analyses within IQ-TREE. As shown by the representative phylogram in Figure 3, the results reveal that EnmuMV1–EnmuMV8 (red) all cluster within the same discernible subclade of current genus *Mitovirus*, within a portion of the major clade previously designated Ia. This clustering suggests that all eight of these mitoviruses from *E. muscae* shared a most recent common ancestor, from which they all diverged, near the root of this subclade (red arrow in Figure 3), i.e., fairly early in mitovirus evolution but still well removed from the root.

By aligning the sequences of the different strains within each mitovirus species from *E. muscae*, we observed that the coding sequences for each species are marked by a total absence of indels among the strains, such that RdRp length is conserved within each species (Figure 1). One minor exception is that in EnmuMV2-Berkeley, there is a slightly earlier stop codon (UAA) resulting from a C-to-U substitution relative to the other EnmuMV2 strains, such that the RdRp length of EnmuMV2-Berkeley is reduced by 5 aa. In contrast, within the NTRs, several small indels are seen in the alignments among certain strains from the same species (EnmuMV1, EnmuMV2, EnmuMV3, and EnmuMV7), and especially in their 5´ NTRs (Figure S3). The putative functions of the NTRs in RNA replication, translation, etc., are thus presumably able to accommodate small indels in some positions better than the functions of the coding region and/or the encoded RdRp of each species.

### 3.4. UGA(Trp) Codons in Mitochondrial Core Genes of E. muscae

A previous report has revealed that UGA(Trp) is a rarely used codon in mitochondrial core genes of many basal fungi [4], including *Zancudomyces* (*Smittium*) *culisetae* from subphylum *Kickxellomycotina*, phylum *Zoopagomycota*, the only member of this basal phylum for which a complete mitochondrial genome sequence had been annotated as such at NCBI at that time (accession NC_006837.1) [41]. Simplistically, one might therefore expect UGA(Trp) to be a rarely used codon also in mitochondrial genes of subphylum *Entomophthoromycotina*, phylum *Zoopagomycota*. The evidence presented above for numerous UGA(Trp) codons in the mitovirus sequences from *E. muscae*, however, run counter to this expectation. To address this possible discrepancy, we sought to determine whether UGA(Trp) codons are found, too, in mitochondrial core genes of *E. muscae*. We first performed TBLASTN searches for transcript contigs representing mitochondrial core genes in TSA database entries for subphylum *Entomophthoromycotina*, using the deduced sequences of 14 mitochondrial core proteins from *Z. culisetae* as queries. Strong hits, all from the *E. muscae* transcriptome study BioProject PRJEB10825 [25], were obtained for 12 of the mitochondrial core genes: *atp6*, *atp9*, *cob*, *cox1*, *cox2*, *cox3*, *nad1*, *nad2*, *nad3*, *nad4*, *nad5*, and *nad6*.

**Figure 4:**
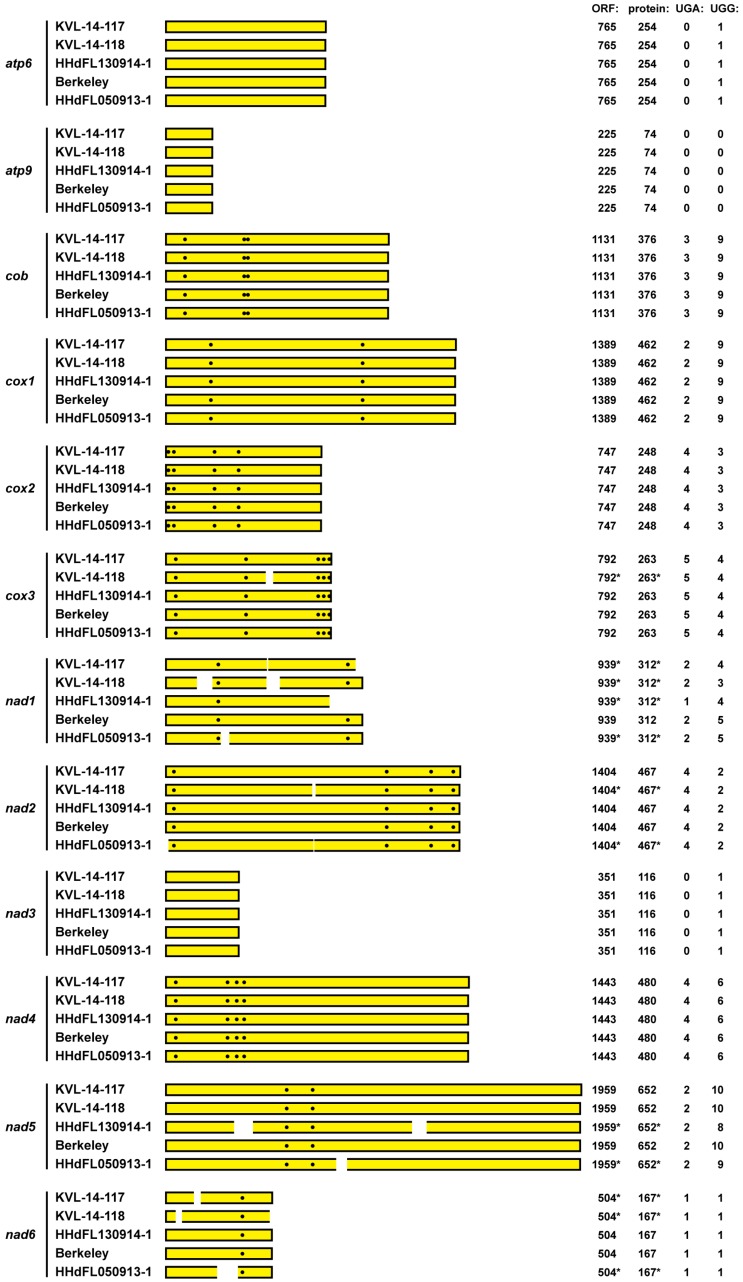
Summary diagram of mitochondrial coding sequences from *E. muscae* isolates. Mitochondrial core genes from each *E. muscae* isolate are labeled at left. The protein-encoding ORF of the respective transcript is shown as a yellow box to represent each assembled RNA sequence, starting with the first in-frame AUG codon by convention. Positions of UGA(Trp) codons in each ORF are shown as white dots. Positions of gaps or truncations in the assembled sequences are indicated by white breaks in the boxes; asterisks indicate inferred values consequent to these breaks. For each sequence, length of the ORF is shown in nt, length of the encoded protein in aa, and UGA and UGG codons in raw counts.

We next performed BLASTP searches of the NR database to discern which of the TSA hits for the 12 mitochondrial core genes appear to have originated from a basal fungus. We then used those TSA accessions (see Materials and Methods for accession numbers) as queries to search the SRA libraries from which the mitovirus sequences from *E. muscae* were assembled as described above, beginning with the SRA transcriptome libraries from *E. muscae* isolate Berkeley (BioProject PRJNA435715) [24]. Using the matching sequence reads that we identified, we were then ultimately able to assemble complete coding sequences for all 12 of these mitochondrial core genes from *E. muscae* isolate Berkeley (Figure 4). Additional BLASTP searches of the NR database strongly supported the conclusion that the mitochondrial protein sequences deduced from these contigs derived from a member of subphylum *Entomophthoromycotina*, phylum *Zoopagomycota*, given that the top-scoring one or two hit(s) for each protein consistently derived from *Conidiobolus* species *C. coronatus* or *C. heterosporus*, the latter for which a complete mitochondrial genome sequence has only recently been deposited and annotated at NCBI (accession MK049352.1) [43] (Table 1). Moreover, phylogenetic analyses using the concatenated sequences of these 12 mitochondrial proteins from *E. muscae*, along with those from other representative fungi, placed the *E. muscae* proteins adjacent to those of *C. heterosporus* within the *Zoopagomycota* clade (Figure S4).

To provide additional evidence that these transcript contigs from *E. muscae* isolate Berkeley represent the respective mitochondrial genes of *E. muscae*, we used the contigs to search the SRA transcriptome libraries from the four other *E. muscae* isolates KVL-14-117, KVL-14-118, HHdFL130914-1, and HHdFL050913-1. The results summarized in Figure 4 reflect that nearly identical coding sequences for all 12 mitochondrial genes were assembled from sequence reads from all four other *E. muscae* isolates. Complete coding sequences were assembled for eight to ten of these genes from each isolate and partial coding sequences for the other genes (Figure 4), with single-nt mismatches at only a few nt positions between any two isolates. These findings provide further strong support for the conclusion that these 12 mitochondrial coding sequences indeed derive from *E. muscae*.

We lastly examined the 12 mitochondrial coding sequences from *E. muscae* for UGA (Trp) codons. At least one and as many as five UGA (Trp) codons each are found in nine of these sequences, namely, in all but those for *atp6*, *atp9*, and *nad3*, which also contain no or only one UGG (Trp) codon each (Figure 4). Notably as well, these UGA (Trp) codons are conserved among the sequences assembled from the five different *E. muscae* isolates (Figure 4). Overall in these coding sequences, 35% of the Trp codons are UGA (vs. 65% UGG). We therefore conclude that UGA (Trp) is not a rarely used codon in the mitochondrial core genes of *E. muscae*, consistent with our findings for the mitovirus sequences from this fungus. Our initial generalization that UGA (Trp) might be expected to be a rarely used codon in the mitochondria of all basal fungi thus appears to have been incorrect.

## 4. Discussion

The eight mitovirus species newly identified in this report are the first to be discovered in a fungal host from phylum *Zoopagomycota* (subphylum *Entomophthoromycotina*). Before this, the most phylogenetically basal fungi [1,2] that had been found to harbor mitoviruses were ones from phylum *Mucoromycota* (subphylum *Glomeromycotina*) [5,26,27,28]. The mitoviruses in *Entomophthora muscae* might have been acquired by horizontal transmission, perhaps from some more apically diverging fungi given their juxtaposition with ascomycete and basidiomycete mitoviruses in the Figure 3 tree. The explanation that we favor, however, is that an early ancestor of the *E. muscae* mitoviruses first entered the fungal lineage in an ancestral species that predated the divergence of phylum *Zoopagomycota* from the three more apically diverging phyla *Mucoromycota*, *Basidiomycota*, and *Ascomycota*, and was then transmitted largely by vertical means over the subsequent course of fungal evolution, including to *E. muscae*. See Figure S4 for a fungal tree of life based on mitochondrial proteins, which shows a similar pattern of progressive divergence of the fungal phyla to that previously shown for nuclear products [1,2], and which was similarly shown for fungal mitochondrial proteins by Nie et al. [43] in their recent report on the complete mitochondrial genome sequence of entomophthoroid fungus *Conidiobolus heterosporus*. The reciprocal possibility, i.e., that *E. muscae* mitoviruses have been horizontally transmitted to some more apically diverging fungi, must also be considered given their juxtapositions in the Figure 3 tree. For example, note the apparent proximities of Erysiphe necator (ascomycete) mitovirus 1 (ATS94400) to EnmuMV4 and Sclerotinia sclerotiorum (ascomycete) mitovirus 6 (AHF48622) to EnmuMV7.

Four of the *E. muscae* isolates whose mitoviruses were characterized in this study were each found to contain strains of either seven or eight different mitovirus species. Although this large number might seem odd, similar findings have been made in other fungi. For example, strains of at least seven different mitovirus species have been found in the same isolate, Ld, of the Dutch elm disease fungus *Ophiostoma novo-ulmi* (phylum *Ascomycota*) [39,44,45]; strains of six different mitovirus species have been found in the same isolate, AG2-2-IV, of *Rhizoctonia solani* (phylum *Basidiomycota*) [46]; and strains of five different mitovirus species have been found in the same isolate, BC-u3, of the white pine blister rust fungus *Cronartium ribicola* (phylum *Basidiomycota*) [47]. Such multiple co-infections raise interesting questions about possible interactions among the different viruses in each isolate and whether there are selective forces or circumstances that favor multiple co-infections. The fungus *E. muscae* is multinucleate throughout all stages of its life cycle, and both protoplast cells and asexual spores may contain up to 20 nuclei each [25]. Although mitochondrial morphology and membrane potential dynamics are considered independent of nuclear cycle state [48], the consistent presence of multiple nuclei might facilitate a heterogeneous intracellular environment conducive to the presence of multiple mitoviruses. Another, more trivial possibility relates to the fact that the *E. muscae* isolates whose sequence reads were analyzed for this report originated not from individual spores, but from conidial showers from infected flies, meaning that there remains a possibility that these isolates are mixtures of *E. muscae* strains, with differences in mitovirus content between strains.

The findings in this report, including both mitovirus and mitochondrial coding sequences, suggest that the mitochondrial protein synthesis machinery in *E. muscae* can translate codon UGA (Trp) more efficiently than can that in many other basal fungi [4]. In this regard, *E. muscae* mitochondria are more like those of most members of the more apically diverging phyla *Ascomycota* and *Basidiomycota*. As noted in Results, UGA (Trp) has been shown to be a rarely used mitochondrial codon in *Zancudomyces culistae* (subphylum *Kickxellomycotina*, phylum *Zoopagomycota*; no UGA(Trp) codons in the 12 mitochondrial core-gene coding sequences examined here), such that there appears to be some variation in mitochondrial UGA (Trp) codon usage across members of phylum *Zoopagomycota*. In the recently reported mitochondrial genome sequence of *Conidiobolus heterosporus* (subphylum *Entomophthoromycotina*, phylum *Zoopagomycota*) [43], we find that UGA (Trp) is also somewhat uncommonly used (6 UGA (Trp) vs. 62 UGG (Trp) codons in the same 12 mitochondrial coding sequences, 8.8%), even though *C. heterosporus* encodes a mitochondrial tRNA sequence with anticodon UCA, which should allow for more efficient translation of UGA (Trp). A complete mitochondrial genome sequence for *E. muscae*, and for other members of subphylum *Entomophthoromycotina*, may be helpful for understanding why UGA (Trp) codons are more commonly used in the mitochondria of *E. muscae* than in those of related species *C. heterosporus* (same order, *Entomophorales*; different families, *Entomophthoraceae* and *Ancylistaceae*, respectively).

The RNA samples that were analyzed for *E. muscae* isolates KVL-14-118, Berkeley, and HHdFL050913-1 in the original transcriptome studies (BioProjects PRJEB10825 and PRJNA435715) [24,25] were obtained from infected host flies. One might therefore have some concern that the mitovirus sequences identified from those samples could have originated from the flies, or from a different fly symbiont, rather than from *E. muscae* itself. Importantly, however, the samples from *E. muscae* isolate KVL-14-117 analyzed in the original transcriptome studies (BioProject PRJNA372837) [25] and then further employed here were obtained from media-grown cultures of the fungus, serving to allay such possible concern. Indeed, the general absence of fly-derived sequence reads in the SRA libraries from media-grown *E. muscae* isolate KVL-14-117 [25] was confirmed in this study by use of several fly-derived mitochondrial gene queries. In addition, for *E. muscae* isolate Berkeley, the original transcriptome study (BioProject PRJNA435715) [24] included a number of samples from flies not inoculated with *E. muscae*, and the SRA libraries from those control samples contain no or very few sequence reads matching the *E. muscae* mitoviruses (Table S4). Moreover, the SRA libraries from the samples from inoculated flies in that study contain increasing numbers of sequence reads matching the *E. muscae* mitoviruses at increasing times after inoculation with *E. muscae* isolate Berkeley, consistent with the growth of *E. muscae* in those flies following inoculation (Table S4). Both of these observations provide strong evidence that the mitoviruses are associated with *E. muscae* isolate Berkeley itself, not the fly hosts. Lastly, we have been recently able to confirm the presence of all seven mitovirus sequences in new transcriptome data from conidial samples of *E. muscae* isolate HHdFL130914-1, which will be described in detail in a future article [49] and also provides important evidence for vertical transmission of the *E. muscae* mitoviruses via conidiospores. The consistency of sequence findings that we obtained from the different *E. muscae* isolates, for both mitovirus and mitochondrial coding sequences and despite these isolates having been originally obtained from three different species of host flies, also strongly supports the conclusion that these sequences originated from *E. muscae*.

By convention, each ORF diagrammed in Figure 1 and Figure 4 is considered to start with the first in-frame AUG codon. Because an alternative start codon might be used in some cases, however, it remains possible that some of these ORFs may be longer or shorter at their 5´ ends than suggested here. In this regard, we observed in particular that the *cox1* ORF of *E. muscae* contains a conserved in-frame GUG codon well-upstream of the first in-frame AUG (see Data S2), which would extend that ORF and its encoded protein considerably, to 1587 nt and 528 aa, respectively, bringing those lengths more in line with those of *C. heterosporus* [43] and most other organisms. For the mitoviruses, considering the strains with a longer region of sequence between the first in-frame AUG and the first upstream in-frame stop codon (see Figure 1), use of an alternative start codon upstream of the first in-frame AUG seems most likely for EnmuMV4, EnmuMV5, and EnmuMV8. Reciprocally, considering those viruses with shorter 5´ NTRs preceding the first in-frame AUG (see Figure 1), use of a start codon downstream of the first in-frame AUG seems most probable for EnmuMV6. Apparently notable in these regards, use of an alternative start codon upstream of the first in-frame AUG in EnmuMV4, EnmuMV5, and EnmuMV8 could provide for several additional residues near the RdRp N-terminus that would be conserved among all of the mitovirus strains reported here, and use of the second in-frame AUG codon to initiate translation in EnmuMV6 would retain these conserved residues (Figure S5). We therefore consider it likely that the first in-frame AUG is not the true start codon for RdRp translation in several of these viruses. In particular, for EnmuMV4, EnmuMV5, and EnmuMV8, we propose that an upstream AUU start codon is used instead, and for EnmuMV6, we propose that the second in-frame AUG codon is used instead (see Figure S3, Figure S5, and Data S2).

Coyle et al. [50] recently identified three SRA transcriptome libraries derived from mixtures of wild-caught flies, at least some of which were apparently harboring *E. muscae* when their RNAs were sampled (SRX955881, SRX955902, and SRX1711976; from BioProjects PRJNA277921 and PRJNA318834 [12]). Upon performing MegaBLAST searches of those libraries using the sequences of EnmuMV1–8 as queries, we found that two of the libraries, SRX955902 and SRX1711976, include sequence reads matching the *E. muscae* mitoviruses. Specifically, SRX955902 includes reads matching all eight of these viruses, and SRX1711976 includes reads matching four of these viruses (EnmuMV1, EnmuMV3, EnmuMV6, and EnmuMV7) (Table S5). Only the reads matching EnmuMV7 from SRX1711976 were sufficient in coverage to allow assembly of a complete coding sequence for this virus, which turns out to correspond (99.9% nt-sequence identity) to Hubei narna-like virus 25 (GenBank KX883546; 88.7–91.0% pairwise nt-sequence identity with the four EnmuMV7 strains from *E. muscae* detailed above). Thus, although Hubei narna-like virus 25 has been previously reported as an insect mitovirus [12], it is now shown to derive more likely from the fly-associated entomophthoroid fungus *E. muscae* or perhaps a related fungus. These findings provide further support for the conclusion that these and perhaps other yet-to-be-discovered mitoviruses are widespread in naturally occurring populations of *E. muscae*. 

The behavioral effects of *E. muscae* on host flies are fascinating and are indeed the subjects of ongoing genetic and neuroscientific investigations in *Drosophila* [24]. Interestingly, a distinct plus-strand RNA virus from *E. muscae*, an iflavirus (order *Picornavirales*), has been recently reported by Coyle et al. [50] and speculated to contribute to the manipulation of fly-host behaviors by *E. muscae*. The *E. muscae* mitoviruses that we describe here might also be considered as possible contributors in that regard. On a related note, two novel mitoviruses were recently identified in the entomopathogenic fungus *Ophiocordyceps sinensis* (phylum *Ascomycota*) [51], which causes behavioral perturbations and death of its caterpillar hosts. In this case, the dying caterpillar, which had been feeding as normal on plant roots underground, appears to be induced by the infecting fungus to crawl into a position and orientation relative to the soil surface that is ideal for growth of the fungal fruiting body aboveground, promoting efficient dispersal of the fungal spores [52]. These effects are clearly analogous to those of *E. muscae* in flies, and it is fascinating to speculate that again in this case the fungal mitoviruses might in some way contribute to the manipulation of insect host behaviors by the entomopathogenic fungus.

## Figures and Tables

**Table 1:** Top NR hits for deduced mitochondrial core-protein sequences derived from *E. muscae*.

Gene ^a^	Accession no.	Fungal Species	Sub–Phy ^b^	E-Value ^c^
atp6	AZZ06694.1	*Conidiobolus heterosporus*	Ent–Zoo	2e−115
	KXN65649.1	*Conidiobolus coronatus*	Ent–Zoo	1e−102
atp9	KXN65653.1	*Conidiobolus coronatus*	Ent–Zoo	6e−38
	AZZ06722.1	*Conidiobolus heterosporus*	Ent–Zoo	1e−37
cob	AZZ06693.1	*Conidiobolus heterosporus*	Ent–Zoo	0
cox1	AZZ06707.1	*Conidiobolus heterosporus*	Ent–Zoo	0
cox2	AZZ06713.1	*Conidiobolus heterosporus*	Ent–Zoo	7e−142
cox3	AZZ06710.1	*Conidiobolus heterosporus*	Ent–Zoo	6e−169
nad1	AZZ06725.1	*Conidiobolus heterosporus*	Ent–Zoo	4e−169
nad2	AZZ06721.1	*Conidiobolus heterosporus*	Ent–Zoo	6e−135
nad3	AZZ06726.1	*Conidiobolus heterosporus*	Ent–Zoo	6e−47
nad4	AZZ06717.1	*Conidiobolus heterosporus*	Ent–Zoo	0
nad5	AZZ06724.1	*Conidiobolus heterosporus*	Ent–Zoo	0
nad6	AZZ06724.1	*Conidiobolus heterosporus*	Ent–Zoo	1e−34
	KXN65652.1	*Conidiobolus coronatus*	Ent–Zoo	3e−23

^a^ Mitochondrial core genes; ^b^ Subphylum–Phylum: *Ent–Zoo*, *Entomophthoromycotina–Zoopagomycota*; ^c^ Top one or two hits in terms of E-value score are shown from searching the NR database with each mitochondrial protein sequence deduced from the apparent mitochondrial core-gene coding sequences from *E. muscae* isolate Berkeley.

## Supplementary Materials

The following are available online at https://www.mdpi.com/1999-4915/11/4/351/s1, Figure S1: Multiple sequence alignment of deduced RdRp sequences of mitoviruses from *E. muscae* isolate Berkeley, Figure S2: Sequence identity scores from pairwise alignments of *E. muscae* mitovirus sequences, Figure S3: Multiple sequence alignments of 5´ and 3´ NTR sequences of *E. muscae* mitoviruses, Figure S4: Fungal tree of life based on mitochondrial core-protein sequences from representative species, Figure S5: Multiple sequence alignment of N-terminal regions of deduced RdRp sequences of *E. muscae* mitoviruses, Table S1: Contig assembly values for coding-complete mitovirus sequences from *E. muscae*, Table S2: Contig assembly values for mitochondrial core-gene coding sequences from *E. muscae*, Table S3: SRA accessions used for contig assemblies in this study, Table S4: Mitovirus-matching reads in samples from fruit flies infected or not with *E. muscae* isolate Berkeley per Elya et al. [24], Table S5: Mitovirus-matching reads in samples from SRA transcriptome libraries derived from mixtures of wild-caught flies apparently infected with *E. muscae* per Coyle et al. [50], Table S6: Mitovirus RdRp sequences used for phylogenetic analyses, Data S1: *E. muscae* mitovirus sequences, Data S2: *E. muscae* mitochondrial coding sequences.
