# Supplementary material for: Mitovirus and Mitochondrial Coding Sequences from Basal Fungus Entomophthora muscae
Source: Viruses. 2019 Apr 17;11(4):351. doi: 10.3390/v11040351 (PMC6520771; doi:10.3390/v11040351)
Supplement: Supplementary file 1 [file viruses-11-00351-s001.zip › Supp1-EnmuMV-TablesS1-S6.docx]

**Supplementary Tables**

**Table S1.** Contig assembly values for coding-complete mitovirus sequences from *E. muscae*

-------------------------------------------------------------------------------------

Mitovirus^a^ Isolate/strain Mapped RPKM^b^ Depth^c^

reads

-------------------------------------------------------------------------------------

EnmuMV1 KVL-14-117 2808 8.2 97

KVL-14-118 633 1.9 21

HHdFL130914-1 2348 7.8 122

Berkeley 2558 1.5 89

EnmuMV2 KVL-14-117 456 1.5 19

KVL-14-118^d^ 186 0.6 7

HHdFL130914-1 564 2.2 34

Berkeley 581 0.4 23

EnmuMV3 KVL-14-117 518 1.7 20

KVL-14-118 166 0.6 6

HHdFL130914-1 478 1.7 28

Berkeley 1421 0.9 54

HHdFL050913-1 228 2.2 9

EnmuMV4 KVL-14-117 2361 7.5 92

KVL-14-118 734 2.4 28

HHdFL130914-1 1459 5.3 84

Berkeley 4174 2.8 163

EnmuMV5 KVL-14-117 1114 3.7 46

KVL-14-118 346 1.2 14

HHdFL130914-1 428 1.6 23

Berkeley 1633 1.1 65

EnmuMV6 KVL-14-117 1836 6.4 78

KVL-14-118 318 1.1 12

HHdFL130914-1 640 2.5 39

Berkeley 2514 1.8 106

EnmuMV7 KVL-14-117 163 0.6 7

KVL-14-118 264 0.9 10

HHdFL130914-1 231 0.9 14

Berkeley 1164 0.8 48

EnmuMV8 Berkeley 1424 1.0 57

-------------------------------------------------------------------------------------

^a^Abbreviations defined in text.

^b^Mapped reads (R) per (P) thousand nt of reference sequence (K) per million total reads (M).

^c^Median coverage depth at each nt position.

^d^Sequence includes two small sequencing gaps relative to related sequences (diagrammed in Figure 1).

**Table S2.** Contig assembly values for mitochondrial core-gene coding sequences from *E. muscae*

-------------------------------------------------------------------------------------

Mitochondrial Isolate Mapped RPKM^a^ Depth^b^

core gene reads

-------------------------------------------------------------------------------------

*atp6* KVL-14-117 134 0.7 16

KVL-14-118 119 1.3 15

HHdFL130914-1 268 3.2 49

Berkeley 3746 8.3 468

HHdFL050913-1 172 5.4 19

*atp9* KVL-14-117 123 2.2 42

KVL-14-118 76 2.8 25

HHdFL130914-1 66 2.7 35

Berkeley 608 4.6 210

HHdFL050913-1 73 7.8 26

*cob* KVL-14-117 313 1.1 25

KVL-14-118 146 1.1 11

HHdFL130914-1 299 2.4 33

Berkeley 7597 11.4 638

HHdFL050913-1 363 7.7 33

*cox1* KVL-14-117 425 1.1 16

KVL-14-118 266 1.4 12

HHdFL130914-1 578 3.4 37

Berkeley 9369 10.0 551

HHdFL050913-1 196 3.0 12

*cox2* KVL-14-117 147 0.8 16

KVL-14-118 75 0.8 8

HHdFL130914-1 147 1.8 24

Berkeley 2546 5.8 334

HHdFL050913-1 128 4.1 16

*cox3* KVL-14-117 113 0.6 14

KVL-14-118^c^ 79 0.8 9

HHdFL130914-1 167 1.9 25

Berkeley 2788 5.9 302

HHdFL050913-1 67 2.0 7

*nad1* KVL-14-117^c^ 24 0.1 2

KVL-14-118^c^ 24 0.2 2

HHdFL130914-1^c^ 12 0.1 2

Berkeley 485 0.9 52

HHdFL050913-1^c^ 37 1.0 4

*nad2* KVL-14-117 247 0.7 17

KVL-14-118^c^ 119 0.7 8

HHdFL130914-1 50 0.3 4

Berkeley 2537 3.1 177

HHdFL050913-1^c^ 91 1.6 6

*nad3* KVL-14-117 71 0.9 18

KVL-14-118 62 1.4 13

HHdFL130914-1 99 2.6 31

Berkeley 974 4.7 219

HHdFL050913-1 70 4.8 16

*nad4* KVL-14-117 340 1.0 22

KVL-14-118 138 0.8 7

HHdFL130914-1 120 0.8 12

Berkeley 4055 4.8 270

HHdFL050913-1 107 1.8 6

*nad5* KVL-14-117 377 0.8 18

KVL-14-118 162 0.7 7

HHdFL130914-1^c^ 75 0.4 5

Berkeley 6473 5.6 339

HHdFL050913-1^c^ 141 1.7 6

*nad6* KVL-14-117^c^ 36 0.3 7

KVL-14-118^c^ 19 0.3 4

HHdFL130914-1 16 0.3 3

Berkeley 116 0.5 21

HHdFL050913-1^c^ 10 0.5 2

-------------------------------------------------------------------------------------

^a^Mapped reads (R) per (P) thousand nt of reference sequence (K) per million total reads (M).

^b^Median coverage depth at each nt position.

^c^Sequences include gaps or truncations relative to related sequences (diagrammed in Figure 4).

**Table S3.** SRA accessions used for contig assemblies in this study

---------------------------------------------------------------------------------------------------------

*E. muscae* SRA accessions used for assembling respective contigs:

isolate Mitoviruses Mitochondrial transcripts

---------------------------------------------------------------------------------------------------------

KVL-14-117 ERX1101545–ERX1101547^a^ ERX1101528–ERX1101530^b^,

ERX1101545–ERX1101547^a^

KVL-14-118^b^ ERX1101548–ERX1101550 ERX1101548–ERX1101550

HHdFL130914-1^b^ SRX2782457 SRX2782457

Berkeley^b^ SRX3738070–SRX3738072^c^, SRX3738070–SRX3738072^c^,

SRX3738074–SRX3738076^d^, SRX3738074–SRX3738076^d^,

SRX3738080^e^, SRX3738080^e^,

SRX3738089–SRX3738091^d^, SRX3738089–SRX3738091^d^,

SRX3738099^c^, SRX3738102^c^, SRX3738099^c^, SRX3738102^c^,

SRX3738103^c^, SRX3738106^e^, SRX3738103^c^, SRX3738106^e^,

SRX3738107^e^, SRX3738107^e^,

SRX3738123– SRX3738125^f^ SRX3738123– SRX3738125^f^

HHdFL050913-1^b^ ERX1101553 ERX1101553

HHdFL040913-2^b^ ERX1101552 N/A

---------------------------------------------------------------------------------------------------------

^a^RNA samples from media-grown *E. muscae.*

^b^RNA samples from fly-grown *E. muscae.*

^c^72 h post-infection with *E. muscae*, whole fly bodies.

^d^96 h post-infection with *E. muscae*, whole fly bodies.

^e^120 h post-infection with *E. muscae*, whole fly bodies.

^f^72 h post-infection with *E. muscae*, dissected fly brains.

N/A, not applied.

**Table S4.** Mitovirus-matching reads in samples from fruit flies infected or not with *E. muscae* isolate Berkeley per Elya et al. [24]

--------------------------------------------------------------------------------------------------------------------------------------------------------------

Sample Mean no. of virus-matching sequence reads per fly sample^b^:

set^a^ EnmuMV1 EnmuMV2 EnmuMV3 EnmuMV4 EnmuMV5 EnmuMV6 EnmuMV7 EnmuMV8

--------------------------------------------------------------------------------------------------------------------------------------------------------------

I-24-W^c^ 1.0 0.0 0.0 0.7 0.0 0.7 0.3 1.3

I-48-W^c^ 0.8 0.3 1.7 4.3 1.3 1.3 2.3 1.0

I-72-W^c^ 83.8 18.7 35.2 164.7 61.8 42.3 53.2 41.7

I-96-W^c^ 197.7 47.8 101.3 332.8 111.7 158.2 92.8 108.3

I-120-W^d^ 258.7 54.7 183.3 352.3 168.3 410.3 72.7 158.7

U-24-W^d^ 0.7 0.0 0.0 1.0 0.0 0.0 0.0 0.3

U-48-W^d^ 0.0 0.0 0.0 0.7 0.0 0.0 0.0 0.3

U-72-W^d^ 0.7 0.0 0.0 0.7 0.0 0.0 0.0 0.0

U-96-W^d^ 0.7 0.0 0.0 0.0 0.0 0.0 0.0 0.0

U-120-W^d^ 0.7 0.0 0.0 0.7 0.0 0.0 0.0 0.0

--------------------------------------------------------------------------------------------------------------------------------------------------------------

I-24-B^d^ 0.0 0.0 0.0 0.0 0.7 2.0 0.0 0.0

I-48-B^e^ 0.0 0.0 0.0 0.7 2.0 0.7 1.3 0.7

I-72-B^d^ 31.3 6.0 17.3 44.0 29.0 26.7 23.3 19.3

U-24-B^d^ 0.0 0.0 0.0 0.0 0.0 0.0 0.0 0.0

U-48-B^d^ 0.0 0.0 0.0 0.0 0.0 0.0 0.0 0.0

U-72-B^d^ 0.0 0.0 0.0 0.0 0.0 0.3 0.0 0.0

--------------------------------------------------------------------------------------------------------------------------------------------------------------

^a^I, infected; U, uninfected. 24–48–72–96–120, h after infection or mock infection. W, whole fly bodies; B, dissected fly brains.

^b^Matching reads identified by MegaBLAST with an E-value threshold of 1e–4

^c^Six samples per sample set.

^d^Three samples per sample set.

^e^Two samples per sample set.

**Table S5.** Mitovirus-matching reads in samples from SRA transcriptome libraries derived from mixtures of wild-caught flies infected with *E. muscae* per Coyle et al. [50]

------------------------------------------------------------------------------------------------------------------------------------------------------------------

SRA No. of matching sequence reads^a^:

library EnmuMV1 EnmuMV2 EnmuMV3 EnmuMV4 EnmuMV5 EnmuMV6 EnmuMV7 EnmuMV8

------------------------------------------------------------------------------------------------------------------------------------------------------------------

SRX955881 0 0 0 0 0 0 0 0

SRX955902 33 34 20 118 25 31 11 14

SRX1711976 130 0 65 0 0 102 590 0

------------------------------------------------------------------------------------------------------------------------------------------------------------------

^a^Matching reads identified by MegaBLAST with an E-value threshold of 1e–4 using the virus sequences from *E. muscae* isolate Berkeley as queries.

**Table S6.** Mitovirus RdRp sequences used for phylogenetic analyses

--------------------------------------------------------------------------------------------------------------------

GenBank Virus abbrev. Virus name

accession no.

--------------------------------------------------------------------------------------------------------------------

AAA61703 CrpaMV1 Cryphonectria parasitica mitovirus 1

AAN05635 GrMV-S1 Gremmeniella mitovirus S1

AAR01970 CrcuMV1a Cryphonectria cubensis mitovirus 1a

AAR01973 CrcuMV2a Cryphonectria cubensis mitovirus 2a

AAT09164 ThbaMV Thielaviopsis basicola mitovirus

AEG79311 TuaeMV Tuber aestivum mitovirus

AEP83726 TuexMV Tuber excavatum mitovirus

AEX91878 ScscMV1 Sclerotinia sclerotiorum mitovirus 1

AEX91879 ScscMV2 Sclerotinia sclerotiorum mitovirus 2

AEY76153 GrabNHSMRV-S1 Gremmeniella abietina non-host-specific mitochondrial

RNA virus S1

AGC24232 ScscMV3 Sclerotinia sclerotiorum mitovirus 3

AGC24233 ScscMV4 Sclerotinia sclerotiorum mitovirus 4

AGT55877 OpMV7 Ophiostoma mitovirus 7

AGW51760 AEF2013MV Mitovirus AEF-2013

AHF48620 ScscMV6 Sclerotinia sclerotiorum mitovirus 6

AHF48625 ScscMV9 Sclerotinia sclerotiorum mitovirus 9

AHF48627 ScscMV11 Sclerotinia sclerotiorum mitovirus 11

AHF48628 ScscMV12 Sclerotinia sclerotiorum mitovirus 12

AHI43533 FuciMV1 Fusarium circinatum mitovirus 1

AHI43534 FuciMV2-1 Fusarium circinatum mitovirus 2-1

AHL25281 RhMV1-RS002 Rhizoctonia mitovirus 1 RS002

AHY03257 BespMV1 Buergenerula spartinae mitovirus 1

AIF33766 HeMV1 Heterobasidion mitovirus 1

AIT71973 RhceMV Rhizoctonia cerealis mitovirus

AKN79252 AlbrMV Alternaria brassicicola mitovirus

ALD60243 BRhMV-K1 Binucleate Rhizoctonia mitovirus K1

ALD89116 RhsoMV11 Rhizoctonia solani mitovirus 11

ALD89117 RhsoMV12 Rhizoctonia solani mitovirus 12

ALD89118 RhsoMV13 Rhizoctonia solani mitovirus 13

ALD89120 RhsoMV15 Rhizoctonia solani mitovirus 15

ALD89121 RhsoMV2 Rhizoctonia solani mitovirus 2

ALD89125 RhsoMV6 Rhizoctonia solani mitovirus 6

ALD89127 RhsoMV8 Rhizoctonia solani mitovirus 8

ALD89136 ScscMV19 Sclerotinia sclerotiorum mitovirus 19

ALM62240 SLAMV5 Soybean leaf-associated mitovirus 5

ALM62241 SLAMV1 Soybean leaf-associated mitovirus 1

ALM62243 SLAMV3 Soybean leaf-associated mitovirus 3

AMM45292 MaphMV3 Macrophomina phaseolina mitovirus 3

AMQ67414 CrriMV1 Cronartium ribicola mitovirus 1

AMQ67415 CrriMV2 Cronartium ribicola mitovirus 2

AMQ67416 CrriMV3 Cronartium ribicola mitovirus 3

AMQ67417 CrriMV4 Cronartium ribicola mitovirus 4

AMQ67418 CrriMV5 Cronartium ribicola mitovirus 5

ANA08076 RhosMV1 Rhizoctonia oryzae-sativae mitovirus 1

AOX47577 CeMV-A Ceratobasidium mitovirus A

AQM32767 AgbiMV1 Agaricus bisporus mitovirus 1

ARO49434 NeluMV1 Neofusicoccum luteum mitovirus 1

ATS94398 ErneMV1 Erysiphe necator mitovirus 1

ATS94399 ErneMV2 Erysiphe necator mitovirus 2

ATS94400 ErneMV3 Erysiphe necator mitovirus 3

AVA17449 GimaMV1 Gigaspora margarita mitovirus 1

AVA17450 GimaMV2 Gigaspora margarita mitovirus 2

AVA17451 GimaMV3 Gigaspora margarita mitovirus 3

AVA17452 GimaMV4 Gigaspora margarita mitovirus 4

AWY10986 ScscMV28 Sclerotinia sclerotiorum mitovirus 28

AWY10987 ScscMV29 Sclerotinia sclerotiorum mitovirus 29

AXY40441 RhdiMV2 Rhizophagus diaphanum mitovirus 2

AXY40444 RhirMV1 Rhizophagus irregularis mitovirus 1

AZJ25096 RhdiMV3 Rhizophagus diaphanum mitovirus 3

AZP53929 NiorMV2 Nigrospora oryzae mitovirus 2

BAD72871 HemoMV1-18 Helicobasidium mompa mitovirus 1-18

BAJ23143 RhRF1MV Rhizophagus sp. RF1 mitovirus

BAN85985 RhHR1MV Rhizophagus sp. HR1 mitovirus-like ssRNA

BAV56289 FupoMV1 Fusarium poae mitovirus 1

BAV56292 FupoMV4 Fusarium poae mitovirus 4

BBG56024 FuboMV1 Fusarium boothii mitovirus 1

CAA06228 OpMV3a Ophiostoma mitovirus 3a

CAB42652 OpMV4 Ophiostoma mitovirus 4

CAB42653 OpMV5 Ophiostoma mitovirus 5

CAB42654 OpMV6 Ophiostoma mitovirus 6

CAJ32466 OpMV1a Ophiostoma mitovirus 1a

CAJ32467 OpMV1b Ophiostoma mitovirus 1b

CAJ32468 OpMV3b Ophiostoma mitovirus 3b

CCG47524 ClodMV Clitocybe odora virus

CEZ26301 BociMV2 Botrytis cinerea mitovirus 2

CEZ26302 BociMV3 Botrytis cinerea mitovirus 3

DAB41740 AmarMV1 Ambrosia artemisiifolia mitovirus 1

DAB41741 AzfiMV1 Azolla filiculoides mitovirus 1

DAB41743 SochMV1 Solanum chacoense mitovirus 1

DAB41745 OxruMV1 Oxybasis rubra mitovirus 1

DAB41746 CasaMV1 Cannabis sativa mitovirus 1

DAB41747 DapiMV1 Dahlia pinnata mitovirus 1

DAB41757 BevuMV1 Beta vulgaris mitovirus 1

--------------------------------------------------------------------------------------------------------------------
