# Supplementary material for: Mitovirus and Mitochondrial Coding Sequences from Basal Fungus Entomophthora muscae
Source: Viruses. 2019 Apr 17;11(4):351. doi: 10.3390/v11040351 (PMC6520771; doi:10.3390/v11040351)
Supplement: Supplementary file 1 [file viruses-11-00351-s001.zip › Supp10-EnmuMV-DataS1.pdf]

Supplementary Data

***E. muscae* Mitovirus Sequences**

Red shading: upstream stop codon, first in-frame AUG codon, and downstream stop codon Orange shading: proposed alternative start codon
Gray shading: UGA(Trp) codons
Yellow shading: GDD-motif codons

N residues (cyan lettering) were inserted into the EnmuMV2-KVL-14-118 sequence to fill two gaps relative to the conserved sequences of other EnmuMV2 strains.

>EnmuMV1-KVL-14-117
GACGTCTCTTTACAGAGACGTCTAACAGGACAAGCGTCCTTTGGTCATGAACCAACCTCG
CGAATAGATCCCTCATCAGGACCGAAGGTTATACCTTCTATGGTCGGATGTTAGGTTTCG
ACTTCCAAGCGGGTGAAGACGTCTACACCTAAAATCCTCCTGGCTCTGCGTGATGCAGGC
AACCAGGTCGGGTAAAGTGACCCGGCGTCTAGAGCTAAAGTAGATGAGTAACTTAAGAGG
AGTTATATCCTCTTATAGTTCTTATCCAAATAGATAGAATGGGGGCCCCCGATGTCATTG
TATCCGGTAAA**TAA**TTACCAATTATTTATCGAGATTCCAACAACACAGAAACTAAA**ATG**A
AAAGCAAACAACAATTATTAATCCTTGTCGGATGAGTAATTCGTTGGTACTTTTCTTCGG
TTCCTGAGTCGCGAAATGCTTATAGATACTGAAAACATCTATGAGCTCGGTACGAAGAGT
CCCGAGGGTTAGAATGAACGGTTAAACGTTTCAAACTAATCCGATTATGCGTAACCAGAT
ACATATCTGGAAAACCGCATTATCCTACTGATGTAACAATCGGCATGACGAAAGATGGAC
TTCCAAAGGCTTTAGGGCCTTTAAAGTCTCTCGTTCGTTCCAGGGACGTGCAAAGTCTAA
GATTAGTCCTTACCCTACTTTACGTAGGCCGGGCCTTTAGACCTAGTACGTTAAATCCTG
ATTTAAGGAGTATCACTACTCCTTCATCAGCGAATCCTGAACTGATTGAAGATATATATC
AATTCGCAAAGAACTCGCGAGTTGATCTACATCTTTCATCTATAGATACGGACTTTAGGG
CTTTTCACTTTACAGAGAAGGCCGGCCCGTCTGGACATGGTCTTTTGGCTAGTTATAATG
ATTATTATAATCTGACAGAAGACTTGAAACAAGATTTGCTTCTTATTTCAAACCAGACGA
TTGGAGAGCATTTCAACAATGTTGAGAATACTCATCCAACGTTGCTTGATCACTTTCGGA
CCAATTGGGTTAAGAAGACCGAAGACGGTCAACTTTCCCCCTTTAGATCAAAACTCATAC
GAGTTATTAGTCTAAAGCCCGATCGTGAGTACAAGACAAGGCCGTTTGCTTTATTAGACT
ATTGAAGTCAGACTTGTTTAAAACCAATACATGACAAGATATTCGGTATCTTGAAACAGT
ATGGTAAAACAGACTGTACATTCGGTCATAATGAAGCAATGAACATAGCGTCTAATATTC
CTAATCCAGATGGATCTCAATTCCATAGTCTTGATCTCTCAAGCGCTACAGATAGATTTC
CAATGGACCTTCAAGTTAAGATTTTATCACTCATGATTGGTGAGGATAAAGCCGAGGCTT
GAAGGAGAATATTGGTTAGCAAAGAGTATACAGTGCTCTCGACTGGGGCGAGGGTTCGTT
ACCTCGCCGGACAGCCGATGGGCGCGTATAGCTCTTGAGCTATGTTCACCTTATGCCACC
ACATAGTGGTTAGGTATGCGGCGCGTAATGCGAAGTTTGACCGATACACAATAATTGGCG
ATGATATAGTTATTAGGAACGACAACGTTGCCGAACGCTACAAGAATATCGTAAACCAGT
TAGGTGTAACCATTTCAGAGGCAAAATCTCATGTGTCACCCGACACATTTGAAATAGCTA

AGAAATGGTACTACCGTGGAAAGGAGATTACCCCTTTTCCGATCGATGGTATGGAGAAGA
TATGAAAGTCTTACGACCTTTTATTCTTGTTCCTACTAGACCTTAAAAGAAATAAAGGTC
TAGAGCCTCGATTTGGAGTGGAATCTCTAGCGATGATTAGATCTCTCTGATCAAGACTAG
GGAAACCCCCACGACTAGTACGGTCTGTCTGTAATGGGTACAGACGCTTTGCAGCCTTTC
CGTTCTTTGGTGAGAAGGACGCTGACTATAAGGATCCACGGGGATTCTTAGAATCAGTTA
ACTTCCCAAGTGCAGGACTAATGAGTCAAGAGTCAGCAAAGGCTCTTATTACATCAGTTA
CTGCCGGCCAATTAACGAACTCCCTTGTTAATCAGTTATTTACGTACCAAATGGACATAA
ATACTATAACAAAGAGAGCTTACGAGTCTGTTTCCATCTTGGGATACGGACCGTTTGCTG
AAGGGCTGGAGGACAAGACCATTGATAATCATCCTTTCTTTGCATCGCAGAAGGCGATGG
TTAAAGATAGTCTGAGCATCCTTACGGAGGCCAAGGAGTCTGCTTCTAAGAATAACTTTG
ATGACATAATAAATATGAAACCTCTGATATTCTTAAGCAACCCAACGGCTAGGTTGAGCG
GAGACCGAGAGGTTGAAATTCTCGGTTCTCAAGCAGTTCTAGTCCACTCCGCGGTAGCCG
CCCTTAAAGGGATCATGCTAATCCGTGAACTCGTTTTAACAAACGAGGAAGATTCACAGA
CTGAGCAATGAGTTCTTCGAAGATATACTGACGATTTTAACATCGTCCGAAAACTTGAGA
ACGTGCTGAAGAACTTCATCTTTGATGATGTTCTTCCACGTGCTAAGTTTAAAGCCTTCA
TGAAGGACTTTAAATCTCGAAGTCGAGTCAAATCACTTTTAGATGAT**TAG**CCTCTCCCTC
TGAAGGGGGTCTTTCCTCGTCAAAATCTTAAGATGATGATAGAGAGGGGCTCTTAACACC
AAATTAAAAGGCTCATAACCTTTGGCTAGCAATAGTTTATTCGCCGGGACAGTTCATGAC
TGTCGGGGATAATATCCCCTGTTAGACGACCTCGTAAGAGGTCG
>EnmuMV1-KVL-14-118
AGACGTCTAACAGGACAAGCGTCCTTTGGTCATGAACCAACCTCGCGAATAGATCCCTCA
TCAGGACCGAAGGTTATACCTTCTATGGTCGGATGTTAGGTTTCGACTTCCAAGCGGGTG
AAGACGTCTACACCTAAAATCCTCCTGGCTCTGCGTGATGCAGGCAACCAGGTCGGGTAA
AGTGACCCGGCGTCTAGAGCTAAAGTAGATGAGTAACTTAAGAGGAGTTATATCCTCTTA
TAGTTCTTATCCAAATAGATAGAATGGGGGCCCCCGATGTCATTGTATCCGGTAAA**TAA**T
TACCAATTATTTATCGAGATTCCAACAACACAGAAACTAAA**ATG**AAAAGCAAACAACAAT
TATTAATCCTTGTCGGATGAGTAATTCGTTGGTACTTTTCTTCGGTTCCTGAGTCGCGAA
ATGCTTATAGATACTGAAAACATCTATGAGCTCGGTACGAAGAGTCCCGAGGGTTAGAAT
GAACGGTTAAACGTTTCAAACTAATCCGATTATGCGTAACCAGATACATATCTGGAAAAC
CGCATTATCCTACTGATGTAACAATCGGCATGACGAAAGATGGACTTCCAAAGGCTTTAG
GGCCTTTAAAGTCTCTCGTTCGTTCCAGGGACGTACAAAGTCTAAGATTAGTCCTTACCC
TACTTTACGTAGGCCGGGCCTTTAGACCTAGTACGTTAAATCCTGATTTAAGGAGTATCA
CTACTCCTTCATCAGCGAATCCTGAACTGATTGAAGATATATATCAATTCGCAAAGAACT
CGCGAGTTGATCTACATCTTTCATCTATAGATACGGACTTTAGGGCTTTTCACTTTACAG
AGAAGGCCGGCCCGTCTGGACATGGTCTTTTGGCTAGTTATAATGATTATTATAACCTGA
CAGAAGACTTGAAACAAGATTTGCTTCTTATTTCAAACCAGACGATTGGAGAGCATTTCA
ACAATGTTGAGAATACTCATCCAACGTTGCTTGATCACTTTCGGACCAATTGGGTTAAGA
AGACCGAAGACGGTCAACTTTCCCCCTTTAGATCAAAACTCATACGAGTTATTAGTCTAA
AGCCCGATCGTGAGTACAAGACAAGGCCGTTTGCTTTATTAGACTATTGAAGTCAGACTT
GTTTAAAACCAATACATGACAAGATATTCGGTATCTTGAAACAGTATGGTAAAACAGACT
GTACATTCGGTCATAATGAAGCAATGAACATAGCGTCTAATATTCCTAATCCAGATGGAT
CTCAATTCCATAGTCTTGATCTCTCAAGCGCTACAGATAGATTTCCAATGGACCTTCAAG
TTAAGATTTTATCACTCATGATTGGTGAGGATAAAGCCGAGGCTTGAAGGAGAATATTGG
TTAGCAAAGAGTATACAGTGCTCTCGACTGGGGCGAGGGTTCGTTACCTCGCCGGACAGC

CGATGGGCGCGTATAGCTCTTGAGCTATGTTCACCTTATGCCACCACATAGTGGTTAGGT
ATGCGGCGCGTAATGCGAAGTTTGACCGATACACAATAATTGGCGATGATATAGTTATTA
GGAACGACAACGTTGCCGAACGCTACAAGAATATCGTAAACCAGTTAGGTGTAACCATTT
CAGAGGCAAAGTCTCATGTGTCACCCGACACATTTGAAATAGCTAAGAAATGGTACTACC
GTGGAAAGGAGATTACCCCTTTTCCGATCGATGGTATGGAGAAGATATGAAAGTCTTACG
ACCTTTTATTCTTGTTCCTACTAGACCTTAAAAGAAATAAAGGTCTAGAGCCTCGATTTG
GAGTGGAATCTCTAGCGATGATTAGATCTCTCTGATCAAGACTAGGGAAACCCCCACGAC
TAGTACGGTCTGTCTGTAATGGGTACAGACGCTTTGCAGCCTTTCCGTTCTTTGGTGAGA
AGGACGCTGACTATAAGGATCCACGGGGATTCTTAGAATCAGTTAACTTCCCAAGTGCAG
GACTAATGAGTCAAGAGTCAGCAAAGGCTCTTATTACATCAGTTACTGCCGGCCAATTAA
CGAACTCCCTTGTTAATCAGTTATTTACGTACCAAATGGACATAAATACTATAACAAAGA
GAGCTTACGAGTCTGTTTCCATCTTGGGATACGGACCGTTTGCTGAAGGGCTGGAGGACA
AGACCATTGATAATCATCCTTTCTTTGCATCGCAGAAGGCGATGGTTAAAGATAGTCTAA
GCATCCTTACGGAGGCCAAGGAGTCTGCTTCTAAGAATAACTTTGATGACATAATAAATA
TGAAACCTCTGATATTCTTAAGCAACCCAACGGCTAGGTTGAGCGGAGACCGAGAGGTTG
AAATTCTCGGTTCTCAAGCAGTTCTAGTCCACTCCGCGGTAGCCGCCCTTAAAGGGATCA
TGCTAATCCGTGAACTCGTTTTAACAAACGAGGAAGATTCACAGACTGAGCAATGAGTTC
TTCGAAGATATACTGACGATTTTAACATCGTCCGAAAACTTGAGAACGTGCTGAAGAATT
TCATCTTTGATGATGTTCTTCCACGTGCCAAGTTTAAAGCCTTCATGAAGGACTTTAAAT
CTCGAAGTCGAGTCAAATCACTTTTAGATGAT**TAG**TCTCTCCCTCTGAAGGGGGTCTTTC
CTCGTCAAAATCTTAAGATGATGATAGAGAGGGGCTCTCAACACCAAATTAAAAGGCTCA
TAACCTTTGGCTAGCAATAGTTTATTCGCCGGGACAGTTCATGACTGTCGGGGATAATAT
CCCCTGTTAGACGACCTCGTAAGAGG
>EnmuMV1-HHdFL130914-1
CGTCTCTTTACAGAGACGTCTAACAGGACAAGCGTCCTTTGGTCATGAACCAACCTCGCG
AATAGATCCCTCATCAGGACCGAAGGTTATACCTTCTATGGTCGGATGTTAGGTTTCGAC
TTCCAAGCGGGTGAAGACGTCTACACCTAAAATCCTCCTGGCTCTGCGTGATGCAGGCAA
CCAGGTCGGGTAAAGTGACCCGGCGTCTAGAGCTAAAGTAGATGAGTAACTTAAGAGGAG
TTATATCCTCTTATAGTTCTTATCCAAATAGATAGAATGGGGGCCCCCGATGTCATTGTA
TCCGGTAAA**TAA**TTACCAATTATTTATCGAGATTCCAACAACACAGAAACTAAA**ATG**AAA
AGCAAACAACAATTATTAATCCTTGTCGGATGAGTAATTCGTTGGTACTTTTCTTCGGTT
CCTGAGTCGCGAAATGCTTATAGATACTGAAAACATCTATGAGCTCGGTACGAAGAGTCC
CGAGGGTTAGAATGAACGGTTAAACGTTTCAAACTAATCCGATTATGCGTAACCAGATAC
ATATCTGGAAAACCGCATTATCCTACTGATGTAACAATCGGCATGACGAAAGATGGACTT
CCAAAGGCTTTAGGGCCTTTAAAGTCTCTCGTTCGTTCCAGGGACGTACAAAGTCTAAGA
TTAGTCCTTACCCTACTTTACGTAGGCCGGGCCTTTAGACCTAGTACGTTAAATCCTGAT
TTAAGAAGTATCACTACTCCTTCATCAGCGAATCCTGAACTGATTGAAGATATATATCAA
TTCGCAAAGAACTCGCGAGTTGATCTACATCTTTCATCTATAGATACGGACTTTAGGGCT
TTTCACTTTACAGAGAAGGCCGGCCCGTCTGGACATGGTCTTTTGGCTAGTTATAATGAT
TATTATAATCTGACAGAAGACTTGAAACAAGATTTGCTTCTTATTTCAAACCAGACGATT
GGAGAGCATTTCAACAATGTTGAGAATACTCATCCAACGTTGCTTGATCACTTTCGGACC
AATTGGGTTAAGAAGACCGAAGACGGTCAACTTTCCCCCTTTAGATCAAAACTCATACGA
GTTATTAGTCTAAAGCCCGATCGTGAGTACAAGACAAGGCCGTTTGCTTTATTAGACTAT
TGAAGTCAGACTTGTTTAAAACCAATACATGACAAGATATTCGGTATCTTGAAACAGTAT

GGTAAAACAGACTGTACATTCGGTCATAATGAAGCAATGAACATAGCGTCTAATATTCCT
AATCCAGATGGATCTCAATTCCATAGTCTTGATCTCTCAAGCGCTACAGATAGATTTCCA
ATGGACCTTCAAGTTAAGATTTTATCACTCATGATTGGTGAGGATAAAGCCGAGGCTTGA
AGGAGAATATTGGTTAGCAAAGAGTATACAGTGCTCTCGACTGGGGCGAGGGTTCGTTAC
CTCGCCGGACAGCCGATGGGCGCGTATAGCTCTTGAGCTATGTTCACCTTATGCCACCAC
ATAGTGGTTAGGTATGCGGCGCGTAATGCGAAGTTTGACCGATACACAATAATTGGCGAT
GATATAGTTATTAGGAACGACAACGTTGCCGAACGCTACAAGAATATCGTAAACCAGTTA
GGTGTAACCATTTCAGAGGCGAAATCTCATGTGTCACCCGACACATTTGAAATAGCTAAG
AAATGGTACTACCGTGGAAAGGAGATTACCCCTTTTCCGATCGATGGTATGGAGAAGATA
TGAAAGTCTTACGACCTTTTATTCTTGTTCCTACTAGACCTTAAAAGAAATAAAGGTCTA
GAGCCTCGATTTGGAGTGGAATCTCTAGCGATGATTAGATCTCTCTGATCAAGACTAGGG
AAACCCCCACGACTAGTACGGTCTGTCTGTAATGGGTACAGACGCTTTGCAGCCTTTCCG
TTCTTTGGTGAGAAGGACGCTGACTATAAGGATCCACGGGGATTCTTAGAATCAGTTAAC
TTCCCAAGTGCAGGACTAATGAGTCAAGAGTCAGCAAAGGCTCTTATTACATCAGTTACT
GCCGGCCAATTAACGAACTCCCTTGTTAATCAGTTATTTACGTACCAAATGGACATAAAT
ACTATAACAAAGAGAGCTTACGAGTCTGTTTCCATCTTGGGATACGGACCGTTTGCTGAA
GGGCTGGAGGACAAGACCATTGATAATCATCCTTTCTTTGCATCGCAGAAGGCGATGGTT
AAAGATAGTCTGAGCATCCTTACGGAGGCCAAGGAGTCTGCTTCTAAGAATAACTTTGAT
GACATAATAAATATGAAACCTTTGATATTCTTAAGCAACCCAACGGCTAGGTTGAGCGGA
GACCGAGAGGTTGAAATTCTCGGTTCTCAAGCAGTTCTAGTCCACTCCGCGGTAGCCGCC
CTTAAAGGGATCATGCTAATCCGTGAACTCGTTTTAACAAACGAGGAAGATTCACAGACT
GAGCAATGAGTTCTTCGAAGATATACTGACGACTTTAACATCGTCCGAAAACTTGAGAAC
GTGTTGAAGAATTTCATCTTTGATGATGTTCTTCCACGTGCTAAGTTTAAAGCCTTCATG
AAGGACTTTAAATCTCGAAGTCGAGTCAAATCACTTTTAGATGAT**TAG**TCTCTCCCTCTG
AAGGGGGTCTTTCCTCGTCAAAATCTTAAGATGATGATAGAGAGGGGCTCTTAACACCAA
ATTAAAAGGCTCATAACCTTTGGCTAGCAATAGTTTATTCGCCGGGACAGTTCATGACTG
TCGGGGATAATATCCCCTGTTAGACGACCTCGTAAGAGGTCG
>EnmuMV1-Berkeley
GACGTCTCTTTACAGAGACGTCTAACAGGACAAGCGTCCTTTGGTCATGAACCAACCTCG
CGAATAGATCCCTCATCAGGACCGAAGGTTATACCTTCTATGGTCGGATGTTAGGTTTCG
ACTTCCAAGCGGGTGAAGACGTCTACACCTAAAATCCTCCTGGCTCTGCGTGATGCAGGC
AACCAGGTCGGGTAAAGTGACCCGTCGTCTAGAGCTAAAGTAGATTAGTAACTCTAAGAG
GAGTAACATCCTCTTACTGTTCTAATCCAAATAGATAGAATGGGGGCCCCCGATGTCATT
GTATCCGGTATATAA**TAA**CCTATTATATACCGTGATTCCAACAACACAGAAACTAAA**ATG**
AAAAGCAAACAACAATTATTAATCCTTATCGGATGAGTAATTCGTTGGTACTTTTCTTCG
GTTCCTGAGTCGCGAAATGCTTATAGATACTGAAAACATCTATGAGCTCGGTACGAAGAG
TCCCGAGGGTTAGAATGAACGGTTAAACGTTTCAAACTAATCCGATTATGCGTGACCAGA
TACATATCTGGAAAACCGCATTATCCTACTGATGTAACAATCGGCATGACGAAAGATGGA
CTTCCAAAGGCTTTAGGGCCTTTAAAGTCTCTCGTTCGTTCCAGGGACATACAGAGTCTA
AGATTAGTCCTTACCCTACTTTACGTAGGCCGGGCCTTTAGACCGAGTACGTTAAATCCT
GATTTAAAGAGTATCACTACTCCTTCATCAGCGAATCCTGAACTGATTGAAGATATATAT
CAATTCGCAAAGAACTCGCGAGTTGATCTACATCTTTCATCTATAGATACGGACTTTAGG
GCTTTTCACTTTACAGAGAAGGCCGGCCCGTCTGGACATGGTCTCCTAGCATGTTATAAT
GATTATTATAACTTGACAGAGGACTTGAAACAAGATTTGCTTCTTATTTCAAACCAGACG

ATTGGAGAGCATTTCAACAATGTTGAGAATACTCATCCAACGTTGCTTGATCACTTTCGG
ACCAATTGGGTTAAGAAGACCGAAGACGGTCAACTTTCCCCCTTTAGATCTAAACTCTTA
CGAGTTATAAGTCTAAAGCCCGATCGTGAGTACAAGACTAGGCCGTTTGCTTTATTAGAC
TATTGAAGTCAGACTTGTTTAAAACCAATACATGACAAGATATTCGGTATCTTGAAACAG
TATGGTAAAACAGACTGTACATTCGGTCATAATGAAGCAATGAACATAGCGTCTAATATT
CCTAATCCTGATGGATCTCAATTCCATAGTCTTGATCTCTCAAGCGCTACAGATAGATTT
CCAATGGACCTTCAAGTTAAGATTTTATCACTCATGATTGGTGAGGATAAAGCCGAGGCT
TGAAGGAGAATATTGGTTAGCAAAGAGTATACAGTACTCTCGACTGGGGCGAAGGTTCGT
TACCTCGCCGGACAGCCGATGGGCGCGTATAGCTCTTGAGCTATGTTCACCTTATGCCAC
CACATAGTGGTTAGGTATGCGGCGCGTAATGCGAAGTATGACCGATACACAATAATTGGC
GATGATATAGTTATTAGGAACGACGACGTTGCCGAACGCTACAAGAATATCGTAAACCAA
TTAGGTGTAACCATTTCAGAGGCAAAGTCTCATGTGTCACCCGACACATTTGAAATCGCT
AAGAAATGGTACTACCGTGGAAAGGAGATTACCCCTTTTCCAATCGATGGTATGGAGAAG
ATATGAAAGTCTTACGACCTTTTATTCTTATTCCTACTAGACCTTAAAAGAAACAAAGGT
CTAGAGCCACGGTTTGGAGTGGAATCTCTAGCGATGATTAGATCTCTCTGATCAAGACTA
GGGAAACCCCCACGACTAGTACGGTCTGTCTGTAATGGGTACAGACGCTTTGCAGCCTTT
CCGTTCTTTGGAGAGAAGGACGCTGACTATAAGGATCCTAGGGGATTCTTAGAATCAGTT
AACTTCCCAAGTGCAGGACTAATGAGTCAAGAGTCAGCAAAGGCTCTTATTACATCAGTT
ACTGCCGGCCAATTAACGAACTCCCTTGTTAATCAGTTGTTTACGTACCAAATGGACATA
AACACTATAACAAAGAGAGCTTACGAGTCTGTTTCCATCTTGGGATACGGACCGTTTGCT
GAAGGGCTGGAGGACAAGACCATTGATAATCATCCATTCTTTGCATCGCAGAAGGCGATG
GTTAAAGATAGTCTGAACATCCTTACGGAGGCCAAGGAGTCTGCTTCCAAGAATAACTTT
GATGACATAATAAATATGAAACCACTGATATTCTTAAGCAACCCAACGGCTAGGTTGAGT
GGAGACCGAGAGGTTGAAATCCTCGGTTCTCAAGCAGTCCTAGTTCACTCCGCGGTAGCC
GCCCTTAAAGGGATCATGCTAATCCGTGAACTCGTTTTAACAAACGAGGAAGATTCACAG
ACTGAGCAATGAGTTCTTCGAAGATATACTGACGATTTTAACATCGTCCGAAAACTTGAG
AACGTGCTGAAGAATTTCATCTTTGATGAAGTTCTTCCACGTGCCAAGTTTAAAGCCTTC
ATGAAGGACTTTAAATCGCGAAGTCGAGTTAAATCACTTTTAGATGAT**TAG**TCTCTCCCT
ATGAAGGGGGTCTTTCCTCGTCAAAATCTTAAGATGATGATGAGAGAGGGGCTCTCAATA
CCAAATTAAAAGGCTCATAACCTTTGGCTAGCAATAGTTTATTCGCCGGGACAGTTCATG
ACTGTCGGGGATAGTATCCCCTGTTAGACGACCTCGTGAGAGGTCG

>EnmuMV2-KVL-14-117
CCCGCGTCGAAACGCAGGGCAAGACTCACCTCTGAGTTATTTCCTAGAATGGCCAATCCT
AGGTGGATGACGCACCTTTTCCTTAAGGAAAAGCGATAGCACACCTTCGTGTTACTTTCG
TAGTTTTAGTTAATTAGATACTACCACCAGCTCACGATGGTGCCCTGAAACAATGGGGCG
TTACATCATGACGCGTAGGCGGGATAGGGCTAGTCCCCCGAGTCTCGACAGGATGTCGAA
ACGCTGGAAC**TAG**GAACACGAGAAGCCTCCAAAAATACAAAAACATATCACTACAAATAA
TATA**ATG**ATGAACAAAATTTTCGCCATTTATAAATGGGTAATCAAAATGATCATCAAAAA
TCAAAAGTATGTAATATATATTGATTTTTGAAAAGAGCAAACAAACAAGTGATTAAACAC
GCGAGGATCTTTTAACACAGTCAAAAGGATTAAATCCATGAGACTACATGTTACAAGATT
CTGGTGCGGACAACCACTTATGGAATCTGGTGACCCATCTATCGGTCTAGATTCAAGGGG
ATTACCCAAGAATTTAGGTATACTTAGAGAGTTAATCCTAAGTAATGATATTTGGACAG
AAGGGTAGCTCATACTTTATTGTTAATAAGTAGAGCCATTCCTTGCAATGGTGAGATCTC

GGTAACGAGTATCACTGACAAGGGGCCGGATGTCAACCCGGACCTCTTGAAAGAGTATAG
TTCTGTTTTAAAAGAACTAAACTGAACCATTCCAGTTCCAACTTGGTCTGAATGCCATTT
GTCTACAAAGTCAGGCCCAAACTCCCAGGCGATGATTGGATCAATTGCTGATCTTAATCA
TCTACCCCAAAGCTTGAGAGACGATATATCAGTTCTCGGAGGTCCAGAACTAACCAAAGT
TATGGATTTACTCTTTGAAAAGATAAATGTCAGAGCCTGAAACAGACACTTCAAGGTCAA
AGACAAGTCTTTGATCAGGAAGCTATCTGTTGTTTCTGACCCAGAGGGAAAAGAGCGAGT
AATCGCTATTTTCGATTATTGGTCACAAACTGCACTTAAACCCCTTCATGATAACTTGTT
TGAGTTATTATCTAGGATAAAAGGAGATTGTACCTTTAATCACTCCGCTGGGAGAAACCT
TTCGGACAAGGCCAAGGGTCCGTACTATTCTATGGACTTAACTGCTGCTACAGACCGATT
CCCAATTTGGGTACAACGGCATGTAATCAGTGAGTTAATCTCCGTAGAATACGCAGACTC
CTGATCTCGGGTCATGACCTCTCACGAGTTTTCTGCTCCATTTGCTGACTCGCCCATAAA
CTATGGGGTAGGCCAGCCGATGGGTGCATACTCGTCGTGATCGGCATTTTCCCTATGCCA
TCATCTGACTGTTAGAATTGCTGCGAAAAGGGCTGGATTATCCAGCTCCTGGTCTAAGTA
TATTCTATTAGGAGATGATATAGTTCTAACTAATCCAAAAGTTGCTGATGAATATCAGAA
ACTGATGGCTACGTTAGGAGTATCACTCTCTCCTACAAAGACACATGTATCAAAAGATAC
ATATGAATTTGCGAAAAGATGATACCACGAAGGTAATGAGATCACCGGAATTCAACTTGG
ACCGTTTTTGGCGGCCAAGAATTGGGGCGGATTTGCATCTGCCCTACATGAATTCACAAG
GTGAGGCATCAAACCTCATGAGGTGGAACCCGGGTCGATAGCTGCTGGACTTAGAGCTCT
TGGTTTTGAAAGACTAAGAGATACTCATAAGATCCAAACTTTTCTACACATCCCCACAAA
GGGGGACTCACCAGAAGATCGAAGTGAGAAGTCAATATGACTAATCAACAAGTTCTTCAA
AAGTGAGGTCGGCTGCAACAGACAGGTGACGTTTAAAGAGAAATTTGTTCTCCAAACGCT
CGCTGAAGTTAAAACAGCCTGTATAGAAACTGCTATCAAGCTTATAGCAAAGGAACTCTC
TGCTTGGCAACAGAGTCTTCCCGAGCTAGCAAGCTTAGGATTGGTTGACCAAACTGTACT
ATTAGCTATGCCACCCGTAGCCGTCTGCCGGAAAGCAGCTTTAGAGCTGCAATCTGGGTT
TGACAAGCTTCGGAGTGCTTACTGGGATCTCGATGAAGACATAGTCTTCGGAGAGATACC
GATAAGAGGAGTTAATCCCTCTCGGGTTAATTCCGTACGAGCTAAAGAAGTTTTACTAGC
AACCAAAGTTCTGATCACTAATAGATACTCTCGTTGATCACGAGATTACCGGTCATCTCG
AGACCACCTACTCTCTGACAAAGAGTACAGTGGCCGTCTAAATGACAAACTTCCAACGGG
GGAATCAAATTCTAGCCAAGAATTTGGAGAA**TAG**TTTCTCACGAAACTATTCCGGGATCC
CCGTTGAGTATTGCCCTTTCT
>EnmuMV2-KVL-14-118
CGCAGGGCAATACTCACCTCTGAGTTATTTCCTAGAATGGCCAATCCTAGGTGGATGACG
CACCTTTTCCATAAGGAAAAGCGATAGCACACCTTCGTGTTACTTTCGTAGTTTTAGTTA
ATTAGATACTACCACCAGCTCACGATGGTGCCCTGAAACAATGGGGCGTTACATCATGAC
GCGTAGGCGGGNTAGGGCTAGTCCCCCGAGTCTCGACAGGATGTCGAAACGCTGGAAC**TA**
GGAACACGAGAAGCCTCCAAAAATACAAAAACATATCACTACAAATAATATA**ATG**ATGAA
CAAAATTTTCGCCATTTATAAATGGGTAATCAAAATGATCATCAAAAATCAAAAGTATGT
AATATATATTGATTTTTGAAAAGAGCAAACAAACAAGTGATTAAACACGCGAGGATCTTT
TAACACAGTCAAAAGGATTAAATCCATGAGACTACATGTTACAAGATTCTGGTGCGGACA
ACCACTTATGGAATCTGGTGACCCATCTATCGGTCTAGATTCAAGGGGATTACCCAAGAA
TTTAGGTATACTTAGAGAGTTAATCCTAAGTAATGATATTTGGGACAGAAGGGTAGCNNN
NNNNNNNNNNNNNNNNNNNNNNGCCATTCCTTGCAATGGTGAGATCTCGGTAACGAGTAT
CACTGACAAGGGGCCGGATGTCAACCCGGACCTCTTGAAAGAGTATAGTTCTGTTTTAAA
AGAACTAAACTGAACCATTCCAGTTCCAACTTGGTCTGAATGCCATTTGTCTACAAAGTC

AGGCCCAAACTCCCAGGCGATGATTGGATCAATTGCTGATCTTAATCATCTACCCCAAAG
CTTGAGAGACGATATATCAGTTCTCGGAGGTCCAGAACTAACCAAAGTTATGGATTTACT
CTTTGAAAAGATAAATGTCAAAGCCTGAAACAGACACTTCAAGGTCAAAGACAAGTCTTT
GATCAGGAAGCTGTCTGTTGTTTCTGACCCAGAGGGAAAAGAGCGAGTAATCGCTATTTT
CGATTATTGGTCACAAACTGCACTTAAACCCCTTCATGATAACTTGTTTGAGTTATTATC
TAGGATAAAAGGAGATTGTACCTTTAATCACTCCGCTGGGAGAAACCTTTCGGACAAGGC
CAAGGGTCCGTACTATTCTATGGACTTAACTGCTGCTACAGACCGATTCCCAATTTGGGT
ACAACGGCATGTAATCAGTGAGTTAATCTCCGTAGAATACGCAGACTCCTGATCTCGGGT
CATGACCTCTCACGAGTTTTCTGCTCCATTTGCTGACTCGCCCATAAATTATGGGGTAGG
CCAGCCGATGGGTGCATACTCGTCGTGATCGGCATTTTCCCTATGCCATCATCTGACTGT
TAGAATTGCTGCGAAAAGGGCTGGATTATCCAGCTCCTGGTCTAAGTATATTCTATTAGG
AGATGATATAGTTCTAACTAATCCAAAAGTTGCTGATGAATATCAGAAACTGATGGCTAC
GTTAGGAGTATCACTCTCTCCTACAAAGACACATGTATCAAAAGACACATATGAATTTGC
GAAAAGATGATACCACGAAGGTAATGAGATCACCGGAATTCAACTTGGACCGTTTTTGGC
GGCCAAGAATTGGGGCGGATTTGCATCTGCCCTACATGAATTCACAAGGTGAGGCATCAA
ACCTCATGAGGTGGAACCCGGGTCGATAGCTGCTGGACTTAGAGCTCTTGGTTTTGAAAG
ACTAAGAGATACTCATAAGATCCAAACTTTTCTACACATCCCCACAAAGGGGGACTCACC
AGAAGATCGAAGTGAGAAGTCAATATGACTAATCAACAAGTTCTTCAAAAGTGAGGTCGG
CTGCAACAGACAGGTGACGTTTAAAGAGAAATTTGTTCTCCAAACGCTCGCTGAAGTTAA
AACAGCCTGTATAGAAACTGCTATCAAGCTTATAGCAAAGGAACTCTCTGCTTGGCAACA
GAGTCTTCCCGAGCTAGCAAGCTTAGGATTGGTTGACCAAACTGTACTATTAGCTATGCC
ACCCGTAGCCGTCTGCCGGAAAGCAGCTTTAGAGCTGCAATCTGGGTTTGACAAGCTTCG
GAGTGCTTACTGGGATCTCGATGAAGACATAGTCTTCGGAGAGATACCAATAAGAGGAGT
TAATCCCTCTCGGGTTAATTCCGTGCGAGCTAAAGAAGTTTTACTAGCAACCAAAGTTCT
GATCACTAATAGATACTCTCGCTGATCACGAGATTACCGGTCATCTCGAGACCACCTACT
CTCTGACAAAGAGTACAGTGGCCGTCTAAATGACAAACTTCCAACGGGGGAATCAAATTC
TAGCCAAGAATTTGGAGAA**TAG**TTTCTCACGAAACTATTCCGGGATCCCCGTTGAGTATT
GCCCTTTC
>EnmuMV2-HHdFL130914-1
CCCGCGTCGAAACGCAGGGCAATACTCACCTCTGAGTTATTTCCTAGAATGGCCAATCCT
AGGTGGATGACGCACCTTTTCCTTAAGGAAAAGCGATAGCACACCTTCGTGTTACTTTCG
TAGTTTTAGTTAATTAGATACTACCACCAGCTCACGATGGTGCCCTGGAACAATGGGGCG
TTACATCATGACGCGTAGGCGGGATAGGGCTAGTCCCCCGAGTCTCGACAGGATGTCGAA
ACGCTGGAAC**TAG**GAACACGAGAAGCCTCCAAAAATACAAAAACATATCACTACAAATAA
TATA**ATG**ATGAACAAAATTTTCGCCATTTATAAATGGGTAATCAAAATGATCATCAAAAA
TCAAAAGTATGTAATATATATTGATTTTTGAAAAGAGCAAACAAACAAGTGATTAAACAC
GCGAGGATCTTTTAACACAGTCAAAAGGATTAAATCCATGAGACTACATGTTACAAGATT
CTGGTGCGGACAACCACTTATGGAATCTGGTGACCCATCTATCGGTCTAGATTCAAGGGG
ATTACCCAAGAATTTAGGTATACTTAGAGAGTTAATCCTAAGTAATGATATTTGGGACAG
AAGGGTAGCTCATACTTTATTGTTAATAAGTAGAGCCATTCCTTGCAATGGTGAGATCTC
GGTAACGAGTATCACTGACAAGGGGCCGGATGTCAACCCGGACCTCTTGAAAGAGTATAG
TTCTGTTTTAAAAGAACTAAACTGAACCATTCCAGTTCCAACTTGGTCTGAATGCCATTT
GTCTACAAAGTCAGGCCCAAACTCCCAGGCGATGATTGGATCAATTGCTGATCTTAATCA
TCTACCCCAAAGCTTGAGAGACGATATATCAGTTCTCGGAGGTCCAGAACTAACCAAAGT

TATGGATTTACTCTTTGAAAAGATAAATGTCAAAGCCTGAAACAGACACTTCAAGGTCAA
AGACAAGTCTTTGATCAGGAAGCTATCTGTTGTTTCTGACCCAGAGGGAAAAGAGCGAGT
AATCGCTATTTTCGATTATTGGTCACAAACTGCACTTAAACCCCTTCATGATAACTTGTT
TGAGTTATTATCTAGGATAAAAGGAGATTGTACCTTTAATCACTCCGCTGGGAGAAACCT
TTCGGACAAGGCCAAGGGTCCGTACTATTCTATGGACTTAACTGCTGCTACAGACCGATT
CCCAATTTGGGTACAACGGCATGTAATCAGTGAGTTAATCTCCGTAGAATACGCAGACTC
CTGATCTCGGGTCATGACCTCTCACGAGTTTTCTGCTCCATTTGCTGACTCGCCCATAAA
CTATGGGGTAGGCCAGCCGATGGGTGCATACTCGTCGTGATCGGCATTTTCCCTATGCCA
TCATCTGACTGTTAGAATTGCTGCGAAAAGGGCTGGATTATCCAGCTCCTGGTCTAAGTA
TATTCTATTAGGAGATGATATAGTTCTAACTAATCCAAAAGTTGCTGATGAATATCAGAA
ACTGATGGCTACGTTAGGAGTATCACTCTCTCCTACAAAGACACATGTATCAAAAGATAC
ATATGAATTTGCGAAAAGATGATACCACGAAGGTAATGAGATCACCGGAATTCAACTTGG
ACCGTTTTTGGCGGCCAAGAATTGGGGCGGATTTGCATCTGCCCTACATGAATTCACAAG
GTGAGGCATCAAACCTCATGAGGTGGAACCCGGGTCGATAGCTGCTGGACTTAGAGCTCT
TGGTTTTGAAAGACTAAGAGATACTCATAAGATCCAAACTTTTCTACACATCCCCACAAA
GGGGGACTCACCAGAAGATCGAAGTGAGAAGTCAATATGACTAATCAACAAGTTCTTCAA
AAGTGAGGTCGGCTGCAACAGACAGGTGACGTTTAAAGAGAAATTTGTTCTCCAAACGCT
CGCTGAAGTTAAAACAGCCTGTATAGAAACTGCTATCAAGCTTATAGCAAAGGAACTCTC
TGCTTGGCAACAGAGTCTTCCCGAGCTAGCAAGCTTAGGATTGGTTGACCAAACTGTACT
ATTAGCTATGCCACCCGTAGCCGTCTGCCGGAAAGCAGCTTTAGAGCTGCAATCTGGGTT
TGACAAGCTTCGGAGTGCTTACTGGGATCTCGATGAAGACATAGTCTTCGGAGAGATACC
AATAAGAGGAGTTAATCCCTCTCGGGTTAATTCCGTACGAGCTAAAGAAGTTTTACTAGC
AACCAAAGTTCTGATCACTAATAGATACTCTCGCTGATCACGAGATTACCGGTCATCTCG
AGACCACCTACTCTCTGACAAAGAGTACAGTGGCCGTCTAAATGACAAACTTCCAACGGG
GGAATCAAATTCTAGCCAAGAATTTGGAGAA**TAG**TTTCTCACGAAACTATTCCGGATCC
CCGTTGAGTATTGCCCTTTCTTCC
>EnmuMV2-Berkeley
CCCGCGTCGAAACGCAGGGCAATACTCACCTCTGAGTTATTTCCTAGAATAGCCAATCCT
AGGTGGATGACGCACCTTTTCCGTAAGGAAAAGCGATAGCACACCTTCGTGTTACTTTCG
TAGTTTTAGTTAAT**TAG**ATACTACCACCAGCTCACGATGGTGCCCTGGAACAATGGGGCG
TTACATCATGACGCGTAGGCGGGATAGGGCTAGTCCCCCGAGTCTCGACAGGATATCGAA
ACGCTGGAACTAGGAACACGAGAAGCCTCCAAAATACAAAACATATCACTACAAATAATA
TA**ATG**ATGAACAAAATTTTCGCCATTTATAAATGGGTAATCAAAATGATCATCAAGAATC
AAAAGTATGTAATATATATTGATTTTTGAAAAGAGCAAACAAACAAGTGATTAAACACGC
GAGGATCTTTTAACACAGTCAAAAGGATAAAATCCATGAGACTTCATGTTACAAGATTCT
GGTGCGGACAACCACTTATGGAATCTGGAGACCCATCTATCGGTCTAGACTCTAGGGGTT
TACCCAAGAATCTAGGTATACTTAGAGATTTAATTCTAAGTGACGATATCTGGGACAGGA
GGGTAGTTCATACTTTATTGTTAATAAGTAGAGCTATCCCTTGTAATGGTGAGATCTCGA
TATCGAGTATCACTGACAAGGGGCCGGATGTCAACCCGGATCTCTTGAAAGAGTATAGTT
CTGTTTTAAAAGAACTAAACTGAACCATTCCAGTTCCAACTTGGTCTGAATGCCATTTGT
CTACAAAGTCGGGCCCAAACTCCCAGGCAATGATCGGATCAATTGCTGATCTTAATCATT
TACCCCAAAGCTTGAGAGACGATATATCTGTTCTCGGAGGTCCAGAACTAACCAAAGTTA
TGGATTTACTCTTTGAAAAGATAAATGTCAGAGCCTGAAACAGACATTTCAAAGTTAAAG
ACAAGTCTTTAATTAGGAAGTTATCTGTTGTTTCTGACCCAGAGGGAAAAGAGCGAGTAA

TCGCTATTTTCGATTATTGGTCACAAACTGCACTTAAACCCTTTCATGATAACTTATTTG
AGTTATTATCAAAGATAAAAGGAGATTGTACCTTCAATCATTCCGCTGGGAGAAACCTTT
CGGACAAGGCCAAGGGTCCGTACTATTCCATGGATCTAACAGCTGCTACAGACCGGTTCC
CAATTTGGGTACAACGGCATGTAATAAGCGAGTTAGTCTCCGTAGAATACGCAGACTCCT
GATCTCGGGTCATGACCTCTCACGAGTTTTCCGCTCCATTTGTTGACTCGCCCATAAATT
ATGGGGTAGGTCAGCCGATGGGAGCATACTCGTCGTGATCGGCATTTTCCCTATGCCATC
ATCTTACCGTTAGAATTGCTGCGAAAAGGGCTGGGTTATCCAGTTCCTGATCTAAGTACA
TTCTATTGGGAGATGATATAGTTCTAACTAATCCAAAAGTTGCTGATGAATATCAAAAAC
TAATGGCTACGTTAGGAGTATCACTCTCTCCTACAAAGACACATGTGTCAAAAGACACAT
ATGAATTTGCAAAAAGATGATACCACGAAGGTAATGAGATCACCGGAATTCAACTTGGTC
CGTTTTTGGCGGCCAAGAATTGGGGCGGATTTGCATCCGCTCTACATGAATTCACAAGGT
GAGGCATCAAACCTCATGAGGTGGAACCCGGGTCGATAGCTGCTGGACTTAGGGCTCTTG
GTTTTGATAGACTAAGAGATACCCATAAGATCCAAACTTTTCTACACATCCCTGCAAAGG
GAGATTCACCCGAAGAACGAAGTGAAAAGTCAATTTGACTTATCAATAAGTTCTTTAAAA
GTGAAGTCGGCTGCAACAGACAGGTGACGTTTAAAGAGAAATTTGTTCTCCAAACGCTCG
CTGAAGTTAAAACAGCTTGTATAGAAACTGCTATCAAGCTTATAGCAAAGGAACTCTCTG
CTTGGCAACAGAGTCTTCCAGAGCTAGCAAGCTTAGGATTGGTTGACCAAACTGTACTAT
TAGCAATGCCACCTGTAGCTGTCTGCCGGAAAGCAGCATTAGAACTGCAATCTGGGTTCG
ACAAACTACGGAGTGCTTACTGAGATCTTGATGAAGACATTGTCTTCGGAGAGATTCCAA
TAAGAGGAATTAATCCCTCTCGGGTTAATTCCGTACGTGCTAAAGAAGTTTTACTAGCAA
CCAAAGTTCTGATCACTAATAGATACTCTCGCTGATCACGAGATTACCGGTCATCTCGAG
ACCACTTACTCTCTGACAAAGAGTATAGCGGTCGTCTAAATGACAAACTTCCAACGGGGG
AATCAAATCCTAGC**TAA**GGATTTGAAGAATAGTTTCTCACGAAACTATTCCGGGATCCCC
GTTGAGTATTGCCCTTTCTTCC

>EnmuMV3-KVL-14-117
CCTCCTTTAGGGAGGCCACGTCTGTTCTACCCCCCATTGCTGGGCTAGTAGAAAACGCTT
TCGCGCGTGCCATTGCTGACTTATCAGTAATCTCCGCGATAGATCCAGTTGGTGATAACT
TCAACAGGATCCCTTGATAATACAAGGTTAGTCGACTTGTCACCACATAGAAAGGTTCTA
GATATTTCACTAGTTTAGTCTTTCTATCAATTCAGATTTAAACCTGAATAAATTAGCAAT
AAGTAGTACAGTTGATGTACGAAAGAAAGGTTTGAAACTTCCTTCCCACAATAGAGCACA
TAAACAATACTTACTATC**ATG**AAACTTAATACTACACAAAGACTCCATATTCTTATGAAA
TGGGTTCTAAGGAAGTACTTCGGTTCAAGTCAAGTATATCAAGACTCGTATCTTCACTTT
GTGAAGATATGGAACAGGTACGAATCAAAGTGTGGAACTGTTTGGACCGTTAAACGGTTC
AAGCTTATCAGACTAGGCGTTACAAAATACCTAGCCGGACAAGTTCTCACTCCGACCGAA
TGCCTAGGGATGACCAAAGATGGTCTTCCCAAAGCTTTAGGTCCGATGAAGGACCTGATT
CGATCAAGAGATCCTCAGAACATAAGGTTCGTCCTAACAATATTATATGTTGGAAGGGCT
TATAGACCTAAGGAAGTATCGGGTTTCAACCAAGCGACTGTAAGAGATCTCTCTCGAGCA
GACGCCAATGTTGTAACTCAGATCACTGATTTTATTCAACATAATGTTTTAAATAAATCT
TCAGTGATGGCTTTTGACCCGAACTGAGATCAGTTTCACTTTACAGAGAAGGCGGGGCCA
GCTGGCCACGCCCTTCTGACTTCGATTACGAATTTAAGATCTCTTCCTAAGGAGTTATTA
AACGATATAATAACTCTAGGAGGAGAAAGATTAGAAGAAGCAATTGAAAAAGTGCTTGCG
ACTAATCCGGTCGTTCTTGACTATCTAGACTATCACAATGCAAAAGGTGATAGAAGAGAC
AAGATCAGAGCGATATCTCTTAAACCTGATCGAGAAATGAAAGTGAGACCGTTTGCTTTA

CTAGATTACTGGAGTCAGACATGTCTGAAACCAGTCCACGATAATCTGTTCGAAATTCTT
AAAAGAATCAAAACAGATCATACGTTTGATCACGGTAAAGCTATACAGGCTGTTAAGAAT
CTTAAACCAAATGGTTCTTATCATAGCCTCGATTTATCGAGTGCTACAGATAGATTTCCT
ATGGAACTTCAGCATAATCTAATGAAACTCCTTATTGGAGAAACAAAAGCTAATGCTTGA
AGAAGGATCTTAACAGGTCATGAGTTTGAGGTCTTCCAAACAGGTGATAAGCTCCAGTAC
GGAGCCGGTCAACCGATGGGGGCCTACAGCTCATGAGCTGTATTTACGGTCTGTCACCAT
ATTGTTATATGATATTGTGCCGGTTCAACCGCGTACAGTAACTATGCCATTATTGGTGAT
GACGTCGTTATCGCAGATGATGACGTAGCTGATCGGTATGTAGAAGTGATCAAGGCCCTT
GGGGTTGACATCTCTCCATCTAAATCTCATGTGTCCAAGGACACATTCGAAATAGCGAAG
AGATGATCCTTCAAGGGGACCGAGGTAACACCTTTCCCGATCGACGGTTTATCGGTTATA
TTCAATAAACCAGACATCCTTTACTTATTCTTATTAGAACAAGAAAAGAAAGGCTACATA
GCAAAGGGAGGGATCGAATCCGCCTCTTCAATATCGTCATTGCTGCAACTCTTCGGACTG
CCGGAGCGTAAAATACGTTCGGCAGCGAAGCTTTACAGAATGATTGCCTTGTTTCCCTTT
ATCGGTAATAGACAAGGAGATTATAAAGATCCGAAAGGATTTTATCATCTTCTTGGCTTT
CCTGTATCATGTAACGTGTCAAACGACATTTACATGAATGAGCTTAAAGCTTTTGCAGGA
AGAGCAATCTTTAACCAAGTAGGGGGAAGAAAAGGTAAGATATGAAAGGCAGCAACATAC
GGCTTTTCGGTAGAATTTCTATCGAATTGACTTGTAGTTGCTCCCGGATTCGGGCCACAT
GAGTCAAATCAAGAGGCATCACTTTATCATCTACCCCAAGTGGGGTCTATGCTCTCACTA
AGTCGAAATCTGCAAGCAGAATTCAACGAAGTAAGAATGATGGAAATAGATGAGAACTGA
GATTATTTTCTAAACAGAAAAGTATCAATCCTTATCTCTGACCCGGTCATCGTGTTTAGC
GGTGATCGGTCTCTTGAGGTCTTGGGTACTCAGGCTCAAATCGTACGTTGAGCGGTAGAA
GGAATTAAATCCGGATACCAAACTCGTATCGACGAACTGAACGCCCCTTCCGAAGAGTCA
TTCCAAGATGATTCAGGAATATCAGACAGCGGGGTCATTATGTTG**TAA**GGTATTATCCTT
ACTCATAACGCCCGTTGTCCTCCTGAGTTCATTTCATAATGAAATGGCACCGAGAGGTGT
GGGGTTCCTTTGGAAAGAATCCCGGACGTG
>EnmuMV3-KVL-14-118
TCGCGCGTGCCATTGCTGACTTATCAGTAATCTCCGCGATAGATCCAGTTGGTGATAACT
TCAACAGGATCCCTTGATAATACAAGGTTAGTCGACTTGTCACCACATAGAAAGGTTCTA
GATATTTCACTAGTTTAGTCTTTCTATCAATTCAGATTTAAACCTGAATAAATTAGCAAT
AAGTAGTACAGTTGATGTACGAAAGAAAGGTTTGAAACTTCCTTCCCACAATAGAGCACA
TAAACAATACTTACTATC**ATG**AAACTTAATACTACACAAAGACTCCATATTCTTATGAAA
TGGGTTCTAAGGAAGTACTTCGGTTCAAGTCAAGTATATCAAGACTCGTATCTTCACTTT
GTGAAGATATGGAACAGGTACGAATCAAAGTGTGGAACTGTTTGGACCGTAAAACGGTTC
AAGCTTATCAGGTTAGGCGTTACAAAATACCTAGCCGGACAAGTTCTCACTCCGACCGAA
TGCCTAGGGATGACCAAAGATGGTCTTCCTAAAGCTTTAGGTCCGATGAAGGACCTGATT
CGATCAAGAGATCCTCAGAACATAAGGTTCGTCCTAACAATATTATATGTTGGGAGGGCT
TATAGACCTAAGGAAGTATCGGGTTTCAACCAAGCGACTGTAAGAGATCTCTCTCGAGCA
GACGCCAATGTTGTAACTCAGATCACTGATTTTATTCAACATAATGTTTTAAATAAATCT
TCAGTGATGGCTTTTGACCCGAACTGAGATCAGTTTCACTTTACAGAGAAGGCGGGGCCA
GCTGGCCACGCCCTTCTGACTTCGATTACGAATTTAAGATCTCTTCCTAAGGAGTTATTA
AACGATATAATAACTCTAGGGGGAGAAAGATTAGAAGAAGCAATTGAAAAAGTGCTTGCG
ACTAATCCGGTCGTTCTGGACTATCTAGACTATCACAATGCAAAAGGTGATAGAAGAGAC
AAGATCAGAGCGATATCTCTTAAACCTGATCGAGAAATGAAAGTGAGACCGTTCGCTTTA
CTAGATTACTGGAGTCAGACATGTCTGAAACCAGTCCACGATAATCTGTTCGAAATTCTT

AAAAGAATCAAAACAGATCATACGTTTGATCACGGTAAAGCTATACAGGCTGTTAAGGAT
CTTAAACCAAATGGCTCTTACCATAGCCTCGATTTATCGAGTGCTACGGATAGATTTCCT
ATGGAACTTCAGCATAATCTAATGAAACTCCTTATTGGAGAAACAAAAGCTAATGCATGA
AGAAGGATCCTAACAGGTTATGAGTTTGAGGTCTTCCAAACAGGTGATAAGCTCCAATAC
GGAGCCGGTCAACCAATGGGGGCCTATAGCTCATGAGCTGTATTTACGGTCTGTCACCAT
ATTGTTATATGATATTGTGCCGGTTCAACCGCGTACAGTAACTATGCCATTATTGGTGAT
GACGTCGTTATCGCAGATGATGACGTAGCTGATCGGTATGTAGAAGTGATCAAGGCCCTT
GGGGTTGACATCTCTCCATCTAAATCTCATGTGTCCAAGGACACATTCGAAATAGCGAAG
AGATGATCCTTCAAGGGGACCGAGGTAACACCTTTCCCGATCGACGGTTTATCGGTTATA
TTCAATAAACCAGACATCCTTTACTTATTCTTATTAGAACAAGAAAAGAAAGGCTACATA
GCAAAGGGAGGGATCGAATCCGCCTCTTCAATATCGTCACTGCTGCAACTCTTCGGACTG
CCGGAGCGTAAAATACGTTCGGCAGCGAAGCTTTACAGAATGATTGCCTTGTTTCCCTTT
ATCGGTAATAGACAAGGAGATTATAAAGATCCGAAAGGATTTTATCATCTTCTTGGCTTT
CCTGTATCATGTAACGTGTCAAATGACATCTACATGAATGAGCTTAAAGCTTTTGCAGGA
AGAGCAATCTTTAACCAAGTAGGGGGAAGAAAAGGTAAGATATGAAAGGCAGCAACATAC
GGCTTTTCGGTAGAATTTCTATCGAACTGACTTGTAGTTGCTCCCGGATTCGGGCCACAT
GAGTCAAATCAAGAGGCATCACTTTATCATCTACCCCAAGTGGGGTCTATGCTCTCACTA
AGTCGAAATCTGCAAGCAGAATTCAACGAAGTAAGAATGATGGAAATAGATGAGAACTGA
GATTATTTTCTAAACAGAAAAGTATCAATCCTTATCTCTGACCCGGTCATCGTGTTTAGC
GGTGATCGGTCTCTTGAGGTCTTGGGTACTCAGGCTCAAATCGTACGTTGGGCGGTAGAA
GGAATTAAATCCGGATACCAAACTCGTATCGACGAACTGAACGCCCCTTCCGAAGAGTCA
TTCCAAGATGATTCAGGAATATCAGACAGCGGGGTCATTATGTTG**TAA**G
>EnmuMV3-HHdFL130914-1
CTCCTTTAGGGAGGCCACGTCTGTTCTACCCCCCATTGCTGGGCTAGTAGAAAACGCTTT
CGCGCGTGCCATTGCTGACTTATCAGTAATCTCCGCGATAGATCCAGTTGGTGATAACTT
CAACAGGATCCCTTGATAATACAAGGTTAGTCGACTTGTCACCACATAGAAAGGTTCTAG
ATATTTCACTAGTTTAGTCTTTCTATCAATTCAGATTTAAACCTGAATAAATTAGCAATA
AGTAGTACAGTTGATGTACGAAAGAAAGGTTTGAAACTTCCTTCCCACAATAGAGCACA**T**
AAACAATACTTACTATC**ATG**AAACTTAATACTACACAAAGACTCCATATTCTTATGAAAT
GGGTTCTAAGGAAGTACTTCGGTTCAAGTCAAGTATATCAAGACTCGTATCTTCACTTTG
TGAAGATATGGAACAGGTACGAATCAAAGTGTGGAACTGTTTGGACCGTTAAACGGTTCA
AGCTTATCAGACTAGGCGTTACAAAATACCTAGCCGGACAAGTTCTCACTCCGACCGAAT
GCCTAGGGATGACCAAGGATGGTCTTCCTAAAGCTTTAGGTCCGATGAAGGACCTGATTC
GATCAAGAGATCCTCAGAACATAAGGTTCGTCCTAACAATATTATATGTTGGGAGGGCTT
ATAGACCTAAGGAAGTATCGGGTTTCAACCAAGCGACTGTAAGAGATCTCTCTCGAGCAG
ACGCCAATGTTGTAACTCAGATCACTGATTTTATTCAACATAATGTTTTAAATAAATCTT
CAGTGATGGCTTTTGACCCAAACTGAGATCAGTTTCACTTTACAGAGAAGGCGGGGCCAG
CTGGCCACGCCCTTCTGACTTCGATTACGAATTTAAGATCTCTTCCTAAGGAGTTATTAA
ACGATATAATAACTCTAGGAGGGGAAAGATTAGAAGAAGCAATTGAAAAAGTGCTTGCGA
CTAATCCGGTCGTTCTGGATTATCTAGACTATCACAATGCAAAAGGTGATAGAAGAGATA
AGATCAGAGCGATATCTCTTAAACCTGATCGAGAAATGAAAGTGAGACCGTTCGCTTTAC
TAGATTACTGGAGTCAGACATGTCTGAAACCAGTTCACGATAATCTGTTCGAAATTCTTA
AAAGAATCAAAACAGATCATACGTTTGATCACGGTAAAGCTATACAGGCTGTTAAGAATC
TTAAACCAAATGGTTCTTACCATAGCCTCGATTTATCGAGTGCTACGGATAGATTCCCTA

TGGAACTTCAGCATAATCTAATGAAACTCCTTATTGGAGAAACAAAAGCTAATGCTTGAA
GAAGGATCTTAACAGGTTATGAGTTTGAGGTCTTCCAAACGGGTGATAAGCTCCAATACG
GAGCCGGTCAACCGATGGGGGCCTATAGCTCATGAGCTGTATTTACGGTCTGTCACCATA
TTGTTATATGATATTGTGCCGGTTCAACCGCGTACAGTAACTATGCCATTATTGGTGATG
ACGTCGTTATCGCAGATGATGACGTAGCTGATCGGTATGTAGAAGTGATCAAGGCCCTTG
GGGTTGACATCTCTCCATCTAAATCTCATGTGTCCAAGGACACATTCGAAATAGCGAAGA
GATGATCCTTCAAGGGGACCGAGGTAACACCTTTCCCGATCGACGGTTTATCGGTTATAT
TCAATAAACCAGACATCCTTTACTTATTCTTATTAGAACAAGAAAAGAAAGGCTACATAG
CAAAGGGAGGGATCGAATCCGCCTCTTCAATATCGTCATTGCTGCAACTCTTCGGACTGC
CGGAGCGTAAAATACGTTCGGCAGCGAAGCTTTACAGAATGATTGCCTTGTTTCCCTTTA
TTGGTAATAGACAAGGAGATTATAAGGATCCGAAAGGATTTTATCATCTTCTTGGCTTTC
CTGTATCATGTAACGTGTCAAATGACATTTACATGAATGAGCTTAAAGCTTTTGCAGGAA
GAGCAATCTTTAACCAAGTAGGGGGAAGAAAAGGTAAGATATGAAAGGCAGCAACATACG
GGTTTTCGGTAGAATTTCTATCGAATTGACTTGTAGTTGCTCCCGGATTCGGGCCACATG
AGTCAAATCAAGAGGCATCACTTTATCATCTACCCCAAGTGGGGTCTATGCTCTCACTAA
GTCGAAATCTGCAAGCAGAATTCAACGAAGTAAGAATGATGGAAATAGATGAGAACTGAG
ATTATTTTCTAAACAGAAAAGTATCAATCCTTATCTCTGACCCGGTCATCGTGTTTAGCG
GTGATCGGTCTCTTGAGGTCTTGGGTACTCAGGCTCAAATCGTACGTTGAGCGGTAGAAG
GAATTAAATCCGGATACCAAACTCGTATCGACGAACTGAACGCCCCTTCTGAAGAGTCAT
TCCAAGATGATTCAGGAATATCAGACAGCGGGGTCATTATGTTG**TAA**GGTATTATCCTTA
CTCATAACGCCCGTTGTCCTCCTGAGTTCATTTCATAATGAAATGGCACCGAGAGGTGTG
GGGTTCCTTTGGAAAGAATCCCGGACGTGGCCTCCCTA
>EnmuMV3-Berkeley
CCTCCTTTAGGGAGGCCACGTCTGTTCTACCCCCCATTGCTGGGCTAGTAGAAAACGCTT
TCGCGCTTGCCATTGCTGACTTTATCAGTAATCATCGCGATAGATCCAGTTGGTGATAAC
TTCAACAGGATCCCTTGATAATACAAGGTTAGTCGACTTGTCATCACATAGAAAGGTTCT
AGAATTTCTCTAGTTTAGTCTTTCTATCAATTCAGATTAAAACCTGAATAAATTAGCAAT
AAGTAGTA**TAG**TTGATATACGAAAGAAAGGTTTGAAACTTCCTTCCCACAATAGAGCACA
TTACAATATCTACTATC**ATG**AAACTTAATACTACACAAAGACTCCATATTCTTATGAAAT
GGGTTCTAAGGAAGTACTTCGGTTCAAGTCAAGTATATCAAGACTCGTATCTTCATTTTG
TGAAGATATGGAACAGGTACGAATCAAAGTGTGGAACTGCTTGGACCGTAAAACGGTTCA
AACTTATCAGGCTAGGTGTTACAAAATACCTAGCCGGACAAGTTCTCACTCCGACCGAAT
GCTTAGGAATGACCAAAGATGGTCTTCCAAAAGCGCTAGGTCCGATGAAGGACCTGATCC
GATCAAGAGAACCTCAGAATATAAGATTTGTCCTTACAATATTATATATTGGAAGGGCTT
ATAGACCAAAGGAAGTATCGGGTTTCAACCAAGCGACTGTAAGAGATCTCTCTCGAGCAG
ACGCCAATGTTGTAACTCAGATCACTGATTTTATTAAACATCATGTTTTAAATAAATCTC
CAGTGATGGCTTTTGACCCGAACTGAGATCAGTTTCACTTTACAGAGAAGGCGGGACCAG
CTGGTCACGCCCTTCTAACTTCGATTACGAATTTAAGATCTCTACCAGAGGAGTTACTAA
AAGATATAATAACTCTAGGTGGAGAAAGATTAGAAGAAGCAATCGAAAGAGTGCTTATGA
CTAATCCTGTTGTCTTAGATTATCTGGACTATCACAATGCAAAAGGTGATCGTCGAAATA
AAATAAGGGCAATATCTCTTAAACCTGATCGAGAAATGAAAGTGAGACCGTTCGCTTTAC
TAGATTACTGGAGTCAGACTTGTCTGAAACCAGTTCACGATAATCTGTTTGAAATTCTTA
AACGAATTAAGACAGATCATACGTTTGATCACGGTAAAGCAATACAGTCAGTAAGAGATC
TAATTCCTAACGGATCTTATCATAGTCTTGATTTATCAAGTGCTACTGATAGGTTCCCTA

TGGAACTCCAGCACAATCTAATGAAACTCCTTATCGGAGAGACAAAAGCAGATGCATGGA
GAAGAATCTTGACTGATCATGAATTTGAAGTCTTCCAGACGGGTGACAAACTCCAATACG
GAGCTGGTCAGCCTATGGGGGCTTACAGTTCATGAGCTGTATTTACGGTCTGTCACCATA
TTGTAATATGATATTGTGCCGGTTCAACCGCGTACAGTAACTATGCCATTATTGGTGATG
ACGTCGTTATCGCAGATGATGACGTAGCTGATCGATATGTAGAAGTGATCAAGGCCCTTG
GAGTGGACATCTCTCCAGCAAAATCTCATGTATCTAAAGATACATTTGAAATTGCCAAGA
GATGGTCATTCAAGGGGACCGAGGTTACACCTTTTCCGATCGACGGTTTATCAGTTATAT
TTAATAAACCTGACATCCTTTATCTGTTCTTATTAGAACAGGAAAAGAAAGGCTACATAG
CAAAGGGAGGGATCGAATCCAGCTCTTCAATCTCATCATTACTACAACTTTTCGGACTGC
CACTACGTAAAGTACGTGCGGCAGCGAAACTTTATAGAATGATAGCCCTCTTTCCATTCA
TTGGTAACAGACAAGGAGATTATAAAGATCCGAAAGGATTTTATCATCTACTTGGCTTTC
CTGTTTCATGTAATGTGTCAAATGACGTATACATGAATGAGCTAAAAGCTTACGCAGGAA
GAGCAATCTTTAACCAAATTGGTGGAAGAAAGGGTAAGATTTGAAAAGCAGCTACATACG
GCTTTTCGGTAGAGTTTCTATCGAATTGACTAGTTGTAGCTCCAGGATTCGGGCCACATG
AGTCAAATCAAGAGGCATCACTATATCATCTACCCCAAGTGGGGTCTATGCTCTCACTAA
GTAGAAATCTGCAAGCAGAATTCAACGAAGTAAGAATGATGGAAATAGATGAGAACTGAG
ATTATTTCCTAAACAGGAAAGTTTCAATCCTTATTTCTGACCCTGTCATCGTATTTAGCG
GTGACAGATCTCTTGAGGTCTTGGGTACTCAGGCTCAAATCGTACGATGAGCGGTAGAAG
GAATTAAATCCGGATACCAGACTCGTATCGACGAACTGAACGCCCCTTCTGAAGAGTCAT
TCCAGAATGATTCAGGAATATCAGATAGTGGGGTCATTATGTTG**TAA**GGTATTATCCTTA
CTCATAACGCCCACTATCCTCCTGAGTTCATTTCATAATGAAATGGCACCGAAAGGTGTG
GGGTTCCTTTGGAAAGAATCCCGGACGTGGCCTCCCTA
>EnmuMV3-HHdFL050913-1
CTCCTTTAGGGAGGCCACGTCTGCTTTACCCCCCATTGCTGGGCTAGTAAAGAACGCTTT
CGCGCTTGCCATTGCTGACTTATCAGTAATCGCATCTGCGATAGATCCAGTTGGTGATAA
CTTCAACAGGATCCCTTGATAATACAAGGTTAGTCGACTTGTCAtCACATGGAGGTAGGG
AGAGAAATCTCTCTATATCCATCAACTCGGGATaAAATCCGAGTAAATAGGCAATAAGTA
GTACAGTTGATGTACGAAAGAAAGGTTTGAAACTTCCTTCCCACAA**TAG**ATACATATAAC
AATATTTACTATC**ATG**AAACACAACACTACACAAAGACTCCATATTCTAATGAAATGGGT
TCTAAGGAAGTATTTTGGTTCAAGTCAAGTATATCAAGACTCTTATCTTCACTTTGTGAA
GATATGGAACAGGTACGAATCGAAGTGCGGGACTGTTTGAACCGTTAAACGATTCAAAAT
GATCAGACTAAGTGTTACAAAATACTTAGCTGGTCAAGTTCTCACTCCGACGGAATGCAT
CGGGATGACCAAAGATGGTCTTCCGAAGGCTTTAGGTCCAATGAAGGACCTTATCCGTTC
TAGAGATCCTAGGAATATAAGGTTTGTCCTTACAATATTGTATATTGGAAGGGCTTATAG
ACCCAAAGAAGTTTCAGGTTTCAACCAAGCGACTGTCCGAGATCTCTCTCGAGCAGACGC
CAATGTTGTAACACAAATCACTGAGTTTATTAAACATCATGTTTTAAATAAATCACCAGT
GATGGCTTTCGACCCTAACTGAGATCAGTTCCACTTTACAGAGAAAGCGGGGCCTGCTGG
CCACGCTCTTCTTACTTCGATATCGAATTTAAGATCTCTCCCTGAAAGTTTGTTAAATGA
TATCAAACTTTTAGGTGGAGATAAGTTAGAAGAAGCAATCGAAAGAGTGCTTATGACTAA
CCCAGTCGTTCTAGATTATTTAGACGGTCACAATGCAAAGAGTGATCGGCGAAATAAAAT
AAGAGCGATATCTCTTAAACCTGATAGAGAAATGAAAGTGAGACCGTTTGCCTTACTTGA
TTACTGGAGTCAGACTTGTCTGAAACCAGTACACGATAGATTGTTTGACATTCTAAGGAA
TATCAAAACAGATCACACGTTTGATCATGGTAAAGCAATACAGTCTGTTAAGAACCTAAT
TCCAAAGGACCATTACCATAGCCTTGATTTATCAAGTGCTACAGATAGGTTTCCAATGGA

TCTTCAGCACAACTTAATGAAACTCCTTATAGGAGATCAGAAGGCTGATGCTTGAAGAAG
GATCTTAACAGAACACGAGTTTGAGGTCTTTCAAACGGGAGACAAGCTCCAATACGGAGC
AGGTCAACCAATGGGAGCCTACAGCTCGTGAGCTGTATTTACGGTTTGTCACCATATTGT
TATATGGTATTGTGCCGGTTCTACCGCGTACAATAACTATGCCATTATTGGTGATGACGT
CGTTATCGCAGATGATGATGTCGCTGATCGGTATGTAGAAGTGATCAAGGCCCTTGGGGT
TGATATATCACCTGCTAAATCTCACGTATCAAAAGATACATTCGAAATAGCAAAGCGATG
ATCAATCAAGGGGACCGAGGTAACACCTTTTCCGATAGATGGTTTATCTGTTATATACAA
TAAACCGGACATCCTTTACTTGTTCTTATTAGAGCAAGAAAAGAAAGGCTACATAGCAAA
GGGAGGGATCGAATCCGGGTCTTCCATAAGATCATTGCTGCAACTTTTCGGACTGCCGGA
GCGTAAAATACGTTCGGCAGCGAAACTTTACAGAATGATAGCCTTATTTCCTTTCATTGG
AAATAGAGAAGGAGATTATAAAGATCCAAAAGGATTTTATCATCTTCTTGGCTTCCCTGT
CTCTTGTAATATATCAAACGATATATACTTGAGAGAGCTTAAAGCTTACGCAGGTAAAGC
AATCTATGACCAAGTTGGAGGGAGAAAAGGTAAAATATGGAAGGCTGCCACTTATGGCTT
TTCGGTAGAGTTTCTATCGAATTGACTAGTGGTAGCTCCTGGATACGGGCCTCATGAATC
AAATCAAGAAGCATCATTATACCATCTTCCCCAAGTGGGGTCGATGTTATCACTAAGTCG
AAATCTGCAAGCAGAGTTCAACGAAGTAAGAATGATGGCCATTGACGAGAACTGAGACTA
TTTCTTAAACAGAAAAGTATCAATCCTTATCTCCGACCCAGTCATCGTATTTAGCGGTGA
CAGGTCTCTTGAAGTCTTAGGTACTCAGGCTCAAATCGTACGATGAGCGGTTGAAGGAAT
TAAATCCGGATACCAAACTCGTATCGATGAACTGAATGCCCCTTCCGAAGAGTCATTCCA
AGATGACACAGGAATAACAGATAGTGGGGTCATTATTCTT**TAA**AGAGTATTATCTCTCAG
GAATAACGCCCGCTATCTCCCTGTGCTTATTTCATA

>EnmuMV4-KVL-14-117
CCCTCCTGACGAAGGGAGATACTCGCTACACGCGAGTTATGACAAACTCACGGACGGTCC
AAAAGCCCAGGAGCCCACTGCTCTTGATTAGCGACTCCGTTTCAACGAACCTCTTCGATA
GAAGAGCTCCTAGTACTTATCAGTACGCTGAAAGGCAATCAGGAATGTCATACCTGATCT
GACGATGGAAGGCTAATCCCC**TAA**CCCATTGTTAAAATAGGAAAGGAACTTTTCTATGTT
CAATGAACAAGCGCGACCAAAACGCCAATATACTCG**ATT**AACAATTTAAAATTAAAACAA
CTTATCATCCAACTCAGAGATGTAACAGTCTGAGTGAATGACAGCTATCTTAATGTTCAA
AATGGTAACCTTTCTATCAGCGATTGATCTTGCCGATTAGAAAAG**ATG**GTCAAGGATAAC
GGTGTTGATCACACAATCAAACGAGTTAAACTCGCTAAATTATGTGTAACACGTTACCTA
GCCGGACAGCCTATTAGGATTACTGGGGCACCCGATGTTGGAGTACGTCGAAATGGCTTA
CCTAATTTAATAGGAAGACATCTTCGAGCACTCGCTATGTCAGGAACCGACAATGATATA
AGACTACTAATGACATTACTGTCATGAAATAGAGCTTTACCAGGGTCGGCTTATTACCCA
CCGTTGAGTACAATAACAGAGAAATCAAGAATGAAAGAGTCAGCTTTTGCTGAACTTTCT
TTCTTAATTCCAATGGTTATGCAATCAATGGGTTTAGTAGGATCCAATTGTCCAAAGTGA
TCAAAGTATCACCTAACGACTAAGGCCGGTCCTAATGCAGTTGCACTAGTCTCGAGTCTG
TTAGAAGCTAGAATCCTTCCGGATAAACTTATAGAAAATATAAGAATAGTCGGAGGGGAA
GATCTTTATAAGAATATTAATTTTATAAAATTTCTAAAACCTGAAAAGGTAGCAGAAAGA
TTTAAAATTAAGAACTTTAAAGATAAAATTCTAAGAATTAGGAAGTTATCGATTGTAAAC
GCCCCAGAGCGAAAATCGCGAGTAATCGCAATTTTAGACTACTGGAGTCAGTCTGCTCTA
AAACCCCTTCACGACCGAGTATTCTCGATCTTGAAAGGATTAGAAGGAGACTGTACGTTT
AGACAATCGGCACCTTCAAAATTCGTCTCTAACGGACCATACTTCTCATTAGATCTGTCA
GCAGCGACTGACCGCTTCCCCTTGAAAGTTCAAGAGTTAGTAGTTAGTCAGCTAACAGGT

AGTGAGAAATATGCCAAAGCCTGATCGTCCATTCTTGTTGATCAAGAATTCTGATGTCCT
TGAGAAAAGTGTAACGTTAAGTATAACGCGGGACAGCCTATGGGAGCGTATAGCTCCTGG
GCCATCTTCGCGCTAACTCATCATATCATAATAAGGGTAGCCGCAATGAAGGTAGGGAAA
TCCTTCTTCACGAACTATGCCGTATTAGGAGATGATGTCGTCATTGCTGACGAATCCGTT
GCAAATTCCTACAAGGACCTGATTCGTGATCTCGGAGTAGATATCTCTGATACCAAATCG
CATGTATCGAAAGATACATATGAGTTTGCTAAAAGATGATACCGAAACGGGGTCAACATT
TCAGGAGCACAAGTTAATGCATTCATCAGCTCTAGAAAATGATTTCTAGTAGCGAATGAA
TACAGAAATTTGTGTGGACTATGAGGGATTTCGGACTATGTAGCGGAACCCGGGGTTGTT
AAAGCCTTATTCACTGCCTTGAATCACAACAAGGGGATGATTCCCCGAATTGTGAAGAAA
GCAATGATGTTCTTGTCTCTTCCATGGGATAGACCTGACATGCCTAGGGACGAGCAAATT
TTACAATTTATTCGTTCCTCAGCACCTCAAGTGCTAGGATGTCACCAATTTGCAAAAACT
AGGGCCATGAATTTCTTCATGACCAGTTTAGCAGAGGTGAAATCCAGAATACTTGAACAG
GGGCTTCTCAAAGTTCGGGGGAGTGCACTTGAAACGATTAAATCGTTAAAGTACATTCAC
CAGGACTCTGAAGGTTCGGATGCCCAATCAATACTAAAGGCAGTTCCAGCACTGATGGTA
ACAGCTAATCAATATTCTCAGCTTGCTGAGGATATCGATGACTTGAGAGATCCCCTTGTA
ACGACCCCGGAGAAATTGATTTTCAACGAGGTCCGCTACTTAGGGTTCGACTCTAGTCGT
ATTAACGTTACCAGAAATGCTGAAATGCTAATGGCCACGAACGCTACATTGGTAAACCAA
TATAAACGCTGACTGAAGGAGTATTTCGAGCTTAGTCATAAAATACTAAGCGACGAATTA
CTGGACCAGCACACAGAGCGATCGTTTGCAAGGAAGTTATTCCGAACAAAAGTGATCGGA
TCTGTAATGCCGGGGTTCCCGTTATCAGGAGGAAAAGAATCCTCATCG**TAA**CTGAGATAG
CATTTTGCCATCCCCACTGCGAATGATATAGTGGTGTCATAGTCACTATATTGCCTGATT
AAATTCAGGGGGTTGCAGCGGGAGTATCTCCTCCAGTTTGG
>EnmuMV4-KVL-14-118
CCTGACGAAGGGAGATACTCGCTACACGCGAGTTATGACAAACTCACGGACGGTCCAAAA
GCCCAGGAGCCCACTGCTCTTGATTAGCGACTCCGTTTCAACGAACCTCTTCGATAGAAG
AGCTCCTAGTACTTATCAGTACGCTGAAAGGCAATCAGGAATGTCATACCTGATCTGACG
ATGGAAGGCTAATCCCC**TAA**CCCATTGTTAAAATAGGAAAGGAACTTTTCTATGTTCAAT
GAACAAGCGCGACCAAAACGCCAATATACTCG**ATT**AACAATTTAAAATTAAAACAACTTA
TCATCCAACTCAGAGATGTAACAGTCTGAGTGAATGACAGCTATCTTAATGTTCAAAATG
GTAACCTTTCTATCAGCGATTGATCTTGCCGATTAGAAAAG**ATG**GTCAAGGATAACGGTG
TTGATCACACAATTAAACGAGTTAAACTCGCTAAATTATGTGTAACACGTTACCTAGCCG
GACAGCCTATTAGGATTACTGGGGCACCCGATGTTGGAGTACGTCGAAATGGCTTACCTA
ATTTAATAGGAAGACATCTTCGAGCACTCGCTATGTCAGGAACCGACAATGATATAAGAC
TACTTATGACATTACTGTCATGAAATAGAGCTTTACCAGGGTCGGCTTATTACCCACCGT
TGAGTACAATAACAGAGAAATCAAGAATGAAAGAGTCAGCTTTTGCTGAACTTTCTTTCT
TAATTCCCATGGTTATGCAATCAATGGGTTTAGTAGGATCCAATTGTCCAAAGTGATCAA
AGTATCACCTAACGACTAAGGCCGGTCCTAATGCAGTTGCACTAGTCTCGAGTCTGTTAG
AAGCTAGAATCCTTCCGGATAAACTTATAGAAAATATAAGAATAGTCGGAGGGGAAGATC
TTTATAAGAATATTAATTTTATAAAATTTCTAAAACCTGAAAAGGTAGCAGAAAGATTTA
AAATTAAGAACTTTAAAGATAAAATTCTAAGAATTAGGAAGTTATCGATTGTAAACGCCC
CAGAGCGAAAATCGCGAGTAATCGCAATTTTAGACTACTGGAGTCAGTCTGCCCTAAAAC
CCCTTCATGACCGAGTATTCTCGATCCTGAAAGGATTAGAAGGAGACTGTACGTTTAGAC
AATCGGCGCCTTCAAAATTCGTTTCTAACGGACCATACTTCTCATTAGATCTGTCAGCAG
CGACTGACCGCTTCCCCTTGAAAGTTCAAGAGTTAGTAGTTAGTCAGCTAACAGGTAGTG

AGAAATATGCCAAAGCCTGATCGTCCATTCTTGTTGATCAAGAATTCTGATGTCCTTGAG
AAAAGTGTAACGTTAAGTATAACGCGGGACAGCCTATGGGAGCGTATAGCTCCTGGGCCA
TCTTCGCGCTAACTCATCATATCATAATAAGGGTAGCCGCAATGAAGGTAGGGAAATCCT
TCTTCACGAACTATGCCGTATTAGGAGATGATGTCGTCATCGCTGACGAATCCGTTGCAA
ATTCCTACAAGGACCTGATTCGTGATCTCGGAGTAGATATCTCTGATACCAAATCGCATG
TATCGAAAGATACATATGAGTTTGCTAAAAGATGATACCGAAACGGGGTCAACATTTCAG
GAGCACAAGTTAATGCATTCATCAGCTCTAGAAAATGATTTCTAGTAGCGAATGAATACA
GAAATTTGTGTGGACTATGAGGGATTTCGGACTATGTAGCGGAACCCGGGGTTGTTAAAG
CCTTATTCACTGCCTTGAATCACAACAAGGGGATGATTCCCCGAATTGTGAAGAAAGCAA
TGATGTTCTTGTCTCTTCCATGGGATAGACCTGACATGCCTAGGGACGAGCAAATTTTAC
AATTTATTCGTTCCTCAGCACCTCAAGTGCTAGGATGTCACCAATTTGCAAAAACTAGGG
CCATGAATTTCTTCATGACCAGTTTAGCAGAGGTGAAATCCAGAATACTTGAACAGGGGC
TTCTCAAAGTTCGGGGGAGTGCACTTGAAACGATTAAATCGTTAAAGTACATTCACCAGG
ACTCTGAAGGTTCGGATGCCCAATCAATACTAAAGGCAGTTCCAGCACTGATGGTAACAG
CTAATCAATATTCTCAGCTTGCTGAGGATATCGATGACTTGAGAGATCCCCTTGTAACGA
CCCCGGAGAAATTGATTTTCAACGAGGTCCGCTACTTAGGGTTCGACTCTAGTCGTATTA
ACGTTACCAGAAATGCTGAAATGCTAATGGCCACGAACGCTACATTGGTAAACCAATATA
AACGCTGACTGAAGGAGTATTTCGAGCTTAGTCATAAAATACTAAGCGACGAATTACTGG
ACCAGCACACAGAGCGATCGTTTGCAAGGAAATTATTCCGAACAAAAGTGATCGGATCTG
TAATGCCGGGGTTCCCGTTATCAGGAGGAAAAGAATCCTCATCG**TAA**CTGAGATAGCATT
TTGCCATCCCCACTGCGAATGATATAGTGGTGTCATAGTCACTATATTGCCTGATTAAAT
TCAGGGGGTTGCAGCGGGAGTATCTCC
>EnmuMV4-HHdFL130914-1
CTCCTGACGAAGGGAGATACTCGCTACACGCGAGTTATGACAAACTCACGGACGGTCCAA
AAGCCCAGGAGCCCACTGCTCTTGATTAGCGACTCCGTTTCAACGAACCTCTTCGATAGA
AGAGCTCCTAGTACTTATCAGTACGCTGAAAGGCAATCAGGAATGTCATACCTGATCTGA
CGATGGAAGGCTAATCCCC**TAA**CCCATTGTTAAAATAGGAAAGGAACTTTTCTATGTTCA
ATGAACAAGCGCGACCAAAACGCCAATATACTCG**ATT**AACAATTTAAAATTAAAACAACT
TATCATCCAGCTCAGAGATGTAACAGTCTGAGTGAATGACAGCTATCTTAATGTTCAAAA
TGGTAACCTTTCTATCAGCGATTGATCTTGCCGATTAGAAAAG**ATG**GTCAAGGATAACGG
TGTTGATCACACAATCAAACGAGTTAAACTCGCTAAATTATGTGTAACACGTTACCTAGC
CGGACAGCCTATTAGGATTACTGGGGCACCCGATGTTGGAGTACGTCGAAATGGCTTACC
TAATTTAATAGGAAGACATCTTCGAGCACTCGCTATGTCAGGAACCGACAATGATATAAG
ACTACTTATGACATTACTGTCATGAAATAGAGCTTTACCAGGGTCGGCTTATTACCCACC
GTTGAGTACAATAACAGAGAAATCAAGAATGAAAGAGTCAGCTTTCGCTGAACTTTCTTT
CTTAATTCCCATGGTTATGCAATCAATGGGTTTAGTAGGATCCAATTGTCCAAAGTGATC
AAAGTATCACCTAACGACTAAGGCCGGTCCTAATGCAGTTGCACTAGTCTCGAGTCTGTT
AGAAGCTAGAATCCTTCCGGATAAACTTATAGAAAATATAAGAATAGTCGGAGGGGAAGA
TCTTTATAAGAATATTAATTTTATAAAATTTCTAAAACCTGAAAAGGTAGCAGAAAGATT
TAAAATTAAGAACTTTAAAGATAAAATTCTAAGAATTAGGAAGTTATCGATTGTAAACGC
CCCAGAGCGAAAATCGCGAGTAATCGCAATTTTAGACTACTGGAGTCAGTCTGCCCTAAA
ACCCCTTCATGACCGAGTATTCTCGATCTTGAAAGGATTAGAAGGAGACTGTACGTTTAG
ACAATCGGCGCCTTCAAAATTCGTCTCTAACGGACCATACTTCTCATTAGATCTGTCAGC
AGCGACTGACCGCTTCCCCTTGAAAGTTCAAGAGTTAGTAGTTAGTCAGCTAACAGGTAG

TGAGAAATATGCCAAAGCCTGATCGTCCATTCTTGTTGATCAAGAATTCTGATGTCCTTG
AGAAAAGTGTAACGTTAAGTATAACGCGGGACAGCCTATGGGAGCGTATAGCTCCTGGGC
CATCTTCGCGCTAACTCATCATATCATAATAAGGGTAGCCGCAATGAAGGTAGGGAAATC
CTTCTTCACGAACTATGCCGTATTAGGAGATGATGTCGTCATTGCTGACGAATCCGTTGC
AAATTCCTACAAGGACCTGATTCGTGATCTCGGAGTAGATATCTCTGATACCAAATCGCA
TGTATCGAAAGATACATATGAGTTTGCTAAAAGATGATACCGAAACGGGGTCAACATTTC
AGGAGCACAAGTTAATGCATTCATCAGCTCTAGAAAATGATTTCTAGTAGCGAATGAATA
CAGAAATTTGTGTGGACTATGAGGGATTTCGGACTATGTAGCGGAACCCGGGGTTGTTAA
AGCCTTATTCACTGCCTTGAATCACAACAAGGGGATGATTCCCCGAATTGTGAAGAAAGC
AATGATGTTCTTGTCTCTTCCATGGGATAGACCTGACATGCCTAGGGACGAACAAATTTT
ACAATTTATTCGTTCCTCAGCACCTCAAGTGCTAGGATGTCACCAATTTGCAAAAACTAG
GGCCATGAATTTCTTCATGACCAGTTTAGCAGAGGTGAAATCCAGAATACTTGAACAGGG
GCTTCTCAAAGTTCGGGGGAGTGCACTTGAAACGATTAAATCGTTAAAGTACATTCACCA
GGACTCTGAAGGTTCGGATGCCCAATCAATACTAAAGGCAGTTCCAGCACTGATGGTAAC
AGCTAATCAATATTCTCAGCTTGCTGAGGATATCGATGACTTGAGAGATCCCCTTGTAAC
GACCCCGGAGAAATTGATTTTCAACGAGGTCCGCTACTTAGGGTTCGACTCTAGTCGTAT
TAACGTTACCAGAAATGCTGAAATGCTAATGGCCACGAACGCTACATTGGTAAACCAATA
TAAACGCTGACTGAAGGAGTATTTCGAGCTTAGTCATAAAATACTAAGCGACGAATTACT
GGACCAGCACACAGAGCGATCGTTTGCAAGGAAATTATTCCGAACAAAAGTGATCGGATC
TGTAATGCCGGGCTTCCCGTTATCAGGAGGAAAAGAATCCTCATCG**TAA**CTGAGATAGCA
TTTTGCCATCCCCACTGCGAATGATATAGTGGTGTCATAGTCACTATATTGCCTGATTAA
ATTCAGGGGGTTGCAGCGGGAGTATCTCCTCCAGTTTGGAG
>EnmuMV4-Berkeley
CCCTCCTGACGAAGGGAGATACTCGCTACACGCGAGTTATGACAAACTCACGGAAGGTCC
AAAAGCCCAGGAGTCCACTGCTCCTGATTAGCAACTCCGTTTCAACGAACCTCTTCGATA
GAAGAGCTCTTAGTACTAATCAGTACGCTGAAAGGCAATCAGGAATGTCATACCTGATCT
GACGATGGAAGGCTAATCCCCTAACCCATTGTTAAAATAGGAAAGGAGCTTTTCTATGTT
CAATGAACAAGCAGGACCAATACACCAATATAC**TAGATT**AACAATTTAAAATTAAAACAA
CTTATCATCCAGCTCAGAGATGTAACAGTCTGAGTAAATGACAGCTATCTTAATGTTCAA
AATGGTAACCTTTCTATCAGCGATTGATCTTGCCGATTAGAAAAG**ATG**GTCAAGGATAAC
GGTGTTGATCACACAATTAAACGAGTTAAACTCGCAAAATTATGTGTAACACGTTACCTA
GCCGGACAGCCTATTAGGATTACTGGGGCACCCGATGTTGGAGTGCGAAGAAATGGCTTA
CCTAATTTAATAGGAAGACATCTCCGAGCGCTCGCTATGTCAGGGACCGACAATGATATA
AGACTACTTCTGACATTACTGTCATGAAATAGAGCTTTACCAGGGTCGGCTTATTACCCA
CCGTTGAATACAATAACAGGGAAATCAGAAATGAAAGAATCAGCTTTCGCTGAACTTTCT
TTCCTAATCCCCATGGTTATGCAATCAATGGGTTTAGTAGGATCCAATTGTCCAAAGTGA
TCAAAGTATCACTTAACGACTAAGGCCGGTCCTAATGCTGTTGCACTAGTCTCGAGTCTA
TTAGAAGCTAGAATCCTTCCGGATAAACTTATAGAAAATATAAGGATAGTCGGAGGGGAA
GATCTTTATAAGAACATTAATTTCATAAAATTTCTAAAACCTGAAAAGGTTGCAGAAAGA
TTTAAAATTAAGAACTTTAAAGATAAAATTCTAAGAATTAGGAAGTTATCAATTGTAAAC
GCCCCAGAGCGTAAATCGCGAGTAATCGCGATTTTAGACTACTGGAGTCAGTCTGCTTTA
AAACCTCTTCATGATCGAGTTTTCTCGATACTGAAAGGATTAGAAGGAGACTGTACGTTT
AGACAATCAACACCTTCAAAATTCGTTTCTAACGGACCATATTTCTCATTAGATCTGTCA
GCAGCGACTGACCGCTTCCCCTTGAAAGTTCAAGAGTTAGTAGTTAGTCAACTGACAGGT

AGCGAGAAATATGCCAAAGCCTGATCGTCCATTCTTGTTGATCAAGAATTCTGATGTCCT
TGAGAAAAGTGTAACGTTAAGTATAATGCGGGGCAGCCTATGGGAGCATATAGCTCTTGG
GCCATCTTCGCGTTAACTCATCATATCATAATAAGAGTAGCCGCAATGAAGGTAGGGAAA
TCCTTCTTCACGAACTACGCTGTATTAGGAGATGATGTCGTCATTGCTGACGAATCCGTT
GCAAATTCCTACAAGGACCTGATTCGTGATCTCGGAGTAGATATATCTGATAACAAATCG
CACGTATCGAAAGATACATACGAATTTGCTAAAAGATGATATCGAAACGGGGTCAATATT
TCAGGAGCACAAGTTAATGCATTCATCAGCTCTAGGAAGTGATTCCTGGTGGCCAATGAG
TACAGAAACTTGTGTGGACTGTGAGGGATTTCGGACTATGTAGCGGAACCCGGGGTTGTT
AAAGCCTTATTCACCGCCTTAAATCACAATAAGGGGATGATTCCTCGAATTGTGAAGAAA
GCGATGATGTTCTTGTCTCTCCCGTGGGATAGACCTGACATGCCAAGGGATGAACAAATT
TTACAATTTATTCGTTCCTCAGCACCTCAAGTGCTAGGATGTCACCAATTTGCAAAAACT
AGAGCCATGAATTTCTTCATGACCAGTTTAGCAGAGGTGAAATCCAGAATACTTGAACAG
GGGCTTCTCAAAGTTCGGGGGAGTGCACTTGAAACGATTAAATCGCTAAAGTACATTCAC
CAAGACTCTGAAGGTTCGGATGCCCAATCGATACTAAAGGCAGTTCCAGCTTTAATGGTA
ACAGCTAATCAATATTCTCAGCTTGCTGAGGATATAGATGACTTGAGAGATCCCCTTGTG
ACGACTCCAGAGAAATTGATTTTCAATGAAGTACGTTACTTAGGTTTCGACTCTAGTCGT
ATTAACGTTACTAGAAATGCTGAAATGCTAATGGCCACGAACGCTACATTGGTAAACCAA
TATAAACGCTGATTGAAGGAGTATTTCGAACTTAGTCATAAAATACTAAGTGACGAGTTA
CTGGACCAGCACACAGAGCGATCATTTGCACGGAAGTTATTCCGAACAAAAGTGATTGGA
TCTGTAATGCCGGGGTTCCCGTTATCAGGAGGAAAAGAATCCTCATCG**TAA**CTGAGATAG
CACTTTGCCATCCCCACTGCGAAAGATATAGTGGTGTCATAATCACTATATTGCCTGATT
AAATTCAGGGGGTTGCAGCGGAGTATCTCCTCCAGTTTGGAG

>EnmuMV5-KVL-14-117
CGCCATCATGGCGCGAATAGCTAATAACTGCTACCCGTAGTAGACTCTCTTAGAGGATAT
TACTCCTTTAAGATTATCTATCTACTATAGCGTCCCTAGGTTTCGATGGCCCTCTGTCT
AGAAAGACTAGAGAAGGTCGAGACTGATTGGTAACCCGTTACAATAGTCCTCATCCTAGT
GGATGTAGGTCGTCGTAGACCAGCAGGAGAGAAGGCTAATCCCCTAAACTCTTCTCGCGA
TCAC**TAA**TGAGTCGTCACTATTGGAAGCCAATTCCTACCCACCATTATAAACATAAACAC
G**ATT**TTCAACAACCTATTTAACTTCTCCAGGTGTATAACCTGG**ATG**ATTAAATGGTTCTA
TCCAACCGTGCTCAGTCATAATGGGATTTCAGAGGATTCGTACCAACGATGTTTCGAGGC
GCAACTGCGCTGAGAAAAGCACCGAGGACGAATCTGATGCTCGGACCACTCGAAACTGGT
CAAGCTCTGTGTCTTAAGGTATCTCGAGGGAAGACCTCTCGAGGAACCTGCAGGACTTTC
GATGACTAAAGATAAATTACCTAAGGTTATACCTCAGGATATTCTCAATAGCATCCGATT
AAGGGACTTGAAAGTTATTTCAAGTATTCTATCAGTATTTTCACTGACAGATTCCTTAAA
AGGTGGAAAGCCTGTAGACATAAAGACTATCACAGCTCCGTGGTCAGGATCCATTCCTTC
CGATGTGGAGAAATGAATTCCTGATTTTCTGGACCGATGACATATCGAGCCATTTGATAC
TGACTGAGGTAAATATCATTTTACCCTCAGAGCAGGACCAAATGGACCCGCTCTTGCTGG
AAGTCTATTGGACTATCAAGCTCTAACAGAGCCTCTCAAAGAGAAGTTAAAACTTCTCGG
AGGAGAGTACTTTGCTATGAAACTTGATGAGTTAGAAGAGTGATACACTCAAACTAACAT
TCCAAAGTGCCTAATTAAGGTTTTCAAACTTAAAGATCCAAAAGGACCTATTAAGTTAGC
AAAACTCTCAGTTAGGGATGACAGTTCATGTAAATCACGAATATTTGGAATACTAGACTA
TTTTTCTCAAACAGTCTTGTATGTCATCCATTCGTGCCTGTTCGATATCCTAAGAAGGAT
GCCGACAGACATGACCTTTGATCAGACTAGGGGATTAACATCCCTTAGACCGGACAAAGG

GTCATCTTTCCACTCAATTGATCTAACAGCCGCTACAGACAGGTTTCCTGTAAAATTACA
GACTCTTGTCCTAGCTAAGCTGATAGGTGATGCGAAAGCAAAAGTTTGGGAATCTGTCCT
CATAGATAGAGACTACTACCTCAAAGGGAAACCATGGCGTTATGCCGTAGGTCAACCAAT
GGGAGCGTACTCATCATGAGCGACATTTGCCTTAACTCACCATCTAGTAGTATATTGTGC
TGCTAGACGGGCTGGAATCCTAAAATTCAGTAATTACCGAATTTTAGGGGACGATATTGT
GATCGGAAATGATCAGGTAGCCCACCATTATCGAGAGATAATGACCCATTTAGGGTGTGG
ATTTTCTGAGAAGAAAACTCACGTGAGCCCAGATTTCTTTGAATTTTCAAAGAGACTGGT
TTACCAAGGTCAAGAATTCTCTATCTTTCCATTGTCCGGTCTTATAGAAGTGGTGCCTAA
ATGGCACTTACTCTATGAGTTCCTAAAACAAGTAGAAAACAGAGGATTCGAAATCCAGAG
AAGTTATTGCAACCCCGGTCTAATTTCTGATCTCCTGGAATGTTTCGGAAAACCTCTCAG
ACTACGTCTGATGTACATTCGAAACATGACAGGGATGGCTGCACTTCCTATTGGAAGGAA
TGTAGATTATGAAACGGCAGGCGAGAGTGCACAAGTGCTCTCCCGGTTGTTTAATATCTC
ATTCTCATGTAACTTATCTCTCAAATCTTTGGGTAGAATTCTATCCAATCATGCGAGAGA
TGCGTACACATGATCGCAAGCCAGATCAGCTGAAAAGGTGATCACTAAAGCTGAAGAATG
ACAAGAGCGAACTCTTGCAATTCTTGAAGCAGATAGTGGTCCTCTTCTCAGTGCTTCTGA
CCAAGAAGCATTGTTGGATCAATGAGCAATGGTAGCTCCTCCTTTAGTGGTGTTCGCGCA
ACTTGCGTGGACGACCATTGAAGGGATAGAGCCAGACGTAGAGATGGGGGATCCAATTGA
ATACATATGAGATAAAACTCATTGTAAGAAATTGATTACCCTCCCCGAGTCCGAAGGAAT
CCTTCCTACTCGATCCAGTCATCAACACGCTGGGTCGCGTGCAGCTTACCTGAAATCTCT
AGTATCGGTGATCCACCGATATAGACTTGGGAAACGGCCTGGA**TAG**ACCGTTTAGTCCCC
TTTGCCCGCCTCTAGATAAAATACCAATAATAGGTTCAAATCTATTATTGGGGGATGACT
AGAGAGCTATTCGCGCCAAGATGGCGC
>EnmuMV5-KVL-14-118
GCGCGAATAGCTAATAACTGCTACCCGTAGTAGACTCTCTTAGAGGATATTACTCCTTTA
AGATTATCTATCTACTATAGCGTCCCTAGGTTTCGATGGGCCCTCTGTCTAGAAAGACTA
GAGAAGGTCGAGACTGATTGGTAACCCGTTACAATAGTCCTCATCCTAGTGGATGTAGGT
CGTCGTAGACCAGCAGGAGAGAAGGCTAATCCCCTAAACTCTTCTCGCGATCAC**TAA**TGA
GTCGTCACTATTGGAAGCCAATTCCTACCCACCATTATAAACATAAACACG**ATT**TTCAAC
AACCTATTTAACTTCTCCAGGTGTATAACCTGG**ATG**ATTAAATGGTTCTATCCAACCGTG
CTCAGTCATAATGGGATTTCAGAGGATTCGTACCAACGATGTTTCGAGGCGCAACTGCGC
TGAGAAAAGCACCGAGGACGAATCTGATGCTCGGACCACTCGAAACTGGTCAAGCTCTGT
GTCTTAAGGTATCTCGAGGGAAGACCTCTCGAGGAACCTGCAGGACTTTCGATGACTAAA
GATAAATTACCTAAGGTTATACCTCAGGATATTCTCAATAGCATCCGATTAAGGGACTTG
AAAGTTATTTCAAGTATTCTATCAGTATTTTCACTGACAGATTCCTTAAAAGGTGGAAAG
CCTGTAGACATAAAGACTATCACAGCTCCGTGGTCAGGATCCATTCCTTCCGATGTGGAG
AAATGAATTCCTGATTTTCTGGACCGATGACATATCGAGCCATTTGATACTGACTGAGGT
AAATATCATTTTACCCTCAGAGCAGGACCAAATGGACCCGCTCTTGCTGGAAGTCTATTG
GACTATCAAGCTTTAACAGAGCCTCTCAAAGAGAAGTTAAAACTTCTCGGAGGAGAGTAC
TTTGCTATGAAACTTGATGAGTTAGAAGAGTGATACACTCAAACTAACATTCCAAAGTGC
CTAATTAAGGTTTTCAAACTTAAGGATCCAAAAGGACCTATTAAGTTAGCAAAACTCTCA
GTTAGGGATGACAGTTCATGTAAATCACGAATATTTGGAATACTAGACTATTTTTCTCAA
ACAGTCTTGTATGTCATCCATTCGTGCCTGTTCGATATCCTTAGAAGGATGCCGACAGAC
ATGACCTTTGATCAGACTAAGGGATTAACATCCCTTAGACCGGACAAAGGGTCATCTTTC
CACTCAATTGATCTAACAGCCGCTACAGACAGGTTTCCTGTAAAATTACAGACTCTTGTC

CTAGCTAAGCTGATAGGTGATGCGAAAGCAAAAGTTTGGGAATCTGTCCTCATAGATAGA
GACTACTACCTCAAAGGGAAACCATGGCGTTATGCCGTAGGTCAACCAATGGGAGCGTAC
TCATCATGAGCGACATTTGCCTTAACTCACCATCTAGTAGTATATTGTGCTGCTAGACGG
GCTGGAATCCTAAAATTCAGTAATTACCGAATTTTAGGGGACGATATTGTGATCGGAAAT
GATCAGGTAGCCCACCATTATCGAGAGATAATGACCCATTTAGGGTGTGGATTTTCTGAG
AAGAAAACTCACGTGAGCCCAGATTTCTTTGAATTTTCAAAGAGACTGGTTTACCAAGGT
CAAGAATTCTCTATCTTTCCATTGTCCGGTCTTATAGAAGTGGTGCCTAAATGACACTTA
CTCTATGAGTTCCTAAAACAAGTAGAAAACAGAGGATTCGAAATCCAGAGAAGTTATTGC
AACCCCGGTCTAATTTCTGATCTCCTGGAATGTTTCGGAAAACCTCTCAGACTACGTCTG
ATGTACATTCGAAACATGACAGGGATGGCTGCACTTCCTATCGGAAGGAATGTAGATTAT
GAAACGGCAGGCGAGAGTGCACAAGTGCTCTCCCGGTTGTTTAATATCTCATTCTCATGT
AACTTATCTCTCAAATCTTTGGGTAGAATTCTATCCAATCATGCGAGAGATGCGTACACA
TGATCGCAAGCCAGATCAGCTGAAAAGGTGATCACTAAAGCTGAAGAATGACAAGAGCGA
ACTCTTGCAATTCTTGAAGCAGATAGTGGTCCTCTTCTCAGTGCTTCTGACCAAGAAGCA
TTGTTGGATCAATGAGCAATGGTAGCTCCTCCTTTAGTGGTGTTCGCGCAACTTGCGTGA
ACGACCATTGAAGGGATAGAGCCAGACGTAGAGATGGGGGATCCAATTGAATACATATGA
GATAAAACTCATTGTAAGAAATTGATTACCCTCCCCGAGTCCGAAGGAATCCTTCCTACT
CGATCCAGTCATCAACACGCTGGGTCGCGTGCAGCTTACCTGAAATCTCTAGTATCGGTG
ATCCACCGATATAGACTTGGGAAACGGCCTGGA**TAG**ACCGTTTAGTCCCCTTTGCCCGCC
TCTAGATAAAATACCAATAATAGGTTCAAATCTATTATTGGGGGATGACTAGA
>EnmuMV5-HHdFL130914-1
GCGCCATCATGGCGCGAATAGCTAATAACTGCTACCCGTAGTAGATTCTCTTAGAGGATA
TTACTCCTTTAAGATTATCCATCTACTATAGCGTCCCTAGGTTTCGATGGGCCCTCTGTC
TAGAAAGACTAGAGAAGGTCGAGACTGATTGGTAACCCGTTACAATAGTCCTCATCCTAG
TGGATGTAGGTCGTCGTAGACCAGCAGGAGAGAAGGCTAATCCCCTAAACTCTTCTCGCG
ATCAC**TAA**TGAGTCGTCACTATTGGAAGCCAATTCCTACCCACCATTATAAACATAAACA
CG**ATT**TTCAACAACCTATTTAACTTCTCCAGGTGTATAACCTGG**ATG**ATTAAATGGTTCT
ATCCAACCGTGCTCAGTCATAATGGGATTTCAGAGGATTCGTACCAACGATGTTTCGAGG
CGCAATTGCGCTGAGAAAAGCACCGAGGACGAATCTGATGCTCGGACCACTCGAAACTGG
TCAAGCTCTGTGTCTTAAGGTATCTCGAGGGAAGACCTCTCGAGGAACCTGCAGGACTTT
CGATGACTAAAGATAAATTACCTAAGGTTATACCTCAGGATATTCTCAATAGCATCCGAT
TAAGGGACTTGAAAGTTATTTCAAGTATTCTATCAGTGTTTTCACTGACAGATTCCTTAA
AAGGTGGAAAGCCTGTAGACATAAAGACTATCACAGCTCCGTGGTCAGGATCCATTCCTT
CCGATGTGGAGAAATGAATTCCTGATTTTCTGGACCGATGACATATCGAGCCATTTGATA
CTGACTGAGGTAAATATCATTTTACCCTCAGAGCAGGACCAAATGGACCCGCTCTTGCTG
GAAGTCTATTGGACTATCAAGCTTTAACAGAGCCTCTCAAAGAGAAGTTAAAACTTCTCG
GAGGAGAGTACTTTGCTATGAAACTTGATGAGTTAGAAGAGTGATACACTCAAACTAACA
TTCCAAAGTGCCTAATTAAGGTTTTCAAACTTAAGGATCCAAAAGGACCTATTAAGTTAG
CAAAACTCTCAGTTAGGGATGACAGTTCATGTAAATCACGAATATTTGGAATACTAGACT
ATTTTTCTCAAACAGTCTTGTATGTCATCCATTCGTGCCTGTTCGATATCCTAAGAAGGA
TGCCGACAGACATGACCTTTGATCAGACTAGGGGATTAACATCCCTTAGACCGGACAAAG
GGTCATCTTTCCACTCAATTGATCTAACAGCCGCTACAGACAGGTTTCCTGTAAAATTAC
AGACTCTTGTCCTAGCTAAGCTGATAGGTGATGCGAAAGCAAAAGTTTGGGAATCTGTCC
TCATAGATAGAGACTACTACCTCAAAGGGAAACCATGGCGTTATGCCGTAGGTCAACCAA

TGGGAGCGTACTCATCATGAGCGACATTTGCCTTAACTCACCATCTAGTAGTATATTGTG
CTGCTAGACGGGCTGGAATCCTAAAGTTCAGTAATTACCGAATTTTAGGGGACGATATTG
TGATCGGAAATGATCAGGTAGCCCACCATTATCGAGAGATAATGACCCATTTAGGGTGTG
GATTTTCTGAGAAGAAAACTCACGTGAGCCCAGATTTCTTTGAATTTTCAAAGAGACTGG
TTTACCAAGGTCAAGAATTCTCTATCTTTCCATTGTCCGGTCTTATAGAAGTGGTGCCTA
AATGGCACTTACTCTATGAGTTCCTAAAACAAGTAGAAAACAGAGGATTCGAAATCCAGA
GAAGTTATTGCAACCCCGGTCTAATTTCTGATCTCCTGGAATGTTTCGGAAAACCTCTCA
GACTACGTCTGATGTACATTCGAAACATGACAGGGATGGCTGCACTTCCTATCGGAAGGA
ATGTAGATTATGAAACGGCAGGCGAGAGTGCACAAGTGCTCTCCCGGTTGTTTAATATCT
CATTCTCATGTAACTTATCTCTCAAATCTTTGGGTAGAATTCTATCCAATCATGCGAGAG
ATGCGTACACATGATCGCAAGCCAGATCAGCTGAAAAGGTGATCACTAAAGCTGAAGAAT
GACAAGAGCGAACTCTTGCAATTCTTGAAGCGGATAGTGGTCCTCTTCTCAGTGCTTCTG
ACCAAGAAGCATTGTTGGATCAATGAGCAATGGTAGCTCCTCCTTTAGTGGTGTTCGCGC
AACTTGCGTGAACGACCATTGAAGGGATAGAGCCAGACGTAGAGATGGGGGATCCAATTG
AATACATATGAGATAAAACTCATTGTAAGAAATTGATTACCCTCCCCGAGTCCGAAGGAA
TCCTTCCTACTCGATCCAGTCATCAACACGCTGGGTCGCGTGCAGCTTACCTGAAATCTC
TAGTATCGGTGATCCACCGATATAGACTTGGGAAACGGCCTGGA**TAG**ACCGTTTAGTCCC
CTTTGCCCGCCTCTAGATAAAATACCAATAATAGGTTCAAATCTATTATTGGGGGATGAC
TAGAGAGCTATTCGCGCCAAGATGGCGC
>EnmuMV5-Berkeley
GCGCCATCATGGCGCGAATAGCTAATAACTGCTACCCGTAGTAGACTCTCTTAAGAGAAT
AAAATCTCTTAGGATTATCTATCTACTATAGCGTCCCTAGGTTTCGATGGGCCCTCTGTC
TAGAAAGACTAGAGAAGGTCGAGACTGGTTGGTAACCCGTTACAATAGTCCTCATCCTAG
TGGATGTAGGTCGTCGTAGACCAGCAGGAGAGAAGGCTAATCCCCTAACCTCTTCTCGCG
ATCAC**TAA**TGAGTCGTCACTATTGGAAACCAATTCCTACCCACCATTATATACATAAACA
CG**ATT**TTCAACAACTTATTTAACTTCTCCAGGTGTATAACCTGG**ATG**ATTAAATGGTTCT
ATCCAACCGTGCTTAGTCATAATGGGATTTCAGAGGATTCGTACCAGCGGTGTTTCGAAG
CGCAAATGCGCTGAGAAAAGCACCGGGGACGAATCTGATGCGCGGACCATTCGAAACTGG
TCAAGCTCTGTGTCTTACGGTATCTCGAGGGAAGACCTCTCGAGGAACCTGCAGGACTTT
CGATGACTAAAGATAAATTACCTAAGGTTATACCTCAGGATATTCTCGATAGCATCCGAT
TAAGGGACTTGAAAGTTATTTCAAGTATACTGTCAGTGTTTTCATTGACAGATTCCTTAA
AAGGTGGAAAGCCTGTAGACATAAAGACTATCACAGCTCCGTGGTCAGGGTCCATTCCTT
CCGATGTGGAGAAATGAATCCCTGATTTTCTGGACCGGTGGCATATCGAGCCATTTGATA
CTGACTGAGGTAAATATCATTTTACTCTCAGGGCAGGACCAAATGGACCCGCTCTTGCTG
GGAGTCTATTGGATTATCAAGCTCTAACAGAGCCTCTCAAAGAGAAATTAAAATTTCTCG
GAGGAGAGTACTTTGCTATGAAACTTGATGAGTTAGAAGAGTGATACACTCAAACTAACA
TTCCAAAATGCCTAATTAAAGTTTTCAAACTTAAAGATCCGAAAGGACCTATCAAGTTAG
CAAAACTTTCAGTTAGGGATGACAGTTCATGTAAATCACGAATATTTGGTATACTCGACT
ATTTTTCGCAAACAGTCTTGTATGTCATCCATTCGTGCCTGTTTGATATCCTGCGAAGGA
TGCCAACAGACATGACCTTTGACCAGACTAAAGGATTAAAATCCCTTAGACCGGATAAAG
GGTCATCATTCCACTCAATTGATCTAACAGCCGCTACAGATAGATTTCCTGTAAAATTAC
AGACTCTAGTCTTAGCTAAGCTGATAGGTGATGCGAAAGCAAAGGTTTGGGAATCTGTCC
TCATTGATAGAGATTACTACCTAAAAGGGAAACCTTGGCGTTATGCCGTTGGTCAACCTA
TGGGAGCATACTCATCATGAGCGACATTTGCCTTAACTCACCATCTAGTAGTATATTCTG

CTGCTAGACGGGCTGGAATCCTAAAATTTAGTAATTACCGAATTTTAGGGGACGATATTG
TGATCGGAAATGATCAGGTAGCCCACCATTATCGAGAGATAATGACCCAACTTGGGTGTG
GATTTTCAGAAAAGAAAACTCACGTGAGCCCAGATTTCTTTGAATTTTCAAAGAGACTGG
TATACCAAGGTCAAGAATTCTCTATCTTTCCATTGTCAGGACTAATAGAGGTAGTGCAGA
AATGACACTTACTCTACGAGTTCCTTAAACAAGTAGAAAACAGAGGATTCGAAATCCAGA
GAAGTTATTGCAACCCCGGTCTAATTTCTGATCTCCTGGAATGTTTTGGTAAACCTCTCA
GACTACGTCTGATGTACATCCGAAACATGACAGGGATGGCTGCACTTCCAATTGGAAGGA
ATGTAGATTACGAAACGGCAGGCGAGAGTGCACGAGTGCTCTCCCGGTTGTTCAACATCT
CATTCTCATGTAACTTATCTCTCAAATCATTGGGTAGAATTCTATCCAATCATGCGAGAG
ACGCGTATACATGATCGCAAGCCAGATCAGCTGAAAAGGTGATCACTAAAGCTGAAGAAT
GACAAGAGAGAACTCTTGCAATTCTTGAGACAGATAGTGGTCCACTTCTCAGTGCTTCTG
ACCAAGAAGCATTGTTGGACCAATGAGCAATGGTAGCTCCTCCTTTAGTGGTGTTCGCGC
AACTTGCGTGGACAACCATTGAAGGGATAGAGCCAGACGTAGAGATGGGAGATCCAATTG
AATACATATGAGATAAAACTCATTGTAAGAAATTGATTACTCTCCCTGAGTCTGAAGGAA
TACTTCCTACTCGATCCAGTCATCAACACGCTGGGTCGCGTGCAGCTTACCTGAAATCTC
TCGTATCGGTGATCCACCGATATAGACTAGGAAAACGGCCTGGA**TAG**ACCGTTTAGTCTC
CTTTGCCCGCCTCTAGATAAAATACCAATAATAGGTTCAAATCTATTATTGGGGGATGAC
TAGAGAGCTATTCGCGCCAAGATGGCGC

>EnmuMV6-KVL-14-117
CGCTGATGCGGGATAGCACTACAGCCCCTCTCTAACGAGATTATCTGTAGGCAA**TAA**GAG
TACAGGTTTTATACTGCCTGTACCAGTTCAGTGAGGGGTCACTTG**ATG**ATTCACGGTCTT
CCGTGATACCTGACGGTATCCAACTTACTACCCAAGTATAGCTATTTGCCTCTTAAACAA
ACAACTATC**ATG**GATAATAAAATAACAAACCTTTACTTGCCGCTCTTATTGAGCTGGATC
AAGAAGGTCTATTATCCGGATGTCGACGTGAGTCAGTCGGATCTTAACAGATTTGACTCC
ACAATCCAGAGATGGATTAAGGGGTCTGGTCTGGCGTGGACTGTCTCGAGATTGAAACAA
TCTCGGAACTTACTCACCAAACACATCTGTGAAGACCCGGATTTCCATAATCCGGGGATC
GCAGTGGGGAAGTCAAACTTACCCCGAGTATTACCTGGCTCACTAAGAGCTCAGATCTCC
CGAGGTGACCCAAGGGCCATCTCTTTCGGAATCACCCTACTGTCTGTAGGGAAGATGTCG
TTAGGAGGTACTGAACCAGATACATCTACCATCACTGATAAATGGTCTGGCCAGATACCA
AAGGAGCTCCAAGACGCTATTCCTGACTATTGCAAAGCAATTGGTCAGCTGAACACCGAT
TTTGAAGATTTCCATTGGACTCTTAAGAAGGGACCCGGAGGGGACACGGCAATACACTCC
GCAATGGAGGAATTCCGTATCTTACCTTCGAAACTTATCGACGCCCAGATAACTCTGGCC
GGCGGGAAGTTCAGGGATTGCACTGAATGACTTACGTCGAACATGACTAGAGAAACTCTA
GACAAGATCACGCAGTTGTGCGGTGCAAAACCAAAGTCCATATTACGGAGAATTGCTACA
ATTCCTGATAAGGAAATGAAAACAAGGGTTATAGCTAATCTGGATTATTGATCACAGACT
GCGCTAAAACCTCTCCATAATAGTCTTATGAGTATACTCAAGAGACTTCCAGCGGACATG
ACATATAAGCAAGATAAGGCTGCGGCCCACCTCCCCAAGGAGGGTCCATACTTCTCTTAC
GATTTGTCTGCCGCTACAGATAGGTTCCCGATAGAATTCCAATACCAAGTACTTTCCAGA
TTAATCGGGAAAGAAAAGGCTGATGCGTGAAAACACATCATGGTTGACTATCCCTTTAGA
TATAAAGGAAGAGACTACAAGTACGCCACGGGGCAACCCATGGGTGCTTATAGTTCTTGG
GCCCTATTTGCACTGTGCCATCACATCGTAGTTTATGCTAGTGCCAAGTCAGTCGGTAAA
CACCGATTCATTGGTTATGCTCTATTAGGTGATGACATCGTCATTGCTGACCGTGAGGTT
GCTGAAAAGTACCTCGCGATCATGACGGAGCTAGGGGTACAGATTTCTCCAACGAAATCA

CACGTTTCGGCTCACTCTTTTGAGTTCGCCAAACAGTGATATCATAAAGGGAAACAAGTA
TCCCCATTCCCTCTAGCCGGACTCCAAGAGGTAGGGTCAAAGTATCATCTACTTTTCGCC
TTCCTAGAGGACTTACATCATAAAGGGATAACCCCTTATGAAGATCCGTTTTCAAGGAAA
TACCTTTTCAGGCTGTTCCACATTATGGGTATAAAGGGAACAAGGCTCATGAATAATCTT
AGAAGGAAACTTCTGAGGTTATCCTATGTACCTTCTCCACATGACGATAAACCGACGGTA
GCCCTTAAGGCTAGATCGTTGGCTAAGCTTTGTGGTATCCCGTTATCCTGTAACTACTCC
GTAGATGCTTATTGCGATGCAATAGGCGACATGGGGATGAGAGCAGTATCAAACTCCTTA
GCTAAGGAGGTTGACCGAGCCATGGATGAAACCATGAAGTGATCCATGAAAGTCGAAGAC
TTAATCATGGAGCACGACCTGCCACTTTGCAGTGACGAGATGTTCGCACAGCTTCCTCTC
TTCCGAGCCCTTGACAAGATTGTCAAAGACTCAACAAGGGATGTGAAGAAGATCATGAGA
TCCGACATGCCTTTATCAAGGCATCTGGCAACCCCAAATATTGATATTTGAGGTTACTGT
CGGAAACTGACGATAACTCCCTATTCAGCTTCTGAGGCTCTCGCCCCAGTAAGGACAAAA
GAAATACAAGCCATTGGCTTCGTTTCCTTTGTGGATAGGCTCGTCGAGATCTGGGGTAGA
TCTCACTTG**TAA**CCAGATCTCTCAGGTTAGCAAGTTAAATACCCTGCGAGTTGGGGCGTC
CTCGAAGAGATATTAGACGTTCCGCGCCATCTTAAAACCCTTACTTTGTAAGGTGGGCTA
GATGTGCTATCCCGCATCAGCG
>EnmuMV6-KVL-14-118
CGCTGATGCGGGATAGCACTACAGCCCCTCTCTAACGAGATTATCTGTAGGCAA**TAA**GAG
TACAGGTTTTATACTGCCTGTACCAGTTCAGTGAGGGGTCACTCG**ATG**ATTCACGGTCTT
CCGTGATACCTGACGGTATCCAACTTACTACCCAAGTATAGCTATTTGCCTCTTAAACAA
ACAACTATC**ATG**GATAATAAAATAACAAACCTTTACTTGCCGCTCTTATTGAGCTGGATC
AAGAAGGTCTATTATCCGGATGTCGACGTGAGTCAGTCGGATCTTAACAGATTTGACTCC
ACAATCCAGAAATGGATTAAGGGGTCTGGTCTGGCGTGGACTGTCTCGAGATTGAAACAA
TCTCGGAACTTACTCACCAAACACATCTGTGAAGACCCGGATTTCCATAATCCGGGGATC
GCAGTGGGGAAGTCAAACTTACCCCGAGTATTACCTGGCTCACTAAGAGCTCAGATCTCC
CGAGGTGACCCAAGGGCCATCTCTTTCGGAATCACCCTACTGTCTGTAGGGAAGATGTCG
TTAGGAGGTACTGAACCAGATACATCTACCATCACTGATAAATGGTCTGGGCAGATACCA
AAGGAGCTCCAAGACGCTATTCCTGACTATTGCAAAGCAATTGGTCAGCTGAACACCGAT
TTTGAAGACTTCCATTGGACTCTTAAGAAGGGACCCGGAGGGGACACGGCAATACACTCC
GCAATGGAGGAATTCCGTATCTTACCTTCGAAACTTATCGACGCCCAGATAACTCTGGCC
GGCGGGAAGTTTAGGGATTGCACTGAATGACTTACGTCGAACATGACTAGAGAAACTCTA
GACAAGATCACGCAGTTGTGCGGTGCGAAACCCAAGTCCATATTACGGAGAATTGCTACA
ATTCCTGATAAGGAAATGAAAACAAGGGTTATAGCTAATCTGGATTATTGATCACAGACT
GCGCTAAAACCTCTCCATAATAGTCTTATGAGTATACTCAAGAGACTTCCAGCGGACATG
ACATATAAGCAAGATAAAGCCGCGGCCCACCTCCCCAAGGAGGGTCCGTACTTCTCTTAC
GATTTGTCTGCCGCTACAGATAGGTTCCCGATAGAATTCCAATACCAAGTACTTTCCAGA
TTAATCGGGAAAGAAAAGGCTGATGCGTGAAAACACATCATGGTTGACTATCCCTTTAGA
TATAAAGGAAGAGACTACAAGTACGCCACGGGGCAACCCATGGGTGCTTATAGTTCTTGG
GCCCTATTTGCACTGTGCCATCACATCGTAGTTTATGCTAGTGCCAAGTCAGTCGGTAAA
CACCGATTCATTGGTTATGCTCTATTAGGTGATGACATCGTCATTGCTGACCGCGAGGTT
GCTGAAAAGTACCTCGCGATCATGACGGAGCTAGGGGTACAGATCTCCCCAACGAAATCA
CACGTTTCGGCTCACTCTTTTGAGTTCGCCAAACAGTGATATCATAAAGGGAAACAAGTA
TCCCCATTCCCTCTAGCCGGACTCCAAGAGGTAGGGTCAAAGTATCATCTACTTTTCGCC
TTCCTAGAGGACTTACATCATAAAGGGATAACCCCTTATGAAGATCCGTTTTCAAGGAAA

TACCTTTTCAGGCTGTTCCACATTATGGGTATAAAGGGAACAAGGCTCATGAATAATCTT
AGAAGGAAACTTCTAAGGTTATCCTATGTACCTTCTCCACATGACGATAAACCGACGGTA
GCCCATAAGGCTAGATCGTTGGCTAAGCTTTGTGGTATCCCGTTATCCTGTAACTACTCC
GTAGATGCTTATTGCGATGCAATAGGCGACATGGGGATGAGAGCAGTATCAAACTCCTTA
GCTAAGGAGGTTGACCGAGCCATGGATGAAACCATGAAGTGATCCATGAAAGTCGAAGAC
TTAATCATGGAGCACGACCTGCCACTTTGCAGTGACGAGATGTTCGCACAGCTTCCTCTC
TTCCGAGCCCTTGACAAGATTGTCAAAGACTCAACAAGAGATGTGAAGAAGATCATGAGA
TCCGACATGCCTTTATCAAGGCATCTGGCAACCCCAAATATTGATATTTGAGGTTACTGT
CGGAAACTGACGATAACTCCCTATTCAGCTTCTGAGGCTCTCGCCCCAGTAAGGACAAAA
GAAATACAAGCCATTGGCTTCGTTTCCTTTGTGGATAGGCTCGTCGAGATCTGGGGTAGA
TCTCACTTG**TAA**CCAGATTTCTCAGGTTAGCAAGTCAAATACCCTGCGAGTTGGGGCGTC
CTCGAAGAGATATTAGACGTTCCGCGCCATCTTAAAACCCTTACTTTGTAAGGTGGGCTA
GATGTGCTATCCCGCATCAGC
>EnmuMV6-HHdFL130914-1
CGCTGATGCGGGATAGCACTACAGCCCCTCTCTAACGAGATTATCTGTAGGCAA**TAA**GAG
TACAGGTTTTATACTGCCTGTACCAGTTCAGTGAGGGGTCACTCG**ATG**ATTCACGGTCTT
CCGTGATACCTGACGGTATCCAACTTACTACCCAAGTATAGCTATTTGCCTCTTAAACAA
ACAACTATC**ATG**GATAATAAAATAACAAACCTTTACTTGCCGCTCTTATTGAGCTGGATC
AAGAAGGTCTATTATCCGGATGTCGACGTGAGTCAGTCGGATCTTAACAGATTTGACTCC
ACAATCCAGAGATGGATTAAGGGGTCTGGTCTGGCGTGGACTGTCTCGAGATTGAAACAA
TCTCGGAACTTACTCACCAAACACATCTGTGAAGACCCGGATTTCCATAATCCGGGGATC
GCAGTGGGGAAGTCAAACTTACCCCGAGTGTTACCTGGCTCACTAAGAGCTCAGATCTCC
CGAGGTGACCCAAGGGCCATCTCTTTCGGAATCACCCTACTGTCTGTAGGGAAGATGTCG
TTAGGAGGTACTGAACCAGATACATCTACCATCACTGATAAATGGTCTGGCCAGATACCA
AAGGAGCTCCAAGACGCTATTCCTGACTATTGCAAAGCAATTGGTCAGCTGAACACCGAT
TTTGAAGACTTCCATTGGACTCTTAAGAAGGACCCGGAGGGGACACGGCAATACACTCC
GCAATGGAGGAATTCCGTATCTTACCTTCGAAACTTATCGACGCCCAGATAACTCTGGCC
GGCGGGAAGTTTAGGGATTGCACTGAATGGCTTACGTCGAACATGACTAGAGAAACTCTA
GACAAGATCACGCAGTTATGCGGTGCAAAACCAAAGTCCATATTACGGAGAATTGCTACA
ATTCCTGATAAGGAAATGAAAACAAGGGTTATAGCTAATCTGGATTATTGATCACAGACT
GCGCTAAAACCTCTCCATAATAGTCTTATGAGTATACTCAAGAGACTTCCAGCGGACATG
ACATATAAGCAAGATAAGGCTGCGGCCCACCTCCCCAAGGAGGGTCCATACTTTTCTTAC
GATTTGTCTGCCGCTACAGATAGGTTCCCGATAGAATTCCAATACCAAGTACTTTCCAGA
TTAATCGGGAAAGAAAAGGCTGATGCGTGAAAACACATCATGGTTGACTATCCCTTTAGA
TATAAAGGAAGAGACTACAAGTACGCCACGGGGCAACCCATGGGTGCTTATAGTTCTTGG
GCCCTATTTGCACTGTGCCATCACATCGTAGTTTATGCTAGTGCCAAGTCTGTCGGTAAA
CACCGATTCATTGGTTATGCTCTATTAGGTGATGACATCGTCATTGCTGACCGTGAGGTT
GCTGAAAAGTACCTCGCGATCATGACGGAGCTAGGGGTACAGATCTCCCCAACGAAATCA
CACGTTTCGGCTCACTCTTTTGAGTTCGCCAAACAGTGATATCATAAAGGGAAACAAGTA
TCCCCATTCCCTCTAGCCGGACTCCAAGAGGTAGGGTCAAAGTATCATCTACTTTTCGCC
TTCCTAGAGGACTTACATCATAAAGGGATAACCCCTTATGAAGATCCGTTTTCAAGGAAA
TACCTTTTCAGGCTGTTCCACATTATGGGTATAAAGGGAACAAGGCTCATGAATAATCTT
AGAAGGAAACTCCTAAGGTTATCCTATGTACCTTCTCCACATGACGATAAACCGACGGTA
GCCCTTAAGGCTAAATCGTTGGCTAAGCTTTGTGGTATCCCGTTATCCTGTAACTACTCC

GTAGATGCTTATTGCGATGCAATAGGCGACATGGGGATGAGAGCAGTATCAAACTCCTTA
GCTAAGGAGGTTGACCGAGCCATGGATGAAACCATGAAGTGATCCATGAAAGTCGAAGAC
TTAATCATGGAGCACGACCTGCCACTTTGCAGTGACGAGATGTTCGCACAGCTTCCTCTC
TTCCGAGCCCTTGACAAGATTGTCAAAGACTCAACAAGAGATGTGAAGAAGATCATGAGA
TCCGACATGCCTTTATCAAGGCATCTGGCAACCCCAAATATTGATATTTGAGGTTACTGT
CGGAAACTGACGATAACTCCCTATTCAGCTTCTGAGGCTCTCGCCCCAGTAAGGACAAAA
GAAATACAAGCCATTGGCTTCGTTTCCTTTGTGGATAGGCTCGTCGAGATCTGGGGTAGA
TCTCACTTGTAACCAGATTTCTCAGGTTAGCAAGTTAAATACCCTGCGAGTTGGGGCGTC
CTCGAAGAGATATTAGACGTTCCGCGCCATCTTAAAACCCTTACTTTGTAAGGTGGGCTA
GATGTGCTATCCCGCATCAGCG
>EnmuMV6-Berkeley
CGCTGATGCGGGATAGCTCTACAGCCCCTCTCTAACGAGATTATCTGTAGGCAATAAGAG
TACAGGTTTTATACTGCCTGTACCAGTTCAGTGAGGGGTCACCAGATGATTCACGGTCTT
CCGTGATACCTGACGGTATCCGACTTACTACCCAAGTATAGCCATTTGCCTCTTAAACAA
ATAACTATCATGGATAATAAAATAACAAACCTTAACTTGCCGCTCTTATTGAGCTGGATC
AAGAAGGTCTATTATCCGGATGTCGACGTGAGTCAGTCGGATCTTAACAGATTTGACTCC
ACAATCCAGAGGTGGATTAAGGGGTCTGGTCTGGCGTGGACTGTCTCGAGATTGAAACAA
TCTCGAAACTTACTCACCAAACACATCTGTGAAGACCCGGATTTCCATAATCCGGGGATC
GCAGTGGGGAAGTCTAACTTACCCCGAGTGTTACCCGGCTCGTTGAGAGCTCAGATCTCC
AAAGGTGACCCTAGGGCCATCTCTTTCGGAATCACCCTACTTTCTGTAGGTAAGATGTCG
TTAGGAGGTACTGAACCAGATACATCTACCATCACTGATAAATGATCTGGCCGGATACCA
AAGGAGCTCCAAGACGCTATTCCTGACTATTGCACTGCAATTGGTCAGCTGAATACCGAT
TTTGAAGATTTCCATTGGACCCTTAAGAAAGGACCCGGAGGGGATACAGCGATTCACTCC
GCGATGGAGGAATTCCGTATCCTGTCTTCGGAACTGATCGAGGCACAGATTACTCTGGCC
GGCGATAAGTTTGAGGATTGCACAGAATGACTAACGTGAAACATGACTAGAGAAACTCTA
GACAAGATCACCCAGTTATGCGGTGCTAAACCTAAGTCCGTATTACGGAGAATTGCTACA
ATTCCTGATAAGGAAATGAAGACAAGGGTAATAGCCAATCTGGATTATTGATCCCAGACT
GCACTAAAACCACTCCATAATAGTCTTATGAGTATACTCAAGAGACTTCCAGCGGACATG
ACTTATAAGCAAGACAAGGCTGCGGATCACCTCCCTAAAGAGGGCCCGTACTTCTCTTAC
GATTTGTCTGCCGCTACAGATAGGTTCCCGATAGAATTCCAATACCAAGTACTTTCCAGA
TTAATCGGGAAAGAAAAGGCTGATGCGTGAAAACACATCATGGTGGACTATCCCTTTAGA
TATAAAGGAAGGGACTATAGGTACGCCACGGGACAACCCATGGGTGCGTATAGTTCTTGG
GCCCTATTTGCACTGTGTCATCACATCGTAGTCTACGCAAGTGCCAAATCGGTCGGCAAA
CGCCGATTCATTGGTTACGCTCTACTTGGTGATGACATCGTCATTGCCGATCGCGAGGTG
GCTGAAAAGTACCTCGAGATCATGACAGAGCTAGGGGTACAAATCTCCCCTACGAAATCA
CACGTTTCAGCTCACTCATTTGAGTTCGCTAAACAGTGATATCATAAAGGGAAACAGGTA
TCCCCATTCCCTATGGCCGGACTCCAAGAGGTAGGGTCAAAGTATCATCTACTTTTCGCC
TTCCTAGAGGACTTACATCACAAGGGTATAACCCCTTATGAAGATCCGTTTTCAAGGAAA
TACCTTTTCAGGTTGTTCCACATTATGGGTATAAAGGGAATAAGGCTTATGAATAATCTA
AGAAGGAAACTTCTCAGGTTATCTTATATACCTTCTCCGCATGACGATAAACCGACGATA
GCCATTAAGGCTAGATCGCTGGCTAAACTCTGTGGTATCCCGTTATCCTGTAACTACTCT
GTAGATGCTTATTGCGATGCAATAGGCGACATGGGGATGAGAGCAGTATCAAACTCCTTA
GCTAAGGAGGTTGACCGAGCCATGGATGAAACCATGAAATGGACCATGAAAGTCGAAGAC
TTAATCATGGAACATGATCTGCCACTTTGCAGTGACGAGATGTTCGCACAGCTTCCCCTC

TTCAGAGCTCTTGACAAAATTGTCAAAGACTCTACAAGAGATGTGAAGAAGATCATGAGA
TCCGATATGCCTTTAACAAGGCACCTGGCAACCCCAAATATTGATATTTGAGGTTACTGT
CGGAAACTGACAATAACTCCCTATTCAGCTTCTGAGGCTCTCGCCCCAGTAAGGACAAAA
GAAATTCAAGCCATTGGCTTTGTTTCGTTTGTGGATAGGCTCGTCGAGATCTGGGGTAGA
TCTCACTTG**TAA**CCAGATTTCTCGGGTTAGCAAGTAAAATACCCTGCGAGTTGGGGCGTC
CTCGAAGAGATATTAGACGTTCCGCGCCATCTTAAAACCCTTACTCTGTAAGGTGGGCTA
GATGTGCTATCCCGCATCAGCG

>EnmuMV7-KVL-14-117
CGGGGGGCAACACTTACTTTCGTAAGTCACCTTGTGTGAGTGCCCAACTTTACTTTCTCC
ACCCTAAGAAACTAAAATCACACACCGATCCCCAAAGGGGAAGGATATCGGTTAAAATAT
CCCCCACCATTAAAATAAACTTAAAC**ATG**AAAATAACAAAATTTAACATATTTAAGGTTA
CTCTAAAGGTGGTAACCTGGATATTTCAAGAGTTCAATCTTAAAATATCTGGCCGGATGA
GTGATTATATAGACCTAATCCTGAGACTCGATCGATTATTATATAATCGAGGAGCGCTAG
AAACCCTTAAATATATTAAGGGTACTAGAACTGCCTATTTGGCTTACTTATCAGGTAAGC
CAATACGAGTTAAGGGAGTAAGGACAACGAAGGATGGGATCCCTGTTATCCTAGGTGACT
TTGTTCCCAAAATCCGTAAAGGACCAACTCCAGCTATGCTGCAATTGATCAATACGATTT
TGTTCTGTACAAGGGCACTGAAGCTTGGAAGAACTCCTGATTTCTCACCTATTATTAATC
CCCCCAAAAGGGATCCGATTAATATAGGAGAATTCGTGGACGACTTCTGGCGGGATATGG
GATACCACCGACAGGCTCGTACGAATCCTAAGAGTCTTGACTTCCGTAAGTTTCACTTAA
CGACAAAATCTGGCCCGAATGGGACAGCTAATGCGTTGTGAACCTCTCTCAATGACCTTA
AGGCGTTGTCTGAACAACAACTAACCGATATCGGTATCATTGGAGGAGAGAACTTACGGA
ACAAGATAAGTACTCTCAAAGAGGGACTTTCTAAGATTCCTGAGTTATCCTACTTTATAG
ATACCTCTGCAGGTACCTATACTAGAAGGTTAACCAGTTTCCCTGATAAAGAACTAAAAG
TTCGAGTAATCGCCGTTGGCGACTACTTTAGTCAAGCAGCGTTAAAACCGCTGCATGATT
ATTTATTCAGGGTTCTTAAGAAAATTCCTCAAGATTGTACTTTCAACCAAGGTGGGTTCT
GAGATAAAATTAAAGATCAGGAATACTATGCAAGTATCGACTTAACAGCCGCTACTGATA
GATTCCCTATCTCAACAATATCTCAGGTGCTACTAGGGAGACTCCCAGAATCTTACGTGA
ATGCATGGAGCCGACTTATGGTCGGTACCCCATTTATTCATGAAGGACAGGAAATAAATT
ACTCTGTCGGAAACCCTATGGGTTTCTACTCTTCATGAGCATCCTTTGCAGTAGCACACC
ACTATGTGGTATACTATTGCTGTAAGAAACTAGGTATCCCTTGGAAAACTTTAAAATATT
GTCTCCTTGGAGATGATATTGTTATATGTGATCCAAAAGTAGCAGCCCTGTACAAGGAAA
CTATTTCAGGGTTGGGTGTTGAATTCTCAGAAGAGAAAACAATCACCTCACCACACTTAG
TAGAGTTTGCCAAAAGGCTAATCTACAAAGGTACTGAAATATCACCTTTCCCAATCTCAG
CTTTAGGTGAGTTAGCTAGTAAATATTATTTACTAACTAACTTATTCTTAGAGCTTGAGA
AGAAAGGTTGGGTTTCACTTGGCGGTGCGCCATCAGCAGTTGAATCTTTCCACAAGATCG
TTCTTAACTGATCTACCGTGCGAGCACGAGAGATCGAAGTTAAGGCATCTATTTGCGAAC
GGGTTATGAAGATAGCCCGAGGGGCCGAGAATGCTGGGACTCTCTTAACAGAGTGTTTCA
GGAAACTCGGATACCCATTTGTTCTTAGCGACTTTGTTGCCAGAAATGTTCTGGAAAACA
TAGTTGTTGAGAACTTCGCAAAATCTAATCCTGCTTCTCATGACCCAGAAAGGGACAAGA
AGAAGAAAAGACCATGTGTGGGTCTCGGCATACTTGCTGAGAACCTTACATGTCTTTTAA
CAGGTTTAGATGAGGAAAGATTTTCGCTAGGATTCGACTTAATATCGACATTACCTCACT
TAAATGCCTATGGGCAGATAAGTGAAATGTATATCGAGATTAATAAGAAAGCCCGAAAGA
TTAGCACCACAAATGGTGCGGAATGACCATTGTTATTTAAAACAATGTGTATTCCATGGG

ACGATACCATCTTTACGATGAGATCATCTCATTTAATCGCTAAGGGCTCTTCTAGAGTCG
TCAAAGATCTTCAGAATAGGGCAGAGCTGTTAAGCTTCTACCCTCCTGAAGAGCTCCTTC
GTGAGGATCCTAATTTCTTAAAATCACTCTAAAGCCTAGCTCGGATTCGGGTCAGCGCTG
ATAAGCTGATTCCGCTCGAG
>EnmuMV7-KVL-14-118
CCCGTAGTTAATTCTACGGGGGGCAACACTTACTTTCGTAAGTCACCTTGTGTGAGTGCC
CAACTTTACTTTCTCCACCCTAAGAAACTAAAATCACACACCGATCCCCAAAGGGGAAGG
ATATCGGTTAAAATATCCCCCACCATTAAAATAAACTTAAACATGAAAATAACAAAATTT
AACATATTTAAGGTTACTCTAAAGGTGGTAACCTGGATATTTCAAGAGTTCAATCTTAAA
ATATCTGGCCGGATGAGTGATTATATAGACCTAATCCTGAGACTCGATCGATTATTATAT
AATCGAGGAGCGCTAGAGACCCTTAAATATATTAAGGGTACTAGAACTGCCTATTTGGCT
TACTTATCAGGTAAGCCAATACGAGTTAAGGGAGTAAGGACAACGAAGGATGGGATCCCT
GTTATCCTAGGTGACTTTGTTCCCAAAATCCGTAAAGGACCAACTCCAGCTATGCTGCAA
TTGATCAATACGATTTTGTTCTGTACAAGGGCACTAAAGCTTGGAAGAACTCCTGATTTC
TCACCTATTATTAATCCCCCCAAAAGGGATCCGATTAATATAGGAGAATTCGTGGACGAC
TTCTGGCGGGATATGGATACCACCGACAGGCTCGTACGAATCCTAAGAGTCTTGACTTC
CGTAAGTTTCACTTAACGACAAAATCTGGCCCGAACGGGACAGCTAATGCGTTGTGAACC
TCTCTCAATGACCTTAAGGCGTTGTCTGAACAACAACTAACCGATATCGGTATCATTGGA
GGAGAGAACTTACGGAACAAGATAAGTACTCTCAAAGAGGGACTTTCTAAGATTCCTGAG
TTAGCCTACTTTATAGATACCTCTGCAGGTACCTATACTAGAAGGTTAACCAGTTTCCCT
GATAAAGAACTAAAAGTTCGAGTAATCGCCGTTGGCGACTACTTTAGTCAGGCAGCATTA
AAACCGCTGCATGATTATTTATTCAGGGTTCTTAAGAAAATTCCTCAAGATTGTACTTTC
AACCAAGGTGGGTTCTGAGATAAAATTAAAGATCAGGAATACTATGCAAGTATCGACTTA
ACAGCCGCTACTGATAGATTCCCTATCTCAACAATATCTCAGGTGCTACTAGGGAGACTC
CCGGAATCTTACGTGAATGCATGGAGCCGACTTATGGTCGGTACCCCATTTATTCATGAA
GGACAGGAAATAAATTACTCTGTCGGAAACCCTATGGGTTTCTACTCTTCATGAGCATCC
TTTGCAGTAGCACACCACTATGTGGTATACTATTGCTGTAAGAAACTGGGTATCCCTTGG
AAAACTTTAAAATATTGTCTCCTTGGAGACGATATTGTTATATGTGATCCAAAAGTAGCA
GCCCTGTACAAGGAAACTATTTCAGGGTTGGGTGTTGAATTCTCAGAAGAGAAAACAATC
ACCTCACCACACTTAGTAGAGTTCGCCAAAAGGCTAATCTACAAAGGTACTGAAATATCA
CCTTTCCCAATCTCAGCTTTAGGTGAGCTAGCTAGTAAATATTATTTACTAACTAACTTA
TTCTTAGAGCTTGAGAAGAAAGGTTGGGTTTCACTTGGCGGTGCGCCATCAGCAGTTGAA
TCTTTCCACAAGATCGTTCTTAACTGATCTACCGTGCGAGCACGAGAGATCGAAGTTAAG
GCATCTATTTGCGAACGGGTTATGAAGATAGCCCGAGGGGCCGAGAATGCTGGGACTCTC
TTAACAGAGTGTTTCAGGAAACTCGGATACCCATTTGTTCTTAGTGACTTTGTTGCCAGA
AATGTTCTGGAAAACATAGTCGTTGAGAACTTCGCAAAATCTAATCCTGCTTCTCATGAC
CCAGAAAGGGACAAGAAGAAGAAAAGACCATGTGTGGGTCTCGGCATACTTGCTGAGAAC
CTTACATGTCTTTTAACAGGTTTAGATGAGGAAAGATTTTCGCTAGGATTCGACTTAATA
TCGACATTACCTCACTTAAATGCCTATGGGCAGATAAGTGAAATGTATATCGAGATTAAT
AAGAAAGCCCGAAAGATTAGCACCACAAATGGTGCGGAATGACCATTGTTATTTAAAACA
ATGTGTATTCCATGAGACGATACCATCTTTACGATGAGATCATCTCATTTAATCGCTAAG
GGCTCTTCTAGAGTCGTCAAAGATCTTCAGAATAGGGCAGAGCTGTTAAGCTTCTACCCT
CCTGAAGAGCTCCTTCGTGAGGATCCTAATTTCTTAAAATCACTCTAAAGCCTAGCTCGG
ATTCGGGTCAGCGCTGATAAGCTGATTCCGCTCGAGACCCGTGTCGAAGTCCTTTCTTGC

GAAAGGCGGGGACACGGGTGTATACCCC
>EnmuMV7-HHdFL130914-1
CCCGTAGTTAATTCTACGGGGGGCAACACTTACTTTCGTAAGTCACCTTGTGTGAGTGCC
CAACTTTACTTTCTCCACCCTAAGAAACTAAAATCACACACCGATCCCCAAAGGGGAAGG
ATATCGGTTAAAATATCCCCCACCATTAAAATAAACTTAAAC**ATG**AAAATAACAAAATTT
AACATATTTAAGGTTACTCTAAAGGTGGTAACCTGGATATTTCAAGAGTTCAATCTTAAA
ATATCTGGCCGGATGAGTGATTATATAGACCTAATCCTGAGACTCGATCGATTATTATAT
AATCGAGGAGCGCTAGAAACCCTTAAATATATTAAGGGTACTAGAACTGCCTATTTGGCT
TACTTATCAGGTAAGCCAATACGAGTTAAGGGAGTAAGGACAACGAAGGATGGGATCCCT
GTTATCCTAGGTGACTTTGTTCCCAAAATCCGTAAAGGACCAACTCCAGCTATGCTGCAA
TTGATCAATACGATTTTGTTCTGTACAAGGGCACTGAAGCTTGGAAGAACTCCTGATTTC
TCACCTATTATTAATCCCCCCAAAAGGGATCCGATTAATATAGGAGAATTCGTGGACGAC
TTCTGGCGGGATATGGATACCACCGACAGGCTCGTACGAATCCTAAGAGTCTTGACTTC
CGTAAGTTTCACTTAACGACAAAATCTGGCCCGAATGGGACAGCTAATGCGTTGTGAACC
TCTCTCAATGACCTTAAGGCGTTGTCTGAACAACAACTAACCGATATCGGTATCATTGGA
GGAGAGAACTTACGGAACAAGATAAGTACTCTCAAAGAGGGACTTTCTAAGATTCCTGAG
TTATCCTACTTTATAGATACCTCTGCAGGTACCTATACTAGAAGGTTAACCAGTTTCCCT
GATAAAGAACTAAAAGTTCGAGTAATCGCCGTTGGCGATTACTTTAGTCAGGCAGCGTTA
AAACCGCTGCATGATTATTTATTCAGGGTTCTTAAGAAAATTCCTCAAGATTGTACTTTC
AACCAAGGTGGGTTCTGAGATAAAATTAAAGATCAGGAATACTATGCAAGTATCGACTTA
ACAGCCGCTACTGATAGATTCCCTATCTCAACAATATCTCAGGTGCTACTAGGGAGACTC
CCGGAATCTTACGTGAATGCATGGAGCCGACTTATGGTCGGTACCCCATTTATTCATGAA
GGACAGGAAATAAATTACTCTGTCGGAAACCCTATGGGTTTCTACTCTTCATGAGCATCC
TTTGCAGTAGCACACCACTATGTGGTATACTATTGCTGTAAGAAACTGGGTATCCCTTGG
AAAACTTTAAAATATTGTCTCCTTGGAGATGATATTGTTATATGTGATCCAAAAGTAGCA
GCCCTGTACAAGGAAACTATTTCAGGGTTGGGTGTTGAGTTCTCAGAAGAGAAAACAATC
ACCTCACCACACTTAGTAGAATTCGCCAAAAGGCTAATCTACAAAGGTACTGAAATATCA
CCTTTCCCAATCTCAGCTTTAGGTGAGCTAGCTAGTAAATATTATTTACTAACTAACTTA
TTCTTAGAGCTTGAGAAGAAAGGTTGGGTTTCACTTGGCGGTGCGCCATCAGCAGTTGAA
TCTTTCCACAAGATCGTTCTTAACTGATCTACCGTGCGAGCACGAGAGATTGAAGTTAAG
GCATCTATTTGCGAACGGGTTATGAAGATAGCCCGAGGGGCCGAGAATGCTGGGACTCTC
TTAACAGAGTGTTTCAGGAAACTCGGATACCCATTTGTTCTTAGCGACTTTGTTGCCAGA
AATGTTCTGGAAAACATAGTTGTTGAGAACTTCGCAAAATCTAATCCTGCTTCTCATGAC
CCAGAAAGGGACAAGAAGAAGAAAAGACCATGTGTGGGTCTCGGCATACTTGCTGAGAAC
CTTACATGTCTTTTAACAGGTTTAGATGAGGAAAGATTTTCGCTAGGATTCGACTTAATA
TCGACATTACCTCACTTAAATGCCTATGGGCAGATAAGTGAAATGTATATCGAGATTAAT
AAGAAAGCCCGAAAGATTAGCACCACAAATGGTGCGGAATGACCATTGTTATTTAAAACA
ATGTGTATTCCATGAGACGATACCATCTTTACGATGAGATCATCTCATTTAATCGCTAAG
GGCTCTTCTAGAGTCGTCAAAGATCTTCAGAATAGGGCAGAGCTGTTAAGCTTCTACCCT
CCTGAAGAGCTCCTTCGTGAGGATCCTAATTTCTTAAAATCACTC**TAA**AGCCTAGCTCGG
ATTCGGGTCAGCGCTGATAAGCTGATTCCGCTCGAGACCCGTGTCGAAGTCCTTTCTTGC
GAAAGGCGGGGACACGGGTGTATACCCCCCTAGGAATTAACCTA
>EnmuMV7-Berkeley
CCCGTAGTTAATTCTACGGGGGGCAACACTTACTTTCGTAAGTCACCTTGTGTGAGTGCC

CAACCTTTACTTCCTTCACCCTAAGGAACTAAAATCACACACCGATCCCCAGAGGGGAAG
GATATCGGT**TAA**AATATCCCCCACCATAAAATAAACTTAAAC**ATG**AAAATAACAAAATTT
AACATATTTAAGGTTACTCTAAAGGTGGTAACCTGGATATTTCAAGAGTTCAATCTTAAA
ATATCTGGCCGGATGAGTGATTACATAGACCTAATTCTGAGACTCGATCGATTATTGTAT
AATCGAGGAGCGCTAGAAACCTTAAAATATATTAAGGGAACTAGAACTGCCTACTTGGCT
TACTTATCAGGTAAGCCGGTACGAGTTAAGGGAGTAAGGACAACGAAGGATGGGATCCCT
GTTATCCTAGGTGACTTTGTTCCCAAAATCCGTAAAGGACCAACTCCAGCTATGCTGCAA
TTGATCAATACGATTTTGTTCTGTACAAGGGCACTAAAGCTTGGAAGAACTCCTGATTTC
TCACCTATTATTAATCCCCCCAAAAGGGATCCGATTAATATAGGAGAATTCGTGGACGAC
TTCTGGCGGGATATGGGATACCACCGACAGGCTCGTACGAATCCTAAGAGTCTTGATTTC
CGTAAGTTTCACTTGACGACAAAATCTGGCCCTAACGGGACAGCTAATGCGCTGTGAACC
TCTCTCAATGACCTTAAGGCGTTGTCTGAACAACAACTAACCGATATCGGTATCATTGGA
GGAGAGAACTTACGGAACAAGATAAGTGCTCTCAAAGAGGGACTTTCTAAGATTCCTGAG
TTATCCTACTTTATAGATACCTCTCCAGGTATCTATACTAGAAGAATAACCAGTTTCCCT
GATAAAGAACTAAAAGTTCGGGTAATCGCCGTTGGCGATTACTTTAGTCAAGCAGCATTA
AAACCGCTGCATGATTATTTATTTAGGGTTCTTAAGAAAATTCCTCAAGATTGTACTTTC
AACCAAGGTGCTTTCTGAGATAAAATTAAAGATCAGGAATACTATGCTAGTATCGACTTA
ACAGCCGCTACTGATAGATTCCCTATCTCAACAATATCTCAGGTGCTACTAGGGAGACTC
CCAGAATCTTACGTAAATGCCTGGAGCCGACTTATGGTCGGGACTCCATTTACTTATGAA
GGACAGGAAATAAATTACGCTGTCGGAAACCCTATGGGTTTCTACTCTTCATGAGCATCA
TTTGCAGTAGCTCACCACTATGTGGTTTACTATTGCTGTAAGAAACTAGGTATCCCTTGG
AAAACATTAAAATATTGTCTCTTAGGAGATGATATTGTTATATGTGATCCAAAAGTAGCA
GCCCTGTACAAGGAAACAATTTCAGGGTTAGGTGTTGAATTCTCAGAAGAGAAGACAATT
ACCTCACCACACCTTGTAGAATTCGCCAAAAGGCTCATCTACAAAGGTACTGAAATATCA
CCTTTCCCAATCTCAGCTTTAGGTGAGTTAGCTAGTAAATATTATTTACTAACTAACTTA
TTCTTAGAGCTTGAGAAGAAAGGTTGAGTTTCACTTGGCGGTGCGCCATCAGCAGTTGAA
TCTTTCCATAAGATTGTTCTTAACTGATCAACCGTGCGAGCACGGGAGATTGAAGTTAAG
GCATCTATTTGCGAACGGGTTATGAAAATAGCCCGAGGGGCCGAGAATGCTGGTACTCTT
TTAACAGAGTGTTTCAGGAAACTCGGATACCCATTTGTTCTAAGTGACTTTGTTGCCAGA
AATGTTCTGGAAAACATCGTCGTAGAGAACTTCGCAAAATCTAATCCTGCTTCTCATGAC
CCCGAAAGGGATAAGAAGAAGAAAAGACCATGCGTGGGTCTCGGCATACTTGCTGAGAAC
CTCACATGTCTTTTATCAGGTTTAGATGAGGAAAGATTTTCTCTAGGATTCGACTTAATC
TCGACATTACCTCACTTAAATGCCTATGGGCAAATAAGTGAAATGTATATCGAGATTAAT
AAGAAAGCCCGAAAGATTAGCACCACAAATGGTGCGGAATGGCCATTGTTATTTAAAACA
ATGTGTATTCCATGGGACGATACCATCTTCACGATGAGATCATCTCATTTAATCGCTAAG
GGCTCTTCTAGAGTCGTCAAAGATCTTCAGAATAGGGCAGAGCTATTAAGCTTCTACCCT
CCTGAAGAGCTCCTTCGAGAGGATCCTAATTTCTTAAAATCACTC**TAA**AGCCTAGCTCGG
ATTCGGGTCAGCGCTGATAAGCTGATTCCGCTCGAGACCCGTGTCAAAGTCCTTTCTCTC
GAAAGGCGGGGGACACGGGTGTATACCCCCCTAGGAATTAACCTA

>EnmuMV8-Berkeley
GGCGATCTAATCGATCGCCATACTTTATCTCTGGACTCAGGTTCTTTCCTGGAACCTAAA
TGGAGATAAGGATGCTTATAAGCCCGGAATGGGCTATAAGACATCGACCTTTGGGTCGGT
GAGAGGGAGTAGGAGCGATCCTACAGCACCATGGGCGTCATTCTGTTTGCTAATTAGGTA

CTCTAAAAGGTGGAATTCCTTTTACAATGCGGAAGGCTAATACCCTAACCCGTTGGACAA
CGAGTTAAGACACTTAGCTGCGGACCACTGCCC**TAA**AACCCCCCGCAAATCAAAAT**ATT**
ACTAATAAAATAAAACAGTTTCTTGTCATTCTAAAATGACTAAGAGAATGTAGAATTTTA
AGAGTAGATTCTGACTTGGGGAAGGCATTAGATGAGCACTTTAGCATTATCATAGCGCGA
TGGATATCCAGTAAAGGACAACTAGAGACAATCAATAGACTTAAAAATCTACGGGTT**ATG**
CTCTATGCTTTCCTGTCTGGACGTCCTGTTCGGATGGTTGGGTATTCTCAGTATAAAAAT
GGATTACCTAGAGCTTTTGCTCCAGCTTATTCACTTTTATGTGAGAAAGACCCTCACACA
GTTAGATACATCTTATCACTTTTACAAGTGAGCAGATGCATCCCTGCGTGGAAGAAACCA
GACCTTAGGACAATAACAGATCCAGGGAAGGATGTACCGCAAGTTCTTTTACAAGAATTT
GCTTCCATCGTTCCTAGTCTGTTAGAGACCTTTGGTAATGATATCAATCAACCCCTTACT
TGGGAACGTCTCCATGTCGCTACAACGATGGGACCGTCTGGGCCCTCTATGTTACAATCG
GCAACTCTCATTCCAAAAGTCCTCAAGAGGTTTAAACACCTGTTTGGGGCTTTAGGAGCT
GAGGAATTGCTTAAGTACATGGAGAGCATACCTGAGTCTTTCTCAAAACATTGGGAAAGT
CTATATCCTCAGAAACACAGTGATGTGTTAAGGAGGGTGTCGACCGTTCCTGATGTAGAT
GGAAAGACCAGATTAATAGCGATTCTAGACTATTGGTCACAATCAATCCTGAAAGTCTAT
CACAAGGACCTAATGTCCTCATTGATGAGAATTCAGAAAATTGATATGACCTTTGGTCAA
GGAATTGCCCCATTCGGTCCTGAAGACCAAAAGTACTATAGCTTTGACCTAACTGCCGCT
ACCGATCGTTTTCCAGTTGCAATAACTGAAATAATGATGGCTGCGAAGTATGGACAAGAA
GCTGCTAGTGCTTGAAAGGAAATCATGACAGGTGAGAAGTTCCATTGGAAGGACAAGGTT
CTTAAATACAACCGAGGCCAACCAATGGGAGCTTACTCGTCATGGGCTTCTTTCAGTCTT
AATCATCACATGGTTGTCCAGTGATCTGCATTAAGGGCGGGAGTGGAAACTCCTTTCCTA
GATTACAGACTACTAGGTGATGACATCGTAATACGGAATGACGCTGTTGCGAATTCATAC
CTCAAATTGATGGAACATCTAGGAGTCGAAATCTCCTCAGCTAAAACTCTAATAAGTGAA
AACTCATTTGAGTTCGCTAAAAGATTTTGGCTCAATGGTGAAGAAGTTACAGGATACCCA
ATTGCTGGGCTCCTGAACACCTTTACCAGATGATCTGAATTTCTTCAGGTCACTAGAGAA
GCATCAAGAAGAGGGTATGATTCCCTCATCACTATTCCCGGAAGGAAGTTTCGAGATCTG
TTTACCTCTCTACACTTTGCCGATCCATGTAAGAAAACGAAGGTATTAACTTCGCGTTCT
TACAGTGAAAGACTAGTTAGAAAGATGATCAGTTTAAATTGATTATGGGAGAAAGGGGCG
GACATCGAGCAAACAGTTAAGTTTGCTCAAATCTGATCCTTCAACCATAGCTGTAATATG
AGTTTAAAGACTCTACGACTTATCATCATAGAGTCATTAGCTCAAATAAAAGCAAATCAA
CTTCTCGATTCTTGTAGAACTCAGGTTAAAACCATTAATGGTTTTATAACTAAAGTCAAA
CAAGGACTTCCTAGTGATATAGAGATCCCCTTAACCGAGGTACCGCTGATAAAGGCCTTA
ATGGTCAATTTCAGTGATATGCAAGGTCAAATGGAAGATTTCAGAGAGTTGAGTAAATCC
GTTTCACGGGATAATACTGAACCCTTTGAGAAAATTCTATTCGAGGAATTGTTGATACCA
GAAATCAATCCGGAAATCTTAGACTCCTCAAGGAGGGCTCTTCGAATTGCGACAACTGAT
AAGCAAATGCTCATCAAGGGATGTGTACTCCTCCGTAATACTTCACAAGCTATTACTGAG
GAATTATCCAACCCTAATGTCGAACCCGACGAAGAAGACTATCAC**TAG**TTCTACTTCTCC
CAATTGCTAAAACACATATTCGTATGTGAGGTCCGGCAATTGAGTATGGCGCTCGATCAG
AGCGCCTGGACCCCTCCCG
