# Supplementary material for: Mitovirus and Mitochondrial Coding Sequences from Basal Fungus Entomophthora muscae
Source: Viruses. 2019 Apr 17;11(4):351. doi: 10.3390/v11040351 (PMC6520771; doi:10.3390/v11040351)
Supplement: Supplementary file 1 [file viruses-11-00351-s001.zip › Supp11-EnmuMV-DataS2.pdf]

***E. muscae* Mitochondrial Coding Sequences**

Terminal sequences of each assembly trimmed back to the proposed ORF

Red shading: first in-frame AUG codon and downstream stop codon
Orange shading: proposed alternative start codon
Gray shading: UGA(Trp) codons
Cyan shading: mismatches relative to the consensus sequence

N residues (cyan lettering) were inserted to fill gaps or truncations relative to the conserved sequences of other strains.

>atp6-KVL-14-117
ATGTTATTGTTTAGAAATCCTTTAGAACAATTTATGATTAACGAGTTATTAACGATATTA
TTCCCTTTCTGGGAAGTGTCATTAACAAATATAGGTTTATATTTAATAATGAGTTTAGTA
CTAATAGTAATAATATCACAAATCATCCTCGGCTCGACGGGGGGTGTGCCGATAATAGGG
AATAAAAGTATATTAATAAAACAATCAATATATGATACAGTGCATAAGATAGTAAAAGAT
CAGATAGGTATATCACATGAAATAAATTTACCTTTTTTATACACATTATTTATATTAGTA
TTGACATTAAATTTAATTGGAATTATTCCTTATAATTATAGTACAACCTCGCATCTCGTT
TTAACATTAAGTATGAGTGTATCAATATTAATAGGAGTGACAGTAATGGGAGTAAACCGA
CATAAATTAGTATTTTTTAGTCTACTAATCCCTGGGGGTACTCCGTTAGGTCTGGTACCT
CTATTAGTAATAATAGAAACAATATCTTATCTGGCTAGAGCTATTAGCTTAGGAGTAAGA
TTGGGTGCAAATATGATAGCAGGTCATGTTTTATTAAAAATCTTAGCAGGATTAATCTTG
AAAATAATGAAAACTTCGTTAATAACCGGCTTAATAATAGGATGCCTGCCTATGTTTATC
TTTACTTTATTAGTAGGATTAGAATTAGGTATAAGTGTATTACAAGGGATAGTATTTCTC
ATTTTAACTTCATCTTATATAAAAGATGCTATTGCTTTACAT**TAG**
>atp6-KVL-14-118
ATGTTATTGTTTAGAAATCCTTTAGAACAATTTATGATTAACGAGTTATTAACGATATTA
TTCCCTTTCTGGGAAGTGTCATTAACAAATATAGGTTTATATTTAATAATGAGTTTAGTA
CTAATAGTAATAATATCACAAATCATCCTCGGCTCGACGGGGGGTGTGCCGATAATAGGG
AATAAAAGTATATTAATAAAACAATCAATATATGATACAGTGCATAAGATAGTAAAAGAT
CAGATAGGTATATCACATGAAATAAATTTACCTTTTTTATACACATTATTTATATTAGTA
TTGACATTAAATTTAATTGGAATTATTCCTTATAATTATAGTACAACCTCGCATCTCGTT
TTAACATTAAGTATGAGTGTATCAATATTAATAGGAGTGACAGTAATGGGAGTAAACCGA
CATAAATTAGTATTTTTTAGTCTACTAATCCCTGGGGGTACTCCGTTAGGTCTGGTACCT
CTATTAGTAATAATAGAAACAATATCTTATCTGGCTAGAGCTATTAGCTTAGGAGTAAGA
TTGGGTGCAAATATGATAGCAGGTCATGTTTTATTAAAAATCTTAGCAGGATTAATCTTG
AAAATAATGAAAACTTCGTTAATAACCGGCTTAATAATAGGATGCCTGCCTATGTTTATC
TTTACTTTATTAGTAGGATTAGAATTAGGTATAAGTGTATTACAAGGGATAGTATTTCTC
ATTTTAACTTCATCTTATATAAAAGATGCTATTGCTTTACAT**TAG**
>atp6-HHdFL130914-1
ATGTTATTGTTTAGAAATCCTTTAGAACAATTTATGATTAACGAGTTATTAACGATATTA

```
TTCCCTTTCTGGGAAGTGTCATTAACAAATATAGGTTTATATTTAATAATGAGTTTAGTA
CTAATAGTAATAATATCACAAATCATCCTCGGCTCGACGGGGGGTGTGCCGATAATAGGG
AATAAAAGTATATTAATAAAACAATCAATATATGATACAGTGCATAAGATAGTAAAAGAT
CAGATAGGTATATCACATGAAATAAATTTACCTTTTTTATACACATTATTTATATTAGTA
TTGACATTAAATTTAATTGGAATTATTCCTTATAATTATAGTACAACCTCGCATCTCGTT
TTAACATTAAGTATGAGTGTATCAATATTAATAGGAGTGACAGTAATGGGAGTAAACCGA
CATAAATTAGTATTTTTTAGTCTACTAATCCCTGGGGGTACTCCGTTAGGTCTGGTACCT
CTATTAGTAATAATAGAAACAATATCTTATCTGGCTAGAGCTATTAGCTTAGGAGTAAGA
TTGGGTGCAAATATGATAGCAGGTCATGTTTTATTAAAAATCTTAGCAGGATTAATCTTG
AAAATAATGAAAACTTCGTTAATAACCGGCTTAATAATAGGATGCCTGCCTATGTTTATC
TTTACTTTATTAGTAGGATTAGAATTAGGTATAAGTGTATTACAAGGATAGTATTTCTC
ATTTTAACTTCATCTTATATAAAAGATGCTATTGCTTTACATTAG
>atp6-Berkeley
ATGTTATTGTTTAGAAATCCTTTAGAACAATTTATGATTAACGAGTTATTAACGATATTA
TTCCCTTTCTGGGAAGTGTCATTAACAAATATAGGTTTATATTTAATAATGAGTTTAGTA
CTAATAGTAATAATATCACAAATCATCCTCGGCTCGACGGGGGGTGTGCCGATAATAGGG
AATAAAAGTATATTAATAAAACAATCAATATATGATACAGTGCATAAGATAGTAAAAGAT
CAGATAGGTATATCACATGAAATAAATTTACCTTTTTTATACACATTATTTATATTAGTA
TTGACATTAAATTTAATTGGAATTATTCCTTATAATTATAGTACAACCTCGCATCTCGTT
TTAACATTAAGTATGAGTGTATCAATATTAATAGGAGTGACAGTAATGGGAGTAAACCGA
CATAAATTAGTATTTTTTAGTCTACTAATCCCTGGGGGTACTCCGTTAGGTCTGGTACCT
CTATTAGTAATAATAGAAACAATATCTTATCTGGCTAGAGCTATTAGCTTAGGAGTAAGA
TTGGGTGCAAATATGATAGCAGGTCATGTTTTATTAAAAATCTTAGCAGGATTAATCTTG
AAAATAATGAAAACTTCGTTAATAACCGGCTTAATAATAGGATGCCTGCCTATGTTTATC
TTTACTTTATTAGTAGGATTAGAATTAGGTATAAGTGTATTACAAGGATAGTATTTCTC
ATTTTAACTTCATCTTATATAAAAGATGCTATTGCTTTACATTAG
>atp6-HHdFL050913-1
ATGTTATTGCTTAAAAATCCTTTAGAACAATTTATGATTAACGAGTTATTAACGATATTA
TTCCCTTTCTGGGAAGTGTCATTAACAAATATAGGTTTATATTTAATAATGAGTTTAGTA
CTAATAGTAATAATATCACAAATCATCCTCGGCTCGACGGGGGGTGTGCCGATAATAGGG
AATAAAAGTATATTAATAAAACAATCAATATATGATACAGTGCATAAGATAGTAAAAGAT
CAGATAGGTATATCACATGAAATAAATTTACCTTTTTTATACACATTATTTATATTAGTA
TTGACATTAAATTTAATTGGAATCATTCCTTATAATTATAGTACAACCTCGCATCTCGTT
TTAACATTAAGTATGAGTGTATCAATATTAATAGGAGTGACAGTAATGGGAGTAAACCGA
CATAAATTAGTATTTTTTAGTCTACTAATCCCTGGGGGTACTCCGTTAGGTCTGGTACCT
CTATTAGTAATAATAGAAACAATATCTTATCTGGCTAGAGCTATTAGCTTAGGAGTAAGA
TTGGGTGCAAATATGATAGCAGGTCATGTTTTATTAAAAATCTTAGCAGGATTAATCTTG
AAAATAATGAAAACTTCGTTAATAACCGGCTTAATAATAGGATGCCTGCCTATGTTTATC
TTTACTTTATTAGTAGGATTAGAATTAGGTATAAGTGTATTACAAGGATAGTATTTCTC
ATTTTAACTTCATCTTATATAAAAGATGCTATTGCTTTACATTAG
>atp6-consensus
ATGTTATTGTTTAGAAATCCTTTAGAACAATTTATGATTAACGAGTTATTAACGATATTA
TTCCCTTTCTGGGAAGTGTCATTAACAAATATAGGTTTATATTTAATAATGAGTTTAGTA
CTAATAGTAATAATATCACAAATCATCCTCGGCTCGACGGGGGGTGTGCCGATAATAGGG
```

AATAAAAGTATATTAATAAAACAATCAATATATGATACAGTGCATAAGATAGTAAAAGAT
CAGATAGGTATATCACATGAAATAAATTTACCTTTTTTATACACATTATTTATATTAGTA
TTGACATTAAATTTAATTGGAATTATTCCTTATAATTATAGTACAACCTCGCATCTCGTT
TTAACATTAAGTATGAGTGTATCAATATTAATAGGAGTGACAGTAATGGGAGTAAACCGA
CATAAATTAGTATTTTTTAGTCTACTAATCCCTGGGGGTACTCCGTTAGGTCTGGTACCT
CTATTAGTAATAATAGAAACAATATCTTATCTGGCTAGAGCTATTAGCTTAGGAGTAAGA
TTGGGTGCAAATATGATAGCAGGTCATGTTTTATTAAAAATCTTAGCAGGATTAATCTTG
AAAATAATGAAAACTTCGTTAATAACCGGCTTAATAATAGGATGCCTGCCTATGTTTATC
TTTACTTTATTAGTAGGATTAGAATTAGGTATAAGTGTATTACAAGGGATAGTATTTCTC
ATTTTAACTTCATCTTATATAAAAGATGCTATTGCTTTACAT**TAG**

>atp9-KVL-14-117
ATGTTAGAGTCTGCCAAAATTATAGGATCAGGAATAGCAACGATAGGTTTAGCCGGTGCG
GGTGTGGGTATAGGTTTAGTGTTTGCCGCTCTAATAAATTCAACATCTAGAAATCCTTCA
TTAAAAGGACAATTATTCAGTTATTGTATTTTAGGTTTTGCTTTAACTGAAGCTATAGGC
CTTTTTGCAATAATGATGTCATTCTTATTATTATATGCCGCT**TAA**
>atp9-KVL-14-118
ATGTTAGAGTCTGCCAAAATTATAGGATCAGGAATAGCAACGATAGGTTTAGCCGGTGCG
GGTGTGGGTATAGGTTTAGTGTTTGCCGCTCTAATAAATTCAACATCTAGAAATCCTTCA
TTAAAAGGACAATTATTCAGTTATTGTATTTTAGGTTTTGCTTTAACTGAAGCTATAGGC
CTTTTTGCAATAATGATGTCATTCTTATTATTATATGCCGCT**TAA**
>atp9-HHdFL130914-1
ATGTTAGAGTCTGCCAAAATTATAGGATCAGGAATAGCAACGATAGGTTTAGCCGGTGCG
GGTGTGGGTATAGGTTTAGTGTTTGCCGCTCTAATAAATTCAACATCTAGAAATCCTTCA
TTAAAAGGACAATTATTCAGTTATTGTATTTTAGGTTTTGCTTTAACTGAAGCTATAGGC
CTTTTTGCAATAATGATGTCATTCTTATTATTATATGCCGCT**TAA**
>atp9-Berkeley
ATGTTAGAGTCTGCCAAAATTATAGGATCAGGAATAGCAACGATAGGTTTAGCCGGTGCG
GGTGTGGGTATAGGTTTAGTGTTTGCCGCTCTAATAAATTCAACATCTAGAAATCCTTCA
TTAAAAGGACAATTATTCAGTTATTGTATTTTAGGTTTTGCTTTAACTGAAGCTATAGGC
CTTTTTGCAATAATGATGTCATTCTTATTATTATATGCCGCT**TAA**
>atp9-HHdFL050913-1
ATGTTAGAGTCTGCCAAAATTATAGGATCAGGAATAGCAACGATAGGTTTAGCCGGTGCG
GGTGTGGGTATAGGTTTAGTGTTTGCCGCTCTAATAAATTCAACATCTAGAAATCCTTCA
TTAAAAGGACAATTATTCAGTTATTGTATTTTAGGTTTTGCTTTAACTGAAGCTATAGGC
CTTTTTGCAATAATGATGTCATTCTTATTATTATATGCCGCT**TAA**
>atp9-consensus
ATGTTAGAGTCTGCCAAAATTATAGGATCAGGAATAGCAACGATAGGTTTAGCCGGTGCG
GGTGTGGGTATAGGTTTAGTGTTTGCCGCTCTAATAAATTCAACATCTAGAAATCCTTCA
TTAAAAGGACAATTATTCAGTTATTGTATTTTAGGTTTTGCTTTAACTGAAGCTATAGGC
CTTTTTGCAATAATGATGTCATTCTTATTATTATATGCCGCT**TAA**

>cob-KVL-14-117
ATGGCAAATAGTTATATAATTGATTCCCCTCCCCCAGTAATTTAACGTATTTATGGGGT

TATGGGTCATTATTAGGCCTAGTATTAGTTATGCAAATAATAACCGGTGTAACTCTAGCT
ATGCATTATACTCCTAACATTGAGATGGCATTTAATTCAGTTGAACATATCATGAGAGAC
GTAAATAACGGCTGATTAATTCGTTACACTCACGCAAATGTAGCGTCATTTTTTTTTATA
TTTTTATATATCCATATAGGAAGAGGTTTATATTATGGTTCTTATACCAGACCAAGGATA
TTATTATGGTCTATAGGAGTAATAATATTTGTATTAACAATGGCAACAGGGTTTTTGGGT
TATACTCTTCCCTGAGGTCAAATGAGCCTATGAGGTGCAACGGTTATAACTAATTTATTA
TCGGCAATACCTTGGATGGGAGTTAATATAGTGGAATTTGTCTGGGGTGGATTTTCCGTG
GATAATGCGACATTAAACAGATTTTTCAGTCTTCATTATTTATTACCCTTTATATTAGTG
GGATTAGTGGTATTACATTTAGTAGCTCTTCATCACCATGGAAGTAATAACCCATTAGGG
GTATCAGCCAATGGTGATAGAGTACCATTCCATCCTTATTATACTTTTAAAGATATAGTA
GGATTTTTAGCCTTCTTTTTAGTATTATCAATAATTGTCTTCTATATGCCTAATTTATTA
GGTCATTCAGACAACTACATACCAGCCAACCCGCTGGTAACACCAACTCACATACAACCA
GAATGGTATTTTTTACCTTATTATGCAATATTACGATCAATCCCTAATAAGCTATTAGGG
GTTATAGCAATGTTTGCTGCGATATTAATATTATTAGCAATGCCTTTCTTAGATGAATCA
AGAATACGAGGAAGTCAATTTAGACCTTTTATGAAAATAATCTTTTGGTTATTTATATCA
AATTTCGTATTATTAGGATGGATAGGTGCTAAACCAGTATCAGATCCTTACATAATAATA
GGTCAGTTAAGTTCAATATTTTATTTTGCATGGTTTGCAATAGTAGCCATGGTAGGGATA
ATAGAAAATACTCTATATCAAATGAGACAACTTTGGCTGGGGGCCAAA**TAG**
>cob-KVL-14-118
ATGGCAAATAGTTATATAATTGATTCCCCCTCCCCCAGTAATTTAACGTATTTATGGGGT
TATGGGTCATTATTAGGCCTAGTATTAGTTATGCAAATAATAACCGGTGTAACTCTAGCT
ATGCATTATACTCCTAACATTGAGATGGCATTTAATTCAGTTGAACATATCATGAGAGAC
GTAAATAACGGCTGATTAATTCGTTACACTCACGCAAATGTAGCGTCATTTTTTTTTATA
TTTTTATATATCCATATAGGAAGAGGTTTATATTATGGTTCTTATACCAGACCAAGGATA
TTATTATGGTCTATAGGAGTAATAATATTTGTATTAACAATGGCAACAGGGTTTTTGGGT
TATACTCTTCCCTGAGGTCAAATGAGCCTATGAGGTGCAACGGTTATAACTAATTTATTA
TCGGCAATACCTTGGATGGGAGTTAATATAGTGGAATTTGTCTGGGGTGGATTTTCCGTG
GATAATGCGACATTAAACAGATTTTTCAGTCTTCATTATTTATTACCCTTTATATTAGTG
GGATTAGTGGTATTACATTTAGTAGCTCTTCATCACCATGGAAGTAATAACCCATTAGGG
GTATCAGCCAATGGTGATAGAGTACCATTCCATCCTTATTATACTTTTAAAGATATAGTA
GGATTTTTAGCCTTCTTTTTAGTATTATCAATAATTGTCTTCTATATGCCTAATTTATTA
GGTCATTCAGACAACTACATACCAGCCAACCCGCTGGTAACACCAACTCACATACAACCA
GAATGGTATTTTTTACCTTATTATGCAATATTACGATCAATCCCTAATAAGCTATTAGGG
GTTATAGCAATGTTTGCTGCGATATTAATATTATTAGCAATGCCTTTCTTAGATGAATCA
AGAATACGAGGAAGTCAATTTAGACCTTTTATGAAAATAATCTTTTGGTTATTTATATCA
AATTTCGTATTATTAGGATGGATAGGTGCTAAACCAGTATCAGATCCTTACATAATAATA
GGTCAGTTAAGTTCAATATTTTATTTTGCATGGTTTGCAATAGTAGCCATGGTAGGGATA
ATAGAAAATACTCTATATCAAATGAGACAACTTTGGCTGGGGGCCAAA**TAG**
>cob-HHdFL130914-1
ATGGCAAATAGTTATATAATTGATTCCCCCTCCCCCAGTAATTTAACGTATTTATGGGGT
TATGGGTCATTATTAGGCCTAGTATTAGTTATGCAAATAATAACCGGTGTAACTCTAGCT
ATGCATTATACTCCTAACATTGAGATGGCATTTAATTCAGTTGAACATATCATGAGAGAC
GTAAATAACGGCTGATTAATTCGTTACACTCACGCAAATGTAGCGTCATTTTTTTTTATA
TTTTTATATATCCATATAGGAAGAGGTTTATATTATGGTTCTTATACCAGACCAAGGATA

TTATTATGGTCTATAGGAGTAATAATATTTGTATTAACAATGGCAACAGGGTTTTTGGGT
TATACTCTTCCCTGAGGTCAAATGAGCCTATGAGGTGCAACGGTTATAACTAATTTATTA
TCGGCAATACCTTGGATGGGAGTTAATATAGTGGAATTTGTCTGGGGTGGATTTTCCGTG
GATAATGCGACATTAAACAGATTTTTCAGTCTTCATTATTTATTACCCTTTATATTAGTG
GGATTAGTGGTATTACATTTAGTAGCTCTTCATCACCATGGAAGTAATAACCCATTAGGG
GTATCAGCCAATGGTGATAGAGTACCATTCCATCCTTATTATACTTTTAAAGATATAGTA
GGATTTTTAGCCTTCTTTTTAGTATTATCAATAATTGTCTTCTATATGCCTAATTTATTA
GGTCATTCAGACAACTACATACCAGCCAACCCGCTGGTAACACCAACTCACATACAACCA
GAATGGTATTTTTTACCTTATTATGCAATATTACGATCAATCCCTAATAAGCTATTAGGG
GTTATAGCAATGTTTGCTGCGATATTAATATTATTAGCAATGCCTTTCTTAGATGAATCA
AGAATACGAGGAAGTCAATTTAGACCTTTTATGAAAATAATCTTTTGGTTATTTATATCA
AATTTCGTATTATTAGGATGGATAGGTGCTAAACCAGTATCAGATCCTTACATAATAATA
GGTCAGTTAAGTTCAATATTTTATTTTGCATGGTTTGCAATAGTAGCCATGGTAGGGATA
ATAGAAAATACTCTATATCAAATGAGACAACTTTGGCTGGGGGCCAAA**TAG**
>cob-Berkeley
ATGGCAAATAGTTATATAATTGATTCCCCCTCCCCCAGTAATTTAACGTATTTATGGGGT
TATGGGTCATTATTAGGCCTAGTATTAGTTATGCAAATAATAACCGGTGTAACTCTAGCT
ATGCATTATACTCCTAACATTGAGATGGCATTTAATTCAGTTGAACATATCATGAGAGAC
GTAAATAACGGCTGATTAATTCGTTACACTCACGCAAATGTAGCGTCATTTTTTTTTATA
TTTTTATATATCCATATAGGAAGAGGTTTATATTATGGTTCTTATACCAGACCAAGGATA
TTATTATGGTCTATAGGAGTAATAATATTTGTATTAACAATGGCAACAGGGTTTTTGGGT
TATACTCTTCCCTGAGGTCAAATGAGCCTATGAGGTGCAACGGTTATAACTAATTTATTA
TCGGCAATACCTTGGATGGGAGTTAATATAGTGGAATTTGTCTGGGGTGGATTTTCCGTG
GATAATGCGACATTAAACAGATTTTTCAGTCTTCATTATTTATTACCCTTTATATTAGTG
GGATTAGTGGTATTACATTTAGTAGCTCTTCATCACCATGGAAGTAATAACCCATTAGGG
GTATCAGCCAATGGTGATAGAGTACCATTCCATCCTTATTATACTTTTAAAGATATAGTA
GGATTTTTAGCCTTCTTTTTAGTATTATCAATAATTGTCTTCTATATGCCTAATTTATTA
GGTCATTCAGACAACTACATACCAGCCAACCCGCTGGTAACACCAACTCACATACAACCA
GAATGGTATTTTTTACCTTATTATGCAATATTACGATCAATCCCTAATAAGCTATTAGGG
GTTATAGCAATGTTTGCTGCGATATTAATATTATTAGCAATGCCTTTCTTAGATGAATCA
AGAATACGAGGAAGTCAATTTAGACCTTTTATGAAAATAATCTTTTGGTTATTTATATCA
AATTTCGTATTATTAGGATGGATAGGTGCTAAACCAGTATCAGATCCTTACATAATAATA
GGTCAGTTAAGTTCAATATTTTATTTTGCATGGTTTGCAATAGTAGCCATGGTAGGGATA
ATAGAAAATACTCTATATCAAATGAGACAACTTTGGCTGGGGGCCAAA**TAG**
>cob-HHdFL050913-1
ATGGCAAATAGTTATATAATTGATTCCCCCTCCCCCAGTAATTTAACGTATTTATGGGGT
TATGGGTCATTATTAGGCCTAGTATTAGTTATGCAAATAATAACCGGTGTAACTCTAGCT
ATGCATTATACTCCTAACATTGAGATGGCATTTAATTCAGTTGAACATATCATGAGAGAC
GTAAATAACGGCTGATTAATTCGTTACACTCACGCAAATGTAGCGTCATTTTTTTTTATA
TTTTTATATATCCATATAGGAAGAGGTTTATATTATGGTTCTTATACCAGACCAAGGATA
TTATTATGGTCTATAGGAGTAATAATATTTGTATTAACAATGGCAACAGGGTTTTTGGGT
TATACTCTTCCCTGAGGTCAAATGAGCCTATGAGGTGCAACGGTTATAACTAATTTATTA
TCGGCAATACCTTGGATGGGAGTAAATATAGTGGAATTTGTCTGGGGTGGATTTTCCGTG
GATAATGCGACATTAAACAGATTTTTCAGTCTTCATTATTTATTACCCTTTATATTAGTG

GGATTAGTGGTATTACATTTAGTAGCTCTTCATCACCATGGAAGTAATAACCCATTAGGG
GTATCAGCCAATGGTGATAGAGTACCATTCCATCCTTATTATACTTTTAAAGATATAGTA
GGATTTTTAGCCTTCTTTTTAGTATTATCAATAATTGTCTTCTATATGCCTAATTTATTA
GGTCATTCAGACAACTACATACCAGCCAACCCGCTGGTAACACCAACTCACATACAACCA
GAATGGTATTTTTTACCTTATTATGCAATATTACGATCAATCCCTAATAAGCTATTAGGG
GTTATAGCAATGTTTGCTGCGATATTAATATTATTAGCAATGCCTTTCTTAGATGAATCA
AGAATACGAGGAAGTCAATTTAGACCTTTTATGAAAATAATCTTTTGGTTATTTATATCA
AATTTCGTATTATTAGGATGGATAGGTGCTAAACCAGTATCAGATCCTTACATAATAATA
GGTCAGTTAAGTTCAATATTTTATTTTGCATGGTTTGCAATAGTAGCCATGGTAGGGATA
ATAGAAAATACTCTATATCAAATGAGACAACTTTGGCTGGGGGCCAAA**TAG**
>cob-consensus
ATGGCAAATAGTTATATAATTGATTCCCCCTCCCCCAGTAATTTAACGTATTTATGGGGT
TATGGGTCATTATTAGGCCTAGTATTAGTTATGCAAATAATAACCGGTGTAACTCTAGCT
ATGCATTATACTCCTAACATTGAGATGGCATTTAATTCAGTTGAACATATCATGAGAGAC
GTAAATAACGGCTGATTAATTCGTTACACTCACGCAAATGTAGCGTCATTTTTTTTTATA
TTTTTATATATCCATATAGGAAGAGGTTTATATTATGGTTCTTATACCAGACCAAGGATA
TTATTATGGTCTATAGGAGTAATAATATTTGTATTAACAATGGCAACAGGGTTTTTGGGT
TATACTCTTCCCTGAGGTCAAATGAGCCTATGAGGTGCAACGGTTATAACTAATTTATTA
TCGGCAATACCTTGGATGGGAGTTAATATAGTGGAATTTGTCTGGGGTGGATTTTCCGTG
GATAATGCGACATTAAACAGATTTTTCAGTCTTCATTATTTATTACCCTTTATATTAGTG
GGATTAGTGGTATTACATTTAGTAGCTCTTCATCACCATGGAAGTAATAACCCATTAGGG
GTATCAGCCAATGGTGATAGAGTACCATTCCATCCTTATTATACTTTTAAAGATATAGTA
GGATTTTTAGCCTTCTTTTTAGTATTATCAATAATTGTCTTCTATATGCCTAATTTATTA
GGTCATTCAGACAACTACATACCAGCCAACCCGCTGGTAACACCAACTCACATACAACCA
GAATGGTATTTTTTACCTTATTATGCAATATTACGATCAATCCCTAATAAGCTATTAGGG
GTTATAGCAATGTTTGCTGCGATATTAATATTATTAGCAATGCCTTTCTTAGATGAATCA
AGAATACGAGGAAGTCAATTTAGACCTTTTATGAAAATAATCTTTTGGTTATTTATATCA
AATTTCGTATTATTAGGATGGATAGGTGCTAAACCAGTATCAGATCCTTACATAATAATA
GGTCAGTTAAGTTCAATATTTTATTTTGCATGGTTTGCAATAGTAGCCATGGTAGGGATA
ATAGAAAATACTCTATATCAAATGAGACAACTTTGGCTGGGGGCCAAA**TAG**

>cox1-KVL-14-117
GTGTACGCTATAAGGTGGTTATACTCGACCAATGCCCGAGATATTGGGGTATTATATTTA
ATTTTTGCCATCTTGGCGGGATTAATAGGGACAGTATTATCAATCATAATAAGATTAGAA
TTAGGAGGTGCGGGGGTTCAATATTTACAAGGAGATAACCAATTATACAATGTGGTTGTG
ACGGCACACGCATTCGTC**ATG**ATTTTTTTCATGGTGATGCCGGCGTTAATAGGGGGTTTT
GGGAATTTTATAGTTCCAGTGATGATAGGTGCCGCTGATATGGCATTCCCAAGATTAAAT
AATATATCATTTTGGTTATTACCTCCATCATTGATATTATTATTATTATCATCATTAGTT
GAGAATGGTGCGGGTACTGGTTGGACTGTTTATCCTCCATTATCATCAATCCAAGCTCAC
TCAGGAGGATCGGTTGATTTAGCAATATTCTCGCTTCACCTGGCAGGGATATCTTCTATG
TTAGGTGCAATAAATTTCTTAACAACAATAATTAATATGCGAGCCCCGGGTATGAGCTGG
CATAAATTGCCGTTATTTGTGTGGGCAGTATTTATAACTGCAATATTATTATTATTATCA
TTACCAGTGTTAGCGGGTGGAATAACTATGCTTCTTACAGATAGAAACTTTAATAGTTCT
TTCTATGACCCATCAGCTGGGGGTGATCCAGTGCTTTATCAGCATATTTTCTGATTCTTT

GGTCATCCTGAAGTGTATATATTAATTATACCAGGATTTGGTATAATAAGTCATGTAATA
TCAACTTATTCCGGTAAAAGAGTATTCGGTTATCTAGGTATGATATACGCATTAGCTTCA
ATAGGAATCTTAGGATTCATAGTATGGTCTCATCACATGTTCACTGTAGGTCTAGATGTC
GATACAAGAGCATATTTCACTTCAGCGACATTAATAATTGCAGTGCCAACAGGGATAAAA
ATATTCTCGTGGCTGGCGACGATATACGGGGGAAGTATAAGATTTAGTACTCCTATGTTA
TTTTCTATGGGATTCATATTTTTATTTACAGTAGGAGGATTAACTGGAGTAATATTAGCC
AATGCCTCGTTAGATATAGCTTTACATGATACTTACTACGTGGTAGCACATTTCCATTAT
GTATTATCAATGGGAGCAGTATTTGCATTATTTGCAGCATTCTATTATTGAATAGGTAAA
ATTACTGGAAAACAATATAATGAATTATTAGGGCAGATACATTTTTATACGATGTTTGTC
GGGGTAAATATAACATTCTTCCCAATGCATTTCTTAGGTCTGGCGGGTATGCCACGTAGA
ATACCAGACTATCCAGATGCTTTCATAGGATGGAATTATATAGCATCCTTAGGATCAATA
ATAAGTTTAGTATCAACAGTATTATTCTTATACATAGTCTATGATTTATTAACTAATGAG
GTGGAAGCAAGCGACGGAGCCGATGTAGAAAACGAGTACTTTATATCGAGATGGTCAACT
AAATATGCTCAGACCTTAGAGTGGAGTTTAACAAGCCCACCTGGCTTCCATTGTTATAAT
AGTTTACCAACCTTCCTAACGAAG**TAA**
>cox1-KVL-14-118
GTGTACGCTATAAGGTGGTTATACTCGACCAATGCCCGAGATATTGGGGTATTATATTTA
ATTTTTGCCATCTTGGCGGGATTAATAGGGACAGTATTATCAATCATAATAAGATTAGAA
TTAGGAGGTGCGGGGGTTCAATATTTACAAGGAGATAACCAATTATACAATGTGGTTGTG
ACGGCACACGCATTCGTC**ATG**ATTTTTTTCATGGTGATGCCGGCGTTAATAGGGGGTTTT
GGGAATTTTATAGTTCCAGTGATGATAGGTGCCGCTGATATGGCATTCCCAAGATTAAAT
AATATATCATTTTGGTTATTACCTCCATCATTGATATTATTATTATTATCATCATTAGTT
GAGAATGGTGCGGGTACTGGTTGGACTGTTTATCCTCCATTATCATCAATCCAAGCTCAC
TCAGGAGGATCGGTTGATTTAGCAATATTCTCGCTTCACCTGGCAGGGATATCTTCTATG
TTAGGTGCAATAAATTTCTTAACAACAATAATTAATATGCGAGCCCCGGGTATGAGCTGG
CATAAATTGCCGTTATTTGTGTGGGCAGTATTTATAACTGCAATATTATTATTATTATCA
TTACCAGTGTTAGCGGGTGGAATAACTATGCTTCTTACAGATAGAAACTTTAATAGTTCT
TTCTATGACCCATCAGCTGGGGGTGATCCAGTGCTTTATCAGCATATTTTCTGATTCTTT
GGTCATCCTGAAGTGTATATATTAATTATACCAGGATTTGGTATAATAAGTCATGTAATA
TCAACTTATTCCGGTAAAAGAGTATTCGGTTATCTAGGTATGATATACGCATTAGCTTCA
ATAGGAATCTTAGGATTCATAGTATGGTCTCATCACATGTTCACTGTAGGTCTAGATGTC
GATACAAGAGCATATTTCACTTCAGCGACATTAATAATTGCAGTGCCAACAGGGATAAAA
ATATTCTCGTGGCTGGCGACGATATACGGGGGAAGTATAAGATTTAGTACTCCTATGTTA
TTTTCTATGGGATTCATATTTTTATTTACAGTAGGAGGATTAACTGGAGTAATATTAGCC
AATGCCTCGTTAGATATAGCTTTACATGATACTTACTACGTGGTAGCACATTTCCATTAT
GTATTATCAATGGGAGCAGTATTTGCATTATTTGCAGCATTCTATTATTGAATAGGTAAA
ATTACTGGAAAACAATATAATGAATTATTAGGGCAGATACATTTTTATACGATGTTTGTC
GGGGTAAATATAACATTCTTCCCAATGCATTTCTTAGGTCTGGCGGGTATGCCACGTAGA
ATACCAGACTATCCAGATGCTTTCATAGGATGGAATTATATAGCATCCTTAGGATCAATA
ATAAGTTTAGTATCAACAGTATTATTCTTATACATAGTCTATGATTTATTAACTAATGAG
GTGGAAGCAAGCGACGGAGCCGATGTAGAAAACGAGTACTTTATATCGAGATGGTCAACT
AAATATGCTCAGACCTTAGAGTGGAGTTTAACAAGCCCACCTGGCTTCCATTGTTATAAT
AGTTTACCAACCTTCCTAACGAAG**TAA**
>cox1-HHdFL130914-1

GTGTACGCTATAAGGTGGTTATACTCGACCAATGCCCGAGATATTGGGGTATTATATTTA
ATTTTTGCCATCTTGGCGGGATTAATAGGGACAGTATTATCAATCATAATAAGATTAGAA
TTAGGAGGTGCGGGGGTTCAATATTTACAAGGAGATAACCAATTATACAATGTGGTTGTG
ACGGCACACGCATTCGTC**ATG**ATTTTTTTCATGGTGATGCCGGCGTTAATAGGGGGTTTT
GGGAATTTTATAGTTCCAGTGATGATAGGTGCCGCTGATATGGCATTCCCAAGATTAAAT
AATATATCATTTTGGTTATTACCTCCATCATTGATATTATTATTATTATCATCATTAGTT
GAGAATGGTGCGGGTACTGGTTGGACTGTTTATCCTCCATTATCATCAATCCAAGCTCAC
TCAGGAGGATCGGTTGATTTAGCAATATTCTCGCTTCACCTGGCAGGGATATCTTCTATG
TTAGGTGCAATAAATTTCTTAACAACAATAATTAATATGCGAGCCCCGGGTATGAGCTGG
CATAAATTGCCGTTATTTGTGTGGGCAGTATTTATAACTGCAATATTATTATTATTATCA
TTACCAGTGTTAGCGGGTGGAATAACTATGCTTCTTACAGATAGAAACTTTAATAGTTCT
TTCTATGACCCATCAGCTGGGGGTGATCCAGTGCTTTATCAGCATATTTTCTGATTCTTT
GGTCATCCTGAAGTGTATATATTAATTATACCAGGATTTGGTATAATAAGTCATGTAATA
TCAACTTATTCCGGTAAAAGAGTATTCGGTTATCTAGGTATGATATACGCATTAGCTTCA
ATAGGAATCTTAGGATTCATAGTATGGTCTCATCACATGTTCACTGTAGGTCTAGATGTC
GATACAAGAGCATATTTCACTTCAGCGACATTAATAATTGCAGTGCCAACAGGGATAAAA
ATATTCTCGTGGCTGGCGACGATATACGGGGGAAGTATAAGATTTAGTACTCCTATGTTA
TTTTCTATGGGATTCATATTTTTATTTACAGTAGGAGGATTAACTGGAGTAATATTAGCC
AATGCCTCGTTAGATATAGCTTTACATGATACTTACTACGTGGTAGCACATTTCCATTAT
GTATTATCAATGGGAGCAGTATTTGCATTATTTGCAGCATTCTATTATTGAATAGGTAAA
ATTACTGGAAAACAATATAATGAATTATTAGGGCAGATACATTTTTATACGATGTTTGTC
GGGGTAAATATAACATTCTTCCCAATGCATTTCTTAGGTCTGGCGGGTATGCCACGTAGA
ATACCAGACTATCCAGATGCTTTCATAGGATGGAATTATATAGCATCCTTAGGATCAATA
ATAAGTTTAGTATCAACAGTATTATTCTTATACATAGTCTATGATTTATTAACTAATGAG
GTGGAAGCAAGCGACGGAGCCGATGTAGAAAACGAGTACTTTATATCGAGATGGTCAACT
AAATATGCTCAGACCTTAGAGTGGAGTTTAACAAGCCCACCTGGCTTCCATTGTTATAAT
AGTTTACCAACCTTCCTAACGAAG**TAA**
>cox1-Berkeley
GTGTACGCTATAAGGTGGTTATACTCGACCAATGCCCGAGATATTGGGGTATTATATTTA
ATTTTTGCCATCTTGGCGGGATTAATAGGGACAGTATTATCAATCATAATAAGATTAGAA
TTAGGAGGTGCGGGGGTTCAATATTTACAAGGAGATAACCAATTATACAATGTGGTTGTG
ACGGCACACGCATTCGTC**ATG**ATTTTTTTCATGGTGATGCCGGCGTTAATAGGGGGTTTT
GGGAATTTTATAGTTCCAGTGATGATAGGTGCCGCTGATATGGCATTCCCAAGATTAAAT
AATATATCATTTTGGTTATTACCTCCATCATTGATATTATTATTATTATCATCATTAGTT
GAGAATGGTGCGGGTACTGGTTGGACTGTTTATCCTCCATTATCATCAATCCAAGCTCAC
TCAGGAGGATCGGTTGATTTAGCAATATTCTCGCTTCACCTGGCAGGGATATCTTCTATG
TTAGGTGCAATAAATTTCTTAACAACAATAATTAATATGCGAGCCCCGGGTATGAGCTGG
CATAAATTGCCGTTATTTGTGTGGGCAGTATTTATAACTGCAATATTATTATTATTATCA
TTACCAGTGTTAGCGGGTGGAATAACTATGCTTCTTACAGATAGAAACTTTAATAGTTCT
TTCTATGACCCATCAGCTGGGGGTGATCCAGTGCTTTATCAGCATATTTTCTGATTCTTT
GGTCATCCTGAAGTGTATATATTAATTATACCAGGATTTGGTATAATAAGTCATGTAATA
TCAACTTATTCCGGTAAAAGAGTATTCGGTTATCTAGGTATGATATACGCATTAGCTTCA
ATAGGAATCTTAGGATTCATAGTATGGTCTCATCACATGTTCACTGTAGGTCTAGATGTC
GATACAAGAGCATATTTCACTTCAGCGACATTAATAATTGCAGTGCCAACAGGGATAAAA

ATATTCTCGTGGCTGGCGACGATATACGGGGAAGTATAAGATTTAGTACTCCTATGTTA
TTTTCTATGGGATTCATATTTTTATTTACAGTAGGAGGATTAACTGGAGTAATATTAGCC
AATGCCTCGTTAGATATAGCTTTACATGATACTTACTACGTGGTAGCACATTTCCATTAT
GTATTATCAATGGGAGCAGTATTTGCATTATTTGCAGCATTCTATTATTGAATAGGTAAA
ATTACTGGAAAACAATATAATGAATTATTAGGGCAGATACATTTTTATACGATGTTTGTC
GGGGTAAATATAACATTCTTCCCAATGCATTTCTTAGGTCTGGCGGGTATGCCACGTAGA
ATACCAGACTATCCAGATGCTTTCATAGGATGGAATTATATAGCATCCTTAGGATCAATA
ATAAGTTTAGTATCAACAGTATTATTCTTATACATAGTCTATGATTTATTAACTAATGAG
GTGGAAGCAAGCGACGGAGCCGATGTAGAAAACGAGTACTTTATATCGAGATGGTCAACT
AAATATGCTCAGACCTTAGAGTGGAGTTTAACAAGCCCACCTGGCTTCCATTGTTATAAT
AGTTTACCAACCTTCCTAACGAAG**TAA**
>cox1-HHdFL050913-1
GTGTACGCTATAAGGTGGTTATACTCGACCAATGCCCGAGATATTGGGGTATTATATTTA
ATTTTTGCCATCTTGGCGGGATTAATAGGGACAGTATTATCAATCATAATAAGATTAGAA
TTAGGAGGTGCGGGGGTTCAATATTTACAAGGAGATAACCAATTATACAATGTGGTTGTG
ACGGCACACGCATTCGTC**ATG**ATTTTTTTCATGGTGATGCCGGCGTTAATAGGGGGTTTT
GGGAATTTTATAGTTCCAGTGATGATAGGTGCCGCTGATATGGCATTCCCAAGATTAAAT
AATATATCATTTTGGTTATTACCTCCATCATTGATATTATTATTATTATCATCATTAGTT
GAGAATGGTGCGGGTACTGGTTGGACTGTTTATCCTCCATTATCATCAATCCAAGCTCAC
TCAGGAGGATCGGTTGATTTAGCAATATTCTCGCTTCACCTGGCAGGGATATCTTCTATG
TTAGGTGCAATAAATTTCTTAACAACAATAATTAATATGCGAGCCCCGGGTATGAGCTGG
CATAAATTGCCGTTATTTGTGTGGGCAGTATTTATAACTGCAATATTATTATTATTATCA
TTACCAGTGTTAGCGGGTGGAATAACTATGCTTCTTACAGATAGAAACTTTAATAGTTCT
TTCTATGACCCATCAGCTGGGGGTGATCCAGTGCTTTATCAGCATATTTTCTGATTCTTT
GGTCATCCTGAAGTGTATATATTAATTATACCAGGATTTGGTATAATAAGTCATGTAATA
TCAACTTATTCCGGTAAAAGAGTATTCGGTTATCTAGGTATGATATACGCATTAGCTTCA
ATAGGAATCTTAGGATTCATAGTATGGTCTCATCACATGTTCACTGTAGGTCTAGATGTC
GATACAAGAGCATATTTCACTTCAGCGACATTAATAATTGCAGTGCCAACAGGGATAAAA
ATATTCTCGTGGCTGGCGACGATATACGGGGAAGTATAAGATTTAGTACTCCTATGTTA
TTTTCTATGGGATTCATATTTTTATTTACAGTAGGAGGATTAACTGGAGTAATATTAGCC
AATGCCTCGTTAGATATAGCTTTACATGATACTTACTACGTGGTAGCACATTTCCATTAT
GTATTATCAATGGGAGCAGTATTTGCATTATTTGCAGCATTCTATTATTGAATAGGTAAA
ATTACTGGAAAACAATATAATGAATTATTAGGGCAGATACATTTTTATACGATGTTTGTC
GGGGTAAATATAACATTCTTCCCAATGCATTTCTTAGGTCTGGCGGGTATGCCACGTAGA
ATACCAGACTATCCAGATGCTTTCATAGGATGGAATTATATAGCATCCTTAGGATCAATA
ATAAGTTTAGTATCAACAGTATTATTCTTATACATAGTCTATGATTTATTAACTAATGAG
GTGGAAGCAAGCGACGGAGCCGATGTAGAAAACGAGTACTTTATATCGAGATGGTCAACT
AAATATGCTCAGACCTTAGAGTGGAGTTTAACAAGCCCACCTGGCTTCCATTGTTATAAT
AGTTTACCAACCTTCCTAACGAAG**TAA**
>cox1-consensus
GTGTACGCTATAAGGTGGTTATACTCGACCAATGCCCGAGATATTGGGGTATTATATTTA
ATTTTTGCCATCTTGGCGGGATTAATAGGGACAGTATTATCAATCATAATAAGATTAGAA
TTAGGAGGTGCGGGGGTTCAATATTTACAAGGAGATAACCAATTATACAATGTGGTTGTG
ACGGCACACGCATTCGTC**ATG**ATTTTTTTCATGGTGATGCCGGCGTTAATAGGGGGTTTT

GGGAATTTTATAGTTCCAGTGATGATAGGTGCCGCTGATATGGCATTCCCAAGATTAAAT
AATATATCATTTTGGTTATTACCTCCATCATTGATATTATTATTATTATCATCATTAGTT
GAGAATGGTGCGGGTACTGGTTGGACTGTTTATCCTCCATTATCATCAATCCAAGCTCAC
TCAGGAGGATCGGTTGATTTAGCAATATTCTCGCTTCACCTGGCAGGGATATCTTCTATG
TTAGGTGCAATAAATTTCTTAACAACAATAATTAATATGCGAGCCCCGGGTATGAGCTGG
CATAAATTGCCGTTATTTGTGTGGGCAGTATTTATAACTGCAATATTATTATTATTATCA
TTACCAGTGTTAGCGGGTGGAATAACTATGCTTCTTACAGATAGAAACTTTAATAGTTCT
TTCTATGACCCATCAGCTGGGGGTGATCCAGTGCTTTATCAGCATATTTTCTGATTCTTT
GGTCATCCTGAAGTGTATATATTAATTATACCAGGATTTGGTATAATAAGTCATGTAATA
TCAACTTATTCCGGTAAAAGAGTATTCGGTTATCTAGGTATGATATACGCATTAGCTTCA
ATAGGAATCTTAGGATTCATAGTATGGTCTCATCACATGTTCACTGTAGGTCTAGATGTC
GATACAAGAGCATATTTCACTTCAGCGACATTAATAATTGCAGTGCCAACAGGGATAAAA
ATATTCTCGTGGCTGGCGACGATATACGGGGGAAGTATAAGATTTAGTACTCCTATGTTA
TTTTCTATGGGATTCATATTTTTATTTACAGTAGGAGGATTAACTGGAGTAATATTAGCC
AATGCCTCGTTAGATATAGCTTTACATGATACTTACTACGTGGTAGCACATTTCCATTAT
GTATTATCAATGGGAGCAGTATTTGCATTATTTGCAGCATTCTATTATTGAATAGGTAAA
ATTACTGGAAAACAATATAATGAATTATTAGGGCAGATACATTTTTATACGATGTTTGTC
GGGGTAAATATAACATTCTTCCCAATGCATTTCTTAGGTCTGGCGGGTATGCCACGTAGA
ATACCAGACTATCCAGATGCTTTCATAGGATGGAATTATATAGCATCCTTAGGATCAATA
ATAAGTTTAGTATCAACAGTATTATTCTTATACATAGTCTATGATTTATTAACTAATGAG
GTGGAAGCAAGCGACGGAGCCGATGTAGAAAACGAGTACTTTATATCGAGATGGTCAACT
AAATATGCTCAGACCTTAGAGTGGAGTTTAACAAGCCCACCTGGCTTCCATTGTTATAAT
AGTTTACCAACCTTCCTAACGAAG**TAA**

>cox2-KVL-14-117
ATGTGATTAAATTATATAATGAACGATGCACCAGAGGGGTGACAATTAGGTTTTCAAGAT
AGTGCAAGTCCTATAGCAGAAGGGATATTAAGAATACATAATGAAATACAATATTATTTA
TTAATAATTCTAGTATTAATATGTTGGATAATAACTTCAATAATTTTCAAATTCAATGAA
GAAAGTAATAAATTTAAATCTAAATATATGAATCATGGTACATTAATAGAATTAATTTGA
ACAATAACACCTGCGTTAATCTTAGTCGCCATAGCTATACCTTCATTTAAATTATTATAT
TTAATGGATGAAGTAATAGAACCAACCATTACATTACAAATTACCGGTCACCAATGATAT
TGGTCTACAGAATACTCTGACTATGTAAAACAATCAGGCAATATTGAGTTTGACAGTTAT
ATGATCGCAACCTCGGATTTAGAATTAGGTAAATTAAGATTATTGGAAGTAGATAATAGA
GTAATATTACCAGTAGACACACATGTCAGAGTAATAGTTACGAGTACCGATGTAATACAC
TCACTAGCATTACCATCCTTAGGGGTTAAAATTGACTGTTGCCCAGGTCGATTAAACCAA
ACATCTTTCATAATAAAAAGAGAAGGTGTATTCTACGGACAATGCTCAGAACTTTGCGGA
CATGCACACGGTTTCATGCCGATAGTAATAGAGGGGGTAACATTAGAGGATTACTTAGTA
TGGGTTAATGAGTCGGTAGAAGAA**TAG**
>cox2-KVL-14-118
ATGTGATTAAATTATATAATGAACGATGCACCAGAGGGGTGACAATTAGGTTTTCAAGAT
AGTGCAAGTCCTATAGCAGAAGGGATATTAAGAATACATAATGAAATACAATATTATTTA
TTAATAATTCTAGTATTAATATGTTGGATAATAACTTCAATAATTTTCAAATTCAATGAA
GAAAGTAATAAATTTAAATCTAAATATATGAATCATGGTACATTAATAGAATTAATTTGA
ACAATAACACCTGCGTTAATCTTAGTCGCCATAGCTATACCTTCATTTAAATTATTATAT

TTAATGGATGAAGTAATAGAACCAACCATTACATTACAAATTACCGGTCACCAATGATAT
TGGTCTACAGAATACTCTGACTATGTAAAACAATCAGGCAATATTGAGTTTGACAGTTAT
ATGATCGCAACCTCGGATTTAGAATTAGGTAAATTAAGATTATTGGAAGTAGATAATAGA
GTAATATTACCAGTAGACACACATGTCAGAGTAATAGTTACGAGTACCGATGTAATACAC
TCACTAGCATTACCATCCTTAGGGGTTAAAATTGACTGTTGCCCAGGTCGATTAAACCAA
ACATCTTTCATAATAAAAAGAGAAGGTGTATTCTACGGACAATGCTCAGAACTTTGCGGA
CATGCACACGGTTTCATGCCGATAGTAATAGAGGGGGTAACATTAGAGGATTACTTAGTA
TGGGTTAATGAGTCGGTAGAAGAA**TAG**
>cox2-HHdFL130914-1
ATGTGATTAAATTATATAATGAACGATGCACCAGAGGGGTGACAATTAGGTTTTCAAGAT
AGTGCAAGTCCTATAGCAGAAGGGATATTAAGAATACATAATGAAATACAATATTATTTA
TTAATAATTCTAGTATTAATATGTTGGATAATAACTTCAATAATTTTCAAATTCAATGAA
GAAAGTAATAAATTTAAATCTAAATATATGAATCATGGTACATTAATAGAATTAATTTGA
ACAATAACACCTGCGTTAATCTTAGTCGCCATAGCTATACCTTCATTTAAATTATTATAT
TTAATGGATGAAGTAATAGAACCAACCATTACATTACAAATTACCGGTCACCAATGATAT
TGGTCTACAGAATACTCTGACTATGTAAAACAATCAGGCAATATTGAGTTTGACAGTTAT
ATGATCGCAACCTCGGATTTAGAATTAGGTAAATTAAGATTATTGGAAGTAGATAATAGA
GTAATATTACCAGTAGACACACATGTCAGAGTAATAGTTACGAGTACCGATGTAATACAC
TCACTAGCATTACCATCCTTAGGGGTTAAAATTGACTGTTGCCCAGGTCGATTAAACCAA
ACATCTTTCATAATAAAAAGAGAAGGTGTATTCTACGGACAATGCTCAGAACTTTGCGGA
CATGCACACGGTTTCATGCCGATAGTAATAGAGGGGGTAACATTAGAGGATTACTTAGTA
TGGGTTAATGAGTCGGTAGAAGAA**TAG**
>cox2-Berkeley
ATGTGATTAAATTATATAATGAACGATGCACCAGAGGGGTGACAATTAGGTTTTCAAGAT
AGTGCAAGTCCTATAGCAGAAGGGATATTAAGAATACATAATGAAATACAATATTATTTA
TTAATAATTCTAGTATTAATATGTTGGATAATAACTTCAATAATTTTCAAATTCAATGAA
GAAAGTAATAAATTTAAATCTAAATATATGAATCATGGTACATTAATAGAATTAATTTGA
ACAATAACACCTGCGTTAATCTTAGTCGCCATAGCTATACCTTCATTTAAATTATTATAT
TTAATGGATGAAGTAATAGAACCAACCATTACATTACAAATTACCGGTCACCAATGATAT
TGGTCTACAGAATACTCTGACTATGTAAAACAATCAGGCAATATTGAGTTTGACAGTTAT
ATGATCGCAACCTCGGATTTAGAATTAGGTAAATTAAGATTATTGGAAGTAGATAATAGA
GTAATATTACCAGTAGACACACATGTCAGAGTAATAGTTACGAGTACCGATGTAATACAC
TCACTAGCATTACCATCCTTAGGGGTTAAAATTGACTGTTGCCCAGGTCGATTAAACCAA
ACATCTTTCATAATAAAAAGAGAAGGTGTATTCTACGGACAATGCTCAGAACTTTGCGGA
CATGCACACGGTTTCATGCCGATAGTAATAGAGGGGGTAACATTAGAGGATTACTTAGTA
TGGGTTAATGAGTCGGTAGAAGAA**TAG**
>cox2-HHdFL050913-1
ATGTGATTAAATTATATAATGAACGATGCACCAGAGGGGTGACAATTAGGTTTTCAAGAT
AGTGCAAGTCCTATAGCAGAAGGGATATTAAGAATACATAATGAAATACAATATTATTTA
TTAATAATTCTAGTATTAATATGTTGGATAATAACTTCAATAATTTTCAAATTCAATGAA
GAAAGTAATAAATTTAAATCTAAATATATGAATCATGGTACATTAATAGAATTAATTTGA
ACAATAACACCTGCGTTAATCTTAGTCGCCATAGCTATACCTTCATTTAAATTATTATAT
TTAATGGATGAAGTAATAGAACCAACCATTACATTACAAATTACCGGTCACCAATGATAT
TGGTCTACAGAATACTCTGACTATGTAAAACAATCAGGCAATATTGAGTTTGACAGTTAT

ATGATCGCAACCTCGGATTTAGAATTAGGTAAATTAAGATTATTGGAAGTAGATAATAGA
GTAATATTACCAGTAGACACACATGTCAGAGTAATAGTTACGAGTACCGATGTAATACAC
TCACTAGCATTACCATCCTTAGGGGTTAAAATTGACTGTTGCCCAGGTCGATTAAACCAA
ACATCTTTCATAATAAAAAGAGAAGGTGTATTCTACGGACAATGCTCAGAACTTTGCGGA
CATGCACACGGTTTCATGCCGATAGTAATAGAGGGGGTAACATTAGAGGATTACTTAGTA
TGGGTTAATGAGTCGGTAGAAGAA**TAG**
>cox2-consensus
ATGTGATTAAATTATATAATGAACGATGCACCAGAGGGGTGACAATTAGGTTTTCAAGAT
AGTGCAAGTCCTATAGCAGAAGGGATATTAAGAATACATAATGAAATACAATATTATTTA
TTAATAATTCTAGTATTAATATGTTGGATAATAACTTCAATAATTTTCAAATTCAATGAA
GAAAGTAATAAATTTAAATCTAAATATATGAATCATGGTACATTAATAGAATTAATTTGA
ACAATAACACCTGCGTTAATCTTAGTCGCCATAGCTATACCTTCATTTAAATTATTATAT
TTAATGGATGAAGTAATAGAACCAACCATTACATTACAAATTACCGGTCACCAATGATAT
TGGTCTACAGAATACTCTGACTATGTAAAACAATCAGGCAATATTGAGTTTGACAGTTAT
ATGATCGCAACCTCGGATTTAGAATTAGGTAAATTAAGATTATTGGAAGTAGATAATAGA
GTAATATTACCAGTAGACACACATGTCAGAGTAATAGTTACGAGTACCGATGTAATACAC
TCACTAGCATTACCATCCTTAGGGGTTAAAATTGACTGTTGCCCAGGTCGATTAAACCAA
ACATCTTTCATAATAAAAAGAGAAGGTGTATTCTACGGACAATGCTCAGAACTTTGCGGA
CATGCACACGGTTTCATGCCGATAGTAATAGAGGGGGTAACATTAGAGGATTACTTAGTA
TGGGTTAATGAGTCGGTAGAAGAA**TAG**

>cox3-KVL-14-117
ATGAAGTATAACAAACAATCACATCCTTTTCATTTAGTAGAACCGTCACCTTGACCTTTA
ACCACTTCGATAGGCTTAGGTATAATGGCGATGGGGGGAGTAATATATTTTACGGGTTTA
GATAGTATTGTATTAATATTAGGTTTGGTTGTGACGATAATGACAAGTACATTATGGTGG
AGAGATTGCATTAGAGAGGGTACATTACAAGGTTATCATACAAAAAAAGTAAGAAAAGGT
ATCAATATAGGATTTATATTATTTATAGTCTCTGAAGTATTCTTCTTTTTTTCTATTTTC
TGGGCCTATTTCCACTCTAGTTTATCACCGTCGGTAGAATTAGGTGGTATGTGGCCGCCA
ATGGGGATAGAAGCATTAAACCTTTGAGAGCTACCTTTATTAAATACAGTAATATTGTTA
TCCTCAGGCGCCACAGTGACAACTGCTCATCATGGATTAATCTATGGGGCGAGACGAATA
GTAATATTAAGTTTAATAACAACTTTAATCTTAGCAGTTTTATTTGTAGGGTGCCAAGGA
TATGAATACTATAATGCACCCTTCAGTTTTTCTGATGGAGCGTATGGATCAACTTTCTTC
TTTGCAACAGGTTTCCATGGGTTGCACGTAATAATCGGTACGATATTTTTAGCAGTAGCC
TTTAATAGGGTTATGAATTATGAATTAACTAATCAGCACCACGTTGGTTTTGAATCCGCA
ATCCTGTATTGACATTTCGTAGACATCGTTTGACTGTTTTTATTTATAGTAATCTACGGT
TGAGGGGCG**TAA**
>cox3-KVL-14-118
ATGAAGTATAACAAACAATCACATCCTTTTCATTTAGTAGAACCGTCACCTTGACCTTTA
ACCACTTCGATAGGCTTAGGTATAATGGCGATGGGGGGAGTAATATATTTTACGGGTTTA
GATAGTATTGTATTAATATTAGGTTTGGTTGTGACGATAATGACAAGTACATTATGGTGG
AGAGATTGCATTAGAGAGGGTACATTACAAGGTTATCATACAAAAAAAGTAAGAAAAGGT
ATCAATATAGGATTTATATTATTTATAGTCTCTGAAGTATTCTTCTTTTTTTCTATTTTC
TGGGCCTATTTCCACTCTAGTTTATCACCGTCGGTAGAATTAGGTGGTATGTGGCCGCCA
ATGGGGATAGAAGCATTAAACCTTTGAGAGCTACCTTTATTAAATACAGTAATATTGNNN

NNNNNNNNNNNNNNNNNNNNNNNNNNNNNNNNNNNNNNNNNNNATGGGGCGAGACGAATA
GTAATATTAAGTTTAATAACAACTTTAATCTTAGCAGTTTTATTTGTAGGGTGCCAAGGA
TATGAATACTATAATGCACCCTTCAGTTTTTCTGATGGAGCGTATGGATCAACTTTCTTC
TTTGCAACAGGTTTCCATGGGTTGCACGTAATAATCGGTACGATATTTTTAGCAGTAGCC
TTTAATAGGGTTATGAATTATGAATTAACTAATCAGCACCACGTTGGTTTTGAATCCGCA
ATCCTGTATTGACATTTCGTAGACATCGTTTGACTGTTTTTATTTATAGTAATCTACGGT
TGAGGGGCG**TAA**
>cox2-HHdFL130914-1
ATGAAGTATAACAAACAATCACATCCTTTTCATTTAGTAGAACCGTCACCTTGACCTTTA
ACCACTTCGATAGGCTTAGGTATAATGGCGATGGGGGAGTAATATATTTTACGGGTTTA
GATAGTATTGTATTAATATTAGGTTTGGTTGTGACGATAATGACAAGTACATTATGGTGG
AGAGATTGCATTAGAGAGGGTACATTACAAGGTTATCATACAAAAAAAGTAAGAAAAGGT
ATCAATATAGGATTTATATTATTTATAGTCTCTGAAGTATTCTTCTTTTTTTCTATTTTC
TGGGCCTATTTCCACTCTAGTTTATCACCGTCGGTAGAATTAGGTGGTATGTGGCCGCCA
ATGGGGATAGAAGCATTAAACCTTTGAGAGCTACCTTTATTAAATACAGTAATATTGTTA
TCCTCAGGCGCCACAGTGACAACTGCTCATCATGGATTAATCTATGGGGCGAGACGAATA
GTAATATTAAGTTTAATAACAACTTTAATCTTAGCAGTTTTATTTGTAGGGTGCCAAGGA
TATGAATACTATAATGCACCCTTCAGTTTTTCTGATGGAGCGTATGGATCAACTTTCTTC
TTTGCAACAGGTTTCCATGGGTTGCACGTAATAATCGGTACGATATTTTTAGCAGTAGCC
TTTAATAGGGTTATGAATTATGAATTAACTAATCAGCACCACGTTGGTTTTGAATCCGCA
ATCCTGTATTGACATTTCGTAGACATCGTTTGACTGTTTTTATTTATAGTAATCTACGGT
TGAGGGGCG**TAA**
>cox3-Berkeley
ATGAAGTATAACAAACAATCACATCCTTTTCATTTAGTAGAACCGTCACCTTGACCTTTA
ACCACTTCGATAGGCTTAGGTATAATGGCGATGGGGGAGTAATATATTTTACGGGTTTA
GATAGTATTGTATTAATATTAGGTTTGGTTGTGACGATAATGACAAGTACATTATGGTGG
AGAGATTGCATTAGAGAGGGTACATTACAAGGTTATCATACAAAAAAAGTAAGAAAAGGT
ATCAATATAGGATTTATATTATTTATAGTCTCTGAAGTATTCTTCTTTTTTTCTATTTTC
TGGGCCTATTTCCACTCTAGTTTATCACCGTCGGTAGAATTAGGTGGTATGTGGCCGCCA
ATGGGGATAGAAGCATTAAACCTTTGAGAGCTACCTTTATTAAATACAGTAATATTGTTA
TCCTCAGGCGCCACAGTGACAACTGCTCATCATGGATTAATCTATGGGGCGAGACGAATA
GTAATATTAAGTTTAATAACAACTTTAATCTTAGCAGTTTTATTTGTAGGGTGCCAAGGA
TATGAATACTATAATGCACCCTTCAGTTTTTCTGATGGAGCGTATGGATCAACTTTCTTC
TTTGCAACAGGTTTCCATGGGTTGCACGTAATAATCGGTACGATATTTTTAGCAGTAGCC
TTTAATAGGGTTATGAATTATGAATTAACTAATCAGCACCACGTTGGTTTTGAATCCGCA
ATCCTGTATTGACATTTCGTAGACATCGTTTGACTGTTTTTATTTATAGTAATCTACGGT
TGAGGGGCG**TAA**
>cox3-HHdFL050913-1
ATGAAGTATAACAAACAATCACATCCTTTTCATTTAGTAGAACCGTCACCTTGACCTTTA
ACCACTTCGATAGGCTTAGGTATAATGGCGATGGGGGAGTAATATATTTTACGGGTTTA
GATAGTATTGTATTAATATTAGGTTTGGTTGTGACGATAATGACAAGTACATTATGGTGG
AGAGATTGCATTAGAGAGGGTACATTACAAGGTTATCATACAAAAAAAGTAAGAAAAGGT
ATCAATATAGGATTTATATTATTTATAGTCTCTGAAGTATTCTTCTTTTTTTCTATTTTC
TGGGCCTATTTCCACTCTAGTTTATCACCGTCGGTAGAATTAGGTGGTATGTGGCCGCCA

ATGGGGATAGAAGCATTAAACCTTTGAGAGCTACCTTTATTAAATACAGTAATATTGTTA
TCCTCAGGCGCCACAGTGACAACTGCTCATCATGGATTAATCTATGGGGCGAGACGAATA
GTAATATTAAGTTTAATAACAACTTTAATCTTAGCAGTTTTATTTGTAGGGTGCCAAGGA
TATGAATACTATAATGCACCCTTCAGTTTTTCTGATGGAGCGTATGGATCAACTTTCTTC
TTTGCAACAGGTTTCCATGGGTTGCACGTAATAATCGGTACGATATTTTTAGCAGTAGCC
TTTAATAGGGTTATGAATTATGAATTAACTAATCAGCACCACGTTGGTTTTGAATCCGCA
ATCCTGTATTGACATTTCGTAGACATCGTTTGACTGTTTTTATTTATAGTAATCTACGGT
TGAGGGGCG**TAA**
>cox3-consensus
ATGAAGTATAACAAACAATCACATCCTTTTCATTTAGTAGAACCGTCACCTTGACCTTTA
ACCACTTCGATAGGCTTAGGTATAATGGCGATGGGGGGAGTAATATATTTTACGGGTTTA
GATAGTATTGTATTAATATTAGGTTTGGTTGTGACGATAATGACAAGTACATTATGGTGG
AGAGATTGCATTAGAGAGGGTACATTACAAGGTTATCATACAAAAAAAGTAAGAAAAGGT
ATCAATATAGGATTTATATTATTTATAGTCTCTGAAGTATTCTTCTTTTTTTCTATTTTC
TGGGCCTATTTCCACTCTAGTTTATCACCGTCGGTAGAATTAGGTGGTATGTGGCCGCCA
ATGGGGATAGAAGCATTAAACCTTTGAGAGCTACCTTTATTAAATACAGTAATATTGTTA
TCCTCAGGCGCCACAGTGACAACTGCTCATCATGGATTAATCTATGGGGCGAGACGAATA
GTAATATTAAGTTTAATAACAACTTTAATCTTAGCAGTTTTATTTGTAGGGTGCCAAGGA
TATGAATACTATAATGCACCCTTCAGTTTTTCTGATGGAGCGTATGGATCAACTTTCTTC
TTTGCAACAGGTTTCCATGGGTTGCACGTAATAATCGGTACGATATTTTTAGCAGTAGCC
TTTAATAGGGTTATGAATTATGAATTAACTAATCAGCACCACGTTGGTTTTGAATCCGCA
ATCCTGTATTGACATTTCGTAGACATCGTTTGACTGTTTTTATTTATAGTAATCTACGGT
TGAGGGGCG**TAA**

>nad1-KVL-14-117
ATGATCGAGATACTCTCATTAATAATTCCTTTATTAGTTTCAATAGCTTATTTAACTTTA
GCGGAAAGAAAACTCATGGGTTCAATGCAAAGACGATTAGGTCCAAATAAGGTAGGGATA
CTAGGTCTATTACAGCCATTTGCCGATGGTTTAAAATTAGTGATAAAAGAAACGATTATC
GTATCACAAGCTAATAAAATAATCTTTTTCTTGGCTCCCTATATAACATTGGTATTTGGT
TTCTTGGCTTGAGGTGTCATTCCATTTACTAGAGGGATAATAATAGAGGATATAGGTTAT
AGTACATTATATATATTATTGATAACGGGGATAGGGGTATTAGGGATAATATTAGCAGGT
TGGTCAGCTAATTCTAAATATGCAGTATTAGGGTCGTTAAGGACCACCGCCCAGTTAATA
TCTTACGAGGTAGTAATAGGCTTAATGGTAATAATGGTGGTGATNNNNNNNGATGGGTTG
AGTTTAATATCAATCATAGAAGCACAAAAACATATCTGGAATATAATACCAGTATGGCCT
ATATATATCATATTCATGATATCAGCGATAGCAGAAACAAATCGAGCCCCGATGGACTTA
CCTGAAGCTGAATCAGAGTTAGTGGCAGGGTTTCAAACCGAGCACTCAGCCTTATCTTTT
GCTTATTTCTTTTTAGGTGAGTATGGAATATAATATTAATTTGTACCATCACGGCCAAT
TTCTTTTGGGGGGGTTATAGTATAGGCGGAATTAATTCGGCTTTAATATTAGGCTTAAAA
GTATCGATGCTTTTATTTGTCTTCATCCTCTGCAGGGCTACTTATCCTAGGCTCAGATTT
GACCAATTAATGAGTTTAATGTGAACAGGATTATTGCCACTAGTACTAGGGNNNNNNNNN
NNNNNNNNNNNNNNNNNNNNNNNNNNNNNNNNNNNNNNN
>nad1-KVL-14-118
ATGATCGAGATACTCTCATTAATAATTCCTTTATTAGTTTCAATAGCTTATTTAACTTTA
GCGGAAAGAAAACTCATGGGTTCAATGCAAAGACGATTAGGTCCAAATAAGGTAGGGATA

CTAGGTCTATTACAGCCATTTGCCGNNNNNNNNNNNNNNNNNNNNNNNNNNNNNNNNNNN
NNNNNNNNNNNNNNNNNNNNNNNNNNNNNNNNNNNNNNNNNNNNNNNNNTGGTATTTGGT
TTCTTGGCTTGAGGTGTCATTCCATTTACTAGAGGGATAATAATAGAGGATATAGGTTAT
AGTACATTATATATATTATTGATAACGGGGATAGGGGTATTAGGGATAATATTAGCAGGT
TGGTCAGCTAATTCTAAATATGCAGTATTAGGGTCGTTAAGGACCACCGCCCAGTTAATA
TCTTACGAGGTAGTAATAGGCTTAATGGTAATAATGGTGGTGATGCTAAGTGATGNNNNN
NNNNNNNNNNNNNNNNNNNNNNNNNNNNNNNNNNNNNNNNNNNNNNNNNNNNNNNNNNNN
NNNNNNNTCTTATTCATGATATCAGCGATAGCAGAAACAAATCGAGCCCCGATGGACTTA
CCTGAAGCTGAATCAGAGTTAGTGGCAGGTTTTCAAACCGAGCACTCAGCCTTATCTTTT
GCTTATTTCTTTTTAGGTGAGTATGGGAATATAATATTAATTTGTACCATCACGGCCAAT
TTCTTTTGGGGGGGTTATAGTATAGGCGGAATTAATTCGGCTTTAATATTAGGCTTAAAA
GTATCGATGCTTTTATTTGTCTTCATCCTCTGCAGGGCTACTTATCCTAGGCTCAGATTT
GACCAATTAATGAGTTTAATGTGAACAGGATTATTGCCACTAGTACTAGGGTATTTTGTA
TTAATCTCTAGTATAATGGTTATTATTACCAACTGG**TAG**
>nad1-HHdFL130914-1
ATGATCGAGATACTCTCATTAATAATTCCTTTATTAGTTTCAATAGCTTATTTAACTTTA
GCGGAAAGAAAACTCATGGGTTCAATGCAAAGACGATTAGGTCCAAATAAGGTAGGGATA
CTAGGTCTATTACAGCCATTTGCCGATGGTTTAAAATTAGTGATAAAAGAAACGATTATC
GTATCACAAGCTAATAAAATAATCTTTTTCTTGGCTCCCTATATAACATTGGTATTTGGT
TTCTTGGCTTGAGGTGTCATTCCATTTACTAGAGGGATAATAATAGAGGATATAGGTTAT
AGTACATTATATATATTATTGATAACGGGGATAGGGGTATTAGGGATAATATTAGCAGGT
TGGTCAGCTAATTCTAAATATGCAGTATTAGGGTCGTTAAGGACCACCGCCCAGTTAATA
TCTTACGAGGTAGTAATAGGCTTAATGGTAATAATGGTGGTGATGCTAAGTGATGGGTTG
AGTTTAATATCAATCATAGAAGCACAAAAACATATCTGGAATATAATACCAGTATGGCCT
ATATATATCATATTCATGATATCAGCGATAGCAGAAACAAATCGAGCCCCGATGGACTTA
CCTGAAGCTGAATCAGAGTTAGTGGCAGGTTTTCAAACCGAGCACTCAGCCTTATCTTTT
GCTTATTTCTTTTTAGGTGAGTATGGGAATATAATATTAATTTGTACCATCACGGCCAAT
TTCTTTTGGGGGGGTTATAGTATAGGCAGNNNNNNNNNNNNNNNNNNNNNNNNNNNNNNN
NNNNNNNNNNNNNNNNNNNNNNNNNNNNNNNNNNNNNNNNNNNNNNNNNNNNNNNNNNNN
NNNNNNNNNNNNNNNNNNNNNNNNNNNNNNNNNNNNNNNNNNNNNNNNNNNNNNNNNNNN
NNNNNNNNNNNNNNNNNNNNNNNNNNNNNNNNNNNNNNN
>nad1-Berkeley
ATGATCGAGATACTCTCATTAATAATTCCTTTATTAGTTTCAATAGCTTATTTAACTTTA
GCGGAAAGAAAACTCATGGGTTCAATGCAAAGACGATTAGGTCCAAATAAGGTAGGGATA
CTAGGTCTATTACAGCCATTTGCCGATGGTTTAAAATTAGTGATAAAAGAAACGATTATC
GTATCACAAGCTAATAAAATAATCTTTTTCTTGGCTCCCTATATAACATTGGTATTTGGT
TTCTTGGCTTGAGGTGTCATTCCATTTACTAGAGGGATAATAATAGAGGATATAGGTTAT
AGTACATTATATATATTATTGATAACGGGGATAGGGGTATTAGGGATAATATTAGCAGGT
TGGTCAGCTAATTCTAAATATGCAGTATTAGGGTCGTTAAGGACCACCGCCCAGTTAATA
TCTTACGAGGTAGTAATAGGCTTAATGGTAATAATGGTGGTGATGCTAAGTGATGGGTTG
AGTTTAATATCAATCATAGAAGCACAAAAACATATCTGGAATATAATACCAGTATGGCCT
ATATATATCATATTCATGATATCAGCGATAGCAGAAACAAATCGAGCCCCGATGGACTTA
CCTGAAGCTGAATCAGAGTTAGTGGCAGGTTTTCAAACCGAGCACTCAGCCTTATCTTTT
GCTTATTTCTTTTTAGGTGAGTATGGGAATATAATATTAATTTGTACCATCACGGCCAAT

TTCTTTTGGGGGGGTTATAGTATAGGCGGAATTAATTCGGCTTTAATATTAGGCTTAAAA
GTATCGATGCTTTTATTTGTCTTCATCCTCTGCAGGGCTACTTATCCTAGGCTCAGATTT
GACCAATTAATGAGTTTAATGTGAACAGGATTATTGCCACTAGTACTAGGGTATTTTGTA
TTAATCTCTAGTATAATGGTTATTATTACCAACTGG**TAG**
>nad1-HHdFL050913-1
ATGATCGAGATACTCTCATTAATAATTCCTTTATTAGTTTCAATAGCTTATTTAACTTTA
GCGGAAAGAAAACTCATGGGTTCAATGCAAAGACGATTAGGTCCAAATAAGGTAGGGATA
CTAGGTCTATTACAGCCATTTGCCGATGGTTTAAAATTAGTGATAAAAGAAACGATTATC
GTATCACAAGCTAATAAAATAATCTTTTTCTTGGCTCCCTATATAACATTGGTATTTGGT
TTCTTGGCTTGAGGTGTCATTCCATNNNNNNNNNNNNNNNNNNNNNNNNNNNNNNNNNNN
NNNNNNNNNNNTATATTATTGATAACGGGGATAGGGGTATTAGGGATAATATTAGCAGGT
TGGTCAGCTAATTCTAAATATGCAGTATTAGGGTCGTTAAGGACCACCGCCCAGTTAATA
TCTTACGAGGTAGTAATAGGCTTAATGGTAATAATGGTGGTGATGCTAAGTGATGGGTTG
AGTTTAATATCAATCATAGAAGCACAAAAACATATCTGGAATATAATACCAGTATGGCCT
ATATATATCATATTCATGATATCAGCGATAGCAGAAACAAATCGAGCCCCGATGGACTTA
CCTGAAGCTGAATCAGAGTTAGTGGCAGGTTTTCAAACCGAGCACTCAGCCTTATCTTTT
GCTTATTTCTTTTTAGGTGAGTATGGAATATAATATTAATTTGTACCATCACGGCCAAT
TTCTTTTGGGGGGGTTATAGTATAGGCGGAATTAATTCGGCTTTAATATTAGGCTTAAAA
GTATCGATGCTTTTATTTGTCTTCATCCTCTGCAGGGCTACTTATCCTAGGCTCAGATTT
GACCAATTAATGAGTTTAATGTGAACAGGATTATTGCCACTAGTACTAGGGTATTTTGTA
TTAATCTCTAGTATAATGGTTATTATTACCAACTGG**TAG**
>nad1-consensus
ATGATCGAGATACTCTCATTAATAATTCCTTTATTAGTTTCAATAGCTTATTTAACTTTA
GCGGAAAGAAAACTCATGGGTTCAATGCAAAGACGATTAGGTCCAAATAAGGTAGGGATA
CTAGGTCTATTACAGCCATTTGCCGATGGTTTAAAATTAGTGATAAAAGAAACGATTATC
GTATCACAAGCTAATAAAATAATCTTTTTCTTGGCTCCCTATATAACATTGGTATTTGGT
TTCTTGGCTTGAGGTGTCATTCCATTTACTAGAGGGATAATAATAGAGGATATAGGTTAT
AGTACATTATATATATTATTGATAACGGGGATAGGGGTATTAGGGATAATATTAGCAGGT
TGGTCAGCTAATTCTAAATATGCAGTATTAGGGTCGTTAAGGACCACCGCCCAGTTAATA
TCTTACGAGGTAGTAATAGGCTTAATGGTAATAATGGTGGTGATGCTAAGTGATGGGTTG
AGTTTAATATCAATCATAGAAGCACAAAAACATATCTGGAATATAATACCAGTATGGCCT
ATATATATCATATTCATGATATCAGCGATAGCAGAAACAAATCGAGCCCCGATGGACTTA
CCTGAAGCTGAATCAGAGTTAGTGGCAGGTTTTCAAACCGAGCACTCAGCCTTATCTTTT
GCTTATTTCTTTTTAGGTGAGTATGGAATATAATATTAATTTGTACCATCACGGCCAAT
TTCTTTTGGGGGGGTTATAGTATAGGCGGAATTAATTCGGCTTTAATATTAGGCTTAAAA
GTATCGATGCTTTTATTTGTCTTCATCCTCTGCAGGGCTACTTATCCTAGGCTCAGATTT
GACCAATTAATGAGTTTAATGTGAACAGGATTATTGCCACTAGTACTAGGGTATTTTGTA
TTAATCTCTAGTATAATGGTTATTATTACCAACTGG**TAG**

>nad2-KVL-14-117
ATGTTAACTTTAAGTATAATAATATTAATAACAAGTTTAGGGAGATGAATAAATATAAAT
AGAATGACGATGATAGTATTTATATATTCAGGAATTTTAAGTATAAATATGACGGGGGAG
AGCAATAGCGCCATAGGGTGTTCCGATATGGGGATGATAAATTCAGGGATAGGGCTATAT
AATGGATTATATCAAGTGACACCAATAACTCAATTCATAGAAACTTATATAAATATATTA

GCGGTAGGCTGTATGGCGATGAAGGCTAGGAGTGAATATAGTATATTAGCCTTAATAGTG
ACGGTAGGGATGAGTAGTTTAATAAGTAGTAATGAGATAGTTAGTATATTAATAGGGATA
GAGTTACAGACATTAGGATTATATGTCATAGCCAGTCTAGATAGAGGTTCAGAGACCTCG
ACGGCAGCGGGGTTAAAATATTATTTATTAGGGGGTTTATCGTCATGTATAATAGGTTTA
GGTTTGAGTATGATATATGGGATAACAGGGGTGACAAATATAGAGGAGTTAAATATATTA
ATAAAAATAAATGACGACAGCCGTCATCTAATAATGACGAAGGTATTAATTACAGTAGGA
TTATTATTTAAAATAGGAGCAGCTCCCTTTCATAATTGGTTAGCGGATGTAATAGATGGA
GTACCGACTGCGATATCGGCATGGTTAGCGGTGGTATCAAAGATATCAATAATAATAATA
TTATTAATATTATATAATGCTATATTAATAAAAATAAACACCAGTGGTGGTGGTCCGATA
ATAATATTAAGTATAATATTATGCTTTATAGTAGGAAGTTTAGTAGGGATAATACAATCT
AGGATAAAAACATTATTAGCTTATAGTAGTATAGCCCATGCGGGGTATATATTAATAGGG
ATAATAGTAAATAATAATCTAGGATTAACAGGTTTAATATTTTATATGGTACAATATTCT
TTAACGGCATTAAATATATTTATGATAATAATAGCCTATGGTGAGGTAAAAGGGGCATCG
ATAGTAAAAATATCACAATTAAAAGAAATCCCTAACACGTGATTAGCGATAAGTTTAGCG
ATAAGTTTATTTTCATCTGCAGGGATTCCACCGTTTATAGGATTTTATGCCAAACTTAAT
ATATTATACTCGGCTCTAGATACAGGTTTTTACTTCATAACAATAATCGCCATACTAACC
AGTATAATAAGTGGTTATTATTATATAAAAGTAATAAAAGTAATATATTTTGAGGGGGAG
TGATGTAGGGTTGGAACCGCCAATGAAGTACGGGGGGCGATATCAGAAAATTATTCAATA
ATAATAGCCATGATAAGTTTAGTGATAATATTATTCATCCTCAGTCCCGAGTGACTATAT
AATAGTATTCAGATGATATGC**TAG**
>nad2-KVL-14-118
ATGTTAACYTTAAGTATAATAATATTAATAACAAGTTTAGGGAGATGAATAAATATAAAT
AGAATGACGATGATAGTATTTATATATTCAGGAATTTTAAGTATAAATATGACGGGGGAG
AGCAATAGCGCCATAGGGTGTTCCGATATGGGGATGATAAATTCAGGGATAGGGCTATAT
AATGGATTATATCAAGTGACACCAATAACTCAATTCATAGAAACTTATATAAATATATTA
GCGGTAGGCTGTATGGCGATGAAGGCTAGGAGTGAATATAGTATATTAGCCTTAATAGTG
ACGGTAGGGATGAGTAGTTTAATAAGTAGTAATGAGATAGTTAGTATATTAATAGGGATA
GAGTTACAGACATTAGGATTATATGTCATAGCCAGTCTAGATAGAGGTTCAGAGACCTCG
ACGGCAGCGGGGTTAAAATATTATTTATTAGGGGGTTTATCGTCATGTATAATAGGTTTA
GGTTTGAGTATGATATATGGGATAACAGGGGTGACAAATATAGAGGAGTTAAATATATTA
ATAAAAATAAATGACGACAGCCGTCATCTAATAATGACGAAGGTATTAATTACAGTAGGA
TTATTATTTAAAATAGGAGCAGCTCCCTTTCATAATTGGTTAGCGGATGTAATAGATGGA
GTACCGACTGCGATATCGGCATGGTTAGCGGNNNNNNNNNNNNNNNNNNTAATAATAATA
TTATTAATATTATATAATGCTATATTAATAAAAATAAACACCAGTGGTGGTGGTCCGATA
ATAATATTAAGTATAATATTATGCTTTATAGTAGGAAGTTTAGTAGGGATAATACAATCT
AGGATAAAAACATTATTAGCTTATAGTAGTATAGCCCATGCGGGGTATATATTAATAGGG
ATAATAGTAAATAATAATCTAGGATTAACAGGTTTAATATTTTATATGGTACAATATTCT
TTAACGGCATTAAATATATTTATGATAATAATAGCCTATGGTGAGGTAAAAGGGGCATCG
ATAGTAAAAATATCACAATTAAAAGAAATCCCTAACACGTGATTAGCGATAAGTTTAGCG
ATAAGTTTATTTTCATCTGCAGGGATTCCACCGTTTATAGGATTTTATGCCAAACTTAAT
ATATTATACTCGGCTCTAGATACAGGTTTTTACTTCATAACAATAATCGCCATACTAACC
AGTATAATAAGTGGTTATTATTATATAAAAGTAATAAAAGTAATATATTTTGAGGGGGAG
TGATGTAGGGTTGGAACCGCCAATGAAGTACGGGGGGCGATATCAGAAAATTATTCAATA
ATAATAGCCATGATAAGTTTAGTGATAATATTATTCATCCTCAGTCCCGAGTGACTATAT

AATAGTATTCAGATGATATGC**TAG**
>nad2-HHdFL130914-1
ATGTTAACTTTAAGTATAATAATATTAATAACAAGTTTAGGGAGATGAATAAATATAAAT
AGAATGACGATGATAGTATTTATATATTCAGGAATTTTAAGTATAAATATGACGGGGGAG
AGCAATAGCGCCATAGGGTGTTCCGATATGGGGATGATAAATTCAGGGATAGGGCTATAT
AATGGATTATATCAAGTGACACCAATAACTCAATTCATAGAAACTTATATAAATATATTA
GCGGTAGGCTGTATGGCGATGAAGGCTAGGAGTGAATATAGTATATTAGCCTTAATAGTG
ACGGTAGGGATGAGTAGTTTAATAAGTAGTAATGAGATAGTTAGTATATTAATAGGGATA
GAGTTACAGACATTAGGATTATATGTCATAGCCAGTCTAGATAGAGGTTCAGAGACCTCG
ACGGCAGCGGGGTTAAAATATTATTTATTAGGGGGTTTATCGTCATGTATAATAGGTTTA
GGTTTGAGTATGATATATGGGATAACAGGGGTGACAAATATAGAGGAGTTAAATATATTA
ATAAAAATAAATGACGACAGCCGTCATCTAATAATGACGAAGGTATTAATTACAGTAGGA
TTATTATTTAAAATAGGAGCAGCTCCCTTTCATAATTGGTTAGCGGATGTAATAGATGGA
GTACCGACTGCGATATCGGCATGGTTAGCGGTGGTATCAAAGATATCAATAATAATAATA
TTATTAATATTATATAATGCTATATTAATAAAAATAAACACCAGTGGTGGTGGTCCGATA
ATAATATTAAGTATAATATTATGCTTTATAGTAGGAAGTTTAGTAGGGATAATACAATCC
AGGATAAAAACATTATTAGCTTATAGTAGTATAGCCCATGCGGTGTATATATTAATAGGG
ATAATAGTAAATAATAATCTAGGATTAACAGGTTTAATATTTTATATGGTACAATATTCT
TTAACGGCATTAAATATATTTATGATAATAATAGCCTATGGTGAGGTAAAAGGGGCATCG
ATAGTAAAAATATCACAATTAAAAGAAATCCCTAACACGTGATTAGCGATAAGTTTAGCG
ATAAGTTTATTTTCATCTGCAGGGATTCCACCGTTTATAGGATTTTATGCCAAACTTAAT
ATATTATACTCGGCTCTAGATACAGGTTTTTACTTCATAACAATAATCGCCATACTAACC
AGTATAATAAGTGGTTATTATTATATAAAAGTAATAAAAGTAATATATTTTGAGGGGGAG
TGATGTAGGGTTGGAACCGCCAATGAAGTACGGGGGGCGATATCAGAAAATTATTCAATA
ATAATAGCCATGATAAGTTTAGTGATAATATTATTCATCCTCAGTCCCGAGTGACTATAT
AATAGTATTCAGATGATATGC**TAG**
>nad2-Berkeley
ATGTTAACTTTAAGTATAATAATATTAATAACAAGTTTAGGGAGATGAATAAATATAAAT
AGAATGACGATGATAGTATTTATATATTCAGGAATTTTAAGTATAAATATGACGGGGGAG
AGCAATAGCGCCATAGGGTGTTCCGATATGGGGATGATAAATTCAGGGATAGGGCTATAT
AATGGATTATATCAAGTGACACCAATAACTCAATTCATAGAAACTTATATAAATATATTA
GCGGTAGGCTGTATGGCGATGAAGGCTAGGAGTGAATATAGTATATTAGCCTTAATAGTG
ACGGTAGGGATGAGTAGTTTAATAAGTAGTAATGAGATAGTTAGTATATTAATAGGGATA
GAGTTACAGACATTAGGATTATATGTCATAGCCAGTCTAGATAGAGGTTCAGAGACCTCG
ACGGCAGCGGGGTTAAAATATTATTTATTAGGGGGTTTATCGTCATGTATAATAGGTTTA
GGTTTGAGTATGATATATGGGATAACAGGGGTGACAAATATAGAGGAGTTAAATATATTA
ATAAAAATAAATGACGACAGCCGTCATCTAATAATGACGAAGGTATTAATTACAGTAGGA
TTATTATTTAAAATAGGAGCAGCTCCCTTTCATAATTGGTTAGCGGATGTAATAGATGGA
GTACCGACTGCGATATCGGCATGGTTAGCGGTGGTATCAAAGATATCAATAATAATAATA
TTATTAATATTATATAATGCTATATTAATAAAAATAAACACCAGTGGTGGTGGTCCGATA
ATAATATTAAGTATAATATTATGCTTTATAGTAGGAAGTTTAGTAGGGATAATACAATCT
AGGATAAAAACATTATTAGCTTATAGTAGTATAGCCCATGCGGGGTATATATTAATAGGG
ATAATAGTAAATAATAATCTAGGATTAACAGGTTTAATATTTTATATGGTACAATATTCT
TTAACGGCATTAAATATATTTATGATAATAATAGCCTATGGTGAGGTAAAAGGGGCATCG

ATAGTAAAAATATCACAATTAAAAGAAATCCCTAACACGTGATTAGCGATAAGTTTAGCG
ATAAGTTTATTTTCATCTGCAGGGATTCCACCGTTTATAGGATTTTATGCCAAACTTAAT
ATATTATACTCGGCTCTAGATACAGGTTTTTACTTCATAACAATAATCGCCATACTAACC
AGTATAATAAGTGGTTATTATTATATAAAAGTAATAAAAGTAATATATTTTGAGGGGGAG
TGATGTAGGGTTGGAACCGCCAATGAAGTACGGGGGGCGATATCAGAAAATTATTCAATA
ATAATAGCCATGATAAGTTTAGTGATAATATTATTCATCCTCAGTCCCGAGTGACTATAT
AATAGTATTCAGATGATATGC**TAG**
>nad2-HHdFL050913-1
NNNNNNNNNNNNNNNNNNNNNNNNNNNNNAACAAGTTTAGGGAGATGAATAAATATAAAT
AGAATGACGATGATAGTATTTATATATTCAGGAATTTTAAGTATAAATATGACGGGGGAG
AGCAATAGCGCCATAGGGTGTTCCGATATGGGGATGATAAATTCAGGGATAGGGCTATAT
AATGGATTATATCAAGTGACACCAATTACTCAATTCATAGAAACTTATATAAATATATTA
GCGGTAGGCTGTATGGCGATGAAGGCTAGGAGTGAATATAGTATATTAGCCTTAATAGTG
ACGGTAGGGATGAGTAGTTTAATAAGTAGTAATGAGATAGTTAGTATATTAATAGGGATA
GAGTTACAGACATTAGGATTATATGTCATAGCCAGTCTAGATAGAGGTTCAGAGACCTCG
ACGGCAGCGGGGTTAAAATATTATTTATTAGGGGGTTTATCGTCATGTATAATAGGTTTA
GGTTTGAGTATGATATATGGGATAACAGGGGTGACAAATATAGAGGAGTTAAATATATTA
ATAAAAATAAATGACGACAGCCGTCATCTAATAATGACGAAGGTATTAATTACAGTAGGA
TTATTATTTAAAATAGGAGCAGCTCCCTTTCATAATTGGTTAGCGGATGTAATAGATGGA
GTACCGACTGCGATATCGGCATGGTTAGCGGTGGTCTCAAAGNTATCAATAATAATAATA
TTATTAATATTATATAATGCTATATTAATAAAAATAAACACCAGTGGTGGTGGTCCGATA
ATAATATTAAGTATAATATTATGCTTTATAGTAGGAAGTTTAGTAGGGATAATACAATCT
AGGATAAAAACATTATTAGCTTATAGTAGTATAGCCCATGCGGGGTATATATTAATAGGG
ATAATAGTAAATAATAATCTAGGATTAACAGGTTTAATATTTTATATGGTACAATATTCT
TTAACGGCATTAAATATATTTATGATAATAATAGCCTATGGTGAGGTAAAAGGGGCATCG
ATAGTAAAAATATCACAATTAAAAGAAATCCCTAACACGTGATTAGCGATAAGTTTAGCG
ATAAGTTTATTTTCATCTGCAGGGATTCCACCGTTTATAGGATTTTATGCCAAACTTAAT
ATATTATACTCGGCTCTAGATACAGGTTTTTACTTCATAACAATAATCGCCATACTAACC
AGTATAATAAGTGGTTATTATTATATAAAAGTAATAAAAGTAATATATTTTGAGGGGGAG
TGATGTAGGGTTGGAACCGCCAATGAAGTACGGGGGGCGATATCAGAAAATTATTCAATA
ATAATAGCCATGATAAGTTTAGTGATAATATTATTCATCCTCAGTCCCGAGTGACTATAT
AATAGTATTCAGATGATATGC**TAG**
>nad2-consensus
ATGTTAACTTTAAGTATAATAATATTAATAACAAGTTTAGGGAGATGAATAAATATAAAT
AGAATGACGATGATAGTATTTATATATTCAGGAATTTTAAGTATAAATATGACGGGGGAG
AGCAATAGCGCCATAGGGTGTTCCGATATGGGGATGATAAATTCAGGGATAGGGCTATAT
AATGGATTATATCAAGTGACACCAATAACTCAATTCATAGAAACTTATATAAATATATTA
GCGGTAGGCTGTATGGCGATGAAGGCTAGGAGTGAATATAGTATATTAGCCTTAATAGTG
ACGGTAGGGATGAGTAGTTTAATAAGTAGTAATGAGATAGTTAGTATATTAATAGGGATA
GAGTTACAGACATTAGGATTATATGTCATAGCCAGTCTAGATAGAGGTTCAGAGACCTCG
ACGGCAGCGGGGTTAAAATATTATTTATTAGGGGGTTTATCGTCATGTATAATAGGTTTA
GGTTTGAGTATGATATATGGGATAACAGGGGTGACAAATATAGAGGAGTTAAATATATTA
ATAAAAATAAATGACGACAGCCGTCATCTAATAATGACGAAGGTATTAATTACAGTAGGA
TTATTATTTAAAATAGGAGCAGCTCCCTTTCATAATTGGTTAGCGGATGTAATAGATGGA

GTACCGACTGCGATATCGGCATGGTTAGCGGTGGTATCAAAGATATCAATAATAATAATA
TTATTAATATTATATAATGCTATATTAATAAAAATAAACACCAGTGGTGGTGGTCCGATA
ATAATATTAAGTATAATATTATGCTTTATAGTAGGAAGTTTAGTAGGGATAATACAATCT
AGGATAAAAACATTATTAGCTTATAGTAGTATAGCCCATGCGGGGTATATATTAATAGGG
ATAATAGTAAATAATAATCTAGGATTAACAGGTTTAATATTTTATATGGTACAATATTCT
TTAACGGCATTAAATATATTTATGATAATAATAGCCTATGGTGAGGTAAAAGGGGCATCG
ATAGTAAAAATATCACAATTAAAAGAAATCCCTAACACGTGATTAGCGATAAGTTTAGCG
ATAAGTTTATTTTCATCTGCAGGGATTCCACCGTTTATAGGATTTTATGCCAAACTTAAT
ATATTATACTCGGCTCTAGATACAGGTTTTTACTTCATAACAATAATCGCCATACTAACC
AGTATAATAAGTGGTTATTATTATATAAAAGTAATAAAAGTAATATATTTTGAGGGGGAG
TGATGTAGGGTTGGAACCGCCAATGAAGTACGGGGGGCGATATCAGAAAATTATTCAATA
ATAATAGCCATGATAAGTTTAGTGATAATATTATTCATCCTCAGTCCCGAGTGACTATAT
AATAGTATTCAGATGATATGC**TAG**

>nad3-KVL-14-117
ATGAATAATTTATTTATCTTAATAATCTCAGCCACCGCCATTGTATTCTTATTATTATTT
GTCAATCTTATTGTCGGGGATAAAAAACCTTATACTAATAAATTATCTCCCTATGAATGT
GGTTTAATGCCTTTAGGTGAAGGGAGAGCTAGCTTAAATATACAATATATTTTAGTGGCT
ATTTTATTTATAATATTTGATATTGAGGTAATAGTATTATTTCCATATGCAGTGACTATA
AATAATATATACACATACTGGATAATGATTATCTTTATCATCATTTTAACCATAGGGTTT
TACTTTGAAATTAGTCAAGGGGCTTTAAAATATGTCGATGAAGAGGTT**TAG**
>nad3-KVL-14-118
ATGAATAATTTATTTATCTTAATAATCTCAGCCACCGCCATTGTATTCTTATTATTATTT
GTCAATCTTATTGTCGGGGATAAAAAACCTTATACTAATAAATTATCTCCCTATGAATGT
GGTTTAATGCCTTTAGGTGAAGGGAGAGCTAGCTTAAATATACAATATATTTTAGTGGCT
ATTTTATTTATAATATTTGATATTGAGGTAATAGTATTATTTCCATATGCAGTGACTATA
AATAATATATACACATACTGGATAATGATTATCTTTATCATCATTTTAACCATAGGGTTT
TACTTTGAAATTAGTCAAGGGGCTTTAAAATATGTCGATGAAGAGGTT**TAG**
>nad3-HHdFL130914-1
ATGAATAATTTATTTATCTTAATAATCTCAGCCACCGCCATTGTATTCTTATTATTATTT
GTCAATCTTATTGTCGGGGATAAAAAACCTTATACTAATAAATTATCTCCCTATGAATGT
GGTTTAATGCCTTTAGGTGAAGGGAGAGCTAGCTTAAATATACAATATATTTTAGTGGCT
ATTTTATTTATAATATTTGATATTGAGGTAATAGTATTATTTCCATATGCAGTGACTATA
AATAATATATACACATACTGGATAATGATTATCTTTATCATCATTTTAACCATAGGGTTT
TACTTTGAAATTAGTCAAGGGGCTTTAAAATATGTCGATGAAGAGGTT**TAG**
>nad3-Berkeley
ATGAATAATTTATTTATCTTAATAATCTCAGCCACCGCCATTGTATTCTTATTATTATTT
GTCAATCTTATTGTCGGGGATAAAAAACCTTATACTAATAAATTATCTCCCTATGAATGT
GGTTTAATGCCTTTAGGTGAAGGGAGAGCTAGCTTAAATATACAATATATTTTAGTGGCT
ATTTTATTTATAATATTTGATATTGAGGTAATAGTATTATTTCCATATGCAGTGACTATA
AATAATATATACACATACTGGATAATGATTATCTTTATCATCATTTTAACCATAGGGTTT
TACTTTGAAATTAGTCAAGGGGCTTTAAAATATGTCGATGAAGAGGTT**TAG**
>nad3-HHdFL050913-1
ATGAATAATTTATTTATCTTAATAATCTCAGCCACCGCCATTGTATTCTTATTATTATTT

GTCAATCTTATTGTCGGGGATAAAAAACCTTATACTAATAAATTATCTCCCTATGAATGT
GGTTTAATGCCTTTAGGTGAAGGGAGAGCTAGCTTAAATATACAATATATTTTAGTGGCT
ATTTTATTTATAATATTTGATATTGAGGTAATAGTATTATTTCCATATGCAGTGACTATA
AATAATATATACACATACTGGATAATGATTATCTTTATCATCATTTTAACCATAGGGTTT
TACTTTGAAATTAGTCAAGGGGCTTTAAAATATGTCGATGAAGAGGTT**TAG**
>nad3-consensus
ATGAATAATTTATTTATCTTAATAATCTCAGCCACCGCCATTGTATTCTTATTATTATTT
GTCAATCTTATTGTCGGGGATAAAAAACCTTATACTAATAAATTATCTCCCTATGAATGT
GGTTTAATGCCTTTAGGTGAAGGGAGAGCTAGCTTAAATATACAATATATTTTAGTGGCT
ATTTTATTTATAATATTTGATATTGAGGTAATAGTATTATTTCCATATGCAGTGACTATA
AATAATATATACACATACTGGATAATGATTATCTTTATCATCATTTTAACCATAGGGTTT
TACTTTGAAATTAGTCAAGGGGCTTTAAAATATGTCGATGAAGAGGTT**TAG**

>nad4-KVL-14-117
ATGTTAAGTATAATAGCAATAATACCCTTGATAGGTTTATGAATAATAATGATGAGCAGA
GATGAAAAACAGTATAAAATTATTGGATTAGTAACATCATTACTAAACTTAGTAATATAT
CCAATAATATGGGCGCAACACAATTCAAATAATAATTACTTTCAGATTATAGAGAAAACG
TCTTCACCCCTTGACACTTATTATCCAGAAATAATCGGGGTAGATAGTATATCATTATAT
ATGATAGGGATAACAATAATATTAATACCAATATGTATATTATCAACGTGATCGAGTGTG
AAAAAGGAAGTAAAGTTATATATAGGATTATGATTAGTATTAGAAACCGTCTTAATATTA
GTATTTCTATGAATAGACATACTATTATTTTATATAACCTTTGAAACAAGTTTAATACCA
ATGTATTTAATAATAGGGATATATGGGGGTAAAAAAAGGAAGATATATGCAGCATACCAA
TTTTTCTTAATAACATTATTAGGCTCATTATTGATGTTAATGGGGATAATAATGTTATAT
TCACAAATAGGGGTAACAGATTATCAAATATACACATTAACAGTGGAGCTAACAAAAGAA
AGAGAAAAAATAATATGGTTAGCTTTATTTATATCATTTGCGGTGAAAACACCATTAGTA
CCGGTTCATATTTGGTTACCTGAAGCTCATAGTGAAGCAAATATAGCCGGTTCAATAATA
TTAGCGGGTGTATTATTAAAATTAGCCGGCTATGGCTTTCTGAGATATTCACTGAATATA
TTACCTGAGGCATCAAGATATTATATTCCCTTAGTATATGGTTTATCTATAATAAGTATC
ATTTATTGTAGTTTAACCACATTAAGACAAATAGATATGAAAAAAATCATAGCATATTCA
TCCATAGGTCATATGGGGATAGTAATATTAGGTATATTTTCCAATACGATGGAGGGGCTA
GAGGGGTCGATGATATTAATGATAGGTCATGGATTGGTATCACCGGGGTTATTTATAATA
GTAACGATATTATATGAAAGATATCATAGTCGAATAATAAAATATTATAGGGGGGTGACG
TCGACAAGTCCATTAATAGCGATATTATTTATAATATATACCTTAGCCAATATGGGTGTA
CCTTTATCGAGTAATTTTATAGGGGAAATATTATGTTTAATGGGTGCTTGGGAAACCAAT
CCGATATCGACGATAATAGCGTCAACCGGGTTAATATTAGGGGGGGCTTATAGTATATGG
TTTTATAATAGAATAAGCTTTGGGGAACCCTCAATATATTATAGGGAGCTAGATATAAAC
CGAAGGGAGTTTGAAGAACTATTACCTCTATTAATATTGATAATAATCATAGGTGTGTTT
CCGAATCTGATATTAGACACTTTACATACAGCGGTGAGTAATATATTTTGGGCCGCCGGG
TAG
>nad4-KVL-14-118
ATGTTAAGTATAATAGCAATAATACCCTTGATAGGTTTATGAATAATAATGATGAGCAGA
GATGAAAAACAGTATAAAATTATTGGATTAGTAACATCATTACTAAACTTAGTAATATAT
CCAATAATATGGGCGCAACACAATTCAAATAATAATTACTTTCAGATTATAGAGAAAACG
TCTTCACCCCTTGACACTTATTATCCAGAAATAATCGGGGTAGATAGTATATCATTATAT

ATGATAGGGATAACAATAATATTAATACCAATATGTATATTATCAACGTGATCGAGTGTG
AAAAAGGAAGTAAAGTTATATATAGGATTATGATTAGTATTAGAAACCGTCTTAATATTA
GTATTTCTATGAATAGACATACTATTATTTTATATAACCTTTGAAACAAGTTTAATACCA
ATGTATTTAATAATAGGGATATATGGGGGTAAAAAAAGGAAGATATATGCAGCATACCAA
TTTTTCTTAATAACATTATTAGGCTCATTATTGATGTTAATGGGGATAATAATGTTATAT
TCACAAATAGGGGTAACAGATTATCAAATATACACATTAACAGTGGAGCTAACAAAAGAA
AGAGAAAAAATAATATGGTTAGCTTTATTTATATCATTTGCGGTGAAAACACCATTAGTA
CCGGTTCATATTTGGTTACCTGAAGCTCATAGTGAAGCAAATATAGCCGGTTCAATAATA
TTAGCGGGTGTATTATTAAAATTAGCCGGCTATGGCTTTCTGAGATATTCACTGAATATA
TTACCTGAGGCATCAAGATATTATATTCCCTTAGTATATGGTTTATCTATAATAAGTATC
ATTTATTGTAGTTTAACCACATTAAGACAAATAGATATGAAAAAAATCATAGCATATTCA
TCCATAGGTCATATGGGGATAGTAATATTAGGTATATTTTCCAATACGATGGAGGGGCTA
GAGGGGTCGATGATATTAATGATAGGTCATGGATTGGTATCACCGGGGTTATTTATAATA
GTAACGATATTATATGAAAGATATCATAGTCGAATAATAAAATATTATAGGGGGGTGACG
TCGACAAGTCCATTAATAGCGATATTATTTATAATATATACCTTAGCCAATATGGGTGTA
CCTTTATCGAGTAATTTTATAGGGGAAATATTATGTTTAATGGGTGCTTGGGAAACCAAT
CCGATATCGACGATAATAGCGTCAACCGGGTTAATATTAGGGGGGGCTTATAGTATATGG
TTTTATAATAGAATAAGCTTTGGGGAACCCTCAATATATTATAGGGAGCTAGATATAAAC
CGAAGGGAGTTTGAAGAACTATTACCTCTATTAATATTGATAATAATCATAGGTGTGTTT
CCGAATCTGATATTAGACACTTTACATACAGCGGTGAGTAATATATTTTGGGCCGCCGGG
TAG
>nad4-HHdFL130914-1
ATGTTAAGTATAATAGCAATAATACCCTTGATAGGTTTATGAATAATAATGATGAGCAGA
GATGAAAAACAGTATAAAATTATTGGATTAGTAACATCATTACTAAACTTAGTAATATAT
CCAATAATATGGGCGCAACACAATTCAAATAATAATTACTTTCAGATTATAGAGAAAACG
TCTTCACCCCTTGACACTTATTATCCAGAAATAATCGGGGTAGATAGTATATCATTATAT
ATGATAGGGATAACAATAATATTAATACCAATATGTATATTATCAACGTGATCGAGTGTG
AAAAAGGAAGTAAAGTTATATATAGGATTATGATTAGTATTAGAAACCGTCTTAATATTA
GTATTTCTATGAATAGACATACTATTATTTTATATAACCTTTGAAACAAGTTTAATACCA
ATGTATTTAATAATAGGGATATATGGGGGTAAAAAAAGGAAGATATATGCAGCATACCAA
TTTTTCTTAATAACATTATTAGGCTCATTATTGATGTTAATGGGGATAATAATGTTATAT
TCACAAATAGGGGTAACAGATTATCAAATATACACATTAACAGTGGAGCTAACAAAAGAA
AGAGAAAAAATAATATGGTTAGCTTTATTTATATCATTTGCGGTGAAAACACCATTAGTA
CCGGTTCATATTTGGTTACCTGAAGCTCATAGTGAAGCAAATATAGCCGGTTCAATAATA
TTAGCGGGTGTATTATTAAAATTAGCCGGCTATGGCTTTCTGAGATATTCACTGAATATA
TTACCTGAGGCATCAAGATATTATATTCCCTTAGTATATGGTTTATCTATAATAAGTATC
ATTTATTGTAGTTTAACCACATTAAGACAAATAGATATGAAAAAAATCATAGCATATTCA
TCCATAGGTCATATGGGGATAGTAATATTAGGTATATTTTCCAATACGATGGAGGGGCTA
GAGGGGTCGATGATATTAATGATAGGTCATGGATTGGTATCACCGGGGTTATTTATAATA
GTAACGATATTATATGAAAGATATCATAGTCGAATAATAAAATATTATAGGGGGGTGACG
TCGACAAGTCCATTAATAGCGATATTATTTATAATATATACCTTAGCCAATATGGGTGTA
CCTTTATCGAGTAATTTTATAGGGGAAATATTATGTTTAATGGGTGCTTGGGAAACCAAT
CCGATATCGACGATAATAGCGTCAACCGGGTTAATATTAGGGGGGGCTTATAGTATATGG
TTTTATAATAGAATAAGCTTTGGGGAACCCTCAATATATTATAGGGAGCTAGATATAAAC

CGAAGGGAGTTTGAAGAACTATTACCTCTATTAATATTGATAATAATCATAGGTGTGTTT
CCGAATCTGATATTAGACACTTTACATACAGCGGTGAGTAATATATTTTGGGCCGCCGGG
TAG
>nad4-Berkeley
ATGTTAAGTATAATAGCAATAATACCCTTGATAGGTTTATGAATAATAATGATGAGCAGA
GATGAAAAACAGTATAAAATTATTGGATTAGTAACATCATTACTAAACTTAGTAATATAT
CCAATAATATGGGCGCAACACAATTCAAATAATAATTACTTTCAGATTATAGAGAAAACG
TCTTCACCCCTTGACACTTATTATCCAGAAATAATCGGGGTAGATAGTATATCATTATAT
ATGATAGGGATAACAATAATATTAATACCAATATGTATATTATCAACGTGATCGAGTGTG
AAAAAGGAAGTAAAGTTATATATAGGATTATGATTAGTATTAGAAACCGTCTTAATATTA
GTATTTCTATGAATAGACATACTATTATTTTATATAACCTTTGAAACAAGTTTAATACCA
ATGTATTTAATAATAGGGATATATGGGGGTAAAAAAAGGAAGATATATGCAGCATACCAA
TTTTTCTTAATAACATTATTAGGCTCATTATTGATGTTAATGGGGATAATAATGTTATAT
TCACAAATAGGGGTAACAGATTATCAAATATACACATTAACAGTGGAGCTAACAAAAGAA
AGAGAAAAAATAATATGGTTAGCTTTATTTATATCATTTGCGGTGAAAACACCATTAGTA
CCGGTTCATATTTGGTTACCTGAAGCTCATAGTGAAGCAAATATAGCCGGTTCAATAATA
TTAGCGGGTGTATTATTAAAATTAGCCGGCTATGGCTTTCTGAGATATTCACTGAATATA
TTACCTGAGGCATCAAGATATTATATTCCCTTAGTATATGGTTTATCTATAATAAGTATC
ATTTATTGTAGTTTAACCACATTAAGACAAATAGATATGAAAAAAATCATAGCATATTCA
TCCATAGGTCATATGGGGATAGTAATATTAGGTATATTTTCCAATACGATGGAGGGGCTA
GAGGGGTCGATGATATTAATGATAGGTCATGGATTGGTATCACCGGGGTTATTTATAATA
GTAACGATATTATATGAAAGATATCATAGTCGAATAATAAAATATTATAGGGGGGTGACG
TCGACAAGTCCATTAATAGCGATATTATTTATAATATATACCTTAGCCAATATGGGTGTA
CCTTTATCGAGTAATTTTATAGGGGAAATATTATGTTTAATGGGTGCTTGGGAAACCAAT
CCGATATCGACGATAATAGCGTCAACCGGGTTAATATTAGGGGGGGCTTATAGTATATGG
TTTTATAATAGAATAAGCTTTGGGGAACCCTCAATATATTATAGGGAGCTAGATATAAAC
CGAAGGGAGTTTGAAGAACTATTACCTCTATTAATATTGATAATAATCATAGGTGTGTTT
CCGAATCTGATATTAGACACTTTACATACAGCGGTGAGTAATATATTTTGGGCCGCCGGG
TAG
>nad4-HHdFL050913-1
ATGTTAAGTATAATAGCAATAATACCCTTGATAGGTTTATGAATAATAATGATGAGCAGA
GATGAAAAACAGTATAAAATTATTGGATTAGTAACATCATTACTAAACTTAGTAATATAT
CCAATAATATGGGCGCAACACAATTCAAATAATAATTACTTTCAGATTATAGAGAAAACG
TCTTCACCCCTTGACACTTATTATCCAGAAATAATCGGGGTAGATAGTATATCATTATAT
ATGATAGGGATAACAATAATATTAATACCAATATGTATATTATCAACGTGATCGAGTGTG
AAAAAGGAAGTAAAGTTATATATAGGATTATGATTAGTATTAGAAACCGTCTTAATATTA
GTATTTCTATGAATAGACATACTATTATTTTATATAACCTTTGAAACAAGTTTAATACCA
ATGTATTTAATAATAGGGATATATGGGGGTAAAAAAAGGAAGATATATGCAGCATACCAA
TTTTTCTTAATAACATTATTAGGCTCATTATTGATGTTAATGGGGATAATAATGTTATAT
TCACAAATAGGGGTAACAGATTATCAAATATACACATTAACAGTGGAGCTAACAAAAGAA
AGAGAAAAAATAATATGGTTAGCTTTATTTATATCATTTGCGGTGAAAACACCATTAGTA
CCGGTTCATATTTGGTTACCTGAAGCTCATAGTGAAGCAAATATAGCCGGTTCAATAATA
TTAGCGGGTGTATTATTAAAATTAGCCGGCTATGGCTTTCTGAGATATTCACTGAATATA
TTACCTGAGGCATCAAGATATTATATTCCCTTAGTATATGGTTTATCTATAATAAGTATC

ATTTATTGTAGTTTAACCACATTAAGACAAATAGATATGAAAAAAATCATAGCATATTCA
TCCATAGGTCATATGGGGATAGTAATATTAGGTATATTTTCCAATACGATGGAGGGGCTA
GAGGGGTCGATGATATTAATGATAGGTCATGGATTGGTATCACCGGGGTTATTTATAATA
GTAACGATATTATATGAAAGATATCATAGTCGAATAATAAAATATTATAGGGGGGTGACG
TCGACAAGTCCATTAATAGCGATATTATTTATAATATATACCTTAGCCAATATGGGTGTA
CCTTTATCGAGTAATTTTATAGGGGAAATATTATGTTTAATGGGTGCTTGGGAAACCAAT
CCGATATCGACGATAATAGCGTCAACCGGGTTAATATTAGGGGGGGCTTATAGTATATGG
TTTTATAATAGAATAAGCTTTGGGGAACCCTCAATATATTATAGGGAGCTAGATATAAAC
CGAAGGGAGTTTGAAGAACTATTACCTCTATTAATATTGATAATAATCATAGGTGTGTTT
CCGAATCTGATATTAGACACTTTACATACAGCGGTGAGTAATATATTTTGGGCCGCCGGG
TAG
>nad4-consensus
ATGTTAAGTATAATAGCAATAATACCCTTGATAGGTTTATGAATAATAATGATGAGCAGA
GATGAAAAACAGTATAAAATTATTGGATTAGTAACATCATTACTAAACTTAGTAATATAT
CCAATAATATGGGCGCAACACAATTCAAATAATAATTACTTTCAGATTATAGAGAAAACG
TCTTCACCCCTTGACACTTATTATCCAGAAATAATCGGGGTAGATAGTATATCATTATAT
ATGATAGGGATAACAATAATATTAATACCAATATGTATATTATCAACGTGATCGAGTGTG
AAAAAGGAAGTAAAGTTATATATAGGATTATGATTAGTATTAGAAACCGTCTTAATATTA
GTATTTCTATGAATAGACATACTATTATTTTATATAACCTTTGAAACAAGTTTAATACCA
ATGTATTTAATAATAGGGATATATGGGGGTAAAAAAAGGAAGATATATGCAGCATACCAA
TTTTTCTTAATAACATTATTAGGCTCATTATTGATGTTAATGGGGATAATAATGTTATAT
TCACAAATAGGGGTAACAGATTATCAAATATACACATTAACAGTGGAGCTAACAAAAGAA
AGAGAAAAAATAATATGGTTAGCTTTATTTATATCATTTGCGGTGAAAACACCATTAGTA
CCGGTTCATATTTGGTTACCTGAAGCTCATAGTGAAGCAAATATAGCCGGTTCAATAATA
TTAGCGGGTGTATTATTAAAATTAGCCGGCTATGGCTTTCTGAGATATTCACTGAATATA
TTACCTGAGGCATCAAGATATTATATTCCCTTAGTATATGGTTTATCTATAATAAGTATC
ATTTATTGTAGTTTAACCACATTAAGACAAATAGATATGAAAAAAATCATAGCATATTCA
TCCATAGGTCATATGGGGATAGTAATATTAGGTATATTTTCCAATACGATGGAGGGGCTA
GAGGGGTCGATGATATTAATGATAGGTCATGGATTGGTATCACCGGGGTTATTTATAATA
GTAACGATATTATATGAAAGATATCATAGTCGAATAATAAAATATTATAGGGGGGTGACG
TCGACAAGTCCATTAATAGCGATATTATTTATAATATATACCTTAGCCAATATGGGTGTA
CCTTTATCGAGTAATTTTATAGGGGAAATATTATGTTTAATGGGTGCTTGGGAAACCAAT
CCGATATCGACGATAATAGCGTCAACCGGGTTAATATTAGGGGGGGCTTATAGTATATGG
TTTTATAATAGAATAAGCTTTGGGGAACCCTCAATATATTATAGGGAGCTAGATATAAAC
CGAAGGGAGTTTGAAGAACTATTACCTCTATTAATATTGATAATAATCATAGGTGTGTTT
CCGAATCTGATATTAGACACTTTACATACAGCGGTGAGTAATATATTTTGGGCCGCCGGG
TAG

>nad5-KVL-14-117
ATGTATCTGGGAATTATCACCCTCCCGCTAATAACCGGTTTAATATGTGGTTTATTTGGG
AGAAATCTAGGTAACCAGGGAACAAAACTATTAGGGACTCATTCGATAGTAATAGGGACA
TTAATAAGCATAACTCTATATTATGAGATAGCGATTTCAGAATCACCATTAAGTATATAT
TTACTAGATTGGGTAGATACAGAGTATCTGGGTTTAAGCTGGGGATTTAAACTAGATAGT
TTAAGTATAAGTATATATATACCGGTATTAATAGTATCAAGTTGTGTGCATATATATTCG

AGGATATATATGAGTGAGGATGGGCATAATGCAAGATTTTATAGTTATTTATCTTTGTTT
ACATTTTTCATGATAGTGTTAATAAGTGGTGATAATCTACTTTTAATTTTCATAGGTTGG
GAAGGTGTTGGAGTATGTTCATATATATTAGTAAATTTTTGGTATAAAAGGATACAAGCT
AATAAAGCGGCAATGAAAGCATTAATAGTGAACCGGGTAGGGGATTGGAGTTTAACTTTA
GGGATGATATTAATCTGGTGGATCTTTGGGGATCTAGAGATATCGACAATATTAAGTCTA
TCCCGGTTTATAGATGTAGAGTGAATTACGATAGTAACCATATTAGTATTAATTGGGGCG
ATGGCGAAATCAGCACAGTTAGGCCTTCACACCTGATTGCCGGATGCAATGGAAGGCCCG
ACTCCAGTTTCAGCATTAATTCATGCTGCAACAATGGTAACGGCAGGAGTATATTTATTA
ATAAGATTATCGCCATTAATAGAGTATAGTAGCACAGGGTTACTAATAATAAGTTGGGTG
GGTGGTTTAACTGCAATATTTGCGGCAAGCATAGCCACTTTCCAAAATGATCTAAAAAGA
GTAATAGCTTATTCAACCTGTAGCCAGCTTGGATATATGGTGTTAGCAGTGGGTATGAGT
CAATATACATTAAGTGCCTATCATTTAGTAAATCATGCTTATTTCAAAGCATTATTATTT
TTGTCCGCCGGAGCAGTAATTCATAGTCTTTCAGATGAACAGGACATGAGGAGGCATGGA
GGGTTAATAAATATATTACCTTTCACCTATATATGTATATTAATAGGTTCGTTAAGTTTA
TTAGCCACGCCTTACTTAACAGGGTTTTATTCAAAAGATTTAATCTTAGAAGTAGCCTAT
GGGCAATATAATAGTCAAGGAAGTATAGGATATTGGTTAGGGACAATAACGGCGTGTATA
ACCTCTTTTTATTCGTTTAGATTAATATATTTAACTTTTATATCAAAAACTAATGGGGTG
AGATCAAATTATTTAAACGGACATGAACCTTCAATAATAATGACAACGCCTTTAGTCATA
TTAAGTTTATTCTCCATCGTCCATGGTTATTTAGCTAGTGATGCATATATAGGATTAGGT
AGTGGATTATGGGGTGATGGAAGTATATTAATTACACCAGATAATCTAACCACCATAGAA
GGTGAATTTGGTTTACCTTTACAGAATAAGTTATTGCCTTTATTTGGGAGTTTAATGTTT
ATAATATTAGGTATAGGACTAATATATACCGAAGGGGGACGAGTGAGCTCGGTGAGAGGG
ATAAAAATATATAGATTTTTCAATGAAAGATATCATATCGATAATATATATAATAGTTTA
ATAGTCTCTAATATCTTAAAATTAGCCAGAATAACAAGTAAAAGATTAGATAAAGGAATC
TTGGAATTAATCGGTGTTACCGCCATTATAAGAGTAGTTAATAATATATCTTCTAAATTA
GCTTCCTTTGATAATGGTTATATACCTCATTTAGCTTTAAATATAATAATTGGTTTAATT
TTAGCATTATCACTCGCCGAGGGGCTTTACCCTGGAAAGAATTTAATCTTAATCTTAATA
TTAACTCTGTCGAATAATTCATCATTAAACCGTCAT**TAA**
>nad5-KVL-14-118
ATGTATCTGGGAATTATCACCCTCCCGCTAATAACCGGTTTAATATGTGGTTTATTTGGG
AGAAATCTAGGTAACCAGGGAACAAAACTATTAGGGACTCATTCGATAGTAATAGGGACA
TTAATAAGCATAACTCTATATTATGAGATAGCGATTTCAGAATCACCATTAAGTATATAT
TTACTAGATTGGGTAGATACAGAGTATCTGGGTTTAAGCTGGGGATTTAAACTAGATAGT
TTAAGTATAAGTATATATATACCGGTATTAATAGTATCAAGTTGTGTGCATATATATTCG
AGGATATATATGAGTGAGGATGGGCATAATGCAAGATTTTATAGTTATTTATCTTTGTTT
ACATTTTTCATGATAGTGTTAATAAGTGGTGATAATCTACTTTTAATTTTCATAGGTTGG
GAAGGTGTTGGAGTATGTTCATATATATTAGTAAATTTTTGGTATAAAAGGATACAAGCT
AATAAAGCGGCAATGAAAGCATTAATAGTGAACCGGGTAGGGGATTGGAGTTTAACTTTA
GGGATGATATTAATCTGGTGGATCTTTGGGGATCTAGAGATATCGACAATATTAAGTCTA
TCCCGGTTTATAGATGTAGAGTGAATTACGATAGTAACCATATTAGTATTAATTGGGGCG
ATGGCGAAATCAGCACAGTTAGGCCTTCACACCTGATTGCCGGATGCAATGGAAGGCCCG
ACTCCAGTTTCAGCATTAATTCATGCTGCAACAATGGTAACGGCAGGAGTATATTTATTA
ATAAGATTATCGCCATTAATAGAGTATAGTAGCACAGGGTTACTAATAATAAGTTGGGTG
GGTGGTTTAACTGCAATATTTGCGGCAAGCATAGCCACTTTCCAAAATGATCTAAAAAGA

GTAATAGCTTATTCAACCTGTAGCCAGCTTGGATATATGGTGTTAGCAGTGGGTATGAGT
CAATATACATTAAGTGCCTATCATTTAGTAAATCATGCTTATTTCAAAGCATTATTATTT
TTGTCCGCCGGAGCAGTAATTCATAGTCTTTCAGATGAACAGGACATGAGGAGGCATGGA
GGGTTAATAAATATATTACCTTTCACCTATATATGTATATTAATAGGTTCGTTAAGTTTA
TTAGCCACGCCTTACTTAACAGGGTTTTATTCAAAAGATTTAATCTTAGAAGTAGCCTAT
GGGCAATATAATAGTCAAGGAAGTATAGGATATTGGTTAGGGACAATAACGGCGTGTATA
ACCTCTTTTTATTCGTTTAGATTAATATATTTAACTTTTATATCAAAAACTAATGGGGTG
AGATCAAATTATTTAAACGGACATGAACCTTCAATAATAATGACAACGCCTTTAGTCATA
TTAAGTTTATTCTCCATCGTCCATGGTTATTTAGCTAGTGATGCATATATAGGATTAGGT
AGTGGATTATGGGGTGATGGAAGTATATTAATTACACCAGATAATCTAACCACCATAGAA
GGTGAATTTGGTTTACCTTTACAGAATAAGTTATTGCCTTTATTTGGGAGTTTAATGTTT
ATAATATTAGGTATAGGACTAATATATACCGAAGGGGGACGAGTGAGCTCGGTGAGAGGG
ATAAAAATATATAGATTTTTCAATGAAAGATATCATATCGATAATATATATAATAGTTTA
ATAGTCTCTAATATCTTAAAATTAGCCAGAATAACAAGTAAAAGATTAGATAAAGGAATC
TTGGAATTAATCGGTGTTACCGCCATTATAAGAGTAGTTAATAATATATCTTCTAAATTA
GCTTCCTTTGATAATGGTTATATACCTCATTTAGCTTTAAATATAATAATTGGTTTAATT
TTAGCATTATCACTCGCCGAGGGGCTTTACCCTGGAAAGAATTTAATCTTAATCTTAATA
TTAACTCTGTCGAATAATTCATCATTAAACCGTCAT**TAA**
>nad5-HHdFL130914-1
ATGTATCTGGGAATTATCACCCTCCCGCTAATAACCGGTTTAATATGTGGTTTATTTGGG
AGAAATCTAGGTAACCAGGGAACAAAACTATTAGGGACTCATTCGATAGTAATAGGGACA
TTAATAAGCATAACTCTATATTATGAGATAGCGATTTCAGAATCACCATTAAGTATATAT
TTACTAGATTGGGTAGATACAGAGTATCTGGGTTTAAGCTGGGGATTTAAACTAGATAGT
TTAAGTATAAGTATATATATACCGGTATTAATAGTATCAAGTTGTGTGCATATATATTCG
AGGATATATATGAGTGAGGATGGNNNNNNNNNNNNNNNNNNNNNNNNNNNNNNNNNNNNN
NNNNNNNNNNNNNNNNNNNNNNNNNNNNNNNNNNNNNNNNNNNNNNNNNNNNNNNNNNNN
NNNNNTGTTGGAGTATGTTCATATATATTAGTAAATTTTTGGTATAAAAGGATACAAGCT
AATAAAGCGGCAATGAAAGCATTAATAGTGAACCGGGTAGGGGATTGGAGTTTAACTTTA
GGGATGATATTAATCTGGTGGATCTTTGGGGATCTAGAGATATCGACAATATTAAGTCTA
TCCCGGTTTATAGATGTAGAGTGAATTACGATAGTAACCATATTAGTATTAATTGGGGCG
ATGGCGAAATCAGCACAGTTAGGCCTTCACACCTGATTGCCGGATGCAATGGAAGGCCCG
ACTCCAGTTTCAGCATTAATTCATGCTGCAACAATGGTAACGGCAGGAGTATATTTATTA
ATAAGATTATCGCCATTAATAGAGTATAGTAGCACAGGGTTACTAATAATAAGTTGGGTG
GGTGGTTTAACTGCAATATTTGCGGCAAGCATAGCCACTTTCCAAAATGATCTAAAAAGA
GTAATAGCTTATTCAACCTGTAGCCAGCTTGGATATATGGTGTTAGCAGTGGGTATGAGT
CAATATACATTAAGTGCCTATCATTTAGTAAATCATGCTTATTTCAAAGCATTATTATTT
TTGTCCGCCGGAGCAGTAATTCATAGTCTTTCAGATGAACAGGACATGAGGAGGCATGGA
GGGTTAATAAATATATTACCTTTCACCTATATATGTATATTAATAGGTTCGTTAAGTTTA
TTAGCCACGCCTTACTTAACAGNNNNNNNNNNNNNNNNNNNNNNNNNNNNNNNNNNNNNN
NNNNNNNNNNNNNNNNNNNNNNNNNNNNNNNNNNNNNNNNNGACAATAACGGCGTGTATA
ACCTCTTTTTATTCGTTTAGATTAATATATTTAACTTTTATATCAAAAACTAATGGGGTG
AGATCAAATTATTTAAACGGACATGAACCTTCAATAATAATGACAACGCCTTTAGTCATA
TTAAGTTTATTCTCCATCGTCCATGGTTATTTAGCTAGTGATGCATATATAGGATTAGGT
AGTGGATTATGGGGTGATGGAAGTATATTAATTACACCAGATAATCTAACCACCATAGAA

GGTGAATTTGGTTTACCTTTACAGAATAAGTTATTGCCTTTATTTGGGAGTTTAATGTTT
ATAATATTAGGTATAGGACTAATATATACCGAAGGGGGACGAGTGAGCTCGGTGAGAGGG
ATAAAAATATATAGATTTTTCAATGAAAGATATCATATCGATAATATATATAATAGTTTA
ATAGTCTCTAATATCTTAAAATTAGCCAGAATAACAAGTAAAAGATTAGATAAAGGAATC
TTGGAATTAATCGGTGTTACCGCCATTATAAGAGTAGTTAATAATATATCTTCTAAATTA
GCTTCCTTTGATAATGGTTATATACCTCATTTAGCTTTAAATATAATAATTGGTTTAATT
TTAGCATTATCACTCGCCGAGGGGCTTTACCCTGGAAAGAATTTAATCTTAATCTTAATA
TTAACTCTGTCGAATAATTCATCATTAAACCGTCAT**TAA**
>nad5-Berkeley
ATGTATCTGGGAATTATCACCCTCCCGCTAATAACCGGTTTAATATGTGGTTTATTTGGG
AGAAATCTAGGTAACCAGGGAACAAAACTATTAGGGACTCATTCGATAGTAATAGGGACA
TTAATAAGCATAACTCTATATTATGAGATAGCGATTTCAGAATCACCATTAAGTATATAT
TTACTAGATTGGGTAGATACAGAGTATCTGGGTTTAAGCTGGGGATTTAAACTAGATAGT
TTAAGTATAAGTATATATATACCGGTATTAATAGTATCAAGTTGTGTGCATATATATTCG
AGGATATATATGAGTGAGGATGGGCATAATGCAAGATTTTATAGTTATTTATCTTTGTTT
ACATTTTTCATGATAGTGTTAATAAGTGGTGATAATCTACTTTTAATTTTCATAGGTTGG
GAAGGTGTTGGAGTATGTTCATATATATTAGTAAATTTTTGGTATAAAAGGATACAAGCT
AATAAAGCGGCAATGAAAGCATTAATAGTGAACCGGGTAGGGGATTGGAGTTTAACTTTA
GGGATGATATTAATCTGGTGGATCTTTGGGGATCTAGAGATATCGACAATATTAAGTCTA
TCCCGGTTTATAGATGTAGAGTGAATTACGATAGTAACCATATTAGTATTAATTGGGGCG
ATGGCGAAATCAGCACAGTTAGGCCTTCACACCTGATTGCCGGATGCAATGGAAGGCCCG
ACTCCAGTTTCAGCATTAATTCATGCTGCAACAATGGTAACGGCAGGAGTATATTTATTA
ATAAGATTATCGCCATTAATAGAGTATAGTAGCACAGGGTTACTAATAATAAGTTGGGTG
GGTGGTTTAACTGCAATATTTGCGGCAAGCATAGCCACTTTCCAAAATGATCTAAAAAGA
GTAATAGCTTATTCAACCTGTAGCCAGCTTGGATATATGGTGTTAGCAGTGGGTATGAGT
CAATATACATTAAGTGCCTATCATTTAGTAAATCATGCTTATTTCAAAGCATTATTATTT
TTGTCCGCCGGAGCAGTAATTCATAGTCTTTCAGATGAACAGGACATGAGGAGGCATGGA
GGGTTAATAAATATATTACCTTTCACCTATATATGTATATTAATAGGTTCGTTAAGTTTA
TTAGCCACGCCTTACTTAACAGGGTTTTATTCAAAAGATTTAATCTTAGAAGTAGCCTAT
GGGCAATATAATAGTCAAGGAAGTATAGGATATTGGTTAGGGACAATAACGGCGTGTATA
ACCTCTTTTTATTCGTTTAGATTAATATATTTAACTTTTATATCAAAAACTAATGGGGTG
AGATCAAATTATTTAAACGGACATGAACCTTCAATAATAATGACAACGCCTTTAGTCATA
TTAAGTTTATTCTCCATCGTCCATGGTTATTTAGCTAGTGATGCATATATAGGATTAGGT
AGTGGATTATGGGGTGATGGAAGTATATTAATTACACCAGATAATCTAACCACCATAGAA
GGTGAATTTGGTTTACCTTTACAGAATAAGTTATTGCCTTTATTTGGGAGTTTAATGTTT
ATAATATTAGGTATAGGACTAATATATACCGAAGGGGGACGAGTGAGCTCGGTGAGAGGG
ATAAAAATATATAGATTTTTCAATGAAAGATATCATATCGATAATATATATAATAGTTTA
ATAGTCTCTAATATCTTAAAATTAGCCAGAATAACAAGTAAAAGATTAGATAAAGGAATC
TTGGAATTAATCGGTGTTACCGCCATTATAAGAGTAGTTAATAATATATCTTCTAAATTA
GCTTCCTTTGATAATGGTTATATACCTCATTTAGCTTTAAATATAATAATTGGTTTAATT
TTAGCATTATCACTCGCCGAGGGGCTTTACCCTGGAAAGAATTTAATCTTAATCTTAATA
TTAACTCTGTCGAATAATTCATCATTAAACCGTCAT**TAA**
>nad5-HHdFL050913-1
ATGTATCTGGGAATTATCACCCTCCCGCTAATAACCGGTTTAATATGTGGTTTATTTGGG

AGAAATCTAGGTAACCAGGGAACAAAACTATTAGGGACTCATTCGATAGTAATAGGGACA
TTAATAAGCATAACTCTATATTATGAGATAGCGATTTCAGAATCACCATTAAGTATATAT
TTACTAGATTGGGTAGATACAGAGTATCTGGGTTTAAGCTGGGGATTTAAACTAGATAGT
TTAAGTATAAGTATATATATACCGGTATTAATAGTATCAAGTTGTGTGCATATATATTCG
AGGATATATATGAGTGAGGATGGGCATAATGCAAGATTTTATAGTTATTTATCTTTGTTT
ACATTTTTCATGATAGTGTTAATAAGTGGTGATAATCTACTTTTAATTTTCATAGGTTGG
GAAGGTGTTGGAGTATGTTCATATATATTAGTAAATTTTTGGTATAAAAGGATACAAGCT
AATAAAGCGGCAATGAAAGCATTAATAGTGAACCGGGTAGGGGATTGGAGTTTAACTTTA
GGGATGATATTAATCTGGTGGATCTTTGGGGATCTAGAGATATCGACAATATTAAGTCTA
TCCCGGTTTATAGATGTAGAGTGAATAACGATAGTAACCATATTAGTATTAATTGGGGCG
ATGGCGAAATCAGCACAGTTAGGCCTTCACACCTGATTGCCGGATGCAATGGAAGGCCCG
ACTCCAGTTTCAGCATTAATTCATGCTGCAACAATGGTAACGGCAGGAGTATATTTATTA
ATAAGATTATCGCCATTAATAGAGTATAGTAGCANNNNNNNNNNNNNNNNNNNNNNNNNN
NNNNNNNNNNNNNNNNNNNNNNNNNNNNNNNNNGCCACTTTCCAAAATGATCTAAAAAGA
GTAATAGCTTATTCAACCTGTAGCCAGCTTGGATATATGGTGTTAGCAGTGGGTATGAGT
CAATATACATTAAGTGCCTATCATTTAGTAAATCATGCTTATTTCAAAGCATTATTATTT
TTGTCCGCCGGAGCAGTAATTCATAGTCTTTCAGATGAACAGGACATGAGGAGGCATGGA
GGGTTAATAAATATATTACCTTTCACCTATATATGTATATTAATAGGTTCGTTAAGTTTA
TTAGCCACGCCTTACTTAACAGGGTTTTATTCAAAAGATTTAATCTTAGAAGTAGCCTAT
GGGCAATATAATAGTCAAGGAAGTATAGGATATTGGTTAGGGACAATAACGGCGTGTATA
ACCTCTTTTTATTCGTTTAGATTAATATATTTAACTTTTATATCAAAAACTAATGGGGTG
AGATCAAATTATTTAAACGGACATGAACCTTCAATAATAATGACAACGCCTTTAGTCATA
TTAAGTTTATTCTCCATCGTCCATGGTTATTTAGCTAGTGATGCATATATAGGATTAGGT
AGTGGATTATGGGGTGATGGAAGTATATTAATTACACCAGATAATCTAACCACCATAGAA
GGTGAATTTGGTTTACCTTTACAGAATAAGTTATTGCCTTTATTTGGGAGTTTAATGTTT
ATAATATTAGGTATAGGACTAATATATACCGAAGGGGGACGAGTGAGCTCGGTGAGAGGG
ATAAAAATATATAGATTTTTCAATGAAAGATATCATATCGATAATATATATAATAGTTTA
ATAGTCTCTAATATCTTAAAATTAGCCAGAATAACAAGTAAAAGATTAGATAAAGGAATC
TTGGAATTAATCGGTGTTACCGCCATTATAAGAGTAGTTAATAATATATCTTCTAAATTA
GCTTCCTTTGATAATGGTTATATACCTCATTTAGCTTTAAATATAATAATTGGTTTAATT
TTAGCATTATCACTCGCCGAGGGGCTTTACCCTGGAAAGAATTTAATCTTAATCTTAATA
TTAACTCTGTCGAATAATTCATCATTAAACCGTCAT**TAA**
>nad5-consensus
ATGTATCTGGGAATTATCACCCTCCCGCTAATAACCGGTTTAATATGTGGTTTATTTGGG
AGAAATCTAGGTAACCAGGGAACAAAACTATTAGGGACTCATTCGATAGTAATAGGGACA
TTAATAAGCATAACTCTATATTATGAGATAGCGATTTCAGAATCACCATTAAGTATATAT
TTACTAGATTGGGTAGATACAGAGTATCTGGGTTTAAGCTGGGGATTTAAACTAGATAGT
TTAAGTATAAGTATATATATACCGGTATTAATAGTATCAAGTTGTGTGCATATATATTCG
AGGATATATATGAGTGAGGATGGGCATAATGCAAGATTTTATAGTTATTTATCTTTGTTT
ACATTTTTCATGATAGTGTTAATAAGTGGTGATAATCTACTTTTAATTTTCATAGGTTGG
GAAGGTGTTGGAGTATGTTCATATATATTAGTAAATTTTTGGTATAAAAGGATACAAGCT
AATAAAGCGGCAATGAAAGCATTAATAGTGAACCGGGTAGGGGATTGGAGTTTAACTTTA
GGGATGATATTAATCTGGTGGATCTTTGGGGATCTAGAGATATCGACAATATTAAGTCTA
TCCCGGTTTATAGATGTAGAGTGAATTACGATAGTAACCATATTAGTATTAATTGGGGCG

ATGGCGAAATCAGCACAGTTAGGCCTTCACACCTGATTGCCGGATGCAATGGAAGGCCCG
ACTCCAGTTTCAGCATTAATTCATGCTGCAACAATGGTAACGGCAGGAGTATATTTATTA
ATAAGATTATCGCCATTAATAGAGTATAGTAGCACAGGGTTACTAATAATAAGTTGGGTG
GGTGGTTTAACTGCAATATTTGCGGCAAGCATAGCCACTTTCCAAAATGATCTAAAAAGA
GTAATAGCTTATTCAACCTGTAGCCAGCTTGGATATATGGTGTTAGCAGTGGGTATGAGT
CAATATACATTAAGTGCCTATCATTTAGTAAATCATGCTTATTTCAAAGCATTATTATTT
TTGTCCGCCGGAGCAGTAATTCATAGTCTTTCAGATGAACAGGACATGAGGAGGCATGGA
GGGTTAATAAATATATTACCTTTCACCTATATATGTATATTAATAGGTTCGTTAAGTTTA
TTAGCCACGCCTTACTTAACAGGGTTTTATTCAAAAGATTTAATCTTAGAAGTAGCCTAT
GGGCAATATAATAGTCAAGGAAGTATAGGATATTGGTTAGGGACAATAACGGCGTGTATA
ACCTCTTTTTATTCGTTTAGATTAATATATTTAACTTTTATATCAAAAACTAATGGGGTG
AGATCAAATTATTTAAACGGACATGAACCTTCAATAATAATGACAACGCCTTTAGTCATA
TTAAGTTTATTCTCCATCGTCCATGGTTATTTAGCTAGTGATGCATATATAGGATTAGGT
AGTGGATTATGGGGTGATGGAAGTATATTAATTACACCAGATAATCTAACCACCATAGAA
GGTGAATTTGGTTTACCTTTACAGAATAAGTTATTGCCTTTATTTGGGAGTTTAATGTTT
ATAATATTAGGTATAGGACTAATATATACCGAAGGGGACGAGTGAGCTCGGTGAGAGGG
ATAAAAATATATAGATTTTTCAATGAAAGATATCATATCGATAATATATATAATAGTTTA
ATAGTCTCTAATATCTTAAAATTAGCCAGAATAACAAGTAAAAGATTAGATAAAGGAATC
TTGGAATTAATCGGTGTTACCGCCATTATAAGAGTAGTTAATAATATATCTTCTAAATTA
GCTTCCTTTGATAATGGTTATATACCTCATTTAGCTTTAAATATAATAATTGGTTTAATT
TTAGCATTATCACTCGCCGAGGGGCTTTACCCTGGAAAGAATTTAATCTTAATCTTAATA
TTAACTCTGTCGAATAATTCATCATTAAACCGTCAT**TAA**

>nad6-KVL-14-117
ATGACGAATTTTTTTATGATCTTGAGCATCCTCGCCACTTTCCTTACTATCTCTTGCTCT
AACCCCGTTTACGCAGTTCTAGGTCTTATCTCTKTATTTTGTTTTTCTTCCTCTTATTTT
ATNNNNNNNNNNNNNNNNNNNNNNNNNNNNNNNNNNNNNCTAATTTATGTCGGGGCCATC
GCTATTTTATTCCTGTTCGTTGTCATGATGATTTATCTCAAATTGTTCCCCCATCAGGTT
TTCCCTAGTTTATTGCCAGTTGCTACTGCTATTACGGTTTTTTACTTGTATAGCTCCCCA
GCCCGGGCTTACTTCGCAGATGCTTTTACCTTTTTCTTTTTCTCGCCCCTCACTCTTTCC
TCTTGAACCTCGCTATTTAACAACTCACATCATTTAACTTCTTTTGCTTACCCCCTTTTT
ACTAGTTTCTCACTTTGGTTTCTTTTATCTAGCCTTATTTTATTATTAGCTATCATCGCC
CCTATTCTTTTGATTGTTTCA**TAA**
>nad6-KVL-14-118
ATGACGAATTTTTTTATGATCTTGAGCATCCTCGCCACTTTCCTNNNNNNNNNNNNNNNN
NNNNNNNNNNNNNNNNNNCTAGGTCTTATCTCTTTATTTTGTTTTTCTTCCTCTTATTTA
ATATTGTTAGGTATCGGTTTCCTTGGTTTAACTTATCTACTAATTTATGTCGGGGCCATC
GCTATTTTATTCCTGTTCGTTGTCATGATGATTTATCTCAAATTGTTCCCCCATCAGGTT
TTCCCTAGTTTATTGCCAGTTGCTACTGCTATTACGGTTTTTTACTTGTATAGCTCCCCA
GCCCGGGCTTACTTCGCAGATGCTTTTACCTTTTTCTTTTTCTCGCCCCTCACTCTTTCC
TCTTGAACCTCGCTATTTAACAACTCACATCATTTAACTTCTTTTGCTTACCCCCTTTTT
ACTAGTTTCTCACTTTGGTTTCTTTTATCTAGCCTTATTTTATTATTAGCTATCATCGCC
CCTATTCTTTTGATTGTTTNNNNN
>nad6-HHdFL130914-1

ATGACGAATTTTTTTATGATCTTGAGCATCCTCGCCACTTTCCTTACTATCTCTTGCTCT
AACCCCGTTTACGCAGTTCTAGGTCTTATCTCTTTATTTTGTTTTTCTTCCTCTTATTTA
ATATTGTTAGGTATCGGTTTCCTTGGTTTAACTTATCTACTAATTTATGTCGGGGCCATC
GCTATTTTATTCCTGTTCGTTGTCATGATGATTTATCTCAATTTGTTCCCCCATCAGGTT
TTCCCTAGTTTATTGCCAGTTGCTACTGCTATTACGGTTTTTTACTTGTATAGCTCCCCA
GCCCGGGCTTACTTCGCAGATGCTTTTACCTTTTTCTTTTTCTCGCCCCTCACTCTTTCC
TCTTGAACCTCGCTATTTAACAACTCACATCATTTAACTTCTTTTGCTTACCCCCTTTTT
ACTAGTTTCTCACTTTGGTTTCTTTTATCTAGCCTTATTTTATTATTAGCTATCATCGCC
CCTATTCTTTTGATTGTTTCA**TAA**
>nad6-Berkeley
ATGACGAATTTTTTTATGATCTTGAGCATCCTCGCCACTTTCCTTACTATCTCTTGCTCT
AACCCCGTTTACGCAGTTCTAGGTCTTATCTCTTTATTTTGTTTTTCTTCCTCTTATTTA
ATATTGTTAGGTATCGGTTTCCTTGGTTTAACTTATCTACTAATTTATGTCGGGGCCATC
GCTATTTTATTCCTGTTCGTTGTCATGATGATTTATCTCAAATTGTTCCCCCATCAGGTT
TTCCCTAGTTTATTGCCAGTTGCTACTGCTATTACGGTTTTTTACTTGTATAGCTCCCCA
GCCCGGGCTTACTTCGCAGATGCTTTTACCTTTTTCTTTTTCTCGCCCCTCACTCTTTCC
TCTTGAACCTCGCTATTTAACAACTCACATCATTTAACTTCTTTTGCTTACCCCCTTTTT
ACTAGTTTCTCACTTTGGTTTCTTTTATCTAGCCTTATTTTATTATTAGCTATCATCGCC
CCTATTCTTTTGATTGTTTCA**TAA**
>nad6-HHdFL050913-1
ATGACGAATTTTTTTATGATCTTGAGCATCCTCGCCACTTTCCTTACTATCTCTTGCTCT
AACCCCGTTTACGCAGTTCTAGGTCTTATCTCTTTATTTTGTTTTTCTTCCTCTTATTTA
ATATTGTTAGGTATCGGTTTCCTTGGTTTAACTTATCTACTAATTTATGTCGGGGCCATC
GCTATTTTATTCCTGTTCGTTGTCATGATGATTTATCTCAAATTGTTCCCCCATCAGGTT
TTCCCNNNNNNNNNNNNNNNNNNNNNNNNNNNNNNNNNNNNNNNNNNNNNNNNNNNNNN
NNNNNNNNNNNNNNNNNNNNNNNNNNNNNNNNNNNNNNNNNNNNNNNNCTCACTCTTTCC
TCTTGAACCTCGCTATTTAACAACTCACATCATTTAACTTCTTTTGCTTACCCCCTTTTT
ACTAGTTTCTCACTTTGGTTTCTTTTATCTAGCCTTATTTTATTATTAGCTATCATCGCC
CCTATTCTTTTGATTGTTTCA**TAA**
>nad6-consensus
ATGACGAATTTTTTTATGATCTTGAGCATCCTCGCCACTTTCCTTACTATCTCTTGCTCT
AACCCCGTTTACGCAGTTCTAGGTCTTATCTCTTTATTTTGTTTTTCTTCCTCTTATTTA
ATATTGTTAGGTATCGGTTTCCTTGGTTTAACTTATCTACTAATTTATGTCGGGGCCATC
GCTATTTTATTCCTGTTCGTTGTCATGATGATTTATCTCAAATTGTTCCCCCATCAGGTT
TTCCCTAGTTTATTGCCAGTTGCTACTGCTATTACGGTTTTTTACTTGTATAGCTCCCCA
GCCCGGGCTTACTTCGCAGATGCTTTTACCTTTTTCTTTTTCTCGCCCCTCACTCTTTCC
TCTTGAACCTCGCTATTTAACAACTCACATCATTTAACTTCTTTTGCTTACCCCCTTTTT
ACTAGTTTCTCACTTTGGTTTCTTTTATCTAGCCTTATTTTATTATTAGCTATCATCGCC
CCTATTCTTTTGATTGTTTCA**TAA**

>atp6-consensus
ATGTTATTGTTTAGAAATCCTTTAGAACAATTTATGATTAACGAGTTATTAACGATATTA
TTCCCTTTCTGGGAAGTGTCATTAACAAATATAGGTTTATATTTAATAATGAGTTTAGTA

CTAATAGTAATAATATCACAAATCATCCTCGGCTCGACGGGGGGTGTGCCGATAATAGGG
AATAAAAGTATATTAATAAAACAATCAATATATGATACAGTGCATAAGATAGTAAAAGAT
CAGATAGGTATATCACATGAAATAAATTTACCTTTTTTATACACATTATTTATATTAGTA
TTGACATTAAATTTAATTGGAATTATTCCTTATAATTATAGTACAACCTCGCATCTCGTT
TTAACATTAAGTATGAGTGTATCAATATTAATAGGAGTGACAGTAATGGGAGTAAACCGA
CATAAATTAGTATTTTTTAGTCTACTAATCCCTGGGGGTACTCCGTTAGGTCTGGTACCT
CTATTAGTAATAATAGAAACAATATCTTATCTGGCTAGAGCTATTAGCTTAGGAGTAAGA
TTGGGTGCAAATATGATAGCAGGTCATGTTTTATTAAAAATCTTAGCAGGATTAATCTTG
AAAATAATGAAAACTTCGTTAATAACCGGCTTAATAATAGGATGCCTGCCTATGTTTATC
TTTACTTTATTAGTAGGATTAGAATTAGGTATAAGTGTATTACAAGGGATAGTATTTCTC
ATTTTAACTTCATCTTATATAAAAGATGCTATTGCTTTACAT**TAG**
>atp9-consensus
ATGTTAGAGTCTGCCAAAATTATAGGATCAGGAATAGCAACGATAGGTTTAGCCGGTGCG
GGTGTGGGTATAGGTTTAGTGTTTGCCGCTCTAATAAATTCAACATCTAGAAATCCTTCA
TTAAAAGGACAATTATTCAGTTATTGTATTTTAGGTTTTGCTTTAACTGAAGCTATAGGC
CTTTTTGCAATAATGATGTCATTCTTATTATTATATGCCGCT**TAA**
>cob-consensus
ATGGCAAATAGTTATATAATTGATTCCCCCTCCCCCAGTAATTTAACGTATTTATGGGGT
TATGGGTCATTATTAGGCCTAGTATTAGTTATGCAAATAATAACCGGTGTAACTCTAGCT
ATGCATTATACTCCTAACATTGAGATGGCATTTAATTCAGTTGAACATATCATGAGAGAC
GTAAATAACGGCTGATTAATTCGTTACACTCACGCAAATGTAGCGTCATTTTTTTTTATA
TTTTTATATATCCATATAGGAAGAGGTTTATATTATGGTTCTTATACCAGACCAAGGATA
TTATTATGGTCTATAGGAGTAATAATATTTGTATTAACAATGGCAACAGGGTTTTTGGGT
TATACTCTTCCCTGAGGTCAAATGAGCCTATGAGGTGCAACGGTTATAACTAATTTATTA
TCGGCAATACCTTGGATGGGAGTTAATATAGTGGAATTTGTCTGGGGTGGATTTTCCGTG
GATAATGCGACATTAAACAGATTTTTCAGTCTTCATTATTTATTACCCTTTATATTAGTG
GGATTAGTGGTATTACATTTAGTAGCTCTTCATCACCATGGAAGTAATAACCCATTAGGG
GTATCAGCCAATGGTGATAGAGTACCATTCCATCCTTATTATACTTTTAAAGATATAGTA
GGATTTTTAGCCTTCTTTTTAGTATTATCAATAATTGTCTTCTATATGCCTAATTTATTA
GGTCATTCAGACAACTACATACCAGCCAACCCGCTGGTAACACCAACTCACATACAACCA
GAATGGTATTTTTTACCTTATTATGCAATATTACGATCAATCCCTAATAAGCTATTAGGG
GTTATAGCAATGTTTGCTGCGATATTAATATTATTAGCAATGCCTTTCTTAGATGAATCA
AGAATACGAGGAAGTCAATTTAGACCTTTTATGAAAATAATCTTTTGGTTATTTATATCA
AATTTCGTATTATTAGGATGGATAGGTGCTAAACCAGTATCAGATCCTTACATAATAATA
GGTCAGTTAAGTTCAATATTTTATTTTGCATGGTTTGCAATAGTAGCCATGGTAGGGATA
ATAGAAAATACTCTATATCAAATGAGACAACTTTGGCTGGGGGCCAAA**TAG**
>cox1-consensus
GTGTACGCTATAAGGTGGTTATACTCGACCAATGCCCGAGATATTGGGGTATTATATTTA
ATTTTTGCCATCTTGGCGGGATTAATAGGGACAGTATTATCAATCATAATAAGATTAGAA
TTAGGAGGTGCGGGGGTTCAATATTTACAAGGAGATAACCAATTATACAATGTGGTTGTG
ACGGCACACGCATTCGTC**ATG**ATTTTTTTCATGGTGATGCCGGCGTTAATAGGGGGTTTT
GGGAATTTTATAGTTCCAGTGATGATAGGTGCCGCTGATATGGCATTCCCAAGATTAAAT
AATATATCATTTTGGTTATTACCTCCATCATTGATATTATTATTATTATCATCATTAGTT
GAGAATGGTGCGGGTACTGGTTGGACTGTTTATCCTCCATTATCATCAATCCAAGCTCAC

TCAGGAGGATCGGTTGATTTAGCAATATTCTCGCTTCACCTGGCAGGGATATCTTCTATG
TTAGGTGCAATAAATTTCTTAACAACAATAATTAATATGCGAGCCCCGGGTATGAGCTGG
CATAAATTGCCGTTATTTGTGTGGGCAGTATTTATAACTGCAATATTATTATTATTATCA
TTACCAGTGTTAGCGGGTGGAATAACTATGCTTCTTACAGATAGAAACTTTAATAGTTCT
TTCTATGACCCATCAGCTGGGGGTGATCCAGTGCTTTATCAGCATATTTTCTGATTCTTT
GGTCATCCTGAAGTGTATATATTAATTATACCAGGATTTGGTATAATAAGTCATGTAATA
TCAACTTATTCCGGTAAAAGAGTATTCGGTTATCTAGGTATGATATACGCATTAGCTTCA
ATAGGAATCTTAGGATTCATAGTATGGTCTCATCACATGTTCACTGTAGGTCTAGATGTC
GATACAAGAGCATATTTCACTTCAGCGACATTAATAATTGCAGTGCCAACAGGGATAAAA
ATATTCTCGTGGCTGGCGACGATATACGGGGGAAGTATAAGATTTAGTACTCCTATGTTA
TTTTCTATGGGATTCATATTTTTATTTACAGTAGGAGGATTAACTGGAGTAATATTAGCC
AATGCCTCGTTAGATATAGCTTTACATGATACTTACTACGTGGTAGCACATTTCCATTAT
GTATTATCAATGGGAGCAGTATTTGCATTATTTGCAGCATTCTATTATTGAATAGGTAAA
ATTACTGGAAAACAATATAATGAATTATTAGGGCAGATACATTTTTATACGATGTTTGTC
GGGGTAAATATAACATTCTTCCCAATGCATTTCTTAGGTCTGGCGGGTATGCCACGTAGA
ATACCAGACTATCCAGATGCTTTCATAGGATGGAATTATATAGCATCCTTAGGATCAATA
ATAAGTTTAGTATCAACAGTATTATTCTTATACATAGTCTATGATTTATTAACTAATGAG
GTGGAAGCAAGCGACGGAGCCGATGTAGAAAACGAGTACTTTATATCGAGATGGTCAACT
AAATATGCTCAGACCTTAGAGTGGAGTTTAACAAGCCCACCTGGCTTCCATTGTTATAAT
AGTTTACCAACCTTCCTAACGAAG**TAA**
>cox2-consensus
ATGTGATTAAATTATATAATGAACGATGCACCAGAGGGGTGACAATTAGGTTTTCAAGAT
AGTGCAAGTCCTATAGCAGAAGGGATATTAAGAATACATAATGAAATACAATATTATTTA
TTAATAATTCTAGTATTAATATGTTGGATAATAACTTCAATAATTTTCAAATTCAATGAA
GAAAGTAATAAATTTAAATCTAAATATATGAATCATGGTACATTAATAGAATTAATTTGA
ACAATAACACCTGCGTTAATCTTAGTCGCCATAGCTATACCTTCATTTAAATTATTATAT
TTAATGGATGAAGTAATAGAACCAACCATTACATTACAAATTACCGGTCACCAATGATAT
TGGTCTACAGAATACTCTGACTATGTAAAACAATCAGGCAATATTGAGTTTGACAGTTAT
ATGATCGCAACCTCGGATTTAGAATTAGGTAAATTAAGATTATTGGAAGTAGATAATAGA
GTAATATTACCAGTAGACACACATGTCAGAGTAATAGTTACGAGTACCGATGTAATACAC
TCACTAGCATTACCATCCTTAGGGGTTAAAATTGACTGTTGCCCAGGTCGATTAAACCAA
ACATCTTTCATAATAAAAAGAGAAGGTGTATTCTACGGACAATGCTCAGAACTTTGCGGA
CATGCACACGGTTTCATGCCGATAGTAATAGAGGGGGTAACATTAGAGGATTACTTAGTA
TGGGTTAATGAGTCGGTAGAAGAA**TAG**
>cox3-consensus
ATGAAGTATAACAAACAATCACATCCTTTTCATTTAGTAGAACCGTCACCTTGACCTTTA
ACCACTTCGATAGGCTTAGGTATAATGGCGATGGGGGGAGTAATATATTTTACGGTTTA
GATAGTATTGTATTAATATTAGGTTTGGTTGTGACGATAATGACAAGTACATTATGGTGG
AGAGATTGCATTAGAGAGGGTACATTACAAGGTTATCATACAAAAAAAGTAAGAAAAGGT
ATCAATATAGGATTTATATTATTTATAGTCTCTGAAGTATTCTTCTTTTTTTCTATTTTC
TGGGCCTATTTCCACTCTAGTTTATCACCGTCGGTAGAATTAGGTGGTATGTGGCCGCCA
ATGGGGATAGAAGCATTAAACCTTTGAGAGCTACCTTTATTAAATACAGTAATATTGTTA
TCCTCAGGCGCCACAGTGACAACTGCTCATCATGGATTAATCTATGGGGCGAGACGAATA
GTAATATTAAGTTTAATAACAACTTTAATCTTAGCAGTTTTATTTGTAGGGTGCCAAGGA

TATGAATACTATAATGCACCCTTCAGTTTTTCTGATGGAGCGTATGGATCAACTTTCTTC
TTTGCAACAGGTTTCCATGGGTTGCACGTAATAATCGGTACGATATTTTTAGCAGTAGCC
TTTAATAGGGTTATGAATTATGAATTAACTAATCAGCACCACGTTGGTTTTGAATCCGCA
ATCCTGTATTGACATTTCGTAGACATCGTTTGACTGTTTTTATTTATAGTAATCTACGGT
TGAGGGGCG**TAA**
>nad1-consensus
ATGATCGAGATACTCTCATTAATAATTCCTTTATTAGTTTCAATAGCTTATTTAACTTTA
GCGGAAAGAAAACTCATGGGTTCAATGCAAAGACGATTAGGTCCAAATAAGGTAGGGATA
CTAGGTCTATTACAGCCATTTGCCGATGGTTTAAAATTAGTGATAAAAGAAACGATTATC
GTATCACAAGCTAATAAAATAATCTTTTTCTTGGCTCCCTATATAACATTGGTATTTGGT
TTCTTGGCTTGAGGTGTCATTCCATTTACTAGAGGGATAATAATAGAGGATATAGGTTAT
AGTACATTATATATATTATTGATAACGGGGATAGGGGTATTAGGGATAATATTAGCAGGT
TGGTCAGCTAATTCTAAATATGCAGTATTAGGGTCGTTAAGGACCACCGCCCAGTTAATA
TCTTACGAGGTAGTAATAGGCTTAATGGTAATAATGGTGGTGATGCTAAGTGATGGGTTG
AGTTTAATATCAATCATAGAAGCACAAAAACATATCTGGAATATAATACCAGTATGGCCT
ATATATATCATATTCATGATATCAGCGATAGCAGAAACAAATCGAGCCCCGATGGACTTA
CCTGAAGCTGAATCAGAGTTAGTGGCAGGTTTTCAAACCGAGCACTCAGCCTTATCTTTT
GCTTATTTCTTTTTAGGTGAGTATGGGAATATAATATTAATTTGTACCATCACGGCCAAT
TTCTTTTGGGGGGGTTATAGTATAGGCGGAATTAATTCGGCTTTAATATTAGGCTTAAAA
GTATCGATGCTTTTATTTGTCTTCATCCTCTGCAGGGCTACTTATCCTAGGCTCAGATTT
GACCAATTAATGAGTTTAATGTGAACAGGATTATTGCCACTAGTACTAGGGTATTTTGTA
TTAATCTCTAGTATAATGGTTATTATTACCAACTGG**TAG**
>nad2-consensus
ATGTTAACTTTAAGTATAATAATATTAATAACAAGTTTAGGGAGATGAATAAATATAAAT
AGAATGACGATGATAGTATTTATATATTCAGGAATTTTAAGTATAAATATGACGGGGGAG
AGCAATAGCGCCATAGGGTGTTCCGATATGGGGATGATAAATTCAGGGATAGGGCTATAT
AATGGATTATATCAAGTGACACCAATAACTCAATTCATAGAAACTTATATAAATATATTA
GCGGTAGGCTGTATGGCGATGAAGGCTAGGAGTGAATATAGTATATTAGCCTTAATAGTG
ACGGTAGGGATGAGTAGTTTAATAAGTAGTAATGAGATAGTTAGTATATTAATAGGGATA
GAGTTACAGACATTAGGATTATATGTCATAGCCAGTCTAGATAGAGGTTCAGAGACCTCG
ACGGCAGCGGGGTTAAAATATTATTTATTAGGGGGTTTATCGTCATGTATAATAGGTTTA
GGTTTGAGTATGATATATGGGATAACAGGGGTGACAAATATAGAGGAGTTAAATATATTA
ATAAAAATAAATGACGACAGCCGTCATCTAATAATGACGAAGGTATTAATTACAGTAGGA
TTATTATTTAAAATAGGAGCAGCTCCCTTTCATAATTGGTTAGCGGATGTAATAGATGGA
GTACCGACTGCGATATCGGCATGGTTAGCGGTGGTATCAAAGATATCAATAATAATAATA
TTATTAATATTATATAATGCTATATTAATAAAAATAAACACCAGTGGTGGTGGTCCGATA
ATAATATTAAGTATAATATTATGCTTTATAGTAGGAAGTTTAGTAGGGATAATACAATCT
AGGATAAAAACATTATTAGCTTATAGTAGTATAGCCCATGCGGGGTATATATTAATAGGG
ATAATAGTAAATAATAATCTAGGATTAACAGGTTTAATATTTTATATGGTACAATATTCT
TTAACGGCATTAAATATATTTATGATAATAATAGCCTATGGTGAGGTAAAAGGGGCATCG
ATAGTAAAAATATCACAATTAAAAGAAATCCCTAACACGTGATTAGCGATAAGTTTAGCG
ATAAGTTTATTTTCATCTGCAGGGATTCCACCGTTTATAGGATTTTATGCCAAACTTAAT
ATATTATACTCGGCTCTAGATACAGGTTTTTACTTCATAACAATAATCGCCATACTAACC
AGTATAATAAGTGGTTATTATTATATAAAAGTAATAAAAGTAATATATTTTGAGGGGGAG

TGATGTAGGGTTGGAACCGCCAATGAAGTACGGGGGCGATATCAGAAAATTATTCAATA
ATAATAGCCATGATAAGTTTAGTGATAATATTATTCATCCTCAGTCCCGAGTGACTATAT
AATAGTATTCAGATGATATGC**TAG**
>nad3-consensus
ATGAATAATTTATTTATCTTAATAATCTCAGCCACCGCCATTGTATTCTTATTATTATTT
GTCAATCTTATTGTCGGGGATAAAAAACCTTATACTAATAAATTATCTCCCTATGAATGT
GGTTTAATGCCTTTAGGTGAAGGGAGAGCTAGCTTAAATATACAATATATTTTAGTGGCT
ATTTTATTTATAATATTTGATATTGAGGTAATAGTATTATTTCCATATGCAGTGACTATA
AATAATATATACACATACTGGATAATGATTATCTTTATCATCATTTTAACCATAGGGTTT
TACTTTGAAATTAGTCAAGGGGCTTTAAAATATGTCGATGAAGAGGTT**TAG**
>nad4-consensus
ATGTTAAGTATAATAGCAATAATACCCTTGATAGGTTTATGAATAATAATGATGAGCAGA
GATGAAAAACAGTATAAAATTATTGGATTAGTAACATCATTACTAAACTTAGTAATATAT
CCAATAATATGGGCGCAACACAATTCAAATAATAATTACTTTCAGATTATAGAGAAAACG
TCTTCACCCCTTGACACTTATTATCCAGAAATAATCGGGGTAGATAGTATATCATTATAT
ATGATAGGGATAACAATAATATTAATACCAATATGTATATTATCAACGTGATCGAGTGTG
AAAAAGGAAGTAAAGTTATATATAGGATTATGATTAGTATTAGAAACCGTCTTAATATTA
GTATTTCTATGAATAGACATACTATTATTTTATATAACCTTTGAAACAAGTTTAATACCA
ATGTATTTAATAATAGGGATATATGGGGGTAAAAAAAGGAAGATATATGCAGCATACCAA
TTTTTCTTAATAACATTATTAGGCTCATTATTGATGTTAATGGGGATAATAATGTTATAT
TCACAAATAGGGGTAACAGATTATCAAATATACACATTAACAGTGGAGCTAACAAAAGAA
AGAGAAAAAATAATATGGTTAGCTTTATTTATATCATTTGCGGTGAAAACACCATTAGTA
CCGGTTCATATTTGGTTACCTGAAGCTCATAGTGAAGCAAATATAGCCGGTTCAATAATA
TTAGCGGGTGTATTATTAAAATTAGCCGGCTATGGCTTTCTGAGATATTCACTGAATATA
TTACCTGAGGCATCAAGATATTATATTCCCTTAGTATATGGTTTATCTATAATAAGTATC
ATTTATTGTAGTTTAACCACATTAAGACAAATAGATATGAAAAAAATCATAGCATATTCA
TCCATAGGTCATATGGGGATAGTAATATTAGGTATATTTTCCAATACGATGGAGGGGCTA
GAGGGGTCGATGATATTAATGATAGGTCATGGATTGGTATCACCGGGGTTATTTATAATA
GTAACGATATTATATGAAAGATATCATAGTCGAATAATAAAATATTATAGGGGGGTGACG
TCGACAAGTCCATTAATAGCGATATTATTTATAATATATACCTTAGCCAATATGGGTGTA
CCTTTATCGAGTAATTTTATAGGGGAAATATTATGTTTAATGGGTGCTTGGGAAACCAAT
CCGATATCGACGATAATAGCGTCAACCGGGTTAATATTAGGGGGGGCTTATAGTATATGG
TTTTATAATAGAATAAGCTTTGGGGAACCCTCAATATATTATAGGGAGCTAGATATAAAC
CGAAGGGAGTTTGAAGAACTATTACCTCTATTAATATTGATAATAATCATAGGTGTGTTT
CCGAATCTGATATTAGACACTTTACATACAGCGGTGAGTAATATATTTTGGGCCGCCGGG
TAG
>nad5-consensus
ATGTATCTGGGAATTATCACCCTCCCGCTAATAACCGGTTTAATATGTGGTTTATTTGGG
AGAAATCTAGGTAACCAGGGAACAAAACTATTAGGGACTCATTCGATAGTAATAGGGACA
TTAATAAGCATAACTCTATATTATGAGATAGCGATTTCAGAATCACCATTAAGTATATAT
TTACTAGATTGGGTAGATACAGAGTATCTGGGTTTAAGCTGGGGATTTAAACTAGATAGT
TTAAGTATAAGTATATATATACCGGTATTAATAGTATCAAGTTGTGTGCATATATATTCG
AGGATATATATGAGTGAGGATGGGCATAATGCAAGATTTTATAGTTATTTATCTTTGTTT
ACATTTTTCATGATAGTGTTAATAAGTGGTGATAATCTACTTTTAATTTTCATAGGTTGG

GAAGGTGTTGGAGTATGTTCATATATATTAGTAAATTTTTGGTATAAAAGGATACAAGCT
AATAAAGCGGCAATGAAAGCATTAATAGTGAACCGGGTAGGGGATTGGAGTTTAACTTTA
GGGATGATATTAATCTGGTGGATCTTTGGGGATCTAGAGATATCGACAATATTAAGTCTA
TCCCGGTTTATAGATGTAGAGTGAATTACGATAGTAACCATATTAGTATTAATTGGGGCG
ATGGCGAAATCAGCACAGTTAGGCCTTCACACCTGATTGCCGGATGCAATGGAAGGCCCG
ACTCCAGTTTCAGCATTAATTCATGCTGCAACAATGGTAACGGCAGGAGTATATTTATTA
ATAAGATTATCGCCATTAATAGAGTATAGTAGCACAGGGTTACTAATAATAAGTTGGGTG
GGTGGTTTAACTGCAATATTTGCGGCAAGCATAGCCACTTTCCAAAATGATCTAAAAAGA
GTAATAGCTTATTCAACCTGTAGCCAGCTTGGATATATGGTGTTAGCAGTGGGTATGAGT
CAATATACATTAAGTGCCTATCATTTAGTAAATCATGCTTATTTCAAAGCATTATTATTT
TTGTCCGCCGGAGCAGTAATTCATAGTCTTTCAGATGAACAGGACATGAGGAGGCATGGA
GGGTTAATAAATATATTACCTTTCACCTATATATGTATATTAATAGGTTCGTTAAGTTTA
TTAGCCACGCCTTACTTAACAGGGTTTTATTCAAAAGATTTAATCTTAGAAGTAGCCTAT
GGGCAATATAATAGTCAAGGAAGTATAGGATATTGGTTAGGGACAATAACGGCGTGTATA
ACCTCTTTTTATTCGTTTAGATTAATATATTTAACTTTTATATCAAAAACTAATGGGGTG
AGATCAAATTATTTAAACGGACATGAACCTTCAATAATAATGACAACGCCTTTAGTCATA
TTAAGTTTATTCTCCATCGTCCATGGTTATTTAGCTAGTGATGCATATATAGGATTAGGT
AGTGGATTATGGGGTGATGGAAGTATATTAATTACACCAGATAATCTAACCACCATAGAA
GGTGAATTTGGTTTACCTTTACAGAATAAGTTATTGCCTTTATTTGGGAGTTTAATGTTT
ATAATATTAGGTATAGGACTAATATATACCGAAGGGGGACGAGTGAGCTCGGTGAGAGGG
ATAAAAATATATAGATTTTTCAATGAAAGATATCATATCGATAATATATATAATAGTTTA
ATAGTCTCTAATATCTTAAAATTAGCCAGAATAACAAGTAAAAGATTAGATAAAGGAATC
TTGGAATTAATCGGTGTTACCGCCATTATAAGAGTAGTTAATAATATATCTTCTAAATTA
GCTTCCTTTGATAATGGTTATATACCTCATTTAGCTTTAAATATAATAATTGGTTTAATT
TTAGCATTATCACTCGCCGAGGGGCTTTACCCTGGAAAGAATTTAATCTTAATCTTAATA
TTAACTCTGTCGAATAATTCATCATTAAACCGTCAT**TAA**
>nad6-consensus
ATGACGAATTTTTTTATGATCTTGAGCATCCTCGCCACTTTCCTTACTATCTCTTGCTCT
AACCCCGTTTACGCAGTTCTAGGTCTTATCTCTTTATTTTGTTTTTCTTCCTCTTATTTA
ATATTGTTAGGTATCGGTTTCCTTGGTTTAACTTATCTACTAATTTATGTCGGGGCCATC
GCTATTTTATTCCTGTTCGTTGTCATGATGATTTATCTCAAATTGTTCCCCCATCAGGTT
TTCCCTAGTTTATTGCCAGTTGCTACTGCTATTACGGTTTTTTACTTGTATAGCTCCCCA
GCCCGGGCTTACTTCGCAGATGCTTTTACCTTTTTCTTTTTCTCGCCCCTCACTCTTTCC
TCTTGAACCTCGCTATTTAACAACTCACATCATTTAACTTCTTTTGCTTACCCCCTTTTT
ACTAGTTTCTCACTTTGGTTTCTTTTATCTAGCCTTATTTTATTATTAGCTATCATCGCC
CCTATTCTTTTGATTGTTTCA**TAA**
