# Supplementary material for: Mitovirus and Mitochondrial Coding Sequences from Basal Fungus Entomophthora muscae
Source: Viruses. 2019 Apr 17;11(4):351. doi: 10.3390/v11040351 (PMC6520771; doi:10.3390/v11040351)
Supplement: Supplementary file 1 [file viruses-11-00351-s001.zip › Supp2-EnmuMV-FigureLegendsS1-S5.docx]

**Supplementary Figure Legends**

**Figure S1.** Multiple sequence alignment of deduced RdRp sequences of mitoviruses from *E. muscae* isolate Berkeley. Alignment was performed using MAFFT-G-INS-i. Wholly conserved residues in the 8 sequences are shown in red. The six conserved motifs (I–VI) in mitovirus RdRps as identified by Xie and Ghabrial [17] are underlined and labeled, and the conserved residues within these motifs as identified by Xie and Ghabrial [17] are highlighted by yellow shading. Consensus (100%) was determined using MView as implemented at https://www.ebi.ac.uk/services. Consensus codes from MView: a, aromatic; c, charged; h, hydrophobic; l, aliphatic; o, alcoholic; p, polar; s, small; t, turnlike; u, tiny; -, negative; +, positive.

**Figure S2.** Sequence identity scores from pairwise alignments of *E. muscae* mitovirus sequences. Pairwise scores (in %) were determined using EMBOSS Needleall. Scores for the RNA sequences are shown above the diagonal at upper right; scores for the deduced protein (RdRp) sequences are shown below the diagonal at lower left. For comparing the RNA sequences, terminal residues not determined for all strains were trimmed back to ones shared by all 4 or 5 compared strains within each species. Numbers assigned to the mitovirus strains are defined at left and then used to identify the strains at top of the scoring matrix. Gray shading highlights the higher identity scores for strains within each species.

**Figure S3.** Multiple sequence alignments of 5´ and 3´ NTR sequences of *E. muscae* mitoviruses. Alignments were performed using MAFFT-L-INS-i. The alignments for EnmuMV1–EnmuMV7 are shown in panels A–G, respectively, with the 5´ NTR shown at top and the 3´ NTR shown at bottom in each panel. Indels are highlighted by cyan shading. The proposed start codon (first in-frame AUG) and the apparent stop codon (first in-frame UAA or UAA downstream of the proposed start codon) are shown in red. The first in-frame stop codon upstream of the proposed start codon is shown in orange. A proposed alternative start codon (AUU or AUG) is shown in green for EnmuMV4, EnmuMV5, and EnmuMV6. 5´- and 3´-terminal residues not determined for certain mitovirus strains, likely due to sequence coverage limitations in the original transcriptome studies, are shown as blank spaces. Conserved residues within these terminal regions not determined for all strains are indicated by carets (^).

**Figure S4.** Fungal tree of life based on mitochondrial core-protein sequences from representative species. Deduced sequences for the 12 mitochondrial core proteins that we assembled from *E. muscae* in this report were concatenated and then aligned using MAFFT-E-INS-i. The best-fit (per BIC score) substitution model used for the phylogenetic analysis shown here was LG+F+R5. The site proportions and rates for the FreeRate model were (0.1606,0.0317), (0.1792,0.2354), (0.3252,0.6585), (0.2310,1.5391), and (0.1039,3.6844). The tree is displayed as a rectangular phylogram rooted on a set of three basal opisthokonts included as an outgroup. Branch support values from UFboot (1000 replicates) are shown in %. Scale bar indicates average number of substitutions per alignment position. Fungi are color-coded by phylum as labeled at right.

**Figure S5.** Multiple sequence alignment of N-terminal regions of deduced RdRp sequences of *E. muscae* mitoviruses. In this analysis, to assist in identifying alternative start codons, the RdRp sequences were N-terminally extended by translation to the first upstream in-frame stop codon. Only an N-terminal portion of the alignment is shown. Residues encoded by the first in-frame AUG codon and downstream are shown in black text; upstream residues are shown in gray text. Conserved residues are highlighted with red and orange text. Residues that we propose to be encoded by alternative start codons, likely used instead of the first in-frame AUG codon, are highlighted with gray boxes. For EnmuMV6, this is a Met residue encoded by the second in-frame AUG codon in each strain, and for EnmuMV4, EnmuMV5, and EnmuMV8 this is an Ile residue encoded by an in-frame AUU codon in each strain.
