# Supplementary figures and images for: Mitovirus and Mitochondrial Coding Sequences from Basal Fungus Entomophthora muscae
Source: Viruses. 2019 Apr 17;11(4):351. doi: 10.3390/v11040351 (PMC6520771; doi:10.3390/v11040351)

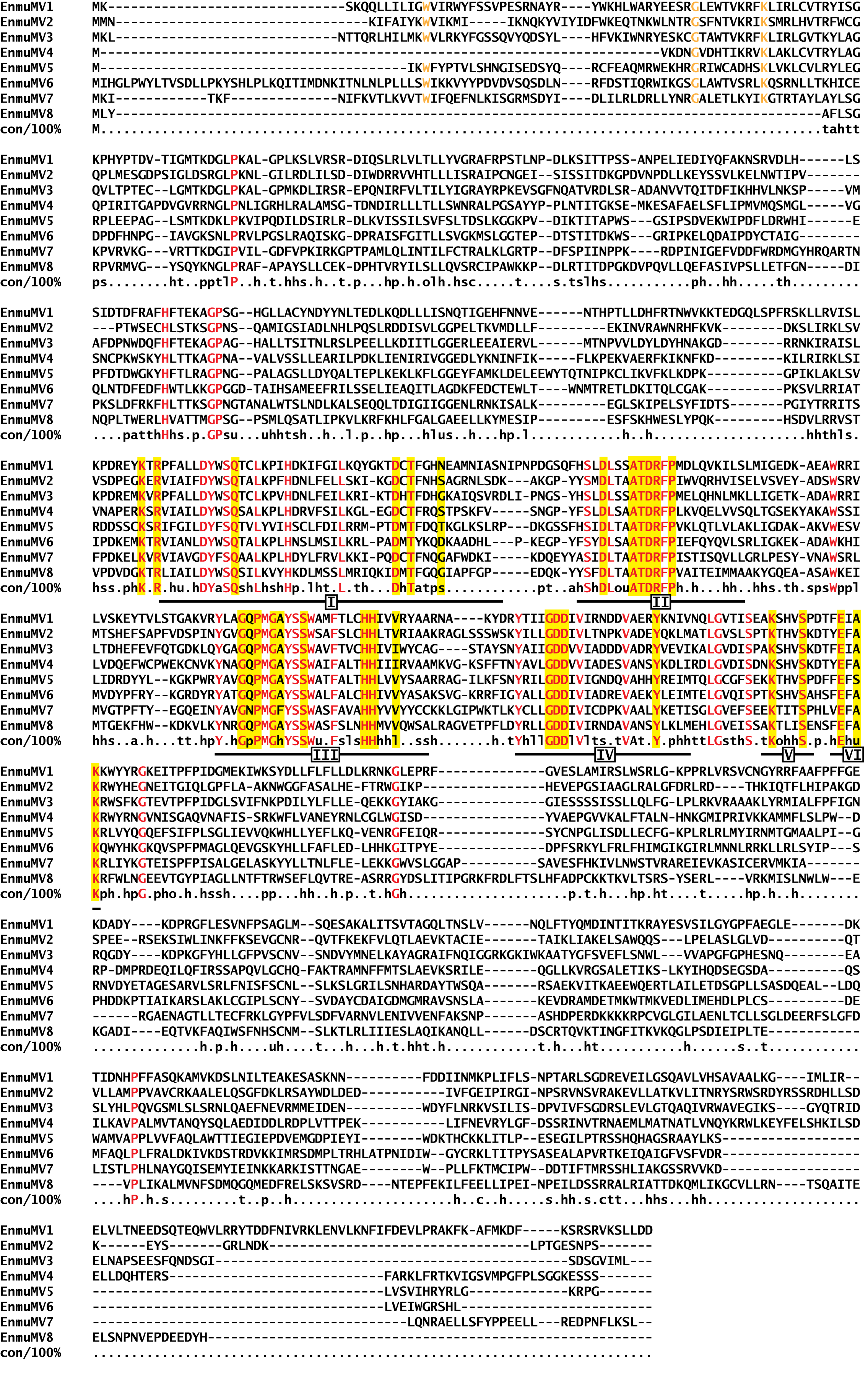

Supplement: Supplementary file 1 [file viruses-11-00351-s001.zip › Supp3-EnmuMV-FigureS1.tif]

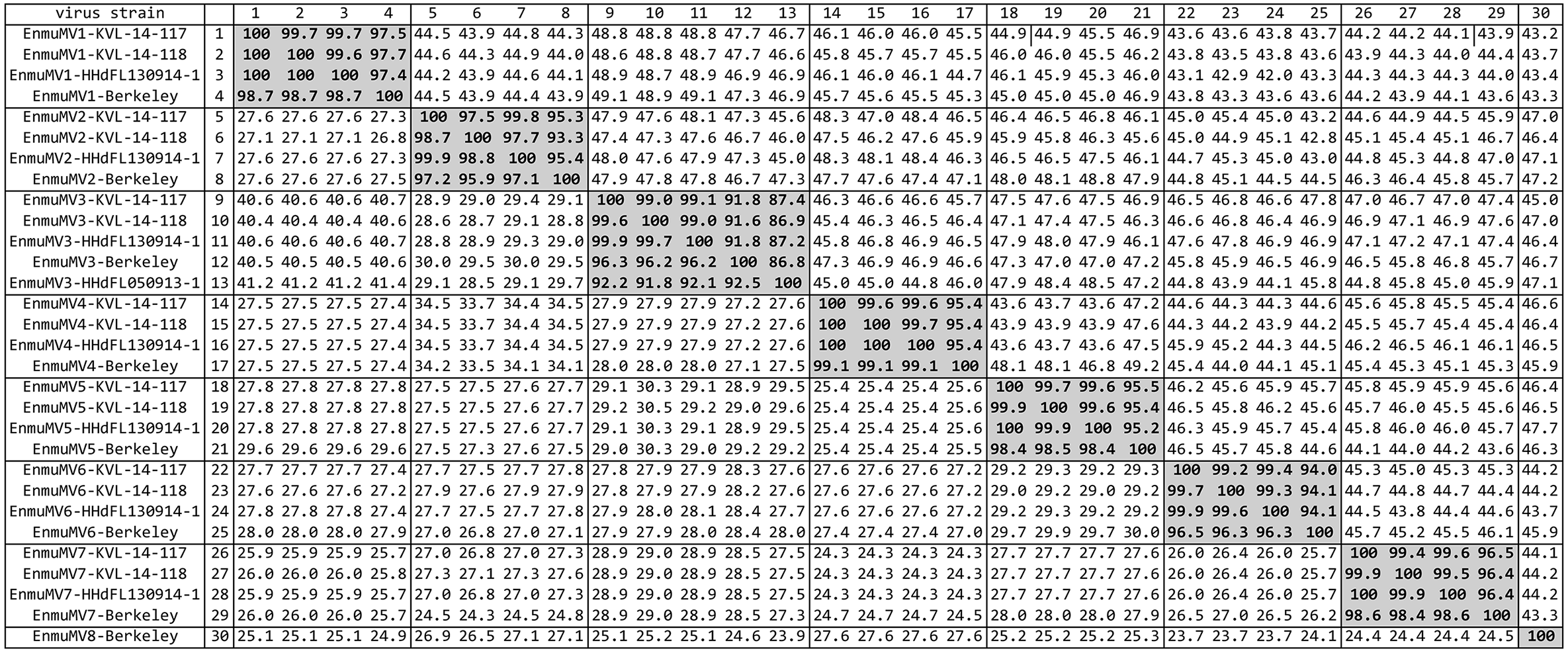

Supplement: Supplementary file 1 [file viruses-11-00351-s001.zip › Supp4-EnmuMV-FigureS2.tif]

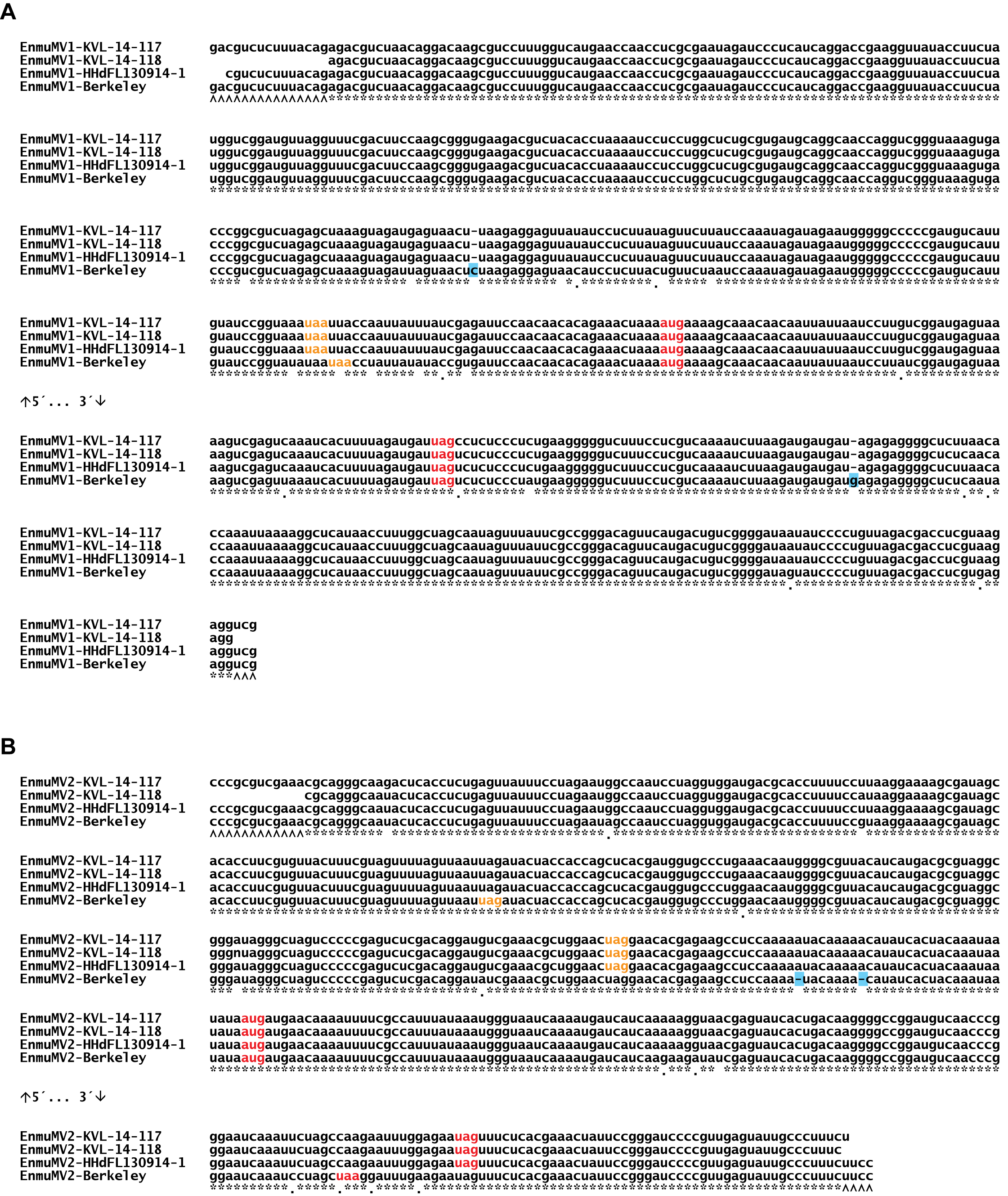

Supplement: Supplementary file 1 [file viruses-11-00351-s001.zip › Supp5-EnmuMV-FigureS3AB.tif]

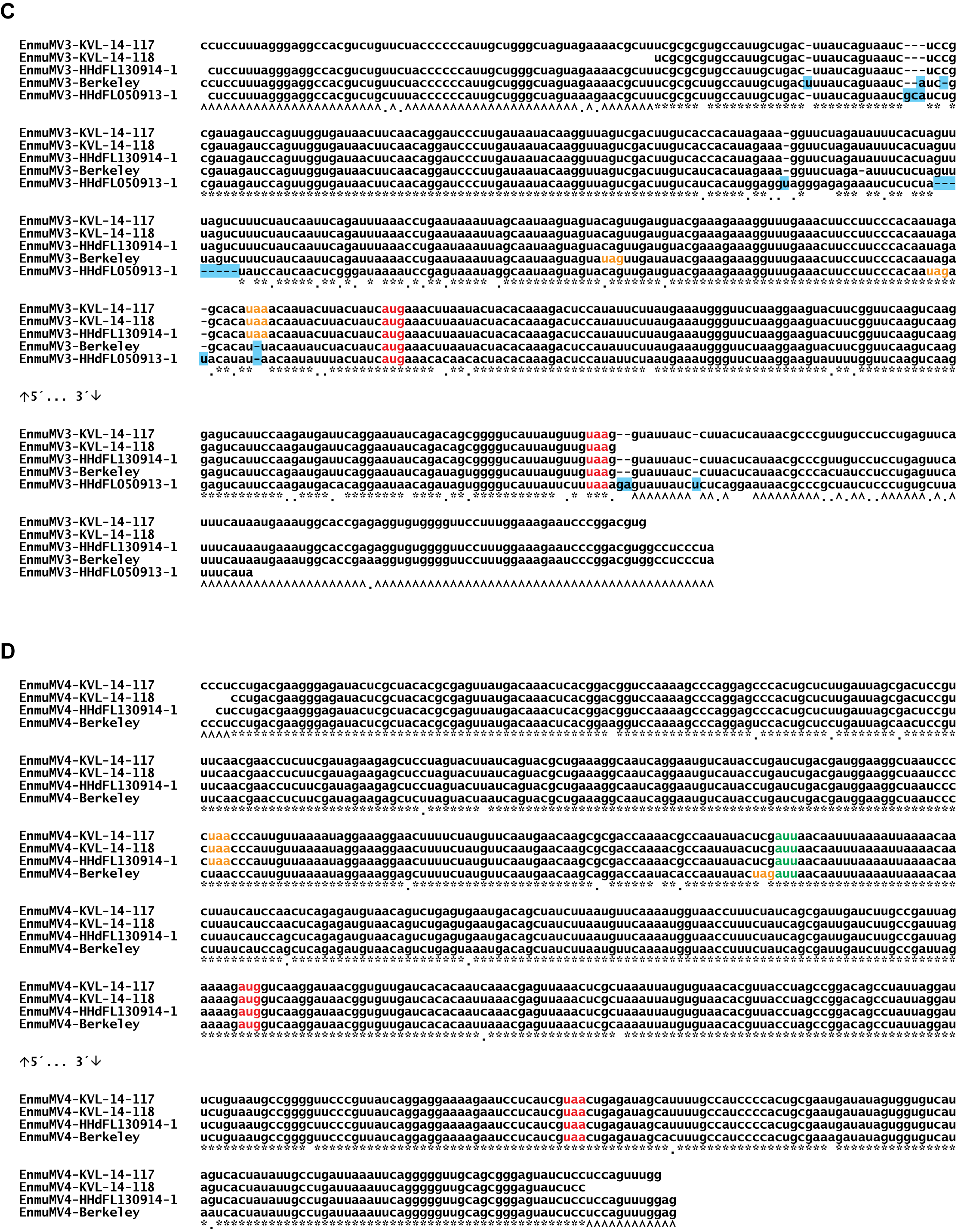

Supplement: Supplementary file 1 [file viruses-11-00351-s001.zip › Supp6-EnmuMV-FigureS3CD.tif]

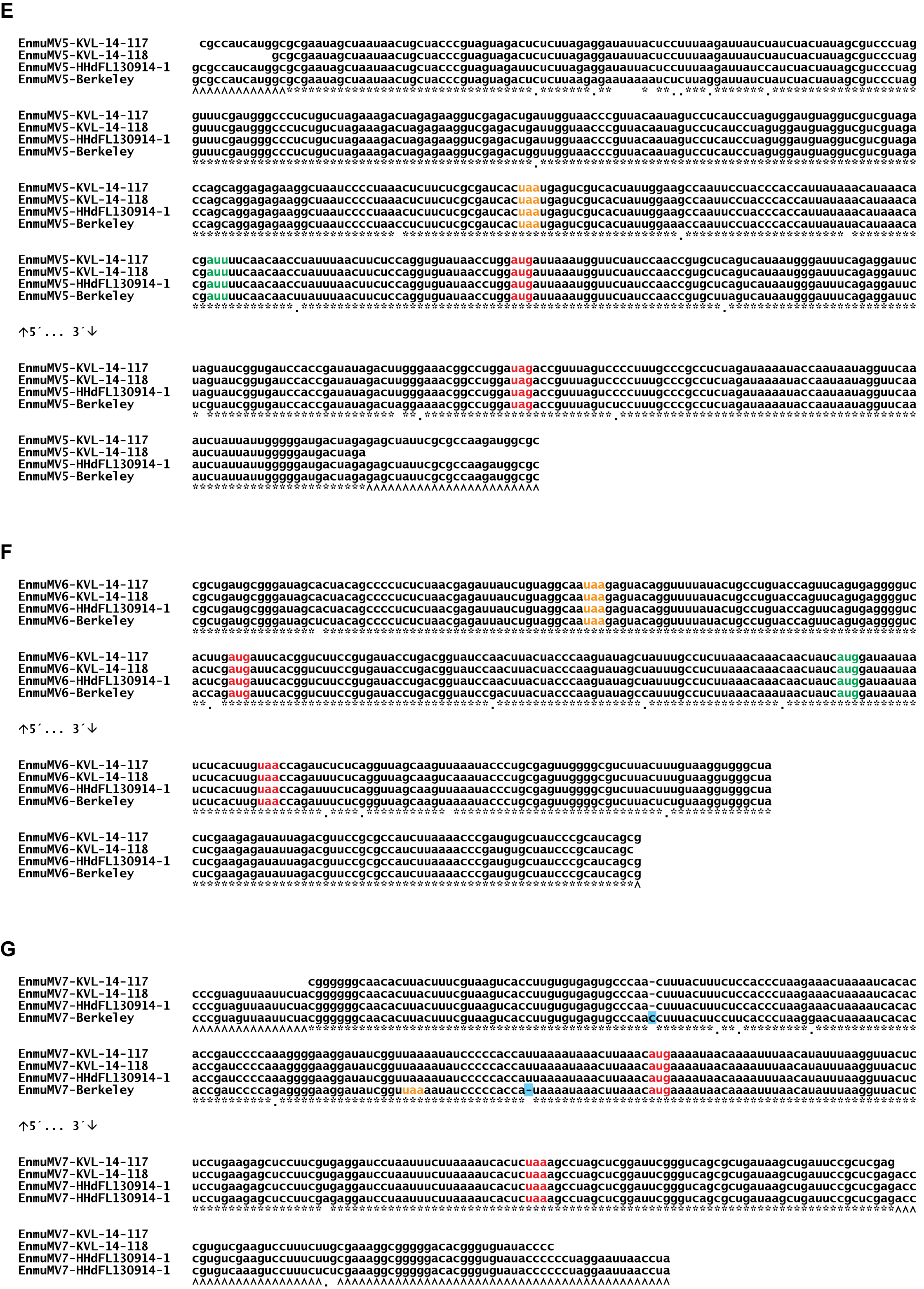

Supplement: Supplementary file 1 [file viruses-11-00351-s001.zip › Supp7-EnmuMV-FigureS3EFG.tif]

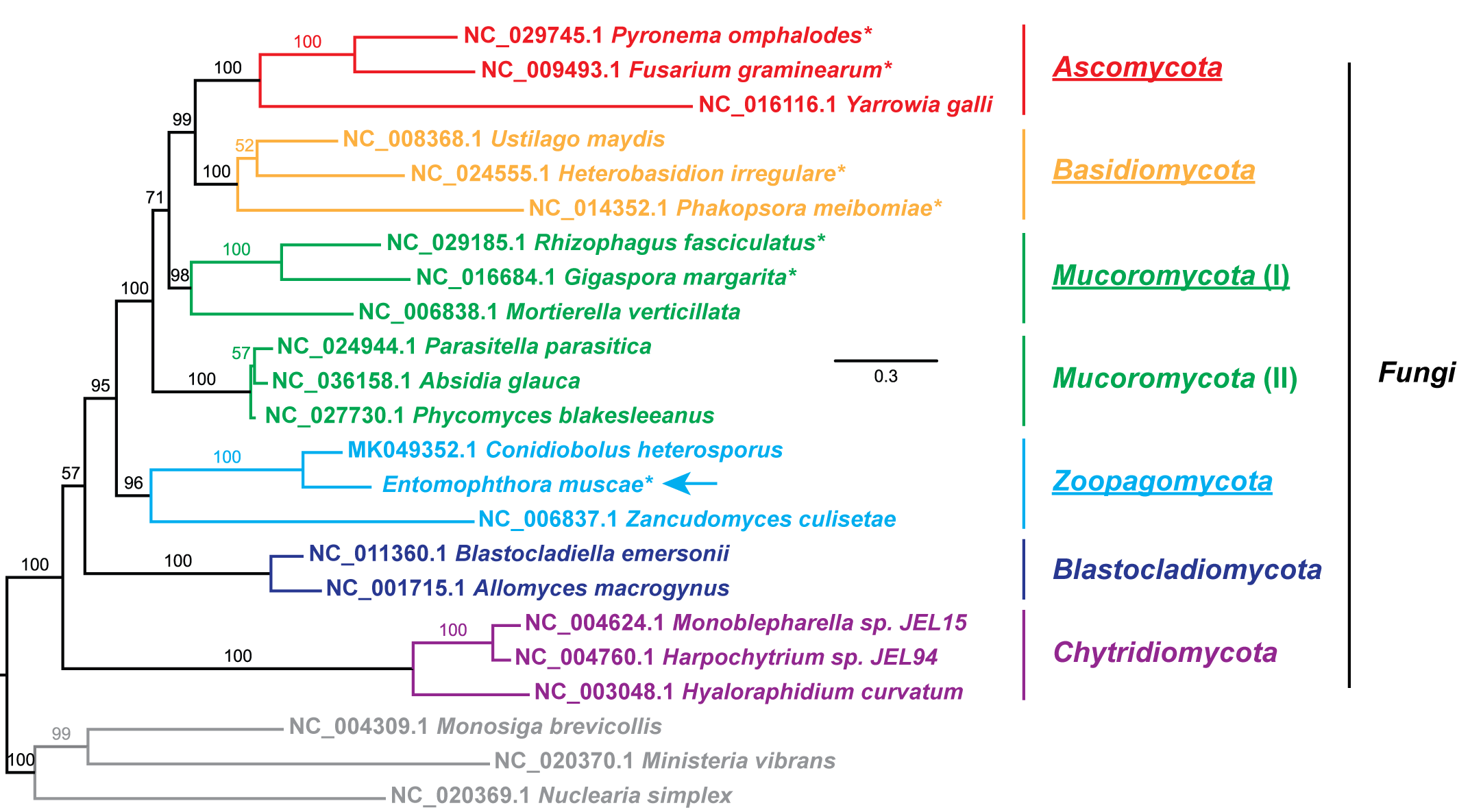

Supplement: Supplementary file 1 [file viruses-11-00351-s001.zip › Supp8-EnmuMV-FigureS4.tif]

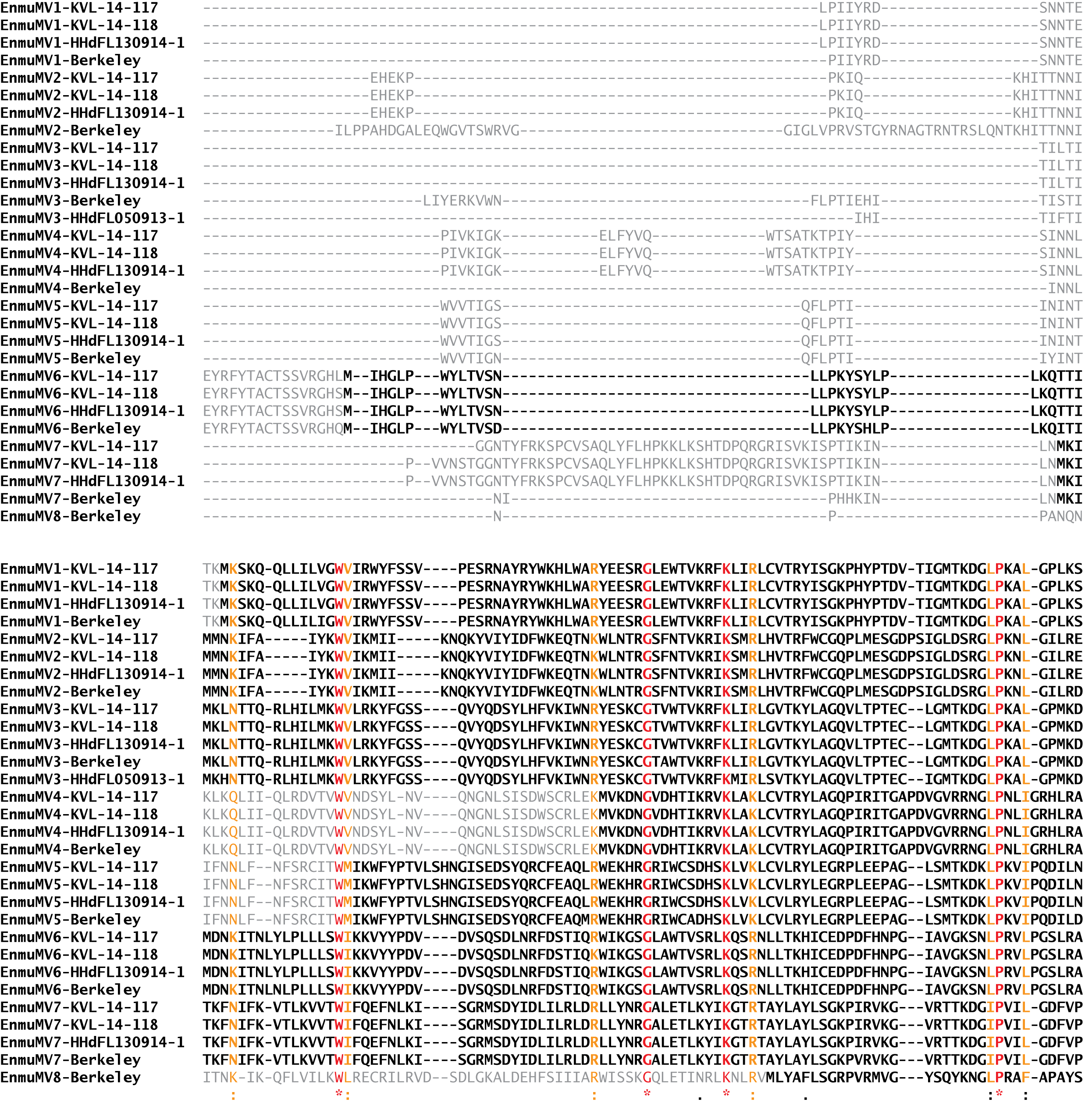

Supplement: Supplementary file 1 [file viruses-11-00351-s001.zip › Supp9-EnmuMV-FigureS5.tif]
